# Integrated computational and experimental design of fluorescent heteroatom-functionalised maleimide derivatives†

**DOI:** 10.1039/d4sc04816d

**Published:** 2024-10-29

**Authors:** Jake E. Barker, Gareth W. Richings, Yujie Xie, Julia Y. Rho, Calum T. J. Ferguson, Rachel K. O'Reilly, Scott Habershon

**Affiliations:** a School of Chemistry, The University of Birmingham, University Rd W Birmingham B15 2TT UK r.oreilly@bham.ac.uk; b School of Medicine, Shanghai University Shanghai P. R. China; c Department of Chemistry, University of Warwick Coventry CV4 7AL UK S.Habershon@warwick.ac.uk

## Abstract

The bottom-up design and synthesis of organic molecular species with tailored photophysical properties stands as an important challenge to both computational and experimental chemical science. Overcoming this challenge presents the potential to usher in new tools and approaches to improve our ability to develop new technologies, such as molecular sensors and attuned molecular switches. Here, we report the bottom-up design and characterisation of new fluorescent maleimide derivatives using coupled computational and experimental insights. Using an extensive set of experimentally-measured UV/visible spectra for different functionalized maleimides in different solvents, we train an artificial neural network (ANN) to rapidly correlate maleimide structure (and solvent) with emission spectra characteristics. We subsequently use this computational predictor to identify design principles for novel functionalised maleimide structures with targeted photophysical properties; synthesis and characterisation of several new maleimides demonstrates how this combined strategy can offer new directions for tuning photochemistry, for example opening new routes to designing tailor-made fluorescent probes.

## Abstract 2

Combining *in silico* and experimental approaches to obtain new maleimide derivatives.

## Graphical abstract

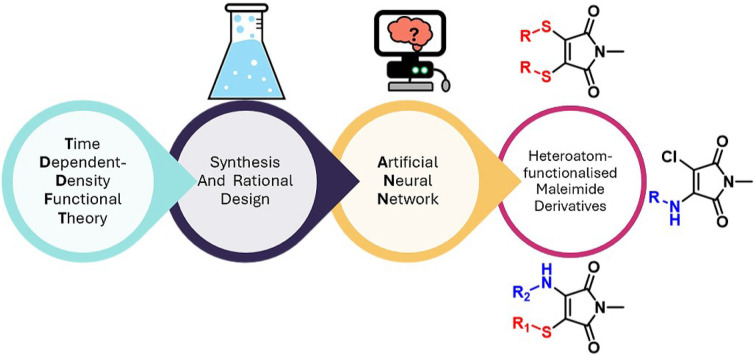

## Introduction

Maleimides have displayed great utility in a multitude of applications, such as photovoltaics, OLEDs, electrochromic materials, coatings, vulcanising agents, immunoconjugates, fluorescence quenchers, and biosensors.^1–4^ Maleimides are attractive fluorophores for a wide range of applications, most notably as fluorescent probes of biological cells, primarily because they possess coupled intramolecular donor and acceptor moieties in close proximity, affording access to photophysical and photochemical properties in the UV/visible (UV/vis) region of the electromagnetic spectrum. As fluorophores, maleimide derivatives can exhibit a large Stokes shift (>100 nm), high quantum yields, and strong solvatofluorochromism.^5^ The inherently small size and solvent-responsive photophysical profile of maleimides^6–10^ allow for distinct differences in photophysical properties to be observed in different microenvironments, such as non-polar and polar (protic) settings, as a result of excited-state solvent stabilisation effects. Combined with their synthetic accessibility and biological compatibility, these interesting (and tuneable) photochemical features mean that maleimides are an excellent candidate for developing new fluorophores for imaging applications.^11–14^

**Fig. 1:**
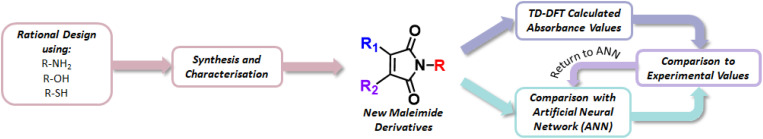
Process of synthesising and characterising new maleimide derivatives with computational evaluation of photophysical properties.

Interestingly, from the viewpoint of molecular design, the maleimide system offers versatile functionalisation at three different sites on the core scaffold (Fig. 1) – specifically, the alkene carbon atoms and the imide nitrogen atom.^15,16^ Functionalisation at these three sites offers a key route to controlling photochemical properties in maleimides; notably, functionalisation at the alkene position impacts the donor region of the maleimide scaffold, whereas imide functionalisation can influence the acceptor region of the carbonyl groups. Incorporation of single alkoxides and amines onto the maleimide alkene typically leads to functionalised maleimides that exhibit relatively-low absorbance wavelengths around the 350–380 nm range and corresponding emission wavelengths around the 450 nm region, with fairly consistent Stokes shift values.^15^ Thiol incorporation, *via* mono or di-substitution, has led to the synthesis of maleimide derivatives with an approximate 30 nm bathochromic shift, leading to an absorption window of around 370–400 nm.^17–19^ Further derivatives that feature expansion of the conjugated π-system have resulted in increased bathochromic shifts and emission peaks exceeding 500 nm.^20^ A disadvantage is that these maleimide systems are typically more complex and rely on the interactions of larger, often polyaromatic, moieties.^20–22^ Together, these results demonstrate the potential tunability of maleimides; however, there is a clear demand to enable more targeted design and tuning of maleimides with specific photoproperties (*e.g.* absorption/emission wavelengths) in mind.

As in many molecular design challenges, the ideal workflow would be to develop clear insight into the correlation between the molecular structure and photophysical properties of the target maleimides, allowing direct targeted design before synthesis and further characterisation is undertaken. One route to achieving this molecular-level insight is to synthesise and characterise a large library of maleimide structures and to characterise their photophysical properties in order to build up a picture of the intersection of maleimide chemical-space and photochemistry. While such a “high throughput” strategy is increasingly popular – for example in the exploration of pharmaceutical components – important challenges remain in rolling-out such an approach to systems such as maleimides. For example, given the presence of different functional groups on the maleimide core – including carbonyl groups, an alkene and an imide – the synthesis of varied derivatives requires careful control and development of appropriate reaction schemes and reactive conditions (as will be highlighted below, and in the corresponding ESI†). Furthermore, the inherent molecular complexity of maleimide derivatives can similarly introduce challenges in terms of purification, characterisation and analysis. Finally, the solvent-dependent photophysics of maleimides indicates a further challenge to high-throughput synthesis, where solvent typically serves as a simple reaction medium, rather than a key part of the observable target property.

In this Article, we adopt a different approach from a solely-experimental strategy – instead integrating information from *ab initio* electronic structure calculations, artificial intelligence/machine-learning (AI/ML) tools, *and* synthesis and characterisation – in order to explore the chemical space of functionalised maleimides in the search for novelty and tuneability in photophysical properties. Specifically, we develop a two-pronged computational strategy – incorporating both AI/ML and *ab initio* validation – to provide guidelines for interesting domains of maleimide chemical space to explore for target photophysical properties. Subsequently, direct synthesis and experimental characterisation enables assessment of computational predictions *and* exploration of new maleimide photophysics. Importantly, access to experimental verification enables us to “close the loop” by feeding back experimental findings into improved AI/ML models.

Fig. 1 summarizes the overall computational/experimental strategy employed here. First, using a dataset of 64 functionalised maleimides, that have been previously synthesised and characterised through spectral measurements in the UV/vis region, for 258 total inputs (Table S1†). We build an artificial neural network (ANN) architecture that relates maleimide structure (defined *via* Extended Connectivity Fingerprints [ECFPs], also referred to as Morgan fingerprints) and solvent (characterised by dielectric constant) to spectral properties such as wavelengths of maximum emission or absorption. This ANN offers a simple route to predicting photophysics given maleimide molecular structure alone – offering a straightforward “first assessment” of the photophysics of novel maleimides before synthesis. Second, proposed novel maleimide structures are then initially screened using the ANN; prospective molecular structures that exhibit good performance against a target metric – such as longer-wavelength fluorescence emission maxima – can be rapidly identified for synthesis and characterization, as well as being further analysed using time-dependent density functional theory (TD-DFT) calculations. Finally, we note that the availability of new experimental characterization (*e.g.* fluorescence measurements) for newly synthesised maleimide structures in this work can subsequently be fed back into the ANN to improve predictive capabilities and guide identification of further synthetic targets.

As we show below, the outcome of this “computational filtering” strategy is the generation of a family of functionalised maleimides that are subsequently synthesised and characterised; in spirit, this approach can be viewed as comparable to the “lead development” of traditional computer-aided drug discovery. Here, this bottom-up approach leads to development of previously-underexplored families of aminomaleimides and thiomaleimides as possible future vehicles for targeted photofunctionality in molecular-based imaging applications.

## Methods

In order to perform the TD-DFT calculations, initial molecules were drawn in Avogadro^23^ and locally optimised using the in-program *steepest descent* algorithm, before TD-DFT calculations were performed at B3LYP 6-31G** level using ORCA version 5.0.3.^24^ Furthermore, the Tamm–Dancoff approximation is applied as standard in ORCA. The ANN was constructed using *SciKit-Learn*,* and has additionally been made available as a Jupyter* notebook part of the online ESI.†

The particular combination of exchange–correlation functional and basis set employed here represents – in our experience – a balance of accuracy and cost that is appropriate for this study. Specifically, initial test calculations comparing the TD-DFT-predicted absorption maxima to some known experimental maleimide UV/vis spectra suggested that B3LYP/6-31G** demonstrated a sufficiently reliable level of accuracy as to be useful in this study as a validation of our ANN predictions. This is further highlighted in the ESI,† which presents results of comparison between B3LYP/61-31G**, PBE0/def2-TZVP and ωB97X-D/aug-cc-pVDZ exchange–correlation functionals, where it is shown that B3LYP/6-31G** demonstrates a good level of predictive capability (despite its well-known drawbacks in some settings). We acknowledge that there are certainly further combinations of exchange–correlation functional and basis sets that improve the general accuracy of TD-DFT calculations beyond the approach adopted here (albeit at likely increased computational cost). However, we also emphasize that TD-DFT B3LYP/6-31G** calculations are used in this article only as a “sense check” on the predictions of our ANN – for which our chosen strategy functions well.

## Results and discussion

As a design target, we primarily focus in this paper on the combination of ANN, synthesis, characterization, and B3LYP 6-31G** TD-DFT calculations to identify novel families of maleimide derivatives, and how heteroatom functionalisation affects the established donor–acceptor architecture and subsequent absorbance/emission properties. To date, the large majority of maleimide structures that have been synthesised absorb and exhibit fluorescence in the wavelength range 350–400 and 450–500 nm respectively, primarily driven by π-π* electronic transitions centred on the maleimide core.

As noted above, functionalization of the maleimide core offers a synthetically-accessible route to tuning the spectral characteristics; seeking to identify heteroatom-functionalised maleimides with distinct photophysical properties therefore serves as a useful molecular-design target that is readily evaluated by both computation and experiment alike.

With this design target in mind, we first describe the data-scraping/cleaning strategy used in generation of an initial experimental dataset of maleimide spectral properties. Next, we describe the training, testing, and performance of an ANN trained to predict the excitation wavelengths for maleimide structures in a variety of solvents. Guided by the predictions of this ANN, we subsequently present the synthesis and characterization of 18 new maleimides in attempts to identify maleimide classes with longer fluorescence emission-maxima wavelengths. Rather than relying solely on ANN predictions, we also study the spectral and structural properties of the newly-synthesised maleimides using B3LYP 6-31G** TD-DFT calculations in implicit solvent; as discussed below, these calculations are central in cross-validating ANN and *ab initio* calculations, and enable us to identify systematic shortcomings in both approaches. We conclude by highlighting the key structural properties of the novel classes of maleimides considered here, and offer some further routes towards integrated computational/experimental design of photofunctional molecules. We include the Kohn–Sham orbital projections, and B3LYP 6-31G** TD-DFT geometries of each new maleimide derivative in our ESI (Fig. S103–S120†).

### Dataset for maleimide spectral properties

First, experimental information regarding fluorescent maleimide derivatives were collected from the literature into a ML training set.^2,15,17,19–22,25–39^ A total of 258 maleimide examples were manually scraped from literature, including a range of maleimide derivatives in multiple solvents. Specifically, we incorporated experimental data into our ML dataset if the maleimide structure, wavelength of maximum fluorescence excitation, emission (*λ*_em_), and the corresponding solvent were all clearly reported.

**Fig. 2:**
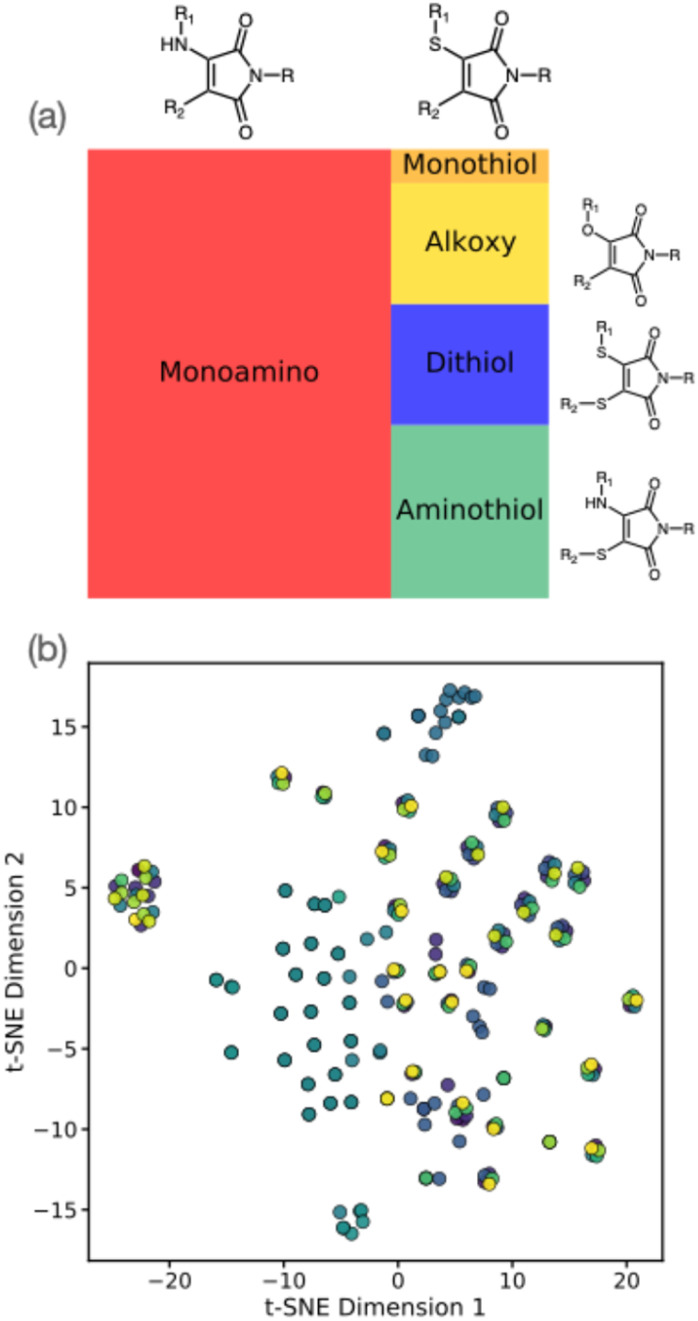
(a) Tree plot showing functional group distribution in the maleimide training dataset generated by literature search. (b) t-Distributed stochastic neighbour embedding (t-SNE) analysis for the maleimide dataset; colours correspond to different solvents.

Fig. 2(a) summarizes the distribution of molecules included in our ML training dataset. Based on our literature search, this dataset generally comprises simple alkoxy-, alkylamino- (AMs), thioalkylamino- (ATMs), and thiol-containing maleimides (DTMs), with the latter three classes comprising the majority of examples due to higher prevalence in the literature of prior synthesis and characterisation. Notably, our literature dataset shows that alkylaminomaleimides display an approximate 35 nm bathochromic shift – as well as higher quantum yields – when compared to their alkoxy-counterparts, which may explain their relatively higher incidence in literature sources. Furthermore, we note that our literature survey was implicitly constrained to those prior studies where photophysical properties, such as excitation/emission wavelengths and quantum yield, were reported (along with the respective solvent). We observed that diethyl ether, dioxane and methanol were the three most common solvents used for photophysical analysis in our collated literature data (Fig. S1†). Furthermore, in some cases, photophysical properties were assessed for the same maleimide derivative in different solvents; the resulting solvatochromic shifts must therefore also be accounted for in our ML models for property prediction.

The structure of our maleimide dataset is further highlighted in Fig. 2(b), showing the t-distributed stochastic neighbour embedding (t-SNE) using the Tanimoto similarity measure as an indicator of molecular similarity. As suggested by Fig. 1(a), we see identifiable clusters of structures in the lower-dimensional embedded space; as highlighted by the colour scheme, these clusters represent dataset examples with the *same* maleimide structure but with experimental emissions data recorded in different solvents. The impact of incorporating solvent is essential in describing the photophysical properties of maleimides, as described later; for now, we simply note the heterogeneous structure of our training dataset, both in terms of solvent and structure distribution. Our maleimide dataset (*i.e.* SMILES strings, literature sources, and emission/absorption properties) is available as part of the online ESI.†

### ANN for maleimide spectral property prediction

As the primary “route of attack” in exploring the photochemistry of functionalised maleimides, we used the maleimide dataset described above to build a simple ML framework to predict photochemical properties, primarily the maximum-intensity excitation/emission wavelength, *λ*_em_, solely given knowledge of the maleimide structure and the solvent.

Specifically, we used our maleimide dataset to train an artificial neural network (ANN) to relate input molecular structure and solvent parameters to emission wavelength, *λ*_em_. To encode information about the different maleimide structures, we used the well-known ECFPs as structural descriptors.^40,41^ Here, the local substructure around each atom in an input molecule is identified according to atom-based information and bond connectivity; these local sub-structure identifiers are iteratively hashed together, before the final list of integer identifiers is hashed into a fixed-length bit-string that is subsequently used as an input ML fingerprint. ECFPs have the advantage that they are readily calculated in accessible packages (such as RDkit^42^) from the SMILES strings alone and do not require knowledge of the three-dimensional molecular structure; this is a significant advantage in ML projects – such as this one – in which the three-dimensional structure of all relevant training examples are not readily available due to extraction from a wide range of literature sources spanning different reporting requirements. More generally, ECFPs are well-known to provide a simple and robust fingerprint in studies targeted at predicting the properties of small-molecule systems; recently, these fingerprints have also been employed in predicting the properties of chemical reactions – primarily activation energies or reaction energy changes.^43–45^ In the context of photophysical predictions, where one might reasonably expect modification of functional groups to play a more significant role than conformational change, the ECFPs used here offer a valid and useful initial approach to develop a qualitative ANN for property prediction.

In all the ANN calculations reported here, we used an ECFP length of 1024 bits, and a sub-structure (bond-radius) length of 5. Further changes to these parameters were found to result in marginal improvements in predictive capability; as a result, extensive hyperparameter optimization was deemed unnecessary for this study.

We employed a standard feed-forward ANN to learn the relationship between molecular structure and *λ*_em_. For each molecule, the input layer comprised the 1024 bit ECFP vector, in addition to a floating-point value representing the dielectric constant of the corresponding solvent; the output layer comprised a single value corresponding to the predicted *λ*_em_. The description of the solvent directly through its dielectric constant is clearly an approximation that is intended to capture the overall (averaged) environmental effect of the solvent, in much the same way as implicit solvent models do in *ab initio* electronic structure calculations. We anticipate that finer, molecular level details – such as the specific impact of hydrogen-bonding to different functional groups in maleimide derivatives – will only be captured by our ANN in the broadest sense. Nevertheless, the results below clearly demonstrate that even this simple level of solvent description is often sufficient to capture key experimental solvatochromic shifts, especially when compared against TD-DFT calculations.

In all calculations reported here, we used an ANN configuration with a single hidden-layer comprising 250 nodes. The standard rectified logistic unit (ReLU) activation function was used throughout. Testing with different ANN set-ups – for example by increasing the number of hidden-layers or modifying the number of nodes per layer – did not appear to significantly improve ANN performance. Furthermore, we note that further assessments of alternative ML strategies, primarily Gaussian Process Regression (GPR), also gave a similar level of predictive performance as demonstrated by our ANN below.

**Fig. 3:**
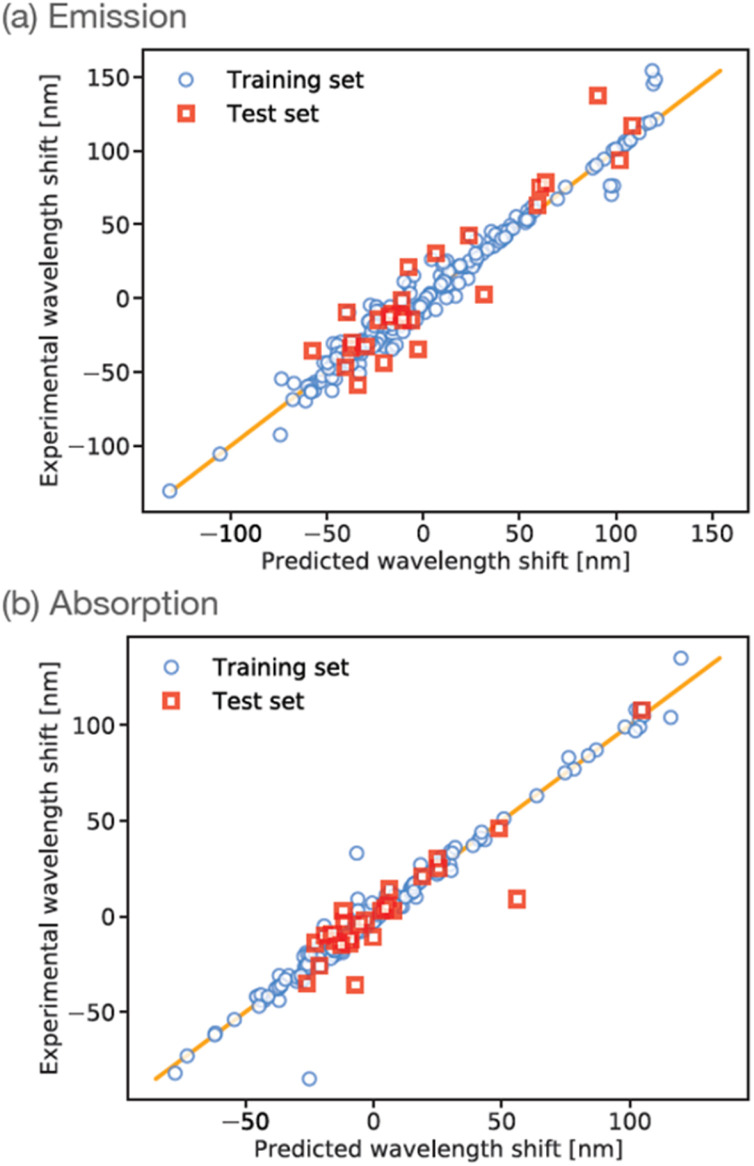
ANN prediction correlation plot emission wavelengths for maleimide training-set (blue circles) and test-set (red squares). The ANN predictions and experimental wavelengths are given relative to the mean of the experimental dataset. Results are shown for prediction against (a) wavelength of maximum emission, and (b) wavelength of maximum absorption.

The training data were randomly split into training (90%) and test (10%) sets, and the ANN was trained for a maximum of 2000 iterations using stochastic gradient descent. Correlation plots for the training and testing datasets are shown in Fig. 3(a). The *R*^2^ correlation coefficients-of-fit for the training set were typically found to be 0.96, whereas the *R*^2^ value for the smaller test-set was typically around 0.87. In our experience to date, these levels of performance are representative of small-molecule property predictions based on ANNs using ECFP descriptors, especially for the relatively small dataset considered here. We note that our initial ANN training used a simple train/test split due to the relatively small amount of available training data. However, to test the impact of possible over-fitting, we have also repeated the training using an additional validation set comprising 10% of the total available data; as shown in the ESI,† we find that this results in an ANN with comparable performance relative to our original ANN (albeit slightly reduced, likely as a result of reduced training data).

Training an ANN using experimental data describing wavelengths of maximum emission is extremely useful from the point of view of screening candidate molecules with different emission properties, as described below. In particular, we note that direct calculation of emission spectra using *ab initio* methods is typically more challenging than the corresponding calculation of absorption; put simply, a good first ‘stick spectrum’ approximation of absorption can be obtained by geometry optimization on the ground-state potential energy surface, followed by calculation of the relative energies of excited states within the Franck–Condon approximation. In contrast, a comparable calculation of emission would require excited-state geometry optimization as a starting point – which is typically more difficult to perform reliably when compared to ground-state geometry optimization.

However, in order to enable further validation and comparison between experiment, ANN predictions, and *ab initio* theory, it is of course useful to similarly train an ANN to predict absorption wavelengths. This is readily achieved by simply changing the training targets in our ANN to focus on prediction of the wavelength of maximum absorption *λ*_abs_, with all other aspects of our ANN set-up remaining the same. The results of this absorption-data fitting are shown in Fig. 3(b). Given the similarity of the problem to ANN prediction of *λ*_em_ it is unsurprising that the overall performance of this ANN is comparable to the case of emission data; for example, we find *R*^2^ = 0.97 and 0.81 for the training- and test-sets, respectively. This absorption-based ANN provides a useful touchpoint of validation against *ab initio* calculations, but also provides insight into the treatment of solvent effects for maleimides, as described in more detail below.

### Novel maleimide synthesis: first iteration

After initial testing of our ANN, we moved on to investigating the synthesis and photophysical characterisation of new maleimide structures, using the ANN – combined with additional *ab initio* evaluations as described below – as a guide to influence selection of new structures for exploration. Here, we initially used the literature training set as inspiration for the synthesis of chemically similar structures – leading us to synthesise and characterize three different chloro-aminomaleimides, as described below.

However, before proceeding to discuss new maleimides and their photophysical properties, it is useful to first consider the evaluation of absorption properties for a series of known maleimides using standard TD-DFT approaches; this serves as a useful future comparison to ANN predictions. Here, ground-state geometry optimization (using the B3LYP exchange–correlation functional and a 6-31G** basis set) was first performed, after which the vertical excitation energies to the first 20 excited electronic states were evaluated; the excitation with the largest predicted oscillator strength was subsequently chosen as the predicted wavelength of maximum absorption. These calculations were performed for a representative series of 14 different maleimide structures drawn from our constructed literature dataset. In all cases, the B3LYP 6-31G** TD-DFT calculations were performed *in vacuo*, whereas all experimental measurements were performed using diethyl ether as solvent. The reasons for this will become clear in the discussion below on solvent-dependent absorption and emission wavelength prediction; however, we also note that the low dielectric constant of diethyl ether (*ε* = 4.33) means that this comparison against vacuum simulations is reasonably well-founded.

We note that our TD-DFT/B3LYP/61-31G** calculations were performed for a single conformer of each maleimide considered; this structure was generated ‘by hand’ based on standard steric considerations. To test the impact of conformational flexibility on absorption spectra predictions by TD-DFT/B3LYP/61-31G**, we report in the ESI† further analyses of the impact of conformational flexibility for three of the maleimides synthesized later. Here, we find that typical spectral shifts are of the order of 7–19 nm, certainly comparable to the uncertainties associated with the ANN.

**Fig. 4:**
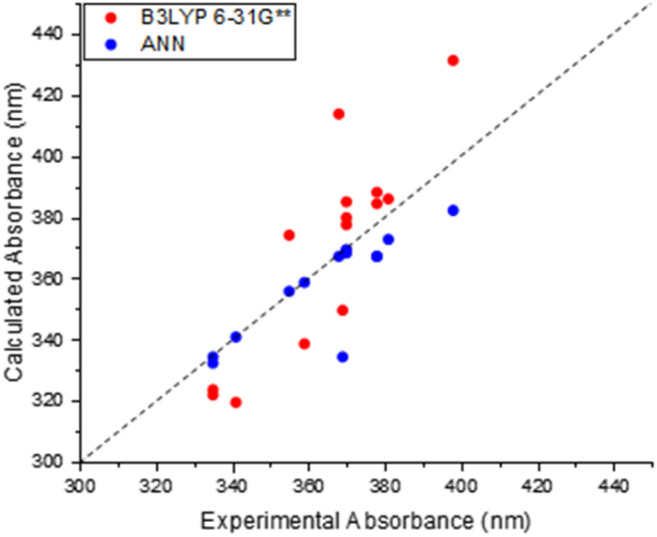
Relationship between calculated TD-DFT (B3LYP 6-31G**) and experimentally determined wavelengths of previous maleimide derivatives. (Present in training set).

The results of this comparison between TD-DFT and experimental absorption measurements are shown in Fig. 4. The straightforward conclusion is that B3LYP 6-31G** TD-DFT can capture some of the structural dependence in absorption properties, although the correlation and agreement are not as good as that noted for our trained ANN model (Fig. 3(b)). For example, the greatest scatter about the “zero line” is observed for molecules with higher excitation wavelengths, with the lowest scatter and thus greatest performance observed around 380 nm. The method is still able to predict the corresponding values to within 20 nm of the experimental counterpart. These results therefore serve to show that TD-DFT is a broadly useful methodology for directly predicting absorption properties of maleimides, but one must bear in mind the corresponding uncertainties – and the additional computational cost when compared to ANN predictions.

**Fig. 5:**
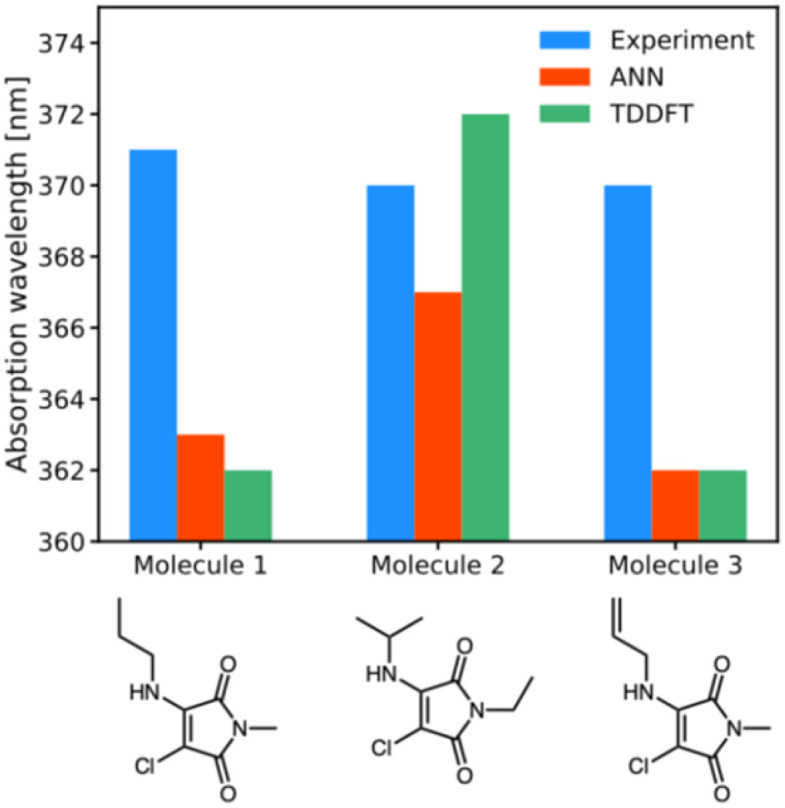
Experimental (blue), ANN (red) and TD-DFT (green) predictions of wavelengths of maximum absorption for molecules 1a, 1b, 1i.

Following validation of both B3LYP 6-31G** TD-DFT and ANN predictions against experimental absorption data, we subsequently chose to synthesise and characterise three new aminomaleimides (Fig. 5) in order to further compare and contrast ANN and TD-DFT absorption predictions. These three particular maleimides were chosen as a starting point for synthesis because they were representative of the largest maleimide class in the literature dataset. Furthermore, the chlorinated aminomaleimides 1–3 could all be readily synthesised and purified in reliable yields. Further details of synthesis and characterisation are given in the ESI;† here, we focus on photophysical properties.

For the three newly-synthesised maleimides in Fig. 5, we subsequently measured the wavelength of maximum absorption using a UV-visible spectrophotometer (see ESI† for further details) using diethyl ether as solvent. Similarly, for the same solvent, we performed ANN predictions and TD-DFT calculations (using the CPCM implicit solvent model) of the wavelength of maximum absorption, with the results shown in Fig. 5.

The results of Fig. 5 are somewhat underwhelming but offer some initial insights into ANN and B3LYP 6-31G** performance. First, it is noteworthy that there is almost no difference in *λ*_abs_ across the three different maleimides studied. Clearly, mild substitution with alkyl or alkenyl functional groups on the substituted amino position makes little difference to the photophysics; this is unsurprising, given that the conjugated ring system of the maleimide core – which is primarily responsible for absorption in such systems – is little altered by such substitutions. The predictions by the ANN show similarly small structural change, varying between 363 nm and 367 nm, whereas the B3LYP 6-31G** calculations show a larger variation of 10 nm. However, an important point is that experimental results all lie within the estimated uncertainties of the TD-DFT and ANN predictions, at least based on our analysis of our literature dataset. In other words, our ANN is as accurate as comparable B3LYP 6-31G** predictions in these examples, but runs at a fraction of the computational cost.

However, the predictive power of our trained ANN really appears when one considers solvent effects. Specifically, our ANNs were trained using both maleimide structural information and solvent dielectric constant as input, such that the ANN learns some aspects of the impact of solvent from the input experimental training data. In contrast, TD-DFT calculations of photophysical properties of maleimides in explicit solvent environments are too expensive to routinely consider, and one is forced to instead consider implicit solvent models as a route to capturing solvent influence.

To assess the ability of our ANN and B3LYP 6-31G** to capture solvent effects, we experimentally measured the absorption spectra of maleimides 1, 2 and 3 in a range of solvents with a range of different dielectric constants. For each maleimide, we considered several solvents spanning hexane (with a low dielectric constant of 1.88) to acetonitrile and methanol (with high dielectric constants of 37.5 and 32.7, respectively). Corresponding predictions of *λ*_abs_ were made using our ANN (changing the input dielectric constant as appropriate) and using TD-DFT; in the case of the *ab initio* calculations, we performed calculations using CPCM as an implicit solvent model, which is commonly used in such calculations.

**Fig. 6:**
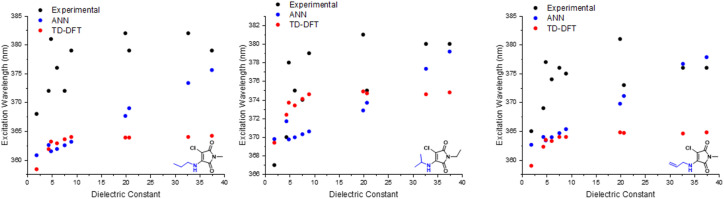
Experimental *vs. in silico* predictions of excitation wavelengths of 1a, 1b & 1i.

The results of this comparison are shown in Fig. 6. For each of the three aminomaleimides considered here, we find that increasing the dielectric constant of the solvent environment leads to an increase in *λ*_abs_. In general, increasing the dielectric constant of the solvent shifts *λ*_abs_ from roughly 365 nm at low *ε* to up to nearly 385 nm for *ε* > 30. These experimental observations indicate that a more polar solvent reduces the energy gap between ground and excited states.

Turning to the ANN results for the three aminomaleimides, we find that the predicted *λ*_abs_ show reasonably good agreement with the experimental solvent-dependence for all three molecules. For example, the increase in *λ*_abs_ upon moving from low to high dielectric constant is around 15 nm in the ANN, comparable to the change observed in experiment, although we note that the absolute absorption wavelengths appear to be systematically underestimated in the ANN (following Fig. 6). Despite this, it is clear that the ANN captures the correct experimentally-observed solvent dependence on *λ*_abs_.

The same cannot be said of the B3LYP 6-31G** results. In this case, regardless of the different maleimides considered or the different implicit solvent model used, we find the same solvent-dependent trend in the B3LYP 6-31G**-predicted *λ*_abs_. In particular, all of these TD-DFT results show a slight increase in *λ*_abs_ of about 5 nm as the solvent dielectric constant is increased up to about *ε* = 10 and, beyond this point, there is effectively no further change in the predicted values of *λ*_abs_. Similar results in vertical-excitation calculations of absorption have been noted in previous analysis of *ab initio* calculations,^46^ with this effect noted to arise due to the absence of both solvent structural relaxation and excited-state charge density relaxation in implicit solvent models.

At higher polarities, the maleimide architecture can strongly interact with solvent molecules, primarily through hydrogen bonding. With explicit interactions such as these, these systems should ideally be modelled as clusters and/or complexes, as modelling these systems should improve the agreement between the experimental and calculated values. We also acknowledge that the continuum solvation models come with their own drawbacks and limit their applicability in aqueous systems, as outlined by Ding and coworkers.^47^

The results of Fig. 6 therefore provide a useful ‘optimal use case’ for ANNs in the context of photophysical property prediction. While both ANN and B3LYP 6-31G** perform similarly for the case of *λ*_abs_ prediction in vacuum and/or weakly-interacting solvents with low dielectric constants, B3LYP 6-31G** using implicit solvent models fails to correctly describe the solvent dependence of *λ*_abs_ for the three maleimides studied here. Instead, these TD-DFT results in implicit solvent predict that *λ*_abs_ reaches a plateau value once *ε* > 10, in contrast to the experimental observations that show a continuing increase as *ε* is increased. On the other hand, the ANN predictions do correctly predict the broad increase in *λ*_abs_ as *ε* increases. This result is clearly important in the current context of predicting solvatochromism in maleimide structures; B3LYP 6-31G** is not applicable to this use-case (and is more computationally demanding), but our ANN is. Interestingly, a common outlier is chloroform, (*ε* = 4.88), which consistently yields wavelengths surpassing other solvents of higher dielectric constants. While there are several important, additional solvent properties that can profoundly affect a molecule microenvironment, the performance of the ANN based on only one parameter captures the experimental trend admirably.

### Novel maleimide synthesis: second iteration

Inspired by the performance of the ANN, we opted to further synthesise additional maleimide derivatives, which fit into three distinct categories: AMs, DTMs and ATMs (Fig. S4, S80, and S91†). The synthesis and subsequent analysis of these would allow for further assessment of our computational toolkit performance. We expected a fluctuation in predictive performance across derivative classes as a result of training set incidence rates, with AMs comprising the majority of our training set at 66%, and the DTMs & ATMs comprising ∼26%. We hypothesised that both ATMs and DTMs would suffer a reduction in ANN predictive accuracy due to their comparatively lower incidence. We also rationalised that the B3LYP 6-31G** TD-DFT values (Fig. S37†) may diverge from experimental results to a greater extent, as in Fig. 4, we observe a greater scatter from the “zero” line at excitation wavelengths exceeding 380 nm.

**Fig. 7:**
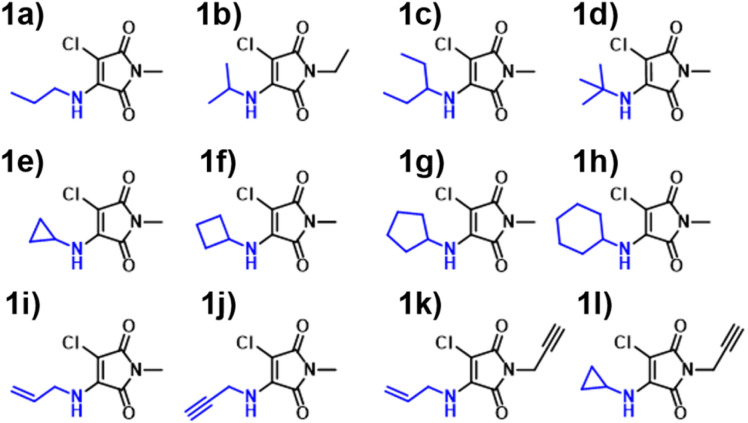
Aminomaleimides (AM) synthesised in this paper 1a–1l.

We synthesised an additional 9 AM derivatives (Fig. 7), containing structurally diverse motifs, and compared the photophysical properties against our *in silico* predictions (Fig. S19–S31†). We opted to include bulkier alkyl moieties, as evidenced by molecules 1c & 1d, along with the inclusion of previously unexplored cycloalkyl amines (1e–1h). Additionally, we sought to include unsaturated alkyl chains into this system, which was achieved with allylamine and propargylamine reagents. Our rationale behind this design choice was the absence of alkene and alkyne moieties observed in the training set. By introducing these “new” and distinct motifs into our ANN system, we hoped that the relationship between structure and imparted photophysical properties could be elucidated.

We observed a high performance of the predicted excitation wavelengths *via* the ANN (Fig. 10), despite this inclusion of structurally diverse amines, and two cases of imide functionalisation. Specifically, the ANN predictions displayed a much lower scatter about the “zero” line, with these predictions typically being within 10 nm of their experimental values, with few outliers. The poorest ANN prediction differed by 11 nm (1g). Whereas conversely, the B3LYP 6-31G** TD-DFT calculations were clustered, typically yielding values 20 nm less than the experimental value.

### DTM performance

**Fig. 8:**
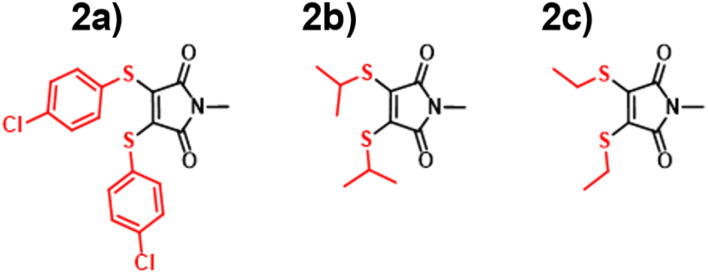
Dithiomaleimides (DTM) synthesised in this paper 2a–2c.

With the ANN proving itself as a valuable predictive tool, we wanted to test the predictive capacity of molecules structurally similar to an underrepresented class in the training set. With a composition of less than 15%, Dithiomaleimides offered an exciting opportunity to test both the ANN and B3LYP 6-31G**. The synthesis of this class of molecules (Fig. 8) was achieved *via* the use of a brominated analogue of the previous starting material used for AMs (Fig S2†). With a lower incidence rate of DTMs within our training set, we opted to employ less structurally-complex reagents for functionalisation, such as alkyl chains and aromatic groups. The resulting experimental spectra (Fig. S84–S90†) display an average absorbance wavelength around 410 nm.

Interestingly, contrary to the previous AM predictions, the ANN predictions of the DTM (Fig. 10) were underestimated by approximately 30 nm. We anticipated a reduction in predictive accuracy, as a result of this lower incidence, but rationalised that this ∼30 nm difference translates to a less than 10% error. The TD-DFT calculations yielded values closer to the experimental results but were typically overestimated, which is concurrent with previous TD-DFT findings of molecules exceeding 380 nm emission (Fig. 4).

### ATM performance

**Fig. 9:**
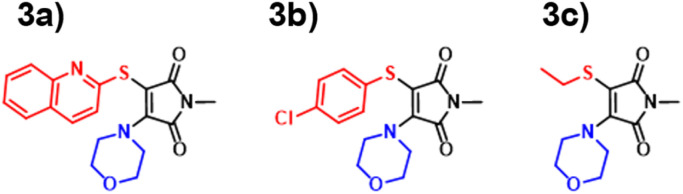
Aminothiomaleimides (ATM) synthesised in this paper 3a–3c.

**Fig. 10:**
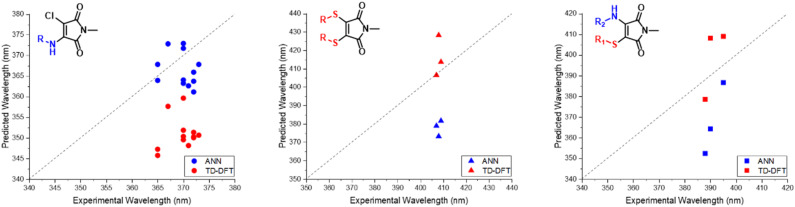
Experimental *vs.* TD-DFT and ANN absorbance/excitation values of maleimide classes AMs (left), DTMs (middle) and ATM (right).

Amino-thiol-maleimides also represent an approximate 10% of the ANN training set and propose interesting photophysical properties as a result of their asymmetrical nature. Their synthesis (Fig. 9) was achieved through the mono-substitution of a chlorinated starting material, with a thiol, before addition of the corresponding amine and base (Fig. S91†). With a lower incidence, we chose to vary the thiol, whilst keeping morpholine as a consistent substituent. Morpholine is a medium strength base, with good electron donor character, thus allowing for active participation in the maleimide donor–acceptor architecture. The grouping of both the B3LYP 6-31G** and ANN values appeared closer in this case as displayed in Fig. 10. These values tended to differ from their experimental counterparts (Fig. S95†) between 20 and 30 nm and showed a greater comparability than those displayed in the DTM plot.

While these TD-DFT results indicate a relatively poor performance in some cases, it is important to acknowledge that TD-DFT methodology encompasses a large number of basis sets and functionals, which can each perform better for different, specific systems. A combination of other basis sets and/or exchange–correlation functionals could perform better. In this work, we focus on the application of B3LYP 6-31G**, a popular combination which offers moderate accuracy at a reduced comparative cost. We note that molecules 2a, 3a and 3b possess pendant aromatic functional groups that could potentially generate charge-transfer states upon excitation; indeed, closer visual analysis of the HOMO/LUMO for these molecules (see ESI†) demonstrates that molecule 3b does exhibit some charge-transfer characteristics. However, we note that the TD-DFT/B3LYP/6-31G** predictions for molecule-sets 2 and 3 are generally better than those of the ANN itself, suggesting that the treatment of charge-transfer states is not strongly impacted by the particular choice of TD-DFT/B3LYP/6-31G** employed here.

### Prediction of emission properties

The calculation of emission wavelengths using TD-DFT poses several issues, as multiple excited states can be proposed, often with variable geometries. This, coupled with an accurate prediction of these states and their subsequent relaxation to lower states resulting in emissive behaviour, remains a challenge. As our training set contained a wealth of information, in the form of various photophysical properties, we altered the ANN to read and predict the emission wavelength of our outlined maleimide derivatives. Here, after measuring the excitation and emission spectra of our synthesised maleimide derivatives (Fig. S39–S67, S88–S90, and S99–S101†) we show the performance of ANN emission predictions in diethyl ether (Fig. S70–S74 for AMs, Table S6† summary*).*

We observed that AMs typically exhibited a low scatter about the “zero” line, with the outliers being 1j and 1d, showing an approximate 15 and 25 nm deviation respectively. Interestingly, despite these molecules performing the “poorest”, these values were still within 5% of the experimentally derived emission. We hypothesise that 1d's performance and high emissivity were due to the *tert*-butyl functionalised amine, as this motif possesses strong electron donor character, and thus complements the donor/acceptor system well. Further information can be found in Table S3 and Fig. S68–S79.†

DTMs 2b and 2c displayed a very low scatter about the main line and showed comparative performance to many AMs. DTM 2a, however, presents the poorest performance in both excitation and emission predictions. The high performance of 2b and 2c's emission prediction was interesting, as we observed a fairly large deviation in the excitation case. ATMs as a class showed the greatest scatter about the main point, with a consistent ∼40–50 nm underestimation of the experimental wavelength. Therefore, we hypothesise that the ANN still does not “understand” the effect of this asymmetrical functionalisation, as other cases tend to rely on mono-amino or dithiol functionalisation.

Additionally, we further utilised the ANN to predict the solvatochromic emission of various AMs (Fig. S68–S79†) where, with five AMs, we observed a greater degree of accuracy at lower dielectric constants, similar to the excitation predictions, but a greater divergence in values at higher dielectric constants. Interestingly, these differences in values encompassed both over and underestimation, with no discernible trend apparent. We did observe, however, that methanol (the most polar solvent used) was underpredicted consistently. We rationalise that this is due to the lower dielectric constant it possesses, as this is the only changing variable the ANN considers. Conversely, in the case of four of these molecules, the emission wavelength in acetonitrile is overpredicted as, in this set of solvents, it has the highest dielectric constant, while not being considered the most polar. Interestingly, comparing these values to excitation predictions, very few cases had a positive difference, where the exceptions only differed by ∼3 nm, indicating that the emission predictions showed greater uncertainty. Reviewing the training set showed that the excitation and emission values for each molecule were typically present, thus this uncertainty could not be attributed to an absence of emission values.

## Conclusions

The design of organic molecular systems with tailored photofunctionality – such as target excitation or emission wavelengths – is a challenging and open problem. Here, by using extensive synthesis and characterisation, combined with computational predictions made by either TD-DFT or a trained ANN, we investigated how computation and experiment might be used to guide the search for target photofunction, using maleimide derivatives as our targets.

Our ANN was constructed and trained on multiple literature accounts featuring several classes of maleimide derivatives, such as those functionalised with amines, alcohols, and thiols. Each molecule was initially converted into its SMILES string to enable ECFP evaluation, and information on the absorbance/excitation wavelength, was used as training targets. Solvation information was also added to the molecular descriptor-set, (when available) to yield a training set with approximately 250 entries. The training set composition was heavily weighted towards aminomaleimide derivatives, due to their prevalence in previous literature. The remaining 33% of the training set comprised dithiolmaleimides, aminothiolmaleimides, alkoxymaleimides and mono-thiomaleimide.

An initial small set of aminomaleimide derivatives were synthesised and their absorption properties were evaluated compared to both B3LYP 6-31G** TD-DFT and ANN predicted properties; the ANN predictions generally performed well, and required only a fraction of the computational cost of TD-DFT. Through a further rational design process – and inspiration from a wealth of literature accounts present in the training set – additional maleimide derivatives belonging to three distinct functionalisation classes were synthesised and analysed in tandem with B3LYP 6-31G** screening and ANN predictions. We continued to observe good ANN performance relative to B3LYP 6-31G**, albeit noting the data-dependent nature of ANN performance. For example, the ANN performed well in all cases of aminomaleimides, with comparable performance in the mixed aminothiolmaleimides, and a poor performance of dithiolmaleimides. The poorer performances could be attributed to a smaller proportion of the training set, and thus a smaller “knowledge base” for the ANN; the further synthesis and characterisation of novel maleimides reported here will therefore enable us to build a ‘second generation’ of our ANN to improve accuracy in future predictions.

**Fig. 11:**
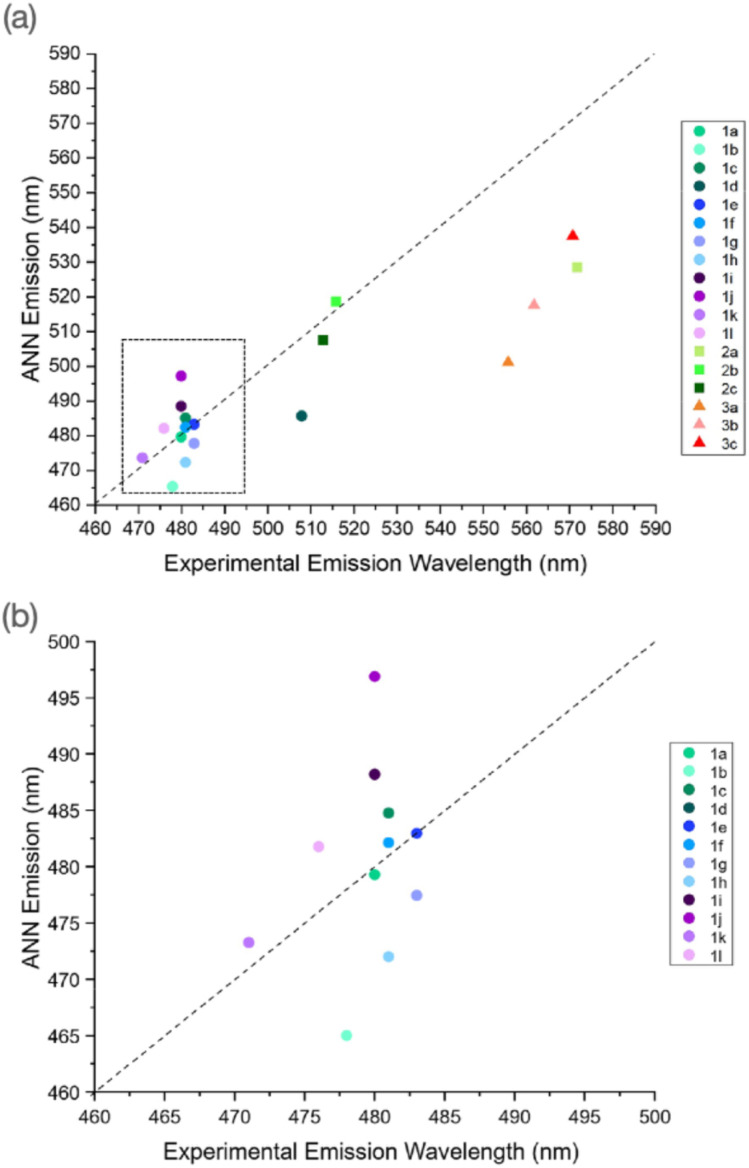
(a) Experimental *vs.* ANN emission wavelengths for maleimide derivatives synthesised in this work, (b) magnified region from (a), highlighting AM predictions in on AMs.

The ANN was further used to investigate the performance of B3LYP 6-31G** with implicit solvation models. Here, the absorbance behaviour of several molecules was characterised in a range of different solvents, and the corresponding B3LYP 6-31G** calculations were performed using the CPCM model. While the ANN predictions displayed small to moderate deviation from the experimental values, these predictions were comparable to, or better than, the implicit solvation model calculations, the wavelengths of which plateau at high dielectric constants/polarities, which contradicts the solvatochromic nature of maleimides. The ANN, using only the dielectric constant of the solvent as a fingerprint, was able to predict the experimental absorbance wavelength much closer than the B3LYP 6-31G** value. Finally, we were able to further modify the ANN to predict the emission wavelengths of our maleimide derivatives, further highlighting the scope of our ANN toolkit; TD-DFT calculations of the corresponding emission properties are challenging, requiring excited-state geometry optimization, for example (Fig. 11).

In summary, this research has highlighted the growing potential for AI/ML approaches to sit alongside synthesis and experimental characterisation in the search for designer photofunction. Data sparsity has been particularly highlighted as a challenge – but we anticipate that the experimental dataset collected here will be of use to others in this regard. Finally, we highlight the promising results obtained in predicting solvatochromic shifts using ANNs; the improved accuracy relative to implicit solvation B3LYP 6-31G** calculation is noteworthy, and we will seek to further exploit this capability in guiding future design of novel photoactive molecules.

## Data availability

The ESI† contains the maleimide training dataset, an example Python notebook for ANN fitting, and extensive information on synthesis, characterisation and quantum chemistry calculations.

## Author contributions

J. E. Barker performed synthesis, purification, characterisation and photophysical analysis of products (full characterisation and photophysical analysis are provided in the ESI†). G. Richings and J. E. Barker performed TD-DFT calculations and modelling. Y. Xie worked on previous synthesis and collation of “training set” data. J. Y. Rho and C. T. J. Ferguson revised manuscript drafts. S. Habershon performed ANN analysis. This project was supervised by both S. Habershon and R. K. O'Reilly.

## Conflicts of interest

There are no conflicts to declare.

## Supplementary Material

SC-OLF-D4SC04816D-s001

SC-OLF-D4SC04816D-s002
