# Supplementary material for: Integrated computational and experimental design of fluorescent heteroatom-functionalised maleimide derivatives
Source: Chem Sci. 2024 Oct 29;15(46):19400–10. doi: 10.1039/d4sc04816d (PMC11536195; doi:10.1039/d4sc04816d)
Supplement: SC-015-D4SC04816D-s002 [file SC-015-D4SC04816D-s002.pdf]

Supplementary Information (SI) for Chemical Science.
This journal is © The Royal Society of Chemistry 2024

# Supporting Information for: Integrated computational/experimental design of fluorescent heteroatom-functionalised maleimide derivatives

Jake E. Barker,† Gareth W. Richings,‡ Yujie Xie, † Julia Y. Rho,† Calum T. J. Ferguson,† Rachel K. O'Reilly*† Scott Habershon,*‡

† School of Chemistry, The University of Birmingham, University Rd W, Birmingham B15 2TT

‡ Department of Chemistry, University of Warwick, Coventry CV4 7SH

*Corresponding author: r.oreilly@bham.ac.uk, s.habershon@warwick.ac.uk

## Contents

Tables ... 3
Figures ... 4
Training Set & Artificial Neural Network Construction ... 9
Training Set ... 9
Training Set Sources ... 19
Training Set Solvent Composition ... 20
Synthesis and characterisation ... 21
Starting material and precursor preparation ... 21
DCM, Dichloro N-methyl maleimide ... 21
DCEM, Dichloro N-ethylmaleimide ... 21
DBM, Dibromo N-methyl maleimide ... 22
DCPM, Dichloro N-propargyl maleimide ... 22
Addition-Elimination Mechanism ... 22
General procedure for AM synthesis ... 23
**1a** (3-chloro-1-methyl-4-(propylamino)-1H-pyrrole-2,5-dione) ... 23
**1b** (3-chloro-1-ethyl-4-(isopropylamino)-1H-pyrrole-2,5-dione) ... 24
**1c** (3-chloro-1-methyl-4-(pentan-3-ylamino)-1H-pyrrole-2,5-dione) ... 25
**1d** (3-(tert-butylamino)-4-chloro-1-methyl-1H-pyrrole-2,5-dione) ... 25
**1e** (3-chloro-4-(cyclopropylamino)-1-methyl-1H-pyrrole-2,5-dione) ... 26
**1f** (3-chloro-4-(cyclobutylamino)-1-methyl-1H-pyrrole-2,5-dione) ... 26
**1g** (3-chloro-4-(cyclopentylamino)-1-methyl-1H-pyrrole-2,5-dione) ... 27
**1h** (3-chloro-4-(cyclohexylamino)-1-methyl-1H-pyrrole-2,5-dione) ... 28
**1i** (3-(allylamino)-4-chloro-1-methyl-1H-pyrrole-2,5-dione) ... 29
**1j** (3-chloro-1-methyl-4-(prop-2-yn-1-ylamino)-1H-pyrrole-2,5-dione) ... 29
**1k** (3-(allylamino)-4-chloro-1-(prop-2-yn-1-yl)-1H-pyrrole-2,5-dione) ... 30
**1l** (3-chloro-4-(cyclopropylamino)-1-(prop-2-yn-1-yl)-1H-pyrrole-2,5-dione) ... 31
NMR spectra and analysis ... 31
UV-visible Spectra ... 46
AM UV-visible spectra ... 46

AM Solvatochromism ....................53
AM Solvatochromism- TD-DFT Predicted Spectra ....................55
Fluorescence Analysis ....................57
AM Fluorescence Spectra: ....................58
AM Fluorescence Spectra- Solvatochromism: ....................60
AM Contour Plots ....................63
ANN Solvatochromism of maleimides ....................69
ANN Combination with Experimental and TD-DFT values: AMs ....................78
General procedure for DTM synthesis ....................83
**2a** (3,4-bis((4-chlorophenyl)thio)-1-methyl-1H-pyrrole-2,5-dione) ....................83
**2b** (3,4-bis(isopropylthio)-1-methyl-1H-pyrrole-2,5-dione) ....................84
**2c** (3,4-bis(ethylthio)-1-methyl-1H-pyrrole-2,5-dione) ....................85
DTM NMR Spectra ....................86
DTM UV-visible spectra ....................89
DTM Fluorescence Spectra ....................91
General procedure for ATM synthesis ....................93
**3a** (1-methyl-3-morpholino-4-(quinolin-2-ylthio)-1H-pyrrole-2,5-dione) ....................93
**3b** (3-((4-chlorophenyl)thio)-1-methyl-4-morpholino-1H-pyrrole-2,5-dione) ....................94
**3c** (3-(ethylthio)-1-methyl-4-morpholino-1H-pyrrole-2,5-dione) ....................95
ATM NMR Spectra ....................96
ATM UV-visible spectra ....................99
ATM Fluorescence Spectra ....................101
Photophysical Summary: ....................103
CIE Diagram- Combined ....................105
HOMO-LUMO Projections ....................106
HOMO-LUMO Projections- AM ....................106
HOMO-LUMO Projections- DTM ....................113
HOMO-LUMO Projections- ATM ....................115
Computational methods: TD-DFT ....................117
Additional ANN training statistics ....................118
ANN training with validation set ....................119
DFT functional/basis-set comparisons ....................120
Appendix- IR Spectra ....................121
Appendix- TD-DFT Geometries (*in vacuo*) ....................124
AMs ....................124
1a (3-chloro-1-methyl-4-(propylamino)-1H-pyrrole-2,5-dione) ....................124
1b (3-chloro-1-ethyl-4-(isopropylamino)-1H-pyrrole-2,5-dione) ....................125

1c (3-chloro-1-methyl-4-(pentan-3-ylamino)-1H-pyrrole-2,5-dione) ....126
1d (3-(tert-butylamino)-4-chloro-1-methyl-1H-pyrrole-2,5-dione) ....127
1e (3-chloro-4-(cyclopropylamino)-1-methyl-1H-pyrrole-2,5-dione) ....128
1f (3-chloro-4-(cyclobutylamino)-1-methyl-1H-pyrrole-2,5-dione) ....129
1g (3-chloro-4-(cyclopentylamino)-1-methyl-1H-pyrrole-2,5-dione) ....130
1h (3-chloro-4-(cyclohexylamino)-1-methyl-1H-pyrrole-2,5-dione) ....131
1i (3-(allylamino)-4-chloro-1-methyl-1H-pyrrole-2,5-dione) ....132
1j (3-chloro-1-methyl-4-(prop-2-yn-1-ylamino)-1H-pyrrole-2,5-dione) ....133
1k (3-(allylamino)-4-chloro-1-(prop-2-yn-1-yl)-1H-pyrrole-2,5-dione) ....134
1l (3-chloro-4-(cyclopropylamino)-1-(prop-2-yn-1-yl)-1H-pyrrole-2,5-dione) ....134
DTMs ....136
2a (3,4-bis((4-chlorophenyl)thio)-1-methyl-1H-pyrrole-2,5-dione) ....136
2b (3,4-bis(isopropylthio)-1-methyl-1H-pyrrole-2,5-dione) ....137
2c (3,4-bis(ethylthio)-1-methyl-1H-pyrrole-2,5-dione) ....138
ATMs ....140
3a (1-methyl-3-morpholino-4-(quinolin-2-ylthio)-1H-pyrrole-2,5-dione) ....140
3b (3-((4-chlorophenyl)thio)-1-methyl-4-morpholino-1H-pyrrole-2,5-dione) ....141
3c (3-(ethylthio)-1-methyl-4-morpholino-1H-pyrrole-2,5-dione) ....143
Appendix- TD-DFT Geometries and absorbance values of conformers (*in vacuo*) ....145
1k conformers ....145
2b conformers ....153
3c conformers ....163
Appendix- Functional/Basis Set Combinations ....176
Aminomaleimides (AMs) ....176
Dithiomaleimides (DTMs) ....177
Aminothiomaleimides (ATMs) ....177

## Tables

Table 1: Training Set Information, including photophysical properties, solvent and SMILES. ....18
Table 2: AM UV-visible characterisation summary (Samples measured at appropriate concentrations in diethyl ether) ....53
Table 3: ANN-derived absorbance values with varying solvent ....76
Table 4: DTM UV-visible characterisation summary (Samples measured at appropriate concentrations in diethyl ether) ....90
Table 5: ATM UV-visible characterisation summary (Samples measured at appropriate concentrations in diethyl ether) ....100
Table 6: UV-visible/ Fluorescence properties summary (n.b. ANN values correspond to diethyl ether, and TD-DFT values were calculated in vacuo) ....105

Table 7: Summary of TD-DFT-determined values, including energies, absorbance wavelengths and their strength. ....117
Table 8: Representative test-set statistics for ANN fitting in the case of absorption and emission. ...118

# Figures

Figure 1: Training Set Solvent Composition ....20
Figure 2: Initial maleimides structures for future functionalisation ....21
Figure 3: Maleimide addition-elimination mechanism....23
Figure 4: General procedure for synthesis of AMs....23
Figure 5: $^{1}$H and $^{13}$C NMR Spectra of DCM ....32
Figure 6: $^{13}$C NMR Spectra of DBM ....33
Figure 7: $^{1}$H and $^{13}$C NMR Spectra of 1a....34
Figure 8: $^{1}$H and $^{13}$C NMR Spectra of 1b ....35
Figure 9: $^{1}$H and $^{13}$C NMR Spectra of 1c....36
Figure 10: $^{1}$H and $^{13}$C NMR Spectra of 1d ....37
Figure 11: $^{1}$H and $^{13}$C NMR Spectra of 1e....38
Figure 12: $^{1}$H and $^{13}$C NMR Spectra of 1f....39
Figure 13: $^{1}$H and $^{13}$C NMR Spectra of 1g ....40
Figure 14: $^{1}$H and $^{13}$C NMR Spectra of 1h ....41
Figure 15: $^{1}$H and $^{13}$C NMR Spectra of 1i ....42
Figure 16: $^{1}$H and $^{13}$C NMR Spectra of 1j ....43
Figure 17: $^{1}$H and $^{13}$C NMR Spectra of 1k ....44
Figure 18: $^{1}$H and $^{13}$C NMR Spectra of 1l ....45
Figure 19: Stacked UV-visible spectra of AMs in diethyl ether....46
Figure 20: UV-visible spectrum of 1a in diethyl ether, $\lambda_{max}$ = 372 nm ....47
Figure 21: UV-visible spectrum of 1b in diethyl ether, $\lambda_{max}$ = 370 nm ....47
Figure 22: UV-visible spectrum of 1c in diethyl ether, $\lambda_{max}$ = 371 nm ....48
Figure 23: UV-visible spectrum of 1d in diethyl ether, $\lambda_{max}$ = 364 nm ....48
Figure 24: UV-visible spectrum of 1e in diethyl ether, $\lambda_{max}$ = 374 nm ....49
Figure 25: UV-visible spectrum of 1f in diethyl ether, $\lambda_{max}$ = 373 nm....49
Figure 26: UV-visible spectrum of 1g in diethyl ether, $\lambda_{max}$ = 372 nm ....50
Figure 27: UV-visible spectrum of 1h in diethyl ether, $\lambda_{max}$ = 371 nm ....50
Figure 28: UV-visible spectrum of 1i in diethyl ether, $\lambda_{max}$ = 370 nm....51
Figure 29: UV-visible spectrum of 1j in diethyl ether, $\lambda_{max}$ = 364 nm....51
Figure 30: UV-visible spectrum of 1k in diethyl ether, $\lambda_{max}$ = 367 nm ....52
Figure 31: UV-visible spectrum of 1k in diethyl ether, $\lambda_{max}$ = 369 nm ....52
Figure 32: Stacked UV-visible spectra of 1a ....54
Figure 33: Stacked UV-visible spectra of 1b....54
Figure 34: Stacked UV-visible spectra of 1c ....54
Figure 35: Stacked UV-visible spectra of 1i....54
Figure 36: Stacked UV-visible spectra of 1j....54
Figure 37: 1a TD-DFT Simulated UV-visible absorbance spectra (in vacuo) ....55
Figure 38: TD-DFT Predicted and experimental UV-visible spectra of 1a in various solvents (adjusted for wavenumber)....57
Figure 39: Fluorescence Spectra of 1a in diethyl ether ....58
Figure 40: Fluorescence Spectra of 1b in diethyl ether ....58

Figure 41:Fluorescence Spectra of 1c in diethyl ether ....................58
Figure 42:Fluorescence Spectra of 1d in diethyl ether ....................58
Figure 43: Fluorescence Spectra of 1e in diethyl ether ....................58
Figure 44: Fluorescence Spectra of 1f in diethyl ether ....................58
Figure 45: Fluorescence Spectra of 1g in diethyl ether ....................59
Figure 46: Fluorescence Spectra of 1h in diethyl ether ....................59
Figure 47: Fluorescence Spectra of 1i in diethyl ether ....................59
Figure 48: Fluorescence Spectra of 1j in diethyl ether ....................59
Figure 49: Fluorescence Spectra of 1k in diethyl ether ....................59
Figure 50: Fluorescence Spectra of 1l in diethyl ether ....................59
Figure 51: Stacked Fluorescence Spectra of 1a in various solvents ....................60
Figure 52: Stacked Fluorescence Spectra of 1b in various solvents ....................60
Figure 53: Stacked Fluorescence Spectra of 1c in various solvents ....................61
*Figure 54: Stacked Fluorescence Spectra of 1k in various solvents* ....................61
*Figure 55: Stacked Fluorescence Spectra of 1j in various solvents* ....................62
Figure 56: Fluorescence contour plot of 1a in diethyl ether ....................63
Figure 57: Fluorescence contour plot of 1b in diethyl ether ....................63
Figure 58: Fluorescence contour plot of 1c in diethyl ether ....................64
Figure 59: Fluorescence contour plot of 1d in diethyl ether ....................64
Figure 60: Fluorescence contour plot of 1e in diethyl ether ....................65
Figure 61: Fluorescence contour plot of 1f in diethyl ether ....................65
Figure 62: Fluorescence contour plot of 1g in diethyl ether ....................66
Figure 63: Fluorescence contour plot of 1h in diethyl ether ....................66
Figure 64: Fluorescence contour plot of 1i in diethyl ether ....................67
Figure 65: Fluorescence contour plot of 1j in diethyl ether ....................67
Figure 66: Fluorescence contour plot of 1k in diethyl ether ....................68
Figure 67: Fluorescence contour plot of 1l in diethyl ether ....................68
Figure 68: Figure 68: ANN wavelength response to varying the dielectric constant for AM derivatives ....................77
Figure 69: ANN wavelength response to varying the dielectric constant for AM derivatives (zoomed to dielectric constant 0-10). ....................77
Figure 70: Experimental, TD-DFT and ANN-predicted wavelengths, excitation (left) and emission (right) for 1a ....................78
Figure 71: Experimental, TD-DFT and ANN-predicted wavelengths, excitation (left) and emission (right) for 1b ....................79
Figure 72: Experimental, TD-DFT and ANN-predicted wavelengths, excitation (left) and emission (right) for 1c ....................79
Figure 73: Experimental, TD-DFT and ANN-predicted wavelengths, excitation (left) and emission (right) for 1i ....................79
Figure 74: Experimental, TD-DFT and ANN-predicted wavelengths, excitation (left) and emission (right) for 1j ....................80
Figure 75: ANN predicted difference as a function of dielectric constant for excitation (left) and emission (right) of 1a ....................80
Figure 76: ANN predicted difference as a function of dielectric constant for excitation (left) and emission (right) of 1b ....................80
Figure 77: ANN predicted difference as a function of dielectric constant for excitation (left) and emission (right) of 1c ....................81

Figure 78: ANN predicted difference as a function of dielectric constant for excitation (left) and emission (right) of 1i....81
Figure 79: ANN predicted difference as a function of dielectric constant for excitation (left) and emission (right) of 1j....81
Figure 80: General procedure for synthesis of DTMs....83
Figure 81: $^{1}$H and $^{13}$C NMR Spectra of 2a....86
Figure 82: $^{1}$H and $^{13}$C NMR Spectra of 2b....87
Figure 83: $^{1}$H and $^{13}$C NMR Spectra of 2c....88
Figure 84: Stacked UV-visible spectra of DTMs a-c....89
Figure 85: Experimental (left) and simulated (right, adjusted for wavenumber) of 2a....89
Figure 86: Experimental (left) and simulated (right, adjusted for wavenumber) of 2b....90
Figure 87: Experimental (left) and simulated (right, adjusted for wavenumber) of 2c....90
Figure 88: Fluorescence emission spectra of 2a in diethyl ether....91
Figure 89: Fluorescence excitation/ emission spectra of 2b in diethyl ether....91
Figure 90: Fluorescence excitation/ emission spectra of 2c in diethyl ether....92
Figure 91: General procedure for synthesis of ATMs....93
Figure 92: $^{1}$H and $^{13}$C NMR Spectra of 3a....96
Figure 93: $^{1}$H and $^{13}$C NMR Spectra of 3b....97
Figure 94: $^{1}$H and $^{13}$C NMR Spectra of 3c....98
Figure 95: Stacked UV-visible spectra of ATMs a-c....99
Figure 96: Experimental (left) and simulated (right, adjusted for wavenumber) of 3a....99
Figure 97: Experimental (left) and simulated (right, adjusted for wavenumber) of 3b....100
Figure 98: Experimental (left) and simulated (right, adjusted for wavenumber) of 3c....100
Figure 99: Fluorescence emission spectra of 3a in diethyl ether....101
Figure 100: Fluorescence emission spectra of 3b in diethyl ether....101
Figure 101: Fluorescence emission spectra of 3c in diethyl ether....102
Figure 102: CIE 1931 Chromaticity Diagram of all synthesised maleimide derivatives in this work....105
Figure 103: HOMO-1, HOMO, LUMO and LUMO +1 projection of 1a, plus corresponding energies (eV)....106
Figure 104: HOMO-1, HOMO, LUMO and LUMO +1 projection of 1b, plus corresponding energies (eV)....107
Figure 105: HOMO-1, HOMO, LUMO and LUMO +1 projection of 1c, plus corresponding energies (eV)....107
Figure 106: HOMO-1, HOMO, LUMO and LUMO +1 projection of 1d, plus corresponding energies (eV)....108
Figure 107: HOMO-1, HOMO, LUMO and LUMO +1 projection of 1e, plus corresponding energies (eV)....108
Figure 108: HOMO-1, HOMO, LUMO and LUMO +1 projection of 1f, plus corresponding energies (eV)....109
Figure 109: HOMO-1, HOMO, LUMO and LUMO +1 projection of 1g, plus corresponding energies (eV)....109
Figure 110: HOMO-1, HOMO, LUMO and LUMO +1 projection of 1h, plus corresponding energies (eV)....110
Figure 111: HOMO-1, HOMO, LUMO and LUMO +1 projection of 1i, plus corresponding energies (eV)....110
Figure 112: HOMO-1, HOMO, LUMO and LUMO +1 projection of 1j, plus corresponding energies (eV)....111

Figure 113: HOMO-1, HOMO, LUMO and LUMO +1 projection of 1k, plus corresponding energies (eV). ....111
Figure 114: HOMO-1, HOMO, LUMO and LUMO +1 projection of 1l, plus corresponding energies (eV). ....112
Figure 115: HOMO-1, HOMO, LUMO and LUMO +1 projection of 2a, plus corresponding energies (eV). ....113
Figure 116: HOMO-1, HOMO, LUMO and LUMO +1 projection of 2b, plus corresponding energies (eV). ....113
Figure 117: HOMO-1, HOMO, LUMO and LUMO +1 projection of 2c, plus corresponding energies (eV). ....114
Figure 118: HOMO-1, HOMO, LUMO and LUMO +1 projection of 3a, plus corresponding energies (eV). ....115
Figure 119: HOMO-1, HOMO, LUMO and LUMO +1 projection of 3b, plus corresponding energies (eV). ....115
Figure 120: HOMO-1, HOMO, LUMO and LUMO +1 projection of 3c, plus corresponding energies (eV). ....116
Figure 121: Training loss curve (upper) and R2 value in validation set (lower panel) for ANN training in which 10% of the dataset is used as a validation set. ....119
Figure 122: Comparison of three different combinations of exchange-correlation functional and basis sets in TD-DFT calculations for novel maleimides synthesised in this article (see Figs. 7-10 in main text). ....120
Figure 123: FTIR Spectrum of 1a ....121
Figure 124: FTIR Spectrum of 1b ....121
Figure 125: FTIR Spectrum of 1c ....121
Figure 126: FTIR Spectrum of 1d ....121
Figure 127: FTIR Spectrum of 1e ....121
Figure 128: FTIR Spectrum of 1f ....121
Figure 129: FTIR Spectrum of 1g ....122
Figure 130: FTIR Spectrum of 1h ....122
Figure 131: FTIR Spectrum of 1i ....122
Figure 132: FTIR Spectrum of 1j ....122
Figure 133: FTIR Spectrum of 1k ....122
Figure 134: FTIR Spectrum of 1l ....122
Figure 135: FTIR Spectrum of 2a ....123
Figure 136: FTIR Spectrum of 2b ....123
Figure 137: FTIR Spectrum of 2c ....123
Figure 138: FTIR Spectrum of 3a ....123
Figure 139: FTIR Spectrum of 3b ....123
Figure 140: FTIR Spectrum of 3c ....123

# Training Set & Artificial Neural Network Construction

## Training Set

| SMILES | Solvent | Quantum Yield (%) | Extinction Coefficient ($M^{-1}cm^{-1}$) | Abs/Ex (nm) | Em (nm) |
|---|---|---|---|---|---|
| **O=C1N(C(C(NCC)=C1Cl)=O)C** | Diethyl Ether | 37 | 3669 | 369 | 475 |
| **O=C1N(C(C(NCC)=C1Cl)=O)C** | Cyclohexane | 56 | 3198 | 365 | 482 |
| **O=C1N(C(C(NCC)=C1Cl)=O)C** | Methanol | 1 | 3355 | 379 | 550 |
| **O=C1N(C(C(NCC)=C1Cl)=O)C** | Water | <0.1 | 2442 | 398 | 580 |
| **O=C1N(C(C(NCC)=C1Br)=O)C** | Diethyl Ether | 30 | 3733 | 370 | 474 |
| **O=C1N(C(C(NCC)=C1Br)=O)C** | Cyclohexane | 37 | 3496 | 366 | 472 |
| **O=C1N(C(C(NCC)=C1Br)=O)C** | Methanol | 1 | 4811 | 384 | 548 |
| **O=C1N(C(C(NCC)=C1Br)=O)C** | Water | <0.1 | 3645 | 400 | 583 |
| **O=C1N(C(C(NCC)=C1I)=O)C** | Diethyl Ether | 8 | 4419 | 370 | 487 |
| **O=C1N(C(C(NCC)=C1I)=O)C** | Cyclohexane | 11 | 3483 | 372 | 472 |
| **O=C1N(C(C(NCC)=C1I)=O)C** | Methanol | 1 | 3405 | 388 | 560 |
| **O=C1N(C(C(NCC)=C1I)=O)C** | Water | <0.1 | 2925 | 404 | 580 |
| **O=C1NC(C(NCCCC)=C1Cl)=O** | Diethyl Ether | 42 | 5073 | 359 | 461 |
| **O=C1NC(C(NCCCC)=C1Cl)=O** | Cyclohexane | 65 | 4220 | 356 | 442 |
| **O=C1NC(C(NCCCC)=C1Cl)=O** | Methanol | 1 | 4313 | 374 | 512 |
| **O=C1NC(C(NCCCC)=C1Cl)=O** | Water | <0.1 | 2867 | 387 | 550 |
| **O=C1NC(C(NCCCC)=C1Br)=O** | Diethyl Ether | 31 | 5531 | 358 | 461 |
| **O=C1NC(C(NCCCC)=C1Br)=O** | Cyclohexane | 47 | 4489 | 355 | 441 |
| **O=C1NC(C(NCCCC)=C1Br)=O** | Methanol | 2 | 4451 | 374 | 513 |
| **O=C1NC(C(NCCCC)=C1Br)=O** | Water | <0.1 | 2768 | 387 | 547 |
| **O=C1N(C(C(OCC)=C1Cl)=O)C** | Diethyl Ether | 12 | 770 | 335 | 458 |

| | | | | | |
|---|---|---|---|---|---|
| **O=C1N(C(C(OCC)=C1Cl)=O)C** | Cyclohexane | 24 | 875 | 330 | 461 |
| **O=C1N(C(C(OCC)=C1Cl)=O)C** | Methanol | 2 | 825 | 333 | 486 |
| **O=C1N(C(C(OCC)=C1Cl)=O)C** | Water | <0.1 | 548 | 342 | 505 |
| **O=C1N(C(C(OCC)=C1Br)=O)C** | Diethyl Ether | 10 | 666 | 336 | 458 |
| **O=C1N(C(C(OCC)=C1Br)=O)C** | Cyclohexane | 24 | 668 | 333 | 461 |
| **O=C1N(C(C(OCC)=C1Br)=O)C** | Methanol | 2 | 629 | 335 | 486 |
| **O=C1N(C(C(OCC)=C1Br)=O)C** | Water | <0.1 | 432 | 343 | 501 |
| **O=C1N(C(C(OCC2=CC=CC=C2)=C1Cl)=O)C** | Diethyl Ether | 12 | 1267 | 335 | 464 |
| **O=C1N(C(C(OCC2=CC=CC=C2)=C1Br)=O)C** | Diethyl Ether | 9 | 907 | 340 | 465 |
| **O=C1N(C(C(NCC)=C1SCCCC)=O)C** | Diethyl Ether | 4 | 1917 | 379 | 558 |
| **O=C1N(C(C(NCC)=C1SCCCCCCCCCCCCCCCCC)=O)C** | Diethyl Ether | 6 | 2790 | 383 | 561 |
| **O=C1N(C(C(NCC)=C1SC2=CC=CC=C2)=O)C** | Diethyl Ether | 12 | 1761 | 370 | 545 |
| **O=C1N(C(C(NCC)=C1SC2=CC=CC=C2C)=O)C** | Diethyl Ether | 12 | 2595 | 370 | 547 |
| **O=C1N(C(C(NCCCC)=C1SC2=CC=CC=C2)=O)C** | Diethyl Ether | 9 | 3898 | 368 | 544 |
| **O=C1NC(C(NCCCC)=C1SC2=CC=CC=C2)=O.C** | Diethyl Ether | 23 | 2685 | 355 | 526 |
| **O=C1NC(C(NCCCC)=C1SC2=CC=CC=C2)=O.C** | Cyclohexane | 37 | 4743 | 355 | 513 |
| **O=C1NC(C(NCCCC)=C1SC2=CC=CC=C2)=O.C** | Methanol | <0.1 | 4269 | 370 | 593 |
| **O=C1NC(C(NCC)=C1SCCCC)=O** | Diethyl Ether | 13 | 4363 | 378 | 535 |
| **O=C1NC(C(NCCCC)=C1SCC2=CC=CC=C2)=O** | Diethyl Ether | 17 | 2276 | 370 | 532 |
| **O=C1NC(C(NCCCC)=C1)=O** | Diethyl Ether | 57 | -- | 341 | 436 |
| **O=C1NC(C(Br)=C1NC2CCCCC2)=O** | Diethyl Ether | 28 | -- | 361 | 460 |
| **O=C1NC(C(Br)=C1NC(C)C)=O** | Diethyl Ether | 27 | -- | 359 | 460 |
| **O=C(N1)C(NC2(C[C@@H]3C4)CC(C3)CC4C2)=C(Br)C1=O** | Diethyl Ether | 2 | -- | 357 | 473 |
| **CCCCCCCC/C=C\CCCCCCCCNC1=C(Br)C(NC1=O)=O** | Diethyl Ether | 32 | -- | 361 | 460 |
| **O=C1NC(C(Br)=C1N2CCCCC2)=O** | Diethyl Ether | 10 | -- | 383 | 466 |
| **O=C1NC(C(SCCCC)=C1SCCCC)=O** | Cyclohexane | 28 | 5800 | 407 | 486 |
| **O=C1NC(C(SCCCC)=C1SCCCC)=O** | Dioxane | 10 | 4900 | 402 | 504 |
| **O=C1NC(C(SCCCC)=C1SCCCC)=O** | Methanol | 0.43 | 4700 | 402 | 546 |
| **O=C1NC(C(Br)=C1NCCCC)=O** | Cyclohexane | 31 | 3700 | 357 | 442 |

| | | | | | |
|---|---|---|---|---|---|
| **O=C1NC(C(Br)=C1NCCCC)=O** | Dioxane | 38 | 4500 | 367 | 469 |
| **O=C1NC(C(Br)=C1NCCCC)=O** | Methanol | 1.1 | 5500 | 373 | 514 |
| **O=C1NC(C([H])=C1NCCCC)=O** | Cyclohexane | 52 | 5000 | 333 | 412 |
| **O=C1NC(C([H])=C1NCCCC)=O** | Dioxane | 59 | 4900 | 346 | 450 |
| **O=C1NC(C([H])=C1NCCCC)=O** | Methanol | 2.8 | 5500 | 357 | 490 |
| **O=C1NC(C([H])=C1NCCCC)=O** | Water | 0.31 | 5400 | 369 | 520 |
| **O=C1NC(C(Br)=C1N(CC)CC)=O** | Cyclohexane | 16 | 7300 | 380 | 442 |
| **O=C1NC(C(Br)=C1N(CC)CC)=O** | Dioxane | 15 | 6600 | 386 | 474 |
| **O=C1NC(C(Br)=C1N(CC)CC)=O** | Methanol | 0.54 | 6500 | 292 | 515 |
| **O=C1NC(C(Br)=C1N(CC)CC)=O** | Water | 0.37 | 5700 | 410 | 567 |
| **O=C1NC(C([H])=C1N(CC)CC)=O** | Dioxane | 0.43 | 6600 | 367 | 460 |
| **O=C1NC(C([H])=C1N(CC)CC)=O** | Methanol | 0.2 | 6600 | 374 | 500 |
| **O=C1NC(C([H])=C1N(CC)CC)=O** | Water | 0.11 | 6400 | 391 | 535 |
| **O=C1NC(C(Br)=C1NC(C)C)=O** | Dioxane | 35 | 5000 | 364 | 468 |
| **O=C1NC(C(Br)=C1NCC2=CC=CC=C2)=O** | Dioxane | 34 | 3300 | 362 | 466 |
| **O=C1N(C)C(C(Br)=C1NCCCC)=O** | Dioxane | 20 | 3700 | 376 | 486 |
| **O=C1N(C2=CC=CC=C2)C(C(Br)=C1NCCCC)=O** | Dioxane | 0.94 | 3700 | 378 | 493 |
| **O=C1N(CC(F)(F)F)C(C(Cl)=C1NC(C)C)=O** | Diethyl ether | 48 | 6499 | 368 | 467 |
| **O=C1N(CC(F)(F)F)C(C(Cl)=C1NC(C)C)=O** | Toluene | 45 | 7432 | 367 | 470 |
| **O=C1N(CC(F)(F)F)C(C(Cl)=C1NC(C)C)=O** | Tetrahydrofuran | 41 | 5681 | 369 | 476 |
| **O=C1N(CC(F)(F)F)C(C(Cl)=C1NC(C)C)=O** | Dioxane | 51 | 6428 | 371 | 475 |
| **O=C1N(CC(F)(F)F)C(C(Cl)=C1NC(C)C)=O** | DMSO | 12 | 6014 | 378 | 499 |
| **O=C1N(CC(F)(F)F)C(C(Cl)=C1NC(C)C)=O** | Methanol | 2 | 4259 | 375 | 508 |
| **O=C1N(CC(F)(F)F)C(C(Cl)=C1NCC)=O** | Diethyl ether | 49 | 6647 | 368 | 468 |
| **O=C1N(CC(F)(F)F)C(C(Cl)=C1NCC)=O** | Toluene | 48 | 7252 | 369 | 471 |
| **O=C1N(CC(F)(F)F)C(C(Cl)=C1NCC)=O** | Tetrahydrofuran | 53 | 5983 | 341 | 476 |
| **O=C1N(CC(F)(F)F)C(C(Cl)=C1NCC)=O** | Dioxane | 49 | 6040 | 371 | 475 |
| **O=C1N(CC(F)(F)F)C(C(Cl)=C1NCC)=O** | DMSO | 13 | 6752 | 381 | 494 |
| **O=C1N(CC(F)(F)F)C(C(Cl)=C1NCC)=O** | Methanol | 2 | 4132 | 377 | 514 |

| | | | | | |
|---|---|---|---|---|---|
| **O=C1N(CC)C(C(Cl)=C1NCC(F)(F)F)=O** | Diethyl ether | 36 | 4634 | 359 | 469 |
| **O=C1N(CC)C(C(Cl)=C1NCC(F)(F)F)=O** | Toluene | 27 | 5358 | 360 | 471 |
| **O=C1N(CC)C(C(Cl)=C1NCC(F)(F)F)=O** | Tetrahydrofuran | 34 | 4495 | 364 | 475 |
| **O=C1N(CC)C(C(Cl)=C1NCC(F)(F)F)=O** | Dioxane | 39 | 3070 | 363 | 475 |
| **O=C1N(CC)C(C(Cl)=C1NCC(F)(F)F)=O** | DMSO | 29 | 3482 | 376 | 494 |
| **O=C1N(CC)C(C(Cl)=C1NCC(F)(F)F)=O** | Methanol | 4 | 4030 | 369 | 513 |
| **O=C1N(CC(F)(F)F)C(C=C1NC(C)C)=O** | Diethyl ether | 48 | 6509 | 349 | 457 |
| **O=C1N(CC(F)(F)F)C(C=C1NC(C)C)=O** | Toluene | 43 | 6524 | 350 | 459 |
| **O=C1N(CC(F)(F)F)C(C=C1NC(C)C)=O** | Tetrahydrofuran | 44 | 5782 | 352 | 461 |
| **O=C1N(CC(F)(F)F)C(C=C1NC(C)C)=O** | Dioxane | 26 | 6354 | 351 | 460 |
| **O=C1N(CC(F)(F)F)C(C=C1NC(C)C)=O** | DMSO | 15 | 6180 | 363 | 477 |
| **O=C1N(CC(F)(F)F)C(C=C1NC(C)C)=O** | Methanol | 4 | 5260 | 365 | 490 |
| **O=C1N(CC(F)(F)F)C(C=C1NCC)=O** | Diethyl ether | 48 | 7964 | 351 | 451 |
| **O=C1N(CC(F)(F)F)C(C=C1NCC)=O** | Toluene | 47 | 7619 | 351 | 457 |
| **O=C1N(CC(F)(F)F)C(C=C1NCC)=O** | Tetrahydrofuran | 53 | 6637 | 372 | 457 |
| **O=C1N(CC(F)(F)F)C(C=C1NCC)=O** | Dioxane | 50 | 7438 | 352 | 461 |
| **O=C1N(CC(F)(F)F)C(C=C1NCC)=O** | DMSO | 22 | 6576 | 363 | 474 |
| **O=C1N(CC(F)(F)F)C(C=C1NCC)=O** | Methanol | 5 | 5100 | 357 | 489 |
| **O=C1N(CC(F)(F)F)C(C(Cl)=C1NCC(F)(F)F)=O** | Diethyl ether | 41 | 7798 | 358 | 461 |
| **O=C1N(CC(F)(F)F)C(C(Cl)=C1NCC(F)(F)F)=O** | Toluene | 39 | 7569 | 352 | 460 |
| **O=C1N(CC(F)(F)F)C(C(Cl)=C1NCC(F)(F)F)=O** | Tetrahydrofuran | 52 | 6700 | 357 | 467 |
| **O=C1N(CC(F)(F)F)C(C(Cl)=C1NCC(F)(F)F)=O** | Dioxane | 55 | 6649 | 356 | 467 |
| **O=C1N(CC(F)(F)F)C(C(Cl)=C1NCC(F)(F)F)=O** | DMSO | 40 | 6019 | 367 | 490 |
| **O=C1N(CC(F)(F)F)C(C(Cl)=C1NCC(F)(F)F)=O** | Methanol | 6 | 5783 | 361 | 503 |
| **O=C1N(CC)C(C(Cl)=C1NCC)=O** | Diethyl ether | 36 | 3621 | 373 | 460 |
| **O=C1N(CC)C(C(Cl)=C1NCC)=O** | Toluene | 22 | 3923 | 371 | 462 |
| **O=C1N(CC)C(C(Cl)=C1NCC)=O** | Tetrahydrofuran | 21 | 4256 | 374 | 476 |
| **O=C1N(CC)C(C(Cl)=C1NCC)=O** | Dioxane | 26 | 3551 | 375 | 475 |
| **O=C1N(CC)C(C(Cl)=C1NCC)=O** | DMSO | 10 | 3483 | 383 | 482 |

| | | | | | |
|---|---|---|---|---|---|
| **O=C1N(CC)C(C(Cl)=C1NCC)=O** | Methanol | <1 | 2109 | 383 | 499 |
| **O=C1N(CC)C(C=C1NCC)=O** | Diethyl ether | 34 | 3547 | 359 | 477 |
| **O=C1N(CC)C(C=C1NCC)=O** | Toluene | 28 | 3620 | 360 | 478 |
| **O=C1N(CC)C(C=C1NCC)=O** | Tetrahydrofuran | 25 | 4632 | 364 | 484 |
| **O=C1N(CC)C(C=C1NCC)=O** | Dioxane | 23 | 2917 | 363 | 488 |
| **O=C1N(CC)C(C=C1NCC)=O** | DMSO | 9 | 2571 | 376 | 505 |
| **O=C1N(CC)C(C=C1NCC)=O** | Methanol | <1 | 2877 | 369 | 531 |
| **O=C(C=C1NCCCC)N(CCCC)C1=O** | Cyclohexane | 70 | 4165 | 345 | 445 |
| **O=C(C=C1NCCCC)N(CCCC)C1=O** | THF | 62 | 3915 | 357 | 461 |
| **O=C(C=C1NCCCC)N(CCCC)C1=O** | Acetonitrile | 44 | 4040 | 365 | 470 |
| **O=C(C=C1NCCCC)N(CCCC)C1=O** | EtOH | 3 | 5955 | 368 | 498 |
| **O=C(C=C1NCCCC)N(CCCC)C1=O** | Water | 0.1 | 3220 | 384 | 530 |
| **CCCCCCCC/C=C\CCCCCCCCNC1=C(Br)C(N(CCCCCCCC/C=C\CCCCCCCC)C1=O)=O** | Cyclohexane | 72 | 5655 | 346 | 446 |
| **CCCCCCCC/C=C\CCCCCCCCNC1=C(Br)C(N(CCCCCCCC/C=C\CCCCCCCC)C1=O)=O** | THF | 53 | 7100 | 357 | 464 |
| **CCCCCCCC/C=C\CCCCCCCCNC1=C(Br)C(N(CCCCCCCC/C=C\CCCCCCCC)C1=O)=O** | Acetonitrile | 36 | 7040 | 359 | 472 |
| **CCCCCCCC/C=C\CCCCCCCCNC1=C(Br)C(N(CCCCCCCC/C=C\CCCCCCCC)C1=O)=O** | EtOH | 2 | 9675 | 365 | 499 |
| **O=C(C=C1NC)N(C)C1=O** | Cyclohexane | 65 | 5305 | 343 | 448 |
| **O=C(C=C1NC)N(C)C1=O** | THF | 45 | 7850 | 356 | 462 |
| **O=C(C=C1NC)N(C)C1=O** | Acetonitrile | 26 | 7660 | 357 | 473 |
| **O=C(C=C1NC)N(C)C1=O** | EtOH | 2 | 7805 | 361 | 496 |
| **O=C(C=C1NC)N(C)C1=O** | Water | 0.1 | 7490 | 374 | 529 |
| **O=C(C=C1NC2CCCCC2)N(CCCC)C1=O** | Cyclohexane | 71 | 3860 | 345 | 445 |
| **O=C(C=C1NC2CCCCC2)N(CCCC)C1=O** | THF | 48 | 6100 | 358 | 463 |
| **O=C(C=C1NC2CCCCC2)N(CCCC)C1=O** | Acetonitrile | 34 | 5545 | 363 | 472 |
| **O=C(C=C1NC2CCCCC2)N(CCCC)C1=O** | EtOH | 2 | 6105 | 367 | 500 |
| **O=C(C=C1NC2CCCCC2)N(CCCC)C1=O** | Water | 0.03 | 2575 | 379 | 535 |

| | | | | | |
|---|---|---|---|---|---|
| **O=C(C=C1NCCCC)N(CC2=CC=CC=C2)C1=O** | Cyclohexane | 71 | 3150 | 344 | 448 |
| **O=C(C=C1NCCCC)N(CC2=CC=CC=C2)C1=O** | THF | 69 | 3850 | 357 | 463 |
| **O=C(C=C1NCCCC)N(CC2=CC=CC=C2)C1=O** | Acetonitrile | 40 | 4775 | 358 | 470 |
| **O=C(C=C1NCCCC)N(CC2=CC=CC=C2)C1=O** | EtOH | 5.4 | 4895 | 361 | 495 |
| **O=C(C=C1NCCCC)N(CC2=CC=CC=C2)C1=O** | Water | 0.3 | 2460 | 381 | 526 |
| **O=C(C=C1NCC2=CC=CC=C2)N(CCCC)C1=O** | Cyclohexane | 70 | 3040 | 344 | 446 |
| **O=C(C=C1NCC2=CC=CC=C2)N(CCCC)C1=O** | THF | 63 | 3640 | 357 | 460 |
| **O=C(C=C1NCC2=CC=CC=C2)N(CCCC)C1=O** | Acetonitrile | 43 | 3285 | 359 | 473 |
| **O=C(C=C1NCC2=CC=CC=C2)N(CCCC)C1=O** | EtOH | 5 | 4435 | 364 | 496 |
| **O=C(C=C1NCC2=CC=CC=C2)N(CCCC)C1=O** | Water | 0.3 | 1680 | 380 | 527 |
| **O=C(C=C1NCC2=CC=CC=C2)N(C)C1=O** | Cyclohexane | 66 | 3040 | 344 | 446 |
| **O=C(C=C1NCC2=CC=CC=C2)N(C)C1=O** | THF | 45 | 2910 | 357 | 461 |
| **O=C(C=C1NCC2=CC=CC=C2)N(C)C1=O** | Acetonitrile | 43 | 3200 | 360 | 470 |
| **O=C(C=C1NCC2=CC=CC=C2)N(C)C1=O** | EtOH | 0.1 | 4525 | 367 | 497 |
| **O=C(C=C1NCC2=CC=CC=C2)N(C)C1=O** | Water | 0 | 4785 | 377 | 527 |
| **O=C(C=C1N2CCCCC2)N(CC3=CC=CC=C3)C1=O** | Cyclohexane | 88 | 2315 | 370 | 464 |
| **O=C(C=C1N2CCCCC2)N(CC3=CC=CC=C3)C1=O** | THF | 7 | 2465 | 380 | 476 |
| **O=C(C=C1N2CCCCC2)N(CC3=CC=CC=C3)C1=O** | Acetonitrile | 1 | 6705 | 382 | 490 |
| **O=C(C=C1N2CCCCC2)N(CC3=CC=CC=C3)C1=O** | EtOH | 1 | 5620 | 388 | 516 |
| **O=C(C=C1N2CCCCC2)N(CC3=CC=CC=C3)C1=O** | Water | 0.3 | 3680 | 405 | 547 |
| **O=C(C=C1NC2=CC=CC=C2)N(C3=CC=CC=C3)C1=O** | Cyclohexane | 0.3 | 3505 | 370 | 447 |
| **O=C(C=C1NC2=CC=CC=C2)N(C3=CC=CC=C3)C1=O** | THF | 0.9 | 3065 | 379 | 435 |
| **O=C(C=C1NC2=CC=CC=C2)N(CCCC)C1=O** | Cyclohexane | 8.6 | 7235 | 366 | 450 |
| **O=C(C=C1NC2=CC=CC=C2)N(CCCC)C1=O** | THF | 0 | 6670 | 375 | 454 |
| **O=C(C=C1NCCCC)N(C2=CC=CC=C2)C1=O** | Cyclohexane | 2 | 2035 | 352 | 452 |
| **O=C(C=C1NCCCC)N(C2=CC=CC=C2)C1=O** | THF | 0.5 | 2505 | 363 | 473 |
| **O=C(C=C1NCCCC)N(C2=CC=CC=C2)C1=O** | Acetonitrile | 0.3 | 3400 | 367 | 460 |
| **O=C(C=C1N2CCCCC2)N(C3=CC=CC=C3)C1=O** | Cyclohexane | 0.7 | 4335 | 371 | 486 |
| **O=C(C=C1N2CCCCC2)N(C3=CC=CC=C3)C1=O** | THF | 1 | 4365 | 378 | 491 |

| | | | | | |
|---|---|---|---|---|---|
| **O=C(C=C1N2CCCCC2)N(C3=CC=CC=C3)C1=O** | Acetonitrile | 0.9 | 5055 | 382 | 495 |
| **O=C(C=C1N2CCCCC2)N(C3=CC=CC=C3)C1=O** | EtOH | 0.6 | 4425 | 390 | 520 |
| **O=C(C(C1=CC=CC=C1)=C2C3=CC=CC=C3)NC2=O** | Hexane | - | - | 350 | 471 |
| **O=C(C(C1=CC=CC=C1)=C2C3=CC=CC=C3)NC2=O** | DCM | 21 | 6030 | 357 | 493 |
| **O=C(C(C1=CC=CC=C1)=C2C3=CC=CC=C3)N(C)C2=O** | Hexane | - | - | 361 | 490 |
| **O=C(C(C1=CC=CC=C1)=C2C3=CC=CC=C3)N(C)C2=O** | DCM | 31 | 4010 | 363 | 503 |
| **O=C(C(C1=CC(C=CC=C2)=C2C=C1)=C3C4=CC=C(C=CC=C5)C5=C4)NC3=O** | Hexane | - | - | 373 | 497 |
| **O=C(C(C1=CC(C=CC=C2)=C2C=C1)=C3C4=CC=C(C=CC=C5)C5=C4)NC3=O** | DCM | 2 | 4690 | 384 | 544 |
| **O=C(C(C1=CC(C=CC=C2)=C2C=C1)=C3C4=CC=C(C=CC=C5)C5=C4)N(C)C3=O** | Hexane | - | - | 377 | 507 |
| **O=C(C(C1=CC(C=CC=C2)=C2C=C1)=C3C4=CC=C(C=CC=C5)C5=C4)N(C)C3=O** | DCM | 3 | 4410 | 383 | 547 |
| **O=C(C(C1=C(C=CC=C2)C2=CC=C1)=C3C4=CC=CC5=C4C=CC=C5)NC3=O** | Hexane | - | - | 391 | 488 |
| **O=C(C(C1=C(C=CC=C2)C2=CC=C1)=C3C4=CC=CC5=C4C=CC=C5)NC3=O** | DCM | 12 | 10510 | 392 | 523 |
| **O=C(C(C1=C(C=CC=C2)C2=CC=C1)=C3C4=CC=CC5=C4C=CC=C5)N(C)C3=O** | Hexane | - | - | 395 | 499 |
| **O=C(C(C1=C(C=CC=C2)C2=CC=C1)=C3C4=CC=CC5=C4C=CC=C5)N(C)C3=O** | DCM | 22 | 9250 | 398 | 526 |
| **O=C(C(C1=CC(C(F)(F)F)=CC=C1)=C2C3=CC=CC(C(F)(F)F)=C3)NC2=O** | Hexane | - | - | 339 | 460 |
| **O=C(C(C1=CC(C(F)(F)F)=CC=C1)=C2C3=CC=CC(C(F)(F)F)=C3)NC2=O** | DCM | 30d | 5710 | 344 | 471 |
| **O=C(C(C1=CC(C(F)(F)F)=CC=C1)=C2C3=CC=CC(C(F)(F)F)=C3)N(C)C2=O** | Hexane | - | - | 352 | 477 |
| **O=C(C(C1=CC(C(F)(F)F)=CC=C1)=C2C3=CC=CC(C(F)(F)F)=C3)N(C)C2=O** | DCM | 53d | 3630 | 359 | 490 |
| **O=C(C(C1=CC=C(C(F)(F)F)C=C1)=C2C3=CC=C(C(F)(F)F)C=C3)NC2=O** | Hexane | - | - | 339 | 459 |
| **O=C(C(C1=CC=C(C(F)(F)F)C=C1)=C2C3=CC=C(C(F)(F)F)C=C3)NC2=O** | DCM | 8d | 2180 | 340 | 470 |
| **O=C(C(C1=CC=C(C(F)(F)F)C=C1)=C2C3=CC=C(C(F)(F)F)C=C3)N(C)C2=O** | Hexane | - | - | 350 | 475 |
| **O=C(C(C1=CC=C(C(F)(F)F)C=C1)=C2C3=CC=C(C(F)(F)F)C=C3)N(C)C2=O** | DCM | 26d | 3220 | 353 | 487 |
| **O=C(C(C1=CC=C(OC)C=C1)=C2C3=CC=C(OC)C=C3)NC2=O** | Hexane | - | - | 387 | 506 |
| **O=C(C(C1=CC=C(OC)C=C1)=C2C3=CC=C(OC)C=C3)NC2=O** | DCM | 25c | 7560 | 404 | 548 |
| **O=C(C(C1=CC=C(OC)C=C1)=C2C3=CC=C(OC)C=C3)N(C)C2=O** | Hexane | - | - | 399 | 518 |
| **O=C(C(C1=CC=C(OC)C=C1)=C2C3=CC=C(OC)C=C3)N(C)C2=O** | DCM | 26c | 7140 | 408 | 551 |

| | | | | | |
|---|---|---|---|---|---|
| **O=C(C(C1=CC=NC=C1)=C2C3=CC=NC=C3)NC2=O** | Hexane | - | - | 340 | 464 |
| **O=C(C(C1=CC=NC=C1)=C2C3=CC=NC=C3)NC2=O** | DCM | 6d | 5640 | 346 | 482 |
| **O=C(C(C1=CSC=C1)=C2C3=CSC=C3)NC2=O** | Hexane | - | - | 414 | 514 |
| **O=C(C(C1=CSC=C1)=C2C3=CSC=C3)NC2=O** | DCM | 7c | 7650 | 421 | 545 |
| **O=C(C(C1=CSC=C1)=C2C3=CSC=C3)N(C)C2=O** | Hexane | - | - | 423 | 530 |
| **O=C(C(C1=CSC=C1)=C2C3=CSC=C3)N(C)C2=O** | DCM | 5c | 6400 | 428 | 552 |
| **O=C(C(C1=CC=C(N(C2=CC=CC=C2)C3=CC=CC4=C3C=CC=C4)C=C1)=C5C6=CC=C(N(C7=CC=C(C=CC=C8)C8=C7)C9=CC=CC=C9)C=C6)N(C)C5=O** | Hexane | - | - | 474 | 572 |
| **O=C(C(C1=CC=C(N(C2=CC=CC=C2)C3=CC=CC4=C3C=CC=C4)C=C1)=C5C6=CC=C(N(C7=CC=C(C=CC=C8)C8=C7)C9=CC=CC=C9)C=C6)N(C)C5=O** | DCM | 13e | 13900 | 485 | 642 |
| **O=C(C(C1=CC=C(N(C2=CC=CC=C2)C3=CC=C(C)C=C3)C=C1)=C4C5=CC=C(N(C6=CC=CC=C6)C7=CC=C(C)C=C7)C=C5)N(C)C4=O** | Hexane | - | - | 476 | 575 |
| **O=C(C(C1=CC=C(N(C2=CC=CC=C2)C3=CC=C(C)C=C3)C=C1)=C4C5=CC=C(N(C6=CC=CC=C6)C7=CC=C(C)C=C7)C=C5)N(C)C4=O** | DCM | 11e | 14830 | 485 | 650 |
| **O=C(C(C1=CC=C(N(C2=CC=CC=C2)C3=CC=CC=C3)C=C1)=C4C5=CC=C(N(C6=CC=CC=C6)C7=CC=CC=C7)C=C5)N(C)C4=O** | Hexane | - | - | 480 | 581 |
| **O=C(C(C1=CC=C(N(C2=CC=CC=C2)C3=CC=CC=C3)C=C1)=C4C5=CC=C(N(C6=CC=CC=C6)C7=CC=CC=C7)C=C5)N(C)C4=O** | DCM | 10e | 13690 | 485 | 653 |
| **O=C(C(C1=CC=C(N(C)C)C=C1)=C2C3=CC=C(N(C)C)C=C3)N(C)C2=O** | Hexane | - | - | 481 | 581 |
| **O=C(C(C1=CC=C(N(C)C)C=C1)=C2C3=CC=C(N(C)C)C=C3)N(C)C2=O** | DCM | 5e | 11050 | 512 | 659 |
| **O=C(N1C2=CC=C(N3C(C(NN4CCOCC4)=C(NN5CCOCC5)C3=O)=O)C=C2)C(NN6CCOCC6)=C(NN7CCOCC7)C1=O** | DCM | - | 9600 | 418 | 624 |
| **O=C(N1C2=CC(N3C(C(NN4CCOCC4)=C(NN5CCOCC5)C3=O)=O)=CC=C2)C(NN6CCOCC6)=C(NN7CCOCC7)C1=O** | DCM | - | 8700 | 417 | 611 |
| **O=C(N1C2=CC(N3C(C(NN4CCOCC4)=C(NC5=CC=C(C)C=C5)C3=O)=O)=CC=C2)C(NN6CCOCC6)=C(NC7=CC=C(C)C=C7)C1=O** | DCM | - | 9000 | 454 | 612 |
| **O=C(N1C2=CC=C(N3C(C(NN4CCOCC4)=C(NC5=CC=C(C)C=C5)C3=O)=O)C=C2)C(NN6CCOCC6)=C(NC7=CC=C(C)C=C7)C1=O** | DCM | - | 8700 | 417 | 611 |
| **O=C(N1C2=CC=C(C)C=C2)C(NN3CCOCC3)=C(NC4=CC=C(C)C=C4)C1=O** | THF | 1 | - | 460 | 609 |
| **O=C(N1C2CCCCC2)C(NN3CCOCC3)=C(NC4=CC=C(C)C=C4)C1=O** | THF | 6 | - | 440 | 599 |
| **O=C(N1C2=CC=C(C)C=C2)C(NN3CCOCC3)=C(NN4CCOCC4)C1=O** | THF | 11 | - | 386 | 609 |
| **O=C(N1C)C(NC2=CC=CC=C2)=CC1=O** | Chloroform | - | - | 380 | 491 |

| | | | | | |
|---|---|---|---|---|---|
| **O=C(N1CC)C(NC2=CC=CC=C2)=CC1=O** | Chloroform | - | - | 380 | 498 |
| **O=C(N1CCC)C(NC2=CC=CC=C2)=CC1=O** | Chloroform | - | - | 380 | 497 |
| **O=C(N1CCCC)C(NC2=CC=CC=C2)=CC1=O** | Chloroform | - | - | 380 | 495 |
| **O=C(N1CCCCC)C(NC2=CC=CC=C2)=CC1=O** | Chloroform | - | - | 380 | 492 |
| **O=C(N1CCCCCC)C(NC2=CC=CC=C2)=CC1=O** | Chloroform | - | - | 380 | 492 |
| **O=C(N1CCCCCCCC)C(NC2=CC=CC=C2)=CC1=O** | Chloroform | - | - | 380 | 495 |
| **O=C(N1CCCCCCCCCCCC)C(NC2=CC=CC=C2)=CC1=O** | Chloroform | - | - | 380 | 494 |
| **O=C(N1C2CCCCC2)C(NC3=CC=CC=C3)=CC1=O** | Chloroform | - | - | 380 | 492 |
| **O=C(N1CCO)C(NC2=CC=CC=C2)=CC1=O** | Chloroform | - | - | 382 | 516 |
| **O=C(N1CCCO)C(NC2=CC=CC=C2)=CC1=O** | Chloroform | - | - | 383 | 494 |
| **O=C(N1CCCCO)C(NC2=CC=CC=C2)=CC1=O** | Chloroform | - | - | 381 | 496 |
| **O=C(N1CC(O)C)C(NC2=CC=CC=C2)=CC1=O** | Chloroform | - | - | 382 | 508 |
| **O=C(N1C[C@H](O)C)C(NC2=CC=CC=C2)=CC1=O** | Chloroform | - | - | 382 | 507 |
| **O=C(N1C[C@@H](O)C)C(NC2=CC=CC=C2)=CC1=O** | Chloroform | - | - | 382 | 507 |
| **O=C(N1C2=CC=C(N(C3=CC=CC=C3)C4=CC=CC=C4)C=C2)C(CC1=O)NCC(C)C** | Dichloroethane | - | 25100 | 304 | 374 |
| **O=C(N1CC2=CC=CC=C2)C(C3=CC=C(OC)C=C3)=C(C4=CC=C(OC)C=C4)C1=O** | Dioxane | 0.39 | - | 402 | 540 |
| **O=C(N1CC2=CC=CC=C2)C(C3=CC=C(OC)C=C3)=C(C4=CC=C(OC)C=C4)C1=O** | THF | 0.43 | - | 406 | 544 |
| **O=C(N1CC2=CC=CC=C2)C(C3=CC=C(OC)C=C3)=C(C4=CC=C(OC)C=C4)C1=O** | DMSO | 0.24 | - | 406 | 564 |
| **O=C(N1CC2=CC=CC=C2)C(C3=CC=C(OC)C=C3)=C(C4=CC=C(OC)C=C4)C1=O** | DMF | 0.27 | - | 404 | 556 |
| **O=C(N1CC2=CC=CC=C2)C(C3=CC=C(OC)C=C3)=C(C4=CC=C(OC)C=C4)C1=O** | Acetonitrile | 0.24 | - | 400 | 558 |
| **O=C(N1CC2=CC=CC=C2)C(C3=CC=C(O)C=C3)=C(C4=CC=C(OC)C=C4)C1=O** | Dioxane | 0.24 | - | 404 | 540 |
| **O=C(N1CC2=CC=CC=C2)C(C3=CC=C(O)C=C3)=C(C4=CC=C(OC)C=C4)C1=O** | THF | 0.38 | - | 410 | 546 |
| **O=C(N1CC2=CC=CC=C2)C(C3=CC=C(O)C=C3)=C(C4=CC=C(OC)C=C4)C1=O** | DMSO | 0.04 | - | 413 | 568 |

| | | | | | |
|---|---|---|---|---|---|
| **O=C(N1CC2=CC=CC=C2)C(C3=CC=C(O)C=C3)=C(C4=CC=C(OC)C=C4)C1=O** | DMF | 0.11 | - | 411 | 564 |
| **O=C(N1CC2=CC=CC=C2)C(C3=CC=C(O)C=C3)=C(C4=CC=C(OC)C=C4)C1=O** | Acetonitrile | 0.23 | - | 401 | 558 |
| **O=C(N1CC2=CC=CC=C2)C(C3=CC=C(C)C=C3)=C(C4=CC=C(OC)C=C4)C1=O** | Dioxane | 0.32 | - | 393 | 526 |
| **O=C(N1CC2=CC=CC=C2)C(C3=CC=C(C)C=C3)=C(C4=CC=C(OC)C=C4)C1=O** | THF | 0.53 | - | 394 | 526 |
| **O=C(N1CC2=CC=CC=C2)C(C3=CC=C(C)C=C3)=C(C4=CC=C(OC)C=C4)C1=O** | DMSO | 0.34 | - | 396 | 554 |
| **O=C(N1CC2=CC=CC=C2)C(C3=CC=C(C)C=C3)=C(C4=CC=C(OC)C=C4)C1=O** | DMF | 0.36 | - | 394 | 545 |
| **O=C(N1CC2=CC=CC=C2)C(C3=CC=C(C)C=C3)=C(C4=CC=C(OC)C=C4)C1=O** | Acetonitrile | 0.35 | - | 390 | 545 |
| **O=C(N1CC2=CC=CC=C2)C(C3=CC=C(Cl)C=C3)=C(C4=CC=C(OC)C=C4)C1=O** | Dioxane | 0.71 | - | 392 | 526 |
| **O=C(N1CC2=CC=CC=C2)C(C3=CC=C(Cl)C=C3)=C(C4=CC=C(OC)C=C4)C1=O** | THF | 0.35 | - | 394 | 529 |
| **O=C(N1CC2=CC=CC=C2)C(C3=CC=C(Cl)C=C3)=C(C4=CC=C(OC)C=C4)C1=O** | DMSO | 0.24 | - | 394 | 550 |
| **O=C(N1CC2=CC=CC=C2)C(C3=CC=C(Cl)C=C3)=C(C4=CC=C(OC)C=C4)C1=O** | DMF | 0.36 | - | 392 | 548 |
| **O=C(N1CC2=CC=CC=C2)C(C3=CC=C(Cl)C=C3)=C(C4=CC=C(OC)C=C4)C1=O** | Acetonitrile | 0.39 | - | 389 | 543 |
| **O=C(N1C)C(C2CN(CC3=CC=CC=C3)C4=C2C=CC=C4)=C(C5=CN(CC6=CC=CC=C6)C7=CC=CC=C57)C1=O** | DCM | 0.15 | - | 464 | 606 |
| **O=C(N1C)C(C2CN(CC3=CC=C(C[N])C=C3)C4=C2C=CC=C4)=C(C5=CN(CC6=CC=C(C[N])C=C6)C7=CC=CC=C57)C1=O** | DCM | 0.27 | - | 452 | 598 |
| **O=C(N1CC2=CC=C(C[N])C=C2)C(C3CN(CC4=CC=CC=C4)C5=C3C=CC=C5)=C(C6=CN(CC7=CC=CC=C7)C8=CC=CC=C68)C1=O** | DCM | 0.1 | - | 476 | 617 |
| **O=C(N1CC2=CC=CC=C2)C(C3CN(CC4=CC=CC=C4)C5=C3C=CC=C5)=C(C6=CN(CC7=CC=CC=C7)C8=CC=CC=C68)C1=O** | DCM | 0.165 | - | 461 | 605 |
| **O=C(N1CCCCCCN2C(C(C3CN(CC)C4=C3C=CC=C4)=C(C5=CN(CC)C6=CC=CC=C56)C2=O)=O)C(C7CN(CC)C8=C7C=CC=C8)=C(C9=CN(CC)C%10=CC=** | DCM | 0.037 | - | 482 | 623 |

| | | | | | |
|---|---|---|---|---|---|
| **CC=C9%10)C1=O** | | | | | |
| **O=C(N1CCCCCCN2C(C(C3CN(CCCCCCCCCCCC)C4=C3C=CC=C4)=C(C5=CN(CCCCCCCCCCCC)C6=CC=CC=C56)C2=O)=O)C(C7CN(CCCCCCCCCCCC)C8=C7C=CC=C8)=C(C9=CN(CCCCCCCCCCCC)C%10=CC=CC=C9%10)C1=O** | DCM | 0.031 | - | 485 | 622 |
| **O=C(N1)C(C2=CNC3=C2C=CC=C3)=C(C4=CNC5=C4C=CC=C5)C1=O** | THF | 0.31 | 5300 | 366 | 595 |
| **O=C(N1)C2=C(C(C(C(C=CC=C3)=C3N4)=C4C5=C2C6=C(C=CC=C6)N5)C1=O** | THF | 0.42 | 42200 | 315 | 507 |
| **O=C1NNC(C2=C1C(C(C(C=CC=C3)=C3N4)=C4C5=C2C6=C(C=CC=C6)N5)=O** | THF | 0.02 | 42700 | 316 | 517 |
| **O=C(N1C)C(C2=CN(C)C3=C2C=CC=C3)=C(C4=CN(C)C5=C4C=CC=C5)C1=O** | THF | 0.36 | 5610 | 373 | 626 |
| **O=C(N1C)C2=C(C(C(C(C=CC=C3)=C3N4C)=C4C5=C2C6=C(C=CC=C6)N5C)C1=O** | THF | 0.58 | 35700 | 323 | 538 |
| **O=C1NNC(C2=C1C=CC=C2N)=O** | THF | 0.11 | 7970 | 295 | 399 |

*Table 1: Training Set Information, including photophysical properties, solvent and SMILES.*

# Training Set Sources

***Reference 1:*** Rational design of substituted maleimide dyes with tunable fluorescence and solvafluorochromism, Y. Xie, J. T. Husband, M. Torrent-Sucarrat, H. Yang, W. Liu, and R. K. O’Reilly, 2018, Chem. Commun., 2018, 54 (27), 3339-3342. DOI:10.1039/C8CC00772A

***Reference 2:*** Aminomaleimide fluorophores: A simple functional group with bright, solvent dependent emission, A. B. Mabire, M. P. Robin, W.-D. Quan, H. Willcock, V. G. Stavros, R. K. O’Reilly, Chem. Commun, 2015, 51, 9733-9736, DOI:10.1039/C5CC02908B

***R***

***3:*** Conjugation-Induced Fluorescent Labeling of Proteins and Polymer Using Dithiomaleimides, M. Robin, P. Wilson, A. Mabire, J. Kiviaho, J. Raymond, D.M. Haddleton and R. K. O’Reilly, J. Am. Chem. Soc. 2013, 135, 2875-2878. DOI: 10.1021/ja3105494

***Reference 4:*** Rigidochromism by imide functionalisation of an aminomaleimide fluorophore, Jonathan T. Husband, Yujie Xie, Thomas R. Wilks, Louise Male, Miquel Torrent-Sucarrat, Vasilios G. Stavros and Rachel K. O'Reilly *, Chem. Sci., 2021, 12, 10550, DOI: 10.1039/d1sc03307g.

***Reference 5:*** Unpublished results- Yujie Xie thesis (https://wrap.warwick.ac.uk/160943/).

**Note:** Fluorescent quantum yields were measured by a relative method using quinine sulphate as standard reference ($\Phi f$ = 59%).

**Note:** Only the π-π* transition peak was recorded here. Another transition peak around 230-290 nm was not included. As this oftentimes does not contribute to fluorescent behaviour.

## Training Set Solvent Composition

From our literature search, we observed a range of solvents typically utilised for the solvatochromic study of maleimide derivatives. In particular, non-polar solvents (diethyl ether and cyclohexane) found good utility, with intermediate polarity solvents, such as dioxane and THF also appearing. The polar solvents used typically included methanol, DMSO and water.

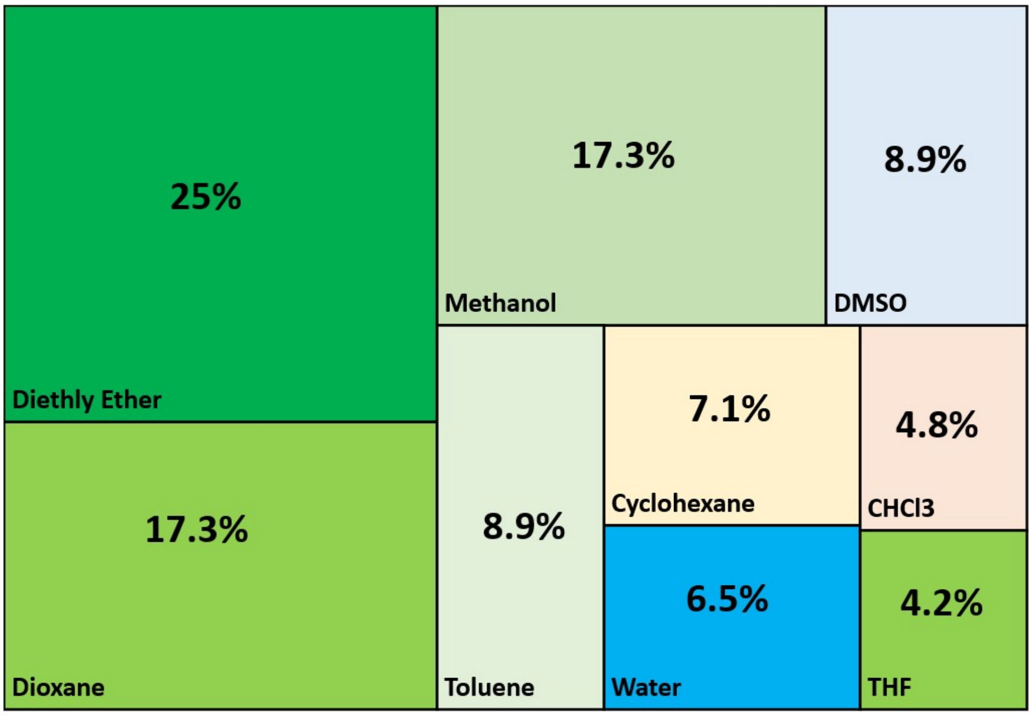


*Figure 1: Training Set Solvent Composition*

# Synthesis and characterisation

## Starting material and precursor preparation

To obtain the maleimide derivatives, di-halogenated maleimides were synthesised from their corresponding maleic anhydrides. Where dibromomaleic anhydride was synthesised *via* bromination with neat bromine in the presence of an aluminium chloride catalyst according to a literature process (***Tetrahedron, 2005, vol. 61, # 19, p. 4585 – 4593***). The halogenated maleic anhydrides then were refluxed in glacial acetic acid with the corresponding amine for 4 hours according to a previously reported method: ***Bioorg. Med. Chem. Lett. 21 (2011) 4577–4580.***

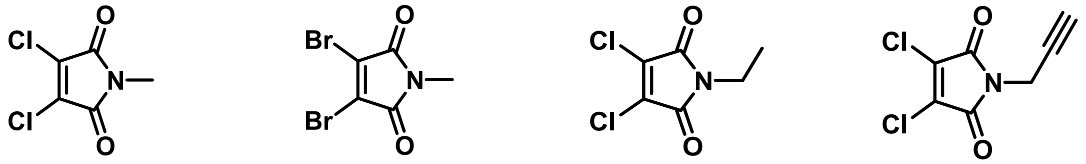


| **DCM (Dichloro methyl maleimide)** | **DBM (Dibromo methyl maleimide)** | **DCEM (Dichloro ethylmaleimide)** | **DCPM (Dichloro propargylmaleimide)** |
|---|---|---|---|

*Figure 2: Initial maleimides structures for future functionalisation*

## DCM, Dichloro N-methyl maleimide

2,3-Dichloromaleic anhydride was transformed into the corresponding maleimide using methylamine (1.2 equivalents) with a weak base (3 equivalents), in the presence of acetic acid (15 mL). The solution was heated under reflux for four hours before the removal of solvent *in vacuo*. The crude solid was then taken up with 150mL of dichloromethane and washed once with saturated sodium carbonate solution (30 mL), before three water washes (3x30 mL) and a final brine wash (30 mL). The organic solution was dried over anhydrous magnesium sulphate and the resulting solution was dried *in vacuo*.

*$^{1}H$ NMR (400 MHz, Chloroform-d) δ 3.05 (s, 1H). $^{13}C$ NMR (101 MHz, Chloroform-d) δ 163.07, 133.34, 25.08.*

*Compared to 2,3-dichloromaleic anhydride $^{13}C$ NMR (101 MHz, Chloroform-d) δ 157.37, 135.79. (No protons to measure).*

## DCEM, Dichloro N-ethylmaleimide

*We used the same procedure as outlined above in the synthesis of dichloro N-methyl maleimide to obtain our imide-functionalised maleimide. 2,3-*Dichloromaleic anhydride was dissolved in acetic acid, before the addition of ethylamine hydrochloride and sodium acetate. The resulting solution was heated under reflux for four hours and the reaction was monitored *via* TLC. The solution was cooled, evaporated under reduced pressure and a steady flow of air, before the crude oil was taken up with 150mL of DCM, washing with saturated sodium carbonated solution (30 mL), three separate water washes (30 mL each)

and brine (30 mL). The resulting solution was dried over magnesium sulphate and evaporated under reduced pressure to afford the solid product and used without further purification.

## DBM, Dibromo N-methyl maleimide

*We used the same procedure as outlined previously in the synthesis of dichloro N-methyl maleimide to obtain our imide-functionalised maleimide, as additional accounts displayed success of this reaction with the bromo-analogue.*

To an oven-dried 50 mL round bottom flask was added dibromomaleic anhydride (11.77 mmol, 3.0152 g), methylamine hydrochloride salt (14.155 mmol, 0.9626 g) and sodium acetate (17.7 mmol, 1.4514 g). The solids were dissolved in acetic acid (20 mL) to give a yellow solution. A reflux condenser was attached, and the solution was heated to 80°C for four hours. Thin layer chromatography was used to assess the extent of the reaction after four hours, where no starting material was observed. The solution was then left to cool, with the acetic acid concentrated under air flow and neutralisation *via* saturated sodium bicarbonate washes. Accumulated salt solids were filtered off, and the resulting solution was diluted with dichloromethane (100 mL) and washed with three portions of distilled water (three 50 mL portions) and a final brine wash (50 mL). The organic phase was then dried over magnesium sulphate and concentrated *in vacuo* to afford a crude orange oil. Diethyl ether recrystallisation was performed to afford an orange solid (4.243 mmol, 1.1457 g) in 36% yield. $^1$H NMR was used to identify the characteristic methyl peak and identify any possible contaminants. The product was used without further purification.

## DCPM, Dichloro N-propargyl maleimide

*We used the same procedure as outlined previously in the synthesis of dichloro N-methyl maleimide to obtain our imide-functionalised maleimide, as additional accounts displayed success of this reaction with the use of propargylamine.*

# Addition-Elimination Mechanism

To functionalise the maleimide precursors with heteroatom reagents, the reagents were treated with a base, to generate the stronger corresponding nucleophile, before proceeding *via* an addition-elimination reaction, resulting in the elimination of a halogen. The choice of base here is important, and we opted to use triethylamine for the majority of reactions, as this reagent is a strong, but non-nucleophilic base.

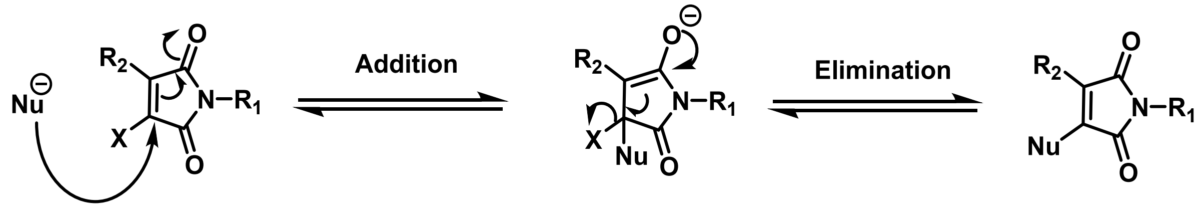


*Figure 3: Maleimide addition-elimination mechanism*

# General procedure for AM synthesis

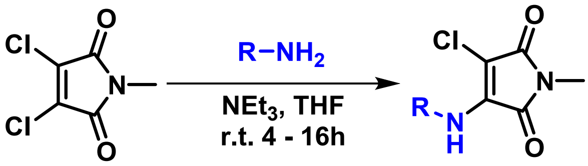


*Figure 4: General procedure for synthesis of AMs*

Aminomaleimides (AM) were synthesised *via* an addition/ elimination route, where an amine was added to solubilised maleimide (in THF), with the addition of excess triethylamine as a strong, non-nucleophilic base. Reactions were monitored *via* TLC analysis to assess completion, which typically ran for between 4- 16 hours. The reaction mixture was diluted in dichloromethane, and washed with ammonium chloride (10 mL), and water (3x 20 mL), before drying over magnesium sulphate and drying under vacuum.

The general procedure to synthesise AMs was adapted from ***Chem. Commun., 2015,51, 9733-9736.***

## **1a** (3-chloro-1-methyl-4-(propylamino)-1H-pyrrole-2,5-dione)

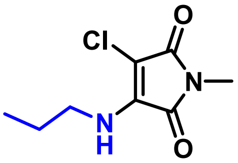


The synthetic procedure followed the general addition/elimination mechanism outlined above. In a dried-round bottom flask, DCM (2.7 mmol, 0.5g) was dissolved in 10 mL of glacial acetic acid. To this, sodium acetate was added with stirring (5.45 mmol, 0.45g) and stirred for several minutes. Propylamine was added dropwise *via* a syringe (5.4 mmol, 0.44 mL). The solution was stirred overnight at room temperature, where a colour change was observed from a dull yellow to a deep orange. The acetic acid was removed *via* air pressure, where the crude solid was taken up with dichloromethane (50 mL) and washed with distilled water (3x20 mL), brine (20 mL) and dried over magnesium sulphate. The yellow solution was dried *in vacuo* to afford a yellowish solid, which was recrystallised from hexane in 20.2% yield. The product was confirmed using both proton and carbon NMR.

*$^{1}$H NMR (400 MHz, Chloroform-d) δ 5.33 (d, J = 28.5 Hz, 1H), 3.63 – 3.51 (m, 2H), 2.99 (d, J = 1.8 Hz, 3H), 1.74 – 1.57 (m, 2H), 0.98 (t, J = 7.4 Hz, 3H).*

*$^{13}$C NMR (101 MHz, Chloroform-d) δ 167.94, 165.78, 140.52, 88.74, 44.76, 30.11, 24.16, 24.04, 10.87.*

MS (ESI) *m/z* calculated 202.0504, found 202.0505.

FTIR (cm$^{-1}$): *3335- sharp, 3010-2805 broad, 1703- sharp, 1642- sharp.*

## **1b** (3-chloro-1-ethyl-4-(isopropylamino)-1H-pyrrole-2,5-dione)

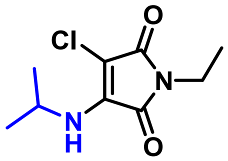

DCEM (0.75 mmol, 156 mg) was dissolved in THF (5 mL) and stirred for several minutes, yielding a faint yellow solution. To this solution, sodium hydrogen carbonate (1.24 mmol, 104 mg) was added before the dropwise addition of isopropylamine (1.13 mmol, 0.1 mL). Upon addition of the amine, the solution appeared a darker yellow. The solution was analysed *via* TLC, which indicated starting material was still present. The solution was left overnight, and the next day, a darker, cloudier solution was observed. TLC analysis was carried out once more, where a distinct difference in RF was observed between the reaction spot and the co-spot. The solution was diluted with additional dichloromethane (50 mL) and washed with distilled water (3x20 mL). The yellow solution was dried with magnesium sulphate and a rotary evaporator to produce a yellow solid powder in a 74.4% yield. The product was confirmed using both proton and carbon NMR.

*$^{1}$H NMR (400 MHz, Chloroform-d) δ 5.16 (s, 1H), 4.28 (dddd, J = 12.0, 8.9, 7.0, 6.1 Hz, 1H), 3.54 (qd, J = 7.2, 1.3 Hz, 2H), 1.29 (dd, J = 6.4, 1.0 Hz, 6H), 1.17 (td, J = 7.2, 1.3 Hz, 3H).*

*$^{13}$C NMR (101 MHz, Chloroform-d) δ 167.95, 165.83, 139.63, 88.61, 45.14, 33.50, 24.01, 14.18.*

MS (ESI) *m/z* calculated 216.0660, found 216.0662.

FTIR (cm$^{-1}$): *3312- sharp, 2998-2805 broad, 1701- sharp, 1642- sharp.*

## **1c** (3-chloro-1-methyl-4-(pentan-3-ylamino)-1H-pyrrole-2,5-dione)

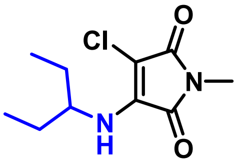

DCM (1.43 mmol, 256.7 mg) was dissolved in 8 mL of THF along with sodium carbonate (2.88 mmol, 305 mg). 3-amino pentane (1.7 mmol, 0.2 mL) was added dropwise to the pale-yellow solution, which instigated a colour change to a strong yellow. After overnight stirring, the solution was analysed *via* TLC, where no starting material was observed. The solution was diluted with dichloromethane and washed with three portions of distilled water (3x 20 mL). The resulting yellow solution was dried with magnesium sulphate and concentrated under reduced pressure. A yellow solid was obtained in 48.1% yield. The product was confirmed using both proton and carbon NMR.

*$^{1}$H NMR (400 MHz, Chloroform-d) δ 5.09 (d, J = 9.1 Hz, 1H), 3.94 (dtt, J = 10.2, 7.6, 5.3 Hz, 1H), 3.00 (d, J = 0.9 Hz, 3H), 1.77 – 1.57 (m, 2H), 1.57 – 1.40 (m, 2H), 0.94 (t, J = 7.4 Hz, 6H).*

*$^{13}$C NMR (101 MHz, Chloroform-d) δ 168.01, 165.86, 140.52, 55.95, 28.16, 25.38, 24.17, 9.96.*

MS (ESI) *m/z* calculated 230.0817, found 230.0819.

FTIR (cm$^{-1}$): *3324- sharp, 2985-2771 broad, 1707- sharp, 1639- sharp.*

## **1d** (3-(tert-butylamino)-4-chloro-1-methyl-1H-pyrrole-2,5-dione)

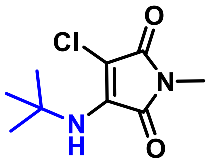


DCM (0.5627 mmol, 101.3 mg) was dissolved in THF (10 mL) to afford a yellow solution. To this was added an excess of sodium carbonate (8.679 mmol, 92 mg). *Tert*-butylamine (0.672 mmol, 0.07 mL) was then added *via* a mechanical pipette and the resulting solution was left to stir overnight for 16 hours. The deeper yellow solution was diluted with dichloromethane (50 mL) and washed with saturated ammonium chloride solution (10 mL), three distilled water washes (20 mL each) and a final brine wash (20 mL) before dried over magnesium sulphate and concentration under reduced pressure to afford a yellow solid (98 mg) in 81% yield.

*$^{1}$H NMR (400 MHz, Chloroform-d) δ 5.56 (s 1H), 2.95 (s, 3H), 1.42 (s, 9H).*

$^{13}$C NMR (101 MHz, Chloroform-*d*) δ 168.61, 166.69, 138.77, 87.81, 52.57, 31.04, 24.53.

MS (ESI) *m/z*= 216.0660, calculated= 216.0662.

FTIR (cm$^{-1}$): *3315- sharp, 3014-2833 broad, 1697- sharp, 1632- sharp.*

## **1e** (3-chloro-4-(cyclopropylamino)-1-methyl-1H-pyrrole-2,5-dione)

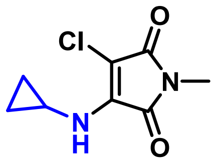


DCM (1.41 mmol, 253.8 mg) was dissolved in THF (10 mL), leaving a pale-yellow solution. Sodium carbonate (2.75 mmol, 291.4 mg) was added to the solution along with cyclopropylamine (1.65 mmol, 0.115 mL). When the amine was added, the pale-yellow solution changed to a bright yellow solution, which also had a very strong UV signal when checked in a lightbox. The solution was left to stir

overnight at room temperature after an initial TLC. The solution was diluted with 50mL of dichloromethane, before being washed with a 30 mL portion of saturated ammonium chloride and three portions (3x30 mL) of distilled water. A brine wash and magnesium sulphate drying treatment were also performed on the solution. Once dried under reduced pressure, a yellow solid was obtained and recrystallised from cyclohexane, resulting in a 72.5% yield and this solid displayed strong solid-state fluorescence. The product was confirmed using both proton and carbon NMR.

*$^1$H NMR (400 MHz, Chloroform-d) δ 5.46 (s, 1H), 2.98 (s, 4H), 0.96 – 0.81 (m, 2H), 0.77 – 0.66 (m, 2H).*

*$^{13}$C NMR (101 MHz, Chloroform-d) δ 167.86, 165.77, 141.19, 91.37, 25.19, 24.11, 8.99.*

MS (ESI) *m/z* calculated 200.0347, found 200.0349.

FTIR (cm$^{-1}$): *3307- sharp, 3041-2840 broad, 1706- sharp, 1652- sharp.*

## **1f** (3-chloro-4-(cyclobutylamino)-1-methyl-1H-pyrrole-2,5-dione)

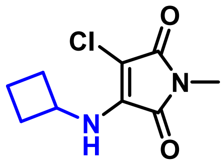


DCM (1.42 mmol, 254.6 mg) was dissolved in THF (12 mL) along with sodium carbonate (2.79 mmol, 296 mg) to this was added cyclobutylamine (1.77 mmol, 0.15 mL) dropwise which immediately changed the solution from pale-yellow to orange. After overnight stirring, the solution was diluted in 50 mL of dichloromethane, then washed with one 30mL portion of saturated ammonium chloride. Three portions of distilled water (30 mL) were used to wash the orange solution before a brine rinse. The solution was dried over magnesium sulphate to leave a red solution and eventual solid in 86.6% yield. The product was confirmed using both proton and carbon NMR.

*$^1$H NMR (400 MHz, Chloroform-d) δ 5.52 (d, J = 8.3 Hz, 1H), 4.64 – 4.36 (m, 1H), 2.97 (d, J = 0.7 Hz, 3H), 2.40 (dddd, J = 12.1, 7.7, 5.8, 3.0 Hz, 2H), 2.08 – 1.92 (m, 2H), 1.85 – 1.67 (m, 2H).*

*$^{13}$C NMR (101 MHz, Chloroform-d) δ 167.86, 165.81, 139.39, 89.39, 48.08, 32.38, 24.17, 14.67.*

MS (ESI) *m/z* calculated 214.0504, found 214.0505.

FTIR (cm$^{-1}$): *3316- sharp, 3017-2805 broad, 1697- sharp, 1650- sharp.*

## **1g** (3-chloro-4-(cyclopentylamino)-1-methyl-1H-pyrrole-2,5-dione)

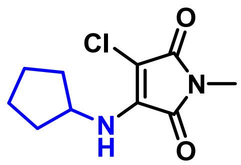


DCM (1.4 mmol, 252.3 mg) was dissolved in 10 mL of THF with sodium carbonate (2.75 mmol, 291.4 mg). The pale-yellow solution was stirred for several minutes before the dropwise addition of cyclopentylamine (1.65 mmol, 0.16 mL), where a deeper yellow colour was observed, along with an opaque characteristic. After overnight stirring, no further colour change was noted, and TLC analysis indicated a lack of starting material, the solution was diluted with 50mL of dichloromethane, washed with a 30mL portion of ammonium chloride, a further three 30mL portions of distilled water, brine and dried over magnesium sulphate. The orange solution was concentrated under reduced pressure to afford an orange solid with 49.6% yield. The product was confirmed using both proton and carbon NMR.

*$^{1}$H NMR (400 MHz, Chloroform-d) δ 5.35 – 5.11 (m, 1H), 4.47 – 4.22 (m, 1H), 2.93 (s, 3H), 2.08 – 1.38 (m, 8H).*

*$^{13}$C NMR (101 MHz, Chloroform-d) δ 167.96, 165.82, 139.96, 88.65, 54.48, 34.71, 24.15, 23.80.*

MS (ESI) *m/z* calculated 228.0660, found 228.061.

FTIR (cm$^{-1}$): *3309- sharp, 2989-2814 broad, 1701- sharp, 1643- sharp.*

## **1h** (3-chloro-4-(cyclohexylamino)-1-methyl-1H-pyrrole-2,5-dione)

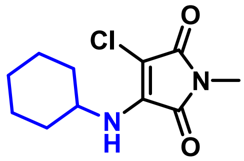


DCM (1.4 mmol, 252.4 mg) was dissolved in THF (10 mL) with sodium carbonate (2.72 mmol, 287.9 mg), giving a pale-yellow solution. To this, a dropwise addition of cyclohexylamine (1.7 mmol, 0.185 mL) was carried out to afford a deeper yellow solution. After TLC analysis and subsequent overnight stirring, the solution was a pale orange. This solution was diluted with 50 mL of dichloromethane, washed with a 30 mL portion of ammonium chloride, a further three 30 mL portions of distilled water, brine and dried over magnesium sulphate before concentrating under reduced pressure. This brown solid was analysed using NMR and purified using cyclohexane recrystallisation and column chromatography. The column was carried out using a 95% dichloromethane and 5% methanol solvent

system, which was gradually increased to 85:15. The orange solid obtained in 33.9% yield. The product was confirmed using both proton and carbon NMR.

*$^{1}H$ NMR (400 MHz, Chloroform-d) δ 5.18 (d, J = 9.0 Hz, 1H), 3.83 (dddd, J = 14.2, 10.4, 8.7, 3.9 Hz, 1H), 2.93 (s, 3H), 2.07 – 1.86 (m, 2H), 1.72 (dt, J = 12.8, 3.9 Hz, 2H), 1.66 – 1.50 (m, 1H), 1.41 – 1.02 (m, 6H).*

*$^{13}C$ NMR (101 MHz, Chloroform-d) δ 168.00, 165.90, 139.61, 88.21, 51.67, 34.11, 25.15, 24.41, 24.19.*

MS (ESI) *m/z* calculated 242.0817, found 242.0817.

FTIR ($cm^{-1}$): *3314- sharp, 2981-2739 broad, 1703- sharp, 1649- sharp.*

## **1i** (3-(allylamino)-4-chloro-1-methyl-1H-pyrrole-2,5-dione)

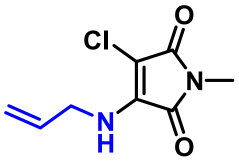


DCM (0.59 mmol, 106 mg) was dissolved in THF (6mL) and stirred for several minutes before sodium acetate (0.69 mmol, 57 mg) was added. Dropwise addition of allylamine (0.69 mmol, 0.06 mL) was achieved using a mechanical pipette. A colour change was observed from pale yellow to bright yellow. Overnight stirring was carried out due to TLC concerns, where a yellow, UV-active solution was observed. The solution was diluted with dichloromethane (50 mL) and washed three times with 20 mL portions of distilled water and one of brine before magnesium sulphate drying. The solution was further purified using column chromatography, using a 9:1 ratio of dichloromethane to methanol. An orange solid was recovered in a 50.8% yield. The product was confirmed using both proton and carbon NMR.

*$^{1}H$ NMR (400 MHz, Chloroform-d) δ 5.85 (ddt, J = 17.1, 10.3, 5.5 Hz, 1H), 5.33 (s, 1H), 5.26 – 5.16 (m, 2H), 4.17 (ddq, J = 6.9, 5.5, 1.7 Hz, 2H), 2.94 (s, 3H).*

*$^{13}C$ NMR (101 MHz, Chloroform-d) δ 166.79, 164.71, 139.13, 132.33, 130.49, 116.72, 88.90, 44.24, 39.39, 28.68, 23.21.*

MS (ESI) *m/z* calculated 200.0347, found 200.0350.

FTIR ($cm^{-1}$): *3326- sharp, 2981-2739 broad, 1703- sharp, 1650- sharp.*

## **1j** (3-chloro-1-methyl-4-(prop-2-yn-1-ylamino)-1H-pyrrole-2,5-dione)

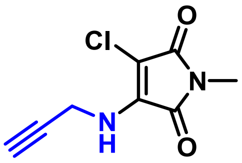


DCM (0.84 mmol, 151.7 mg) was dissolved in THF (10 mL) along with sodium carbonate (1.5 mmol, 161.5mg). To this pale-yellow solution, propargylamine was added dropwise (0.9 mmol, 0.06 mL), which quickly yielded an orange solution. The solution was left to stir overnight, before dilution with 50 mL of chloroform, three washes with 20 mL portions of distilled water, a brine wash and final magnesium sulphate drying. The orange/yellow solution was concentrated under reduced pressure with silica, where a first purification was carried out with an 8:2 ratio of dichloromethane and methanol. This first step removed numerous fractions of impurities, but the starting material was still observed in the analysis. A second column was employed with an increased polarity solvent system (now 30-35% methanol, 70-65% dichloromethane), to give an orange solid in 35.8% yield. The product was confirmed using both proton and carbon NMR.

*$^{1}H$ NMR (400 MHz, Chloroform-d) δ 5.41 (s, 1H), 4.39 (dd, J = 6.3, 2.5 Hz, 2H), 3.00 (s, 3H), 2.40 (t, J = 2.5 Hz, 1H).*

*$^{13}C$ NMR (101 MHz, Chloroform-d) δ 167.40, 165.46, 139.36, 78.46, 73.73, 33.02, 24.25.*

MS (ESI) *m/z* calculated 198.0191, found 198.0192.

FTIR ($cm^{-1}$): *3316- sharp, 3229- sharp, 3002-2795 broad, 1697- sharp, 1650- sharp.*

## **1k** (3-(allylamino)-4-chloro-1-(prop-2-yn-1-yl)-1H-pyrrole-2,5-dione)

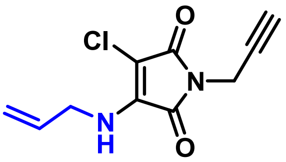


JB-028-3 (0.992 mmol, 202.4 mg) was dissolved in THF (10 mL) with sodium carbonate (1.82 mmol, 192.7 mg). A dark orange/brownish solution was observed, which had mild UV activity. To this, allylamine (1.2 mmol, 0.1 mL) was added dropwise. A colour change was noted, to a cloudy yellow solution. After overnight stirring, and TLC analysis, the solution was diluted with 50 mL of dichloromethane, washed with a 30 mL portion of ammonium chloride, a further three 30 mL portions of distilled water, brine and dried over magnesium sulphate before concentrating under reduced pressure. Orange oil was obtained and recrystallised from cyclohexane. The resulting solid was further

purified *via* column chromatography using petroleum spirit and ethyl acetate on a gradient of 9:1 to 6:4. The orange solid product obtained in 67.6% yield. The product was confirmed using both proton and carbon NMR.

*$^{1}$H NMR (400 MHz, Chloroform-d) δ 5.95 – 5.79 (m, 1H), 5.50 – 5.28 (m, 1H), 5.27 – 5.14 (m, 2H), 4.27 – 4.11 (m, 4H), 2.15 (t, J = 2.6 Hz, 1H).*

*$^{13}$C NMR (101 MHz, Chloroform-d) δ 166.15, 164.56, 140.35, 133.21, 117.82, 90.50, 71.71, 45.34, 40.42, 27.36.*

MS (ESI) *m/z* calculated 224.0347, found 224.0346.

FTIR (cm$^{-1}$): *3312- sharp, 3270- sharp, 3041-2827 broad, 1712- sharp, 1642- sharp.*

## **1l** (3-chloro-4-(cyclopropylamino)-1-(prop-2-yn-1-yl)-1H-pyrrole-2,5-dione)

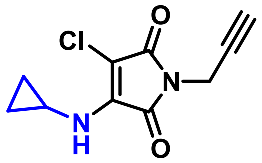


JB-028-3 (0.988 mmol, 201.6 mg) was dissolved in THF (10 mL) along with sodium carbonate (1.45 mmol, 137 mg). A pale-yellow solution changed to an orange colour upon the addition of cyclopropylamine (2.17 mmol, 0.09 mL). After overnight stirring, the murky orange solution was analysed *via* TLC to show a distinct difference in RF values between the reaction and starting material. This solution was diluted with 50 mL of dichloromethane, washed with two 30 mL portions of ammonium chloride (due to amine excess), three 30 mL portions of water, a final brine wash and magnesium sulphate drying. After the resulting solution was dried under reduced pressure, the yellow solid was confirmed using both proton and carbon NMR in 44.2% yield.

*$^{1}$H NMR (400 MHz, Chloroform-d) δ 5.43 (s, 1H), 4.19 (d, J = 2.5 Hz, 2H), 2.93 (dddd, J = 10.6, 6.9, 3.6, 2.9 Hz, 1H), 2.15 (t, J = 2.5 Hz, 1H), 0.89 – 0.74 (m, 2H), 0.74 – 0.58 (m, 2H).*

*$^{13}$C NMR (101 MHz, Chloroform-d) δ 165.23, 163.62, 140.40, 91.05, 75.92, 70.64, 26.28, 24.29, 8.10.*

MS (ESI) *m/z* calculated 224.0349, found 24.0349.

FTIR (cm$^{-1}$): *3324- sharp, 3250- sharp, 3031-2797 broad, 1711- sharp, 1650- sharp.*

# NMR spectra and analysis

All NMR spectra were recorded on a Bruker Avance III HD 400 spectrometer at room temperature (298 K) with a 400 MHz frequency. All proton scans were completed using the “high quality” option of 30 scans within a 28-minute setting, and all carbon spectra were recorded on the ‘dilute’ setting of 2500 scans to achieve higher quality spectra.

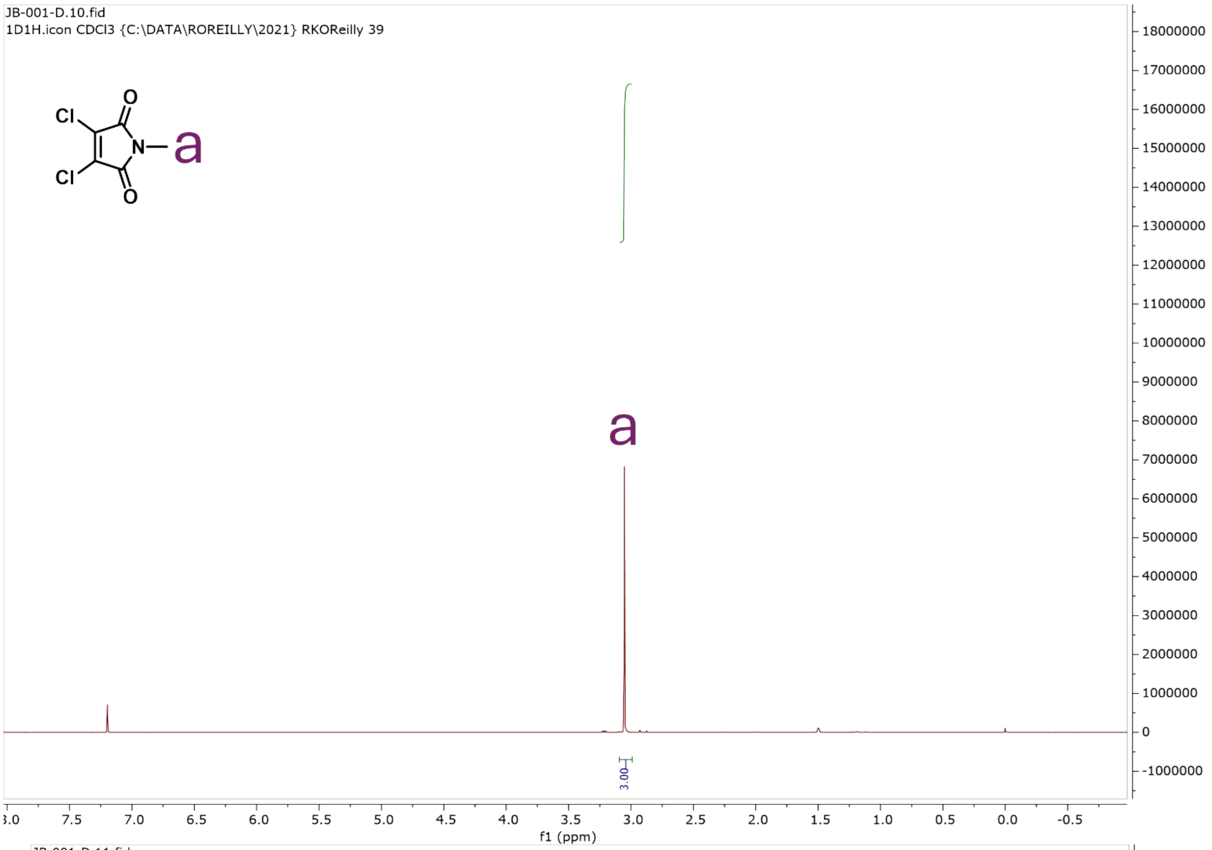


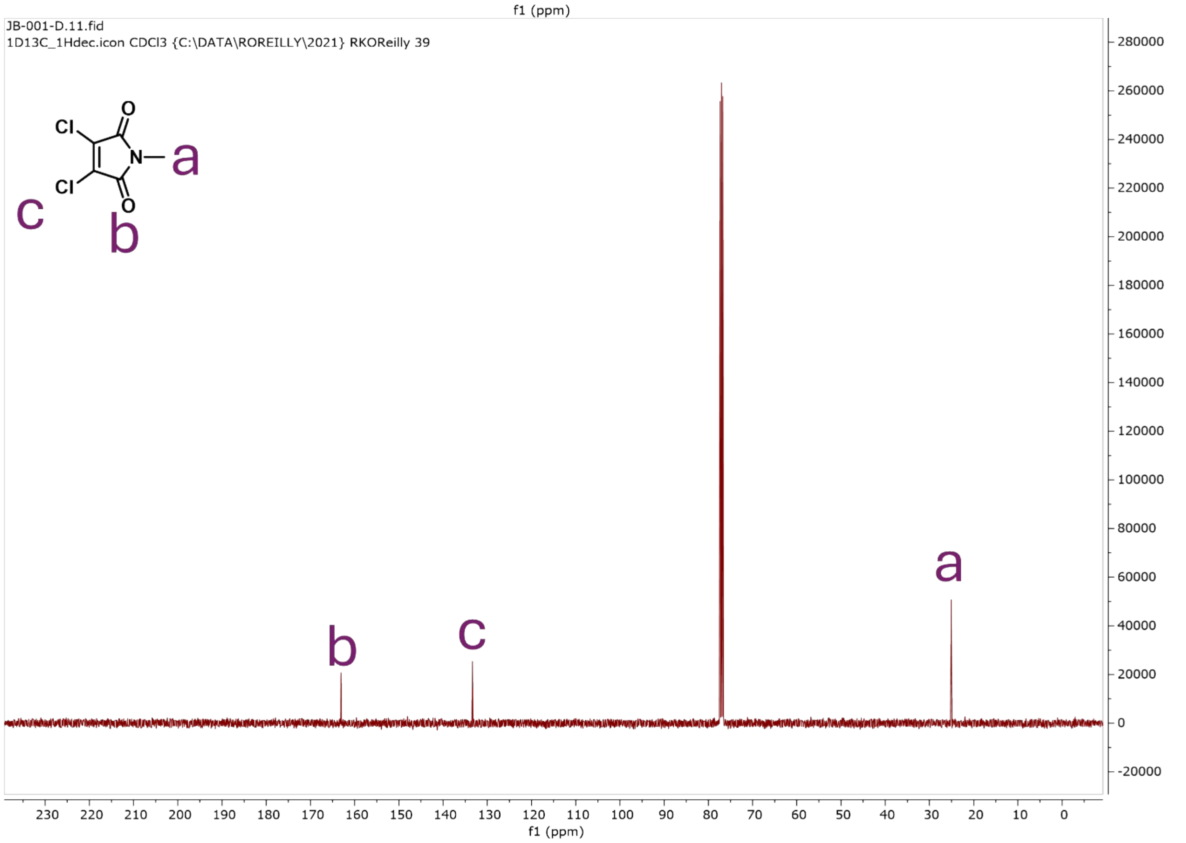


*Figure 5: $^{1}H$ and $^{13}C$ NMR Spectra of DCM*

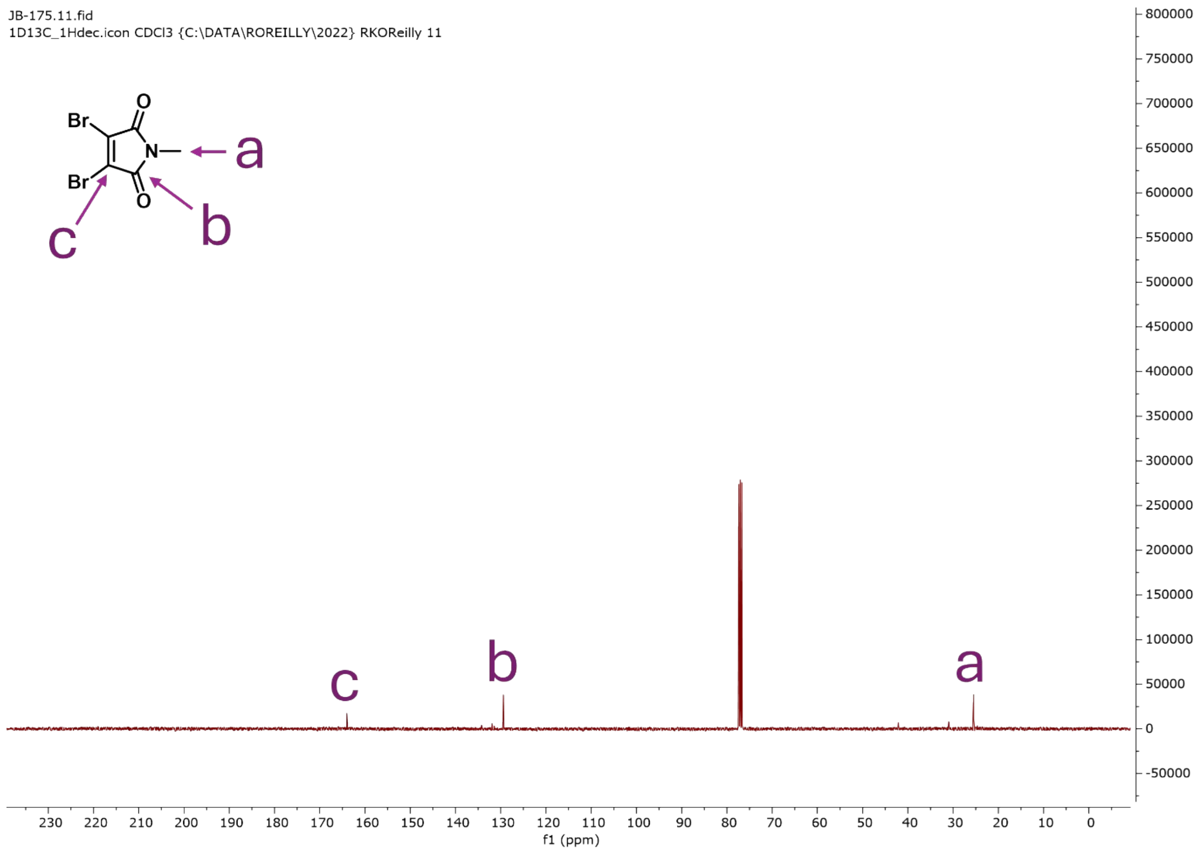


*Figure 6: $^{13}C$ NMR Spectra of DBM*

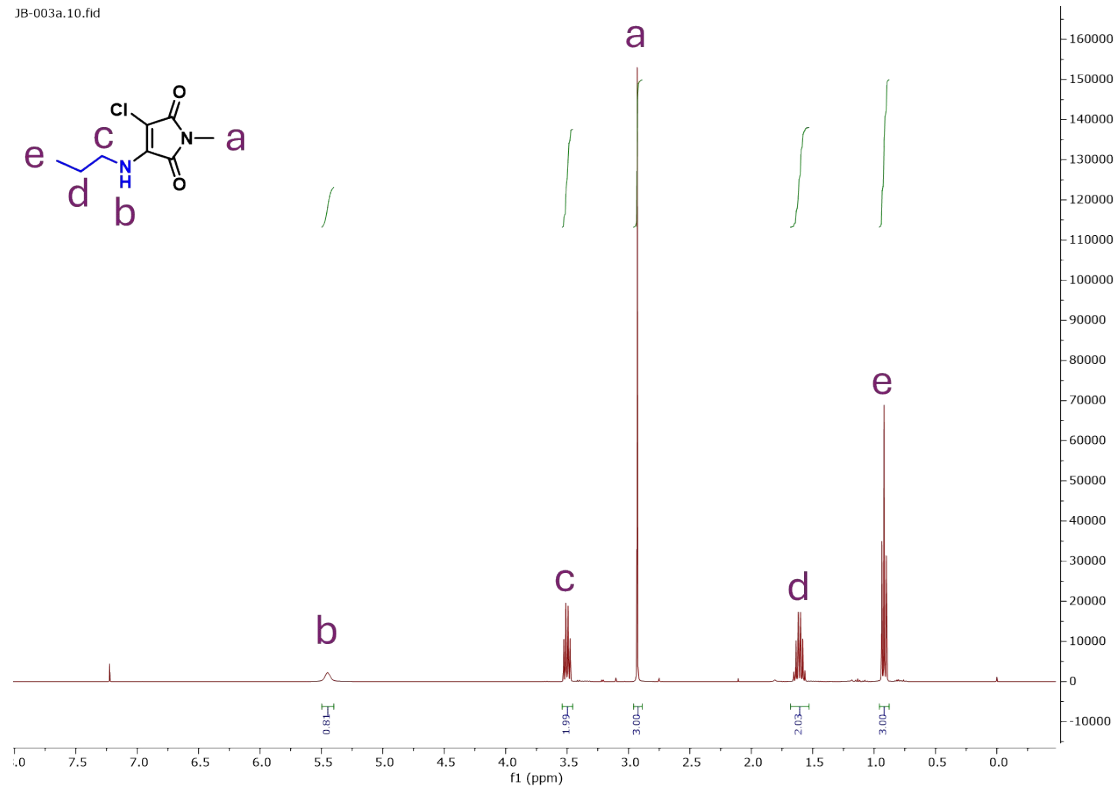


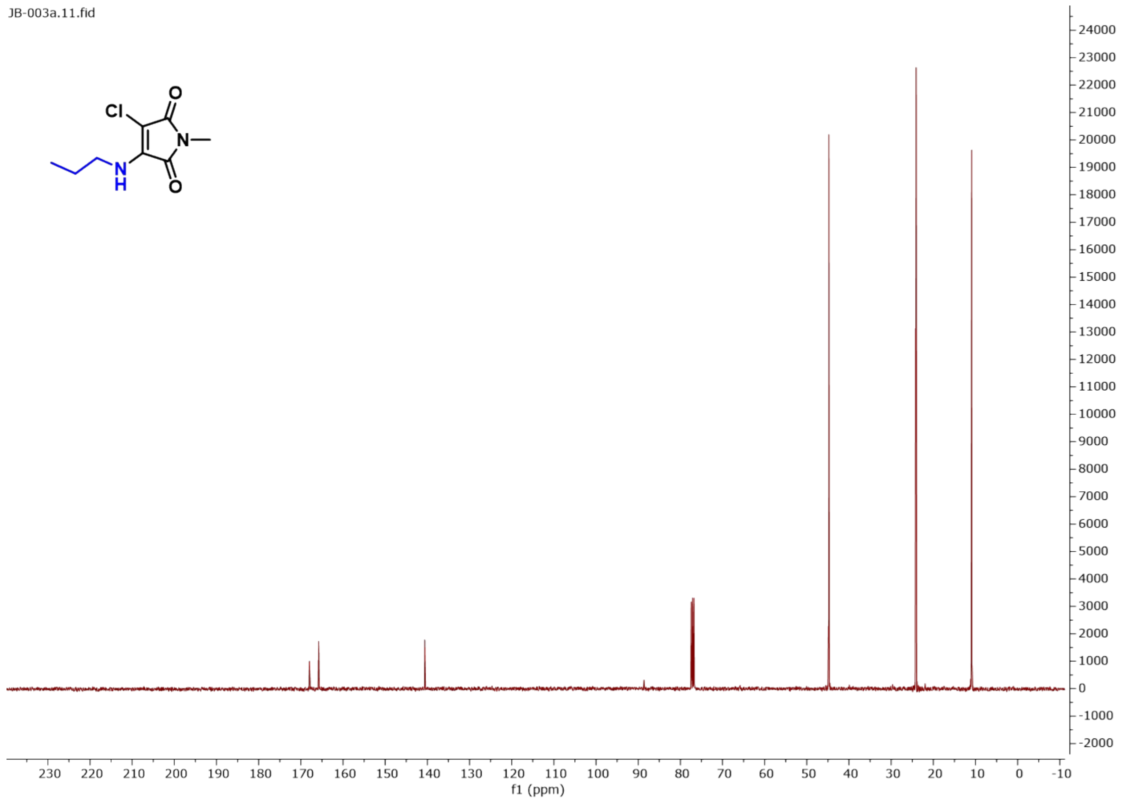


*Figure 7: $^{1}H$ and $^{13}C$ NMR Spectra of 1a*

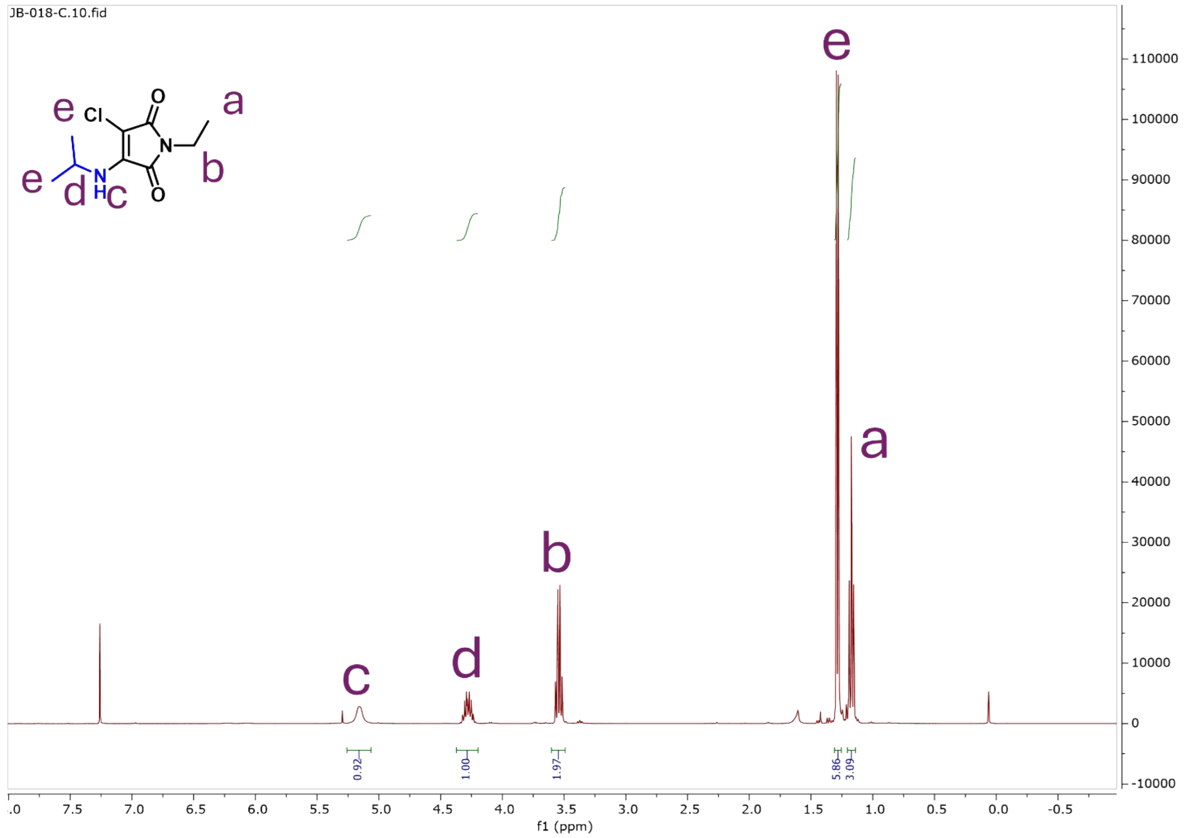


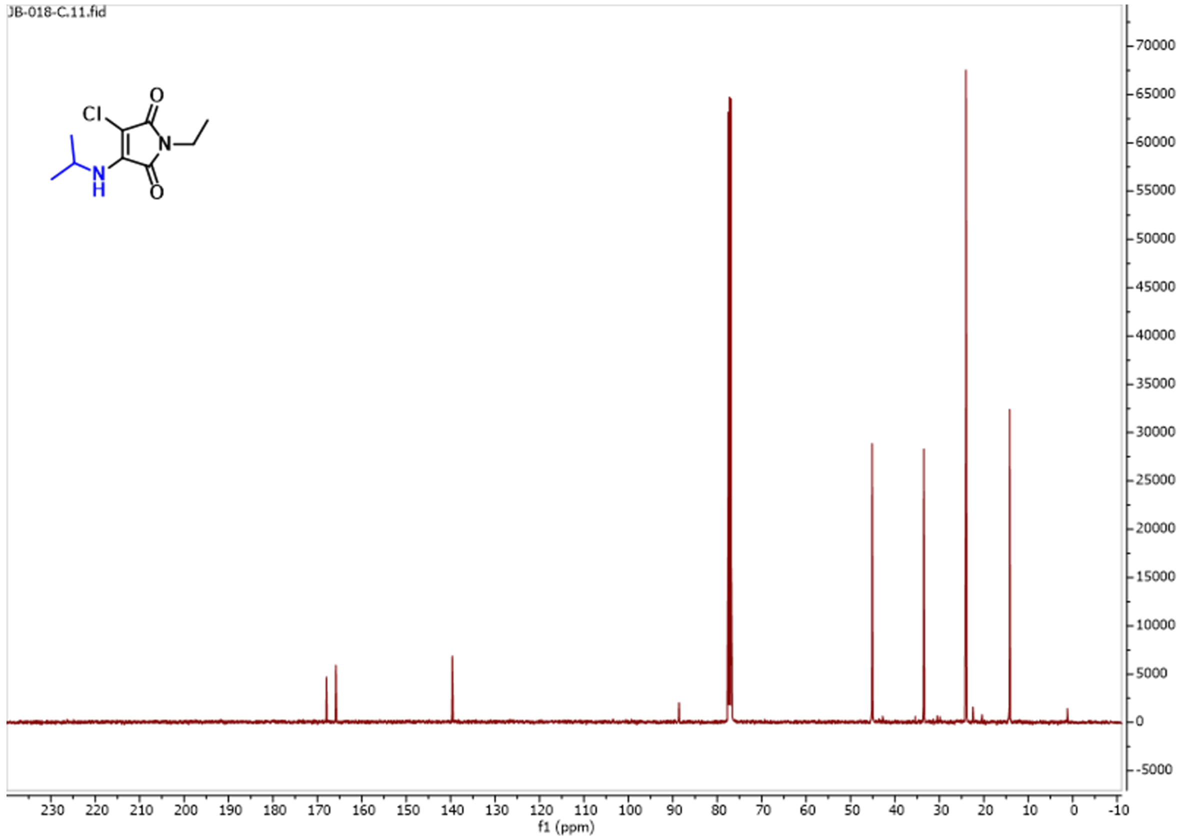


*Figure 8: $^{1}$H and $^{13}$C NMR Spectra of 1b*

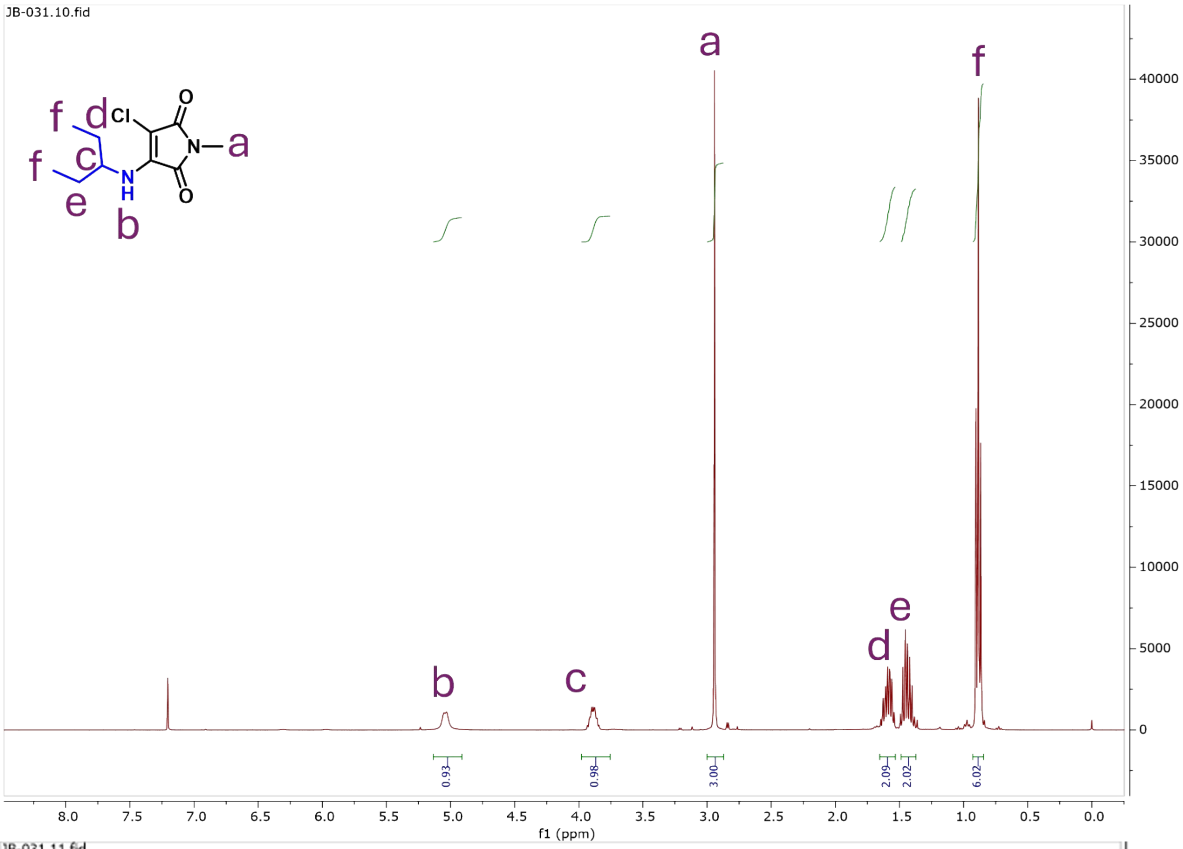


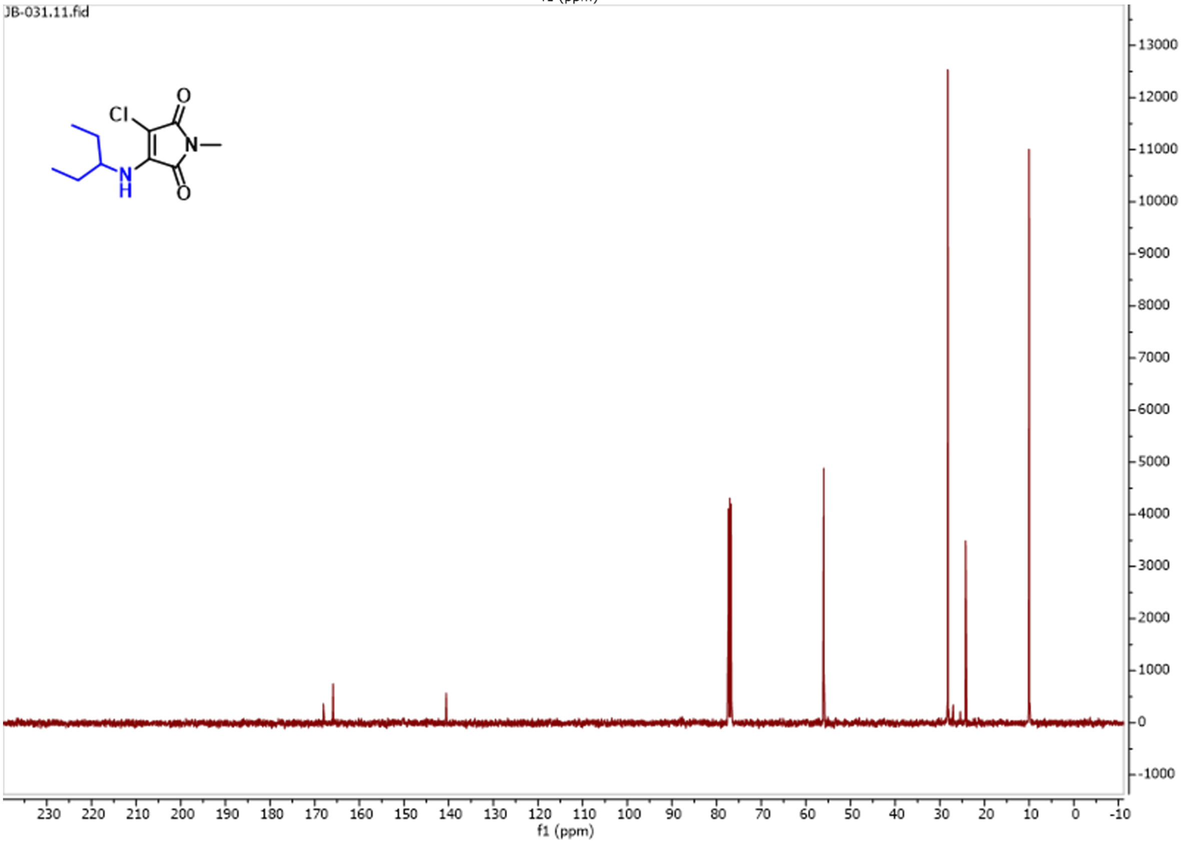


*Figure 9: $^{1}H$ and $^{13}C$ NMR Spectra of 1c*

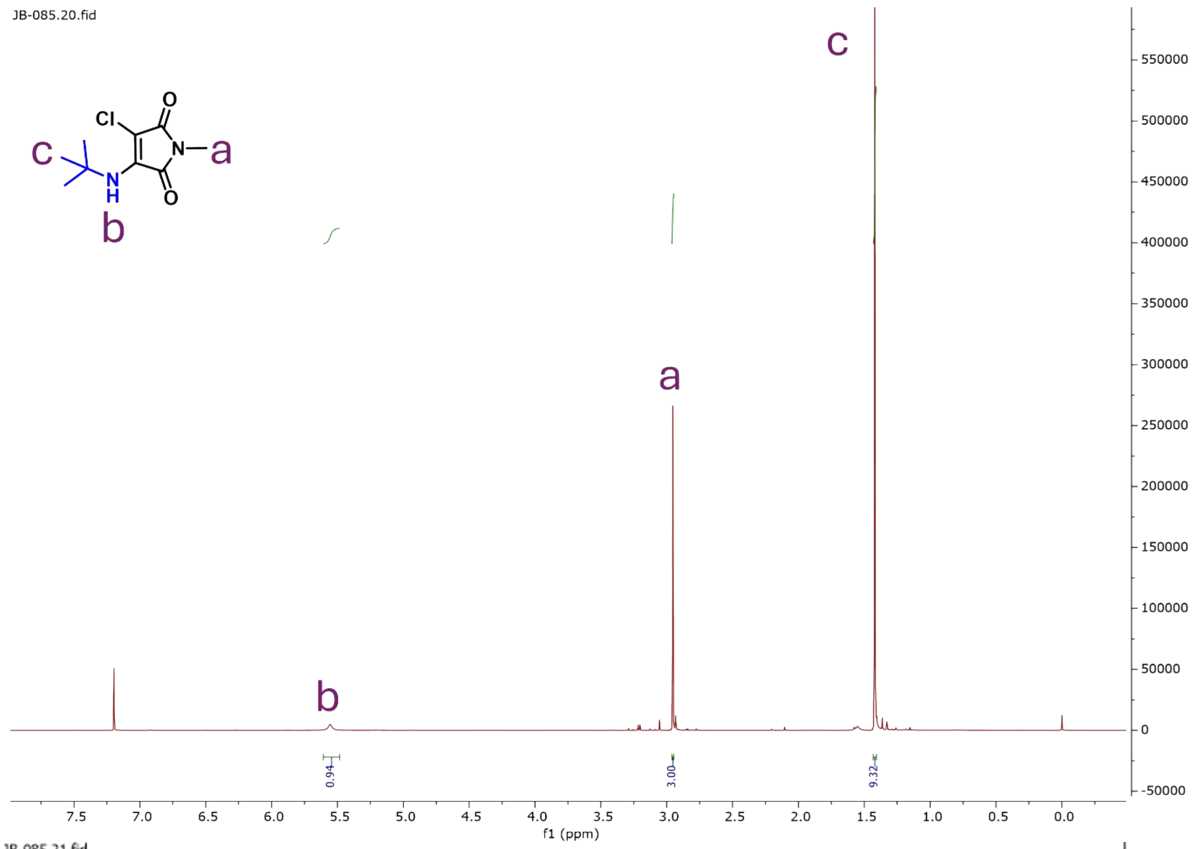


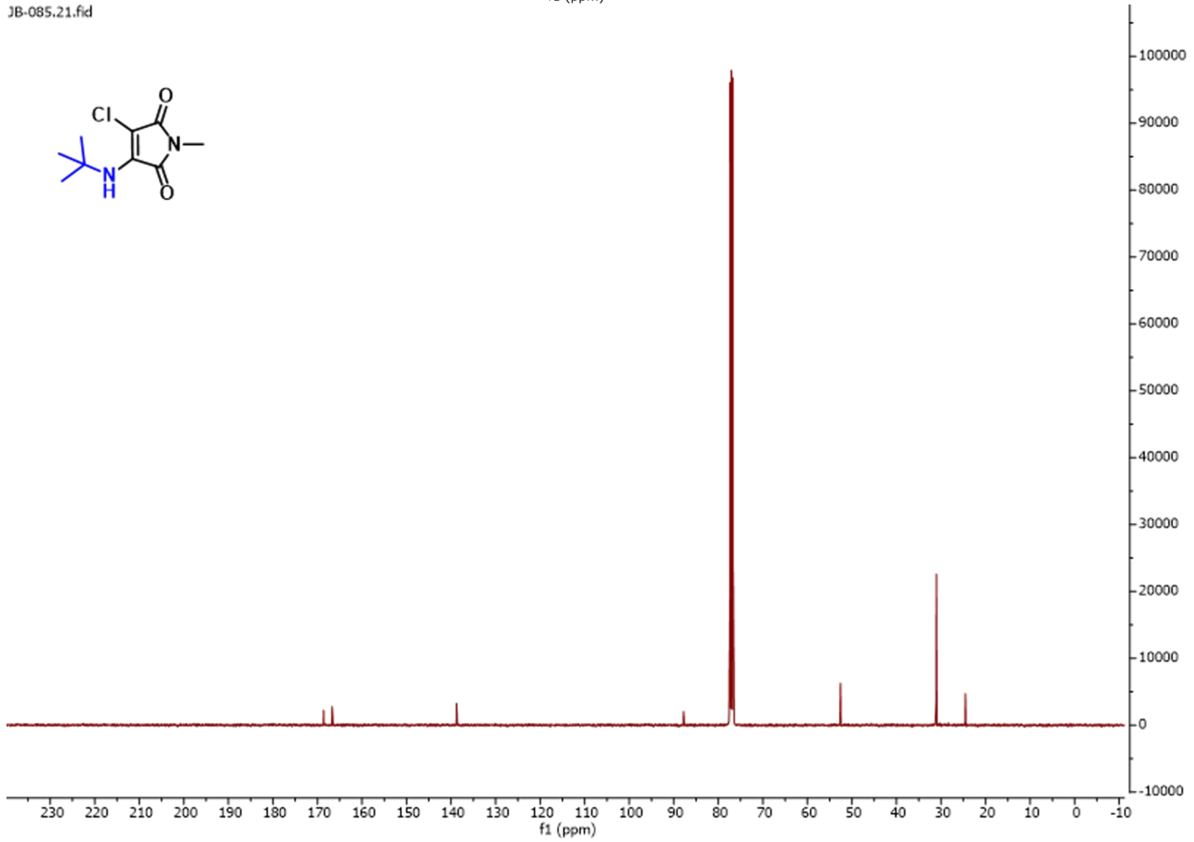


*Figure 10: $^{1}H$ and $^{13}C$ NMR Spectra of 1d*

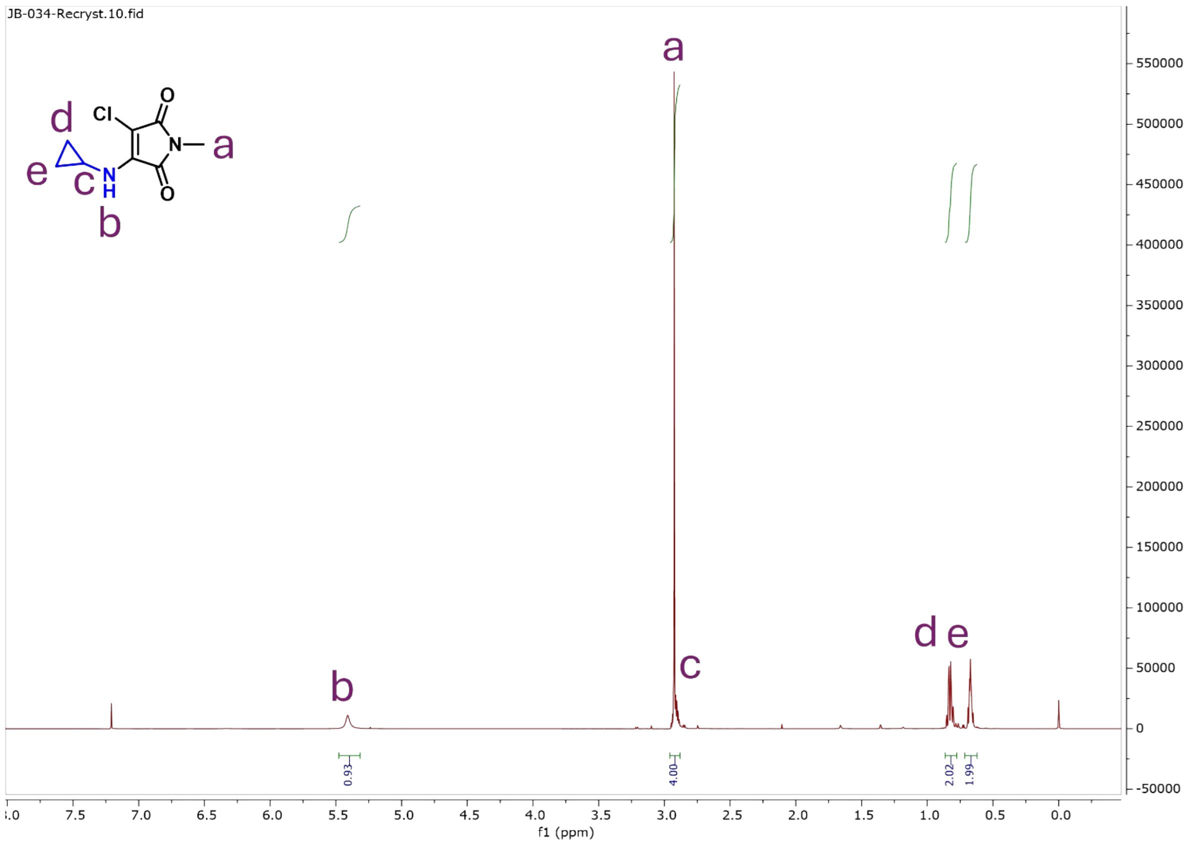


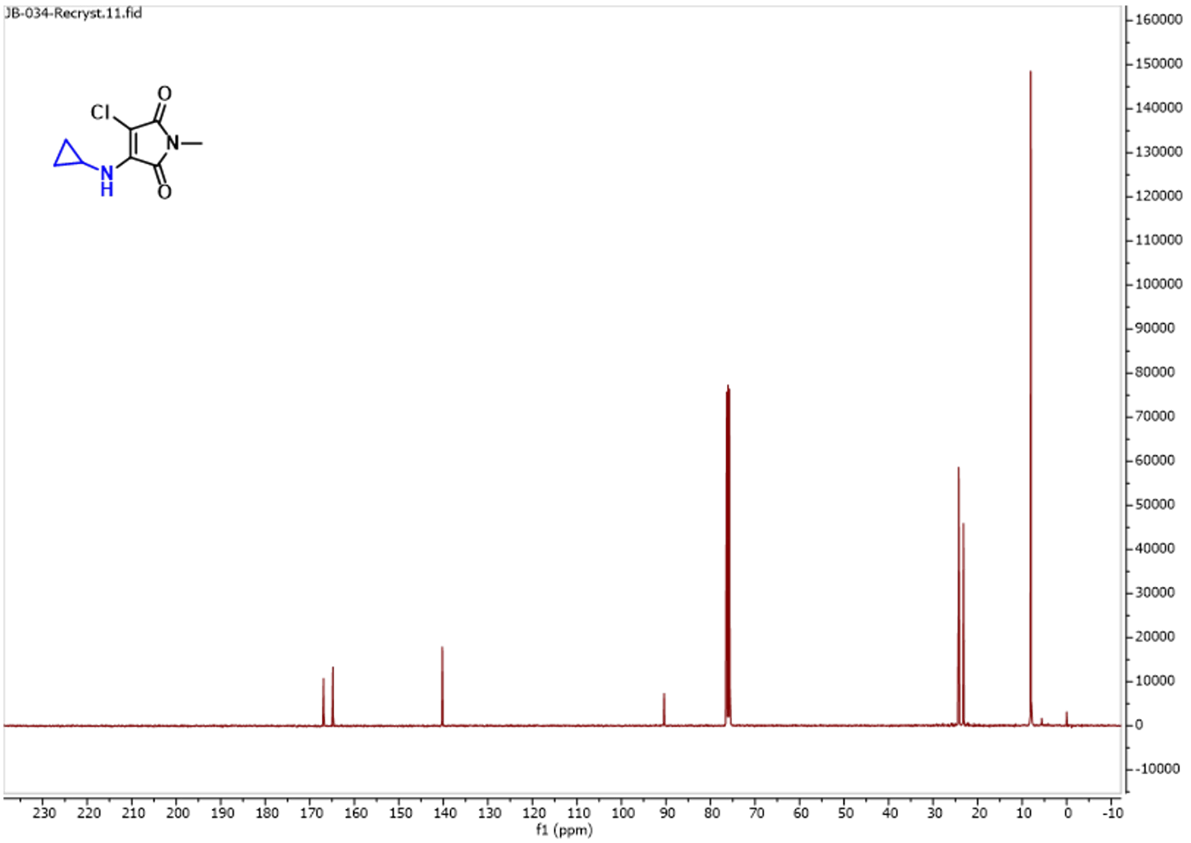


*Figure 11: $^{1}$H and $^{13}$C NMR Spectra of 1e*

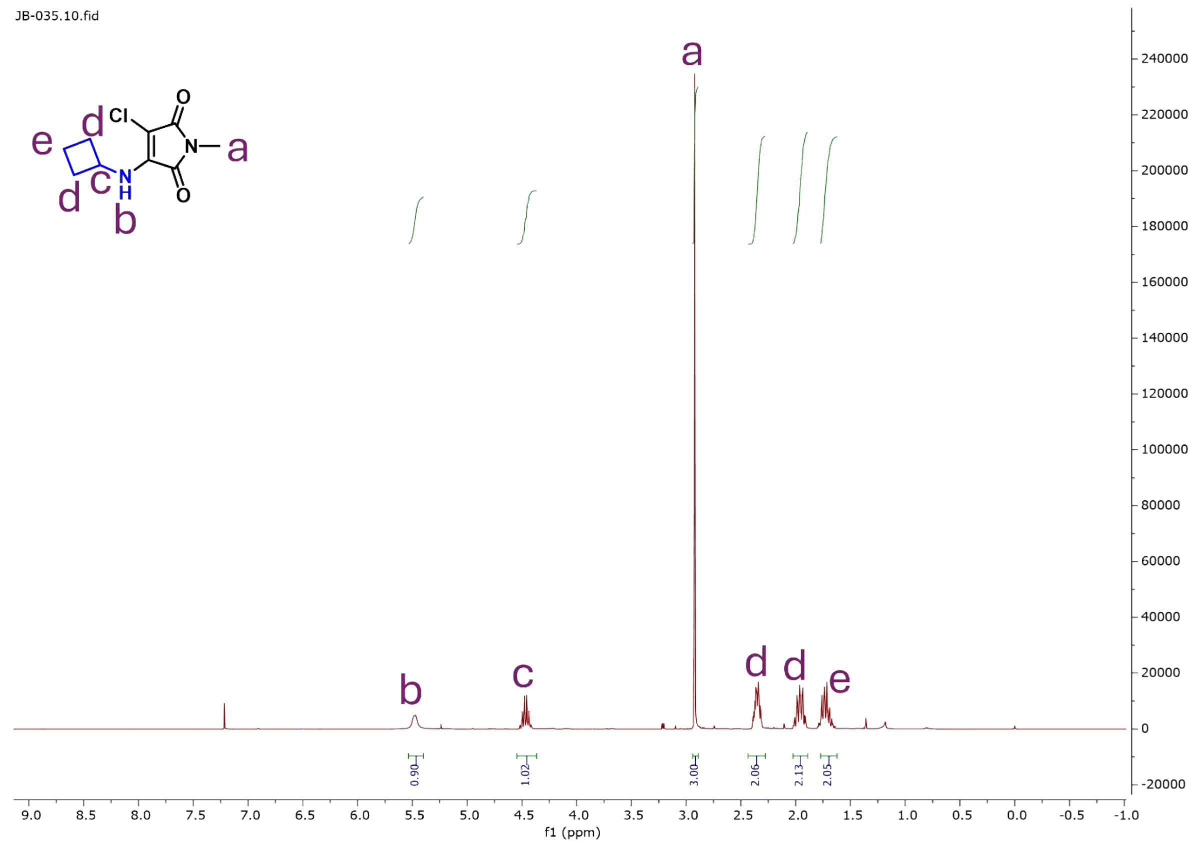


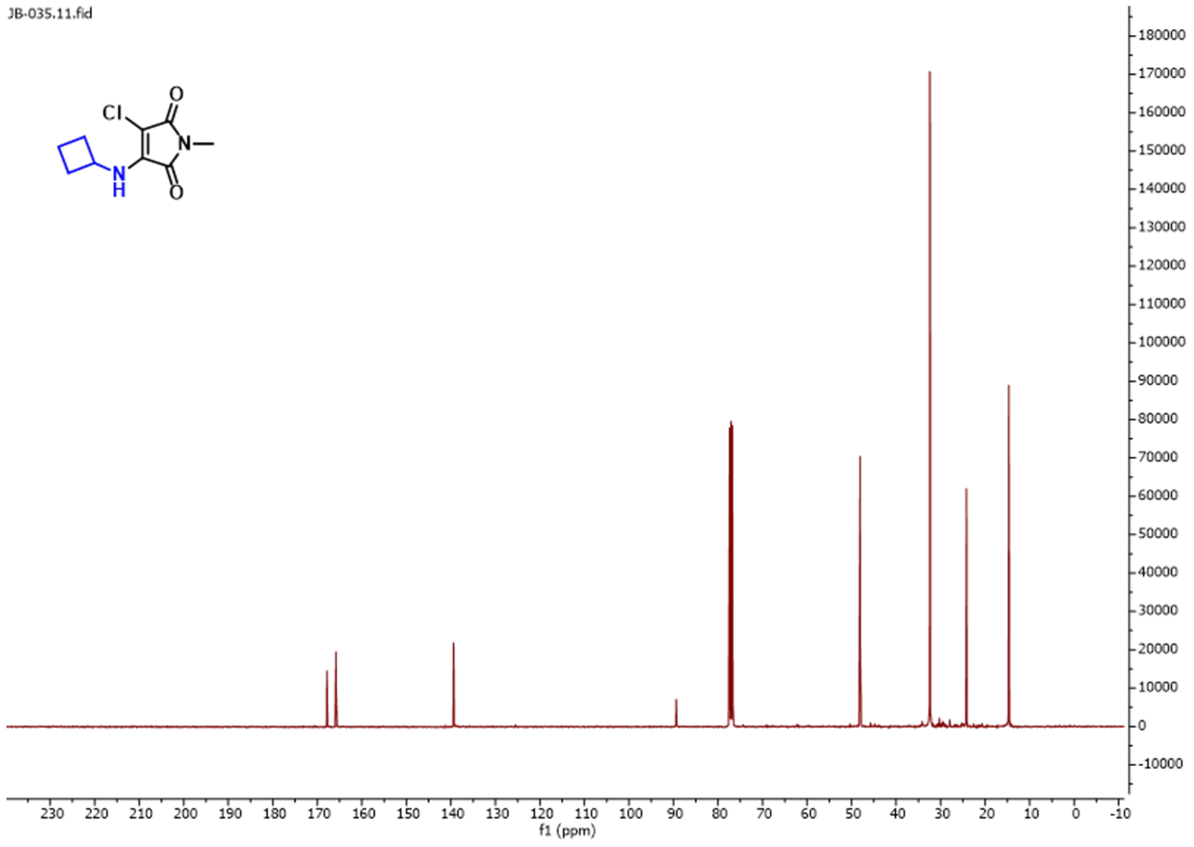


*Figure 12: ¹H and ¹³C NMR Spectra of 1f*

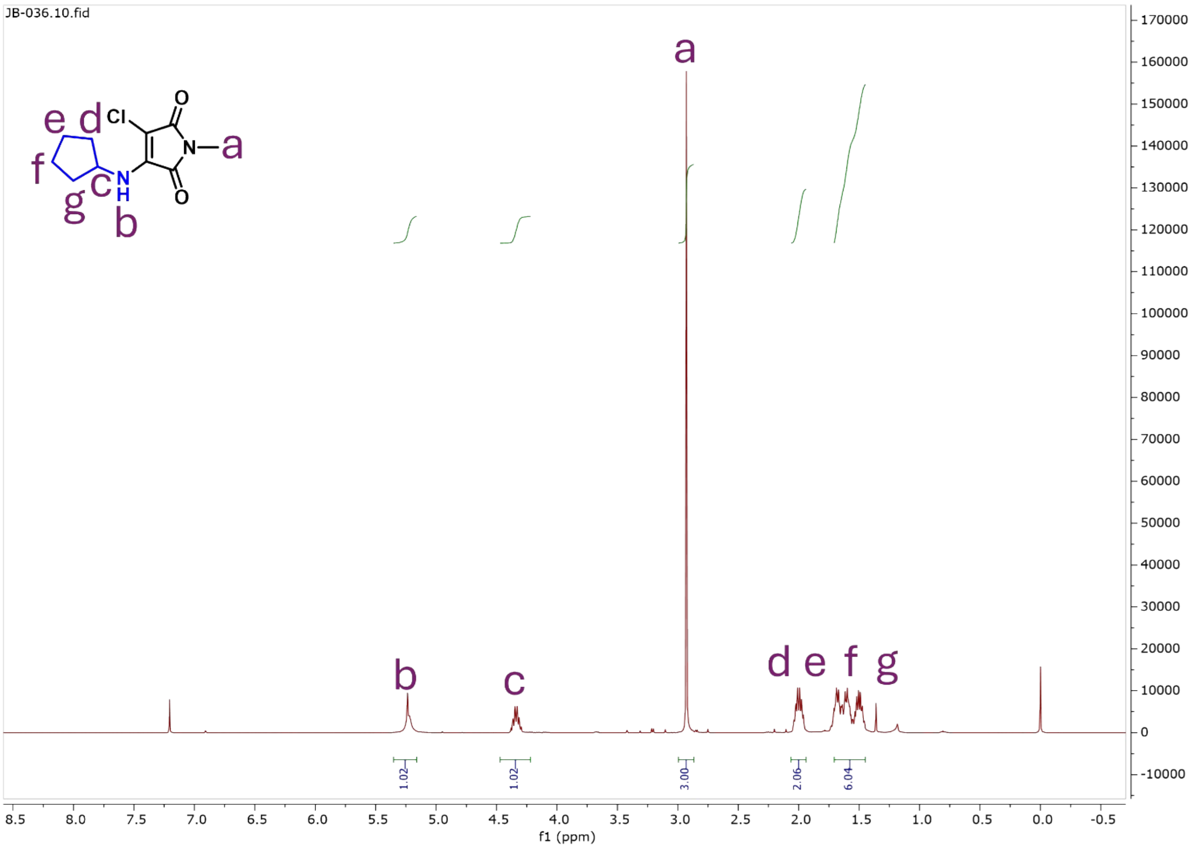


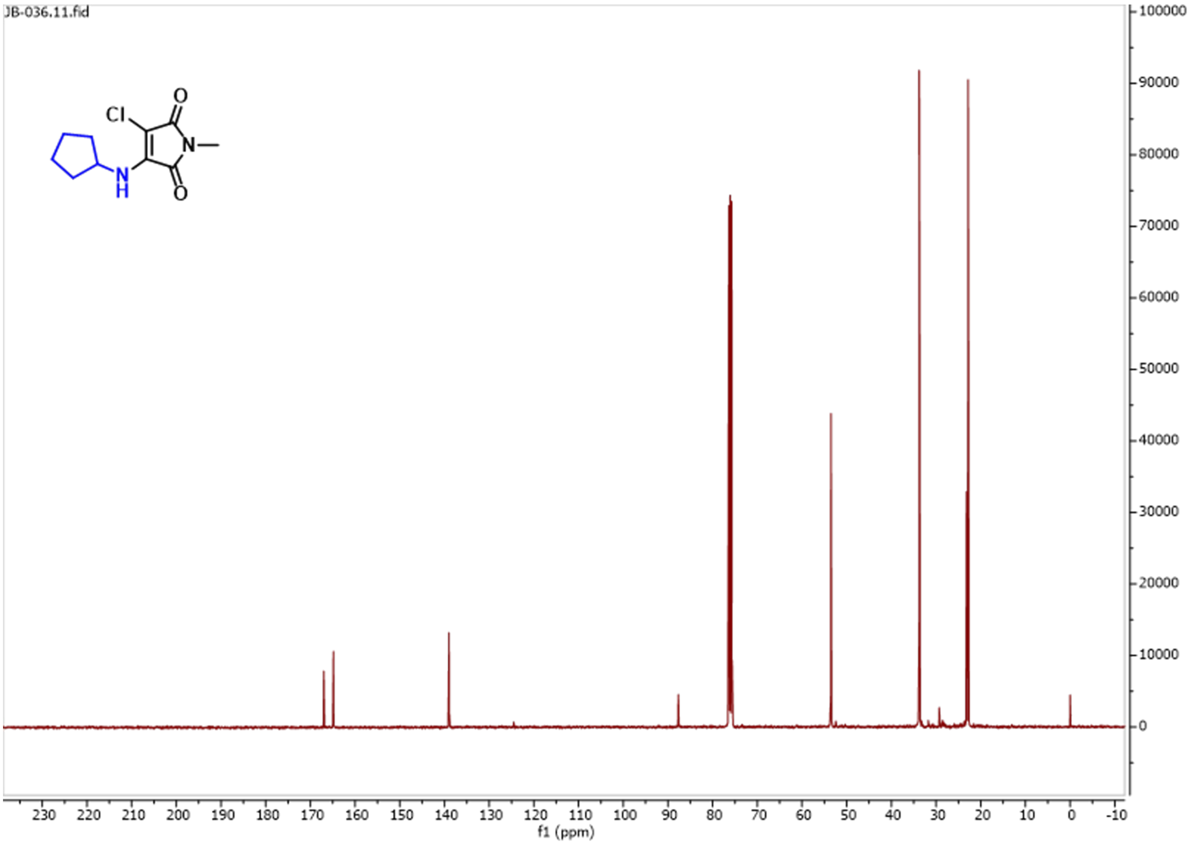


*Figure 13: ¹H and ¹³C NMR Spectra of 1g*

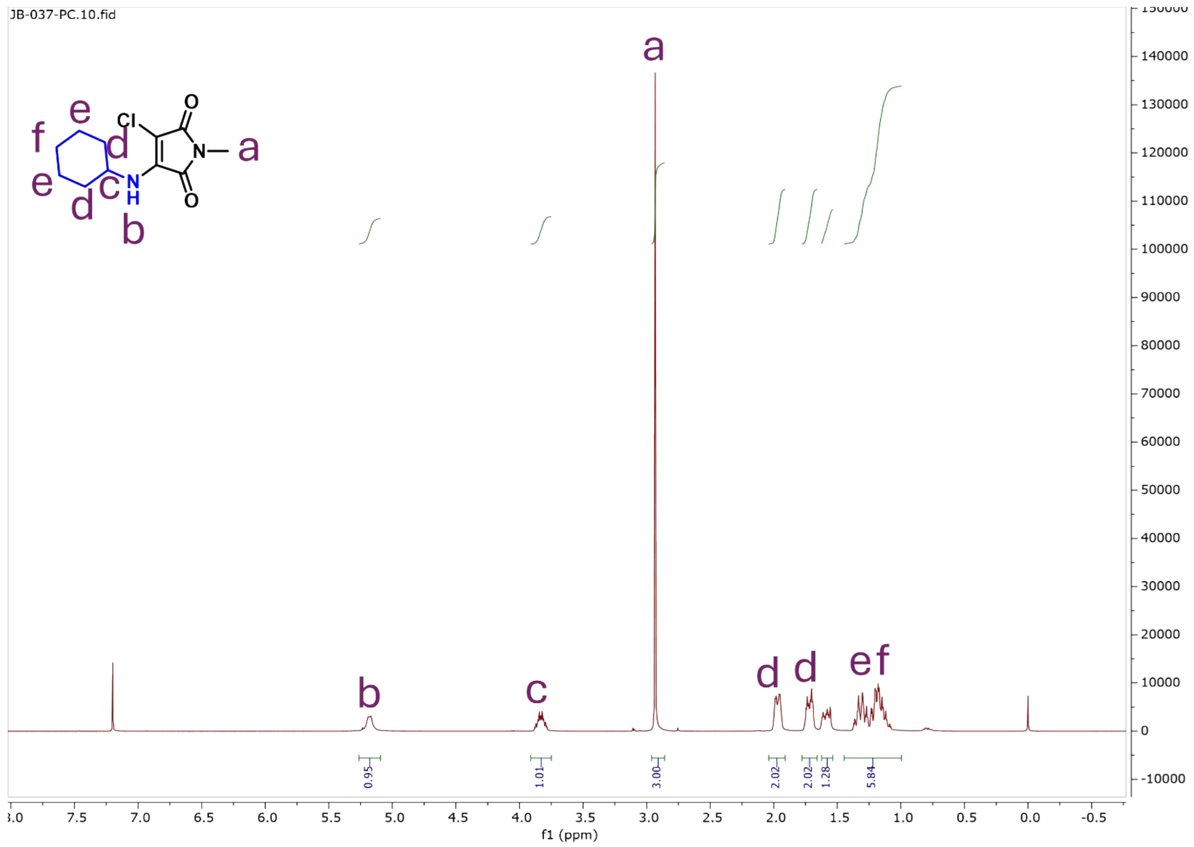


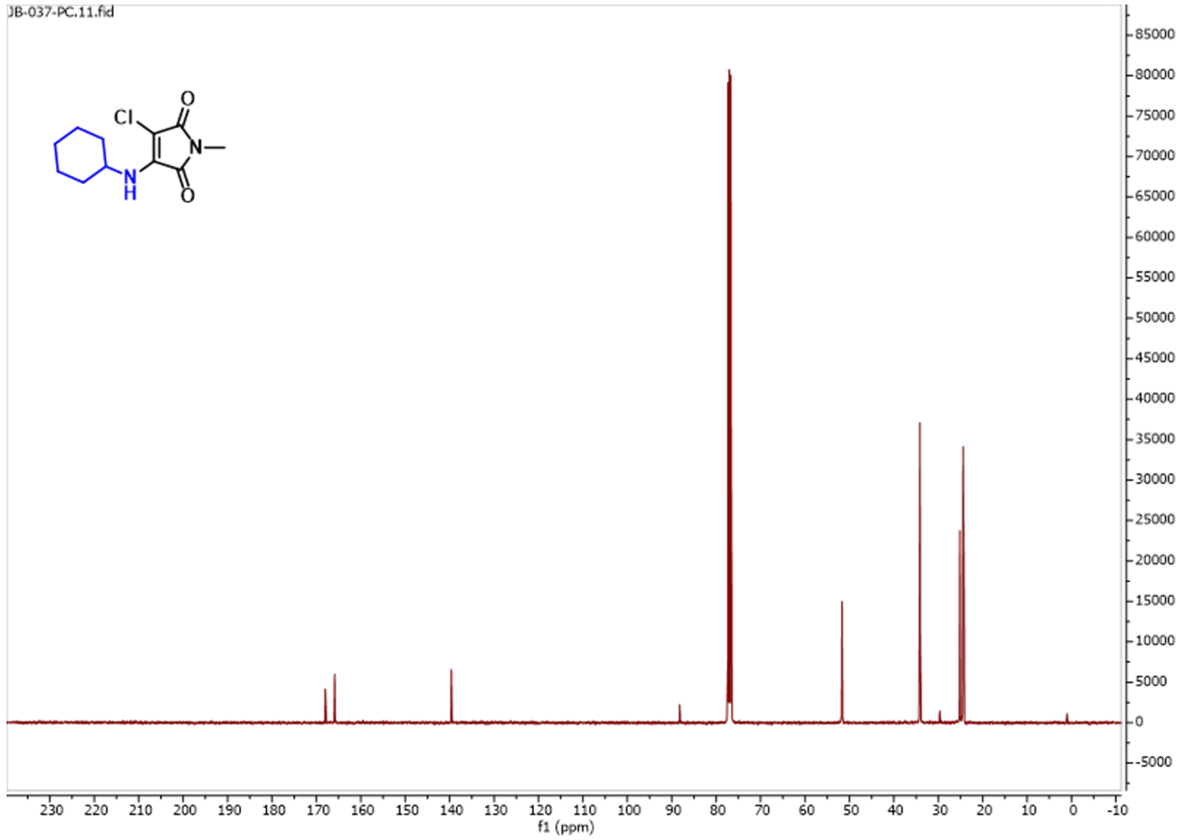


*Figure 14: $^{1}H$ and $^{13}C$ NMR Spectra of 1h*

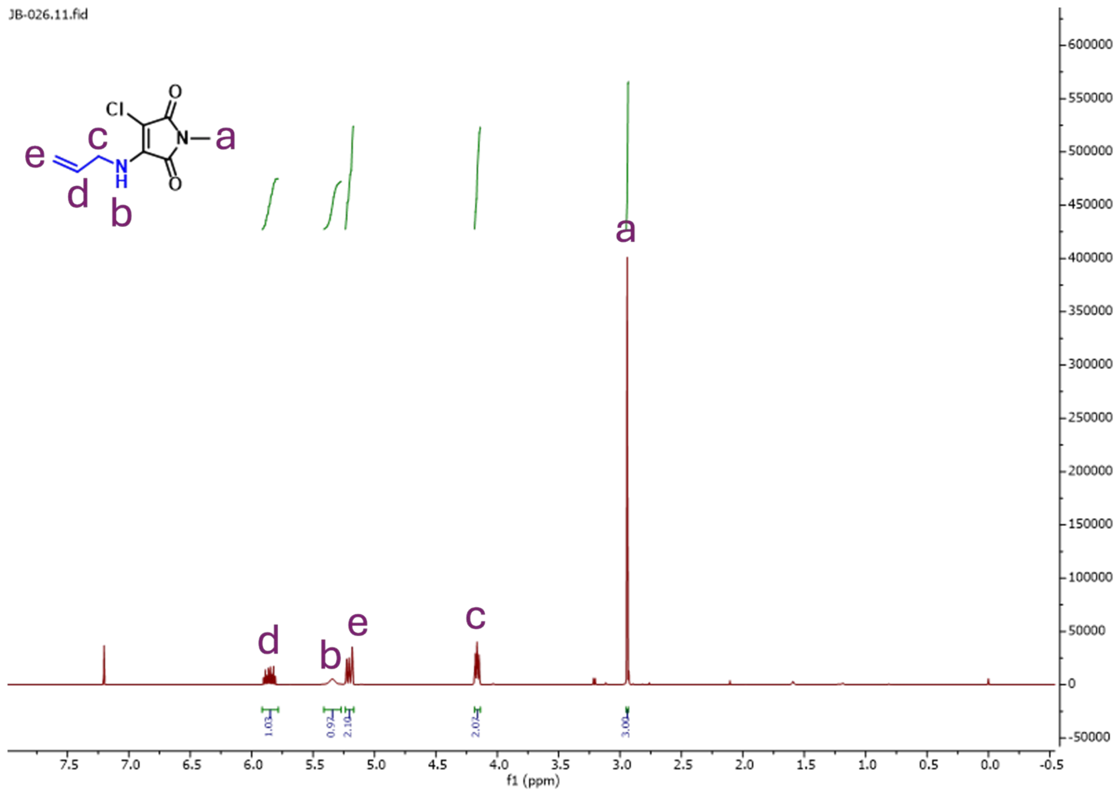


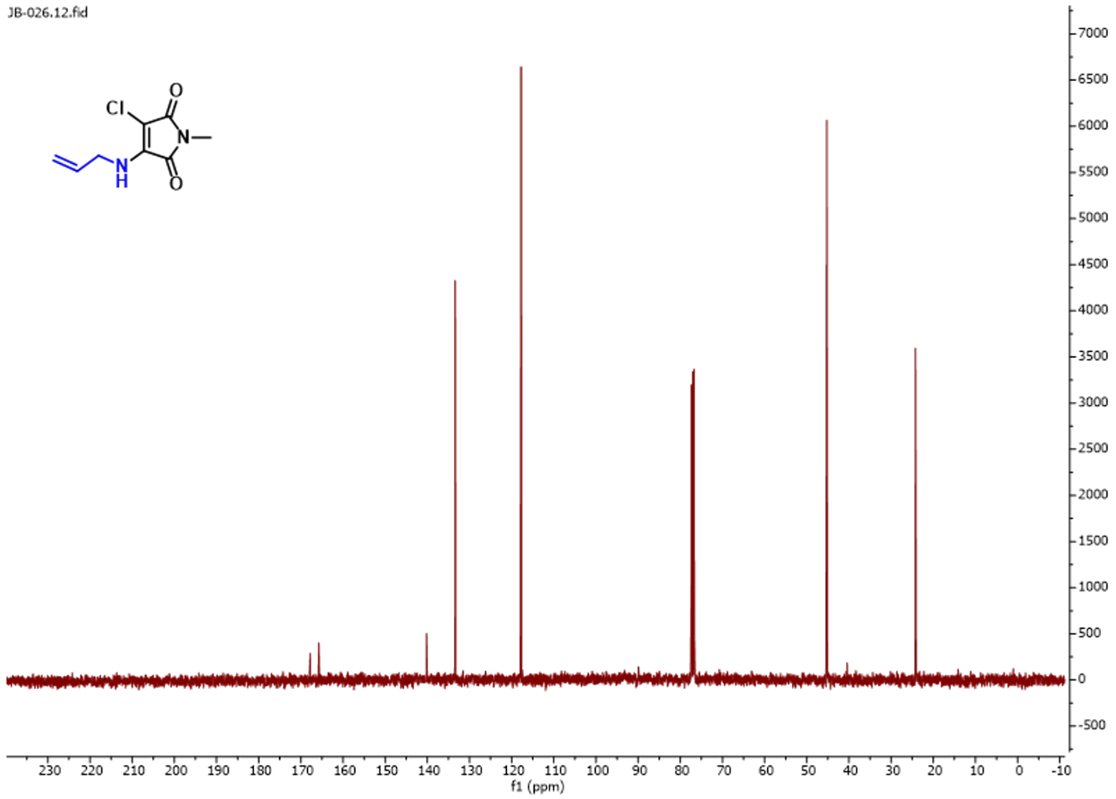


*Figure 15: $^{1}$H and $^{13}$C NMR Spectra of 1i*

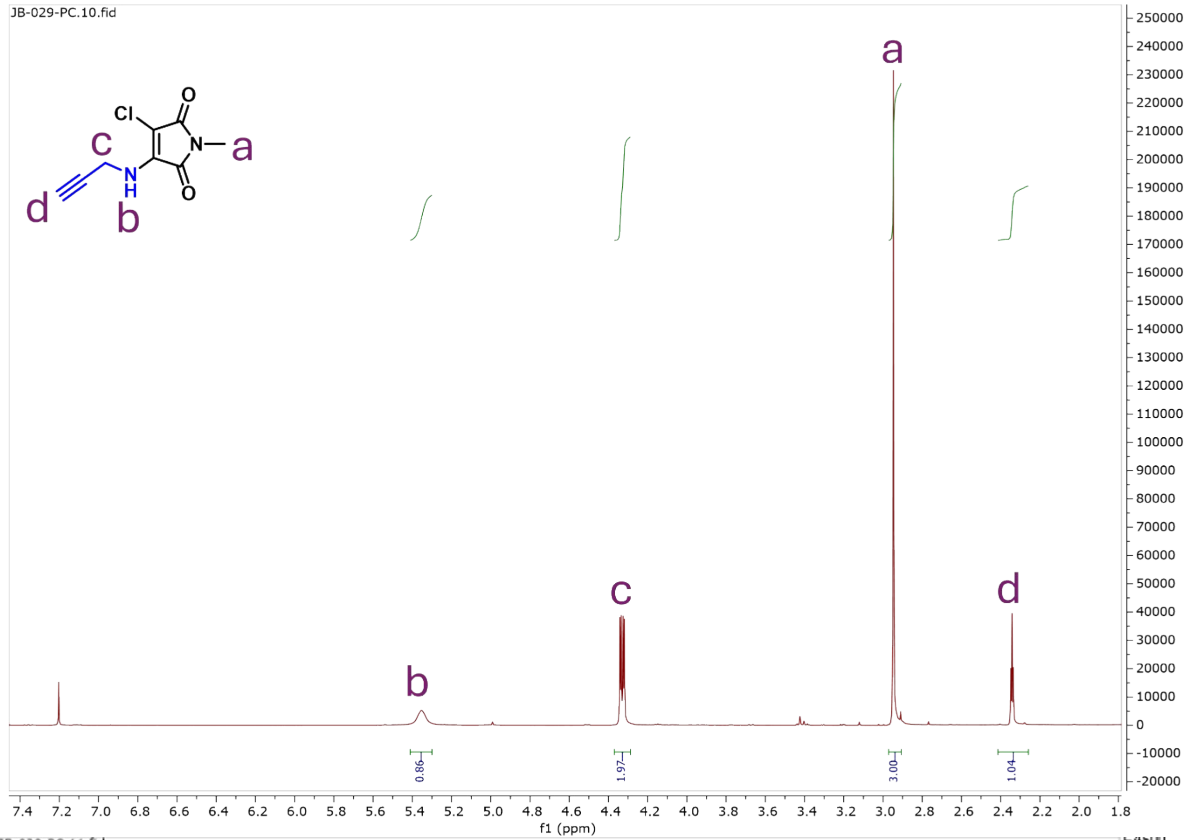


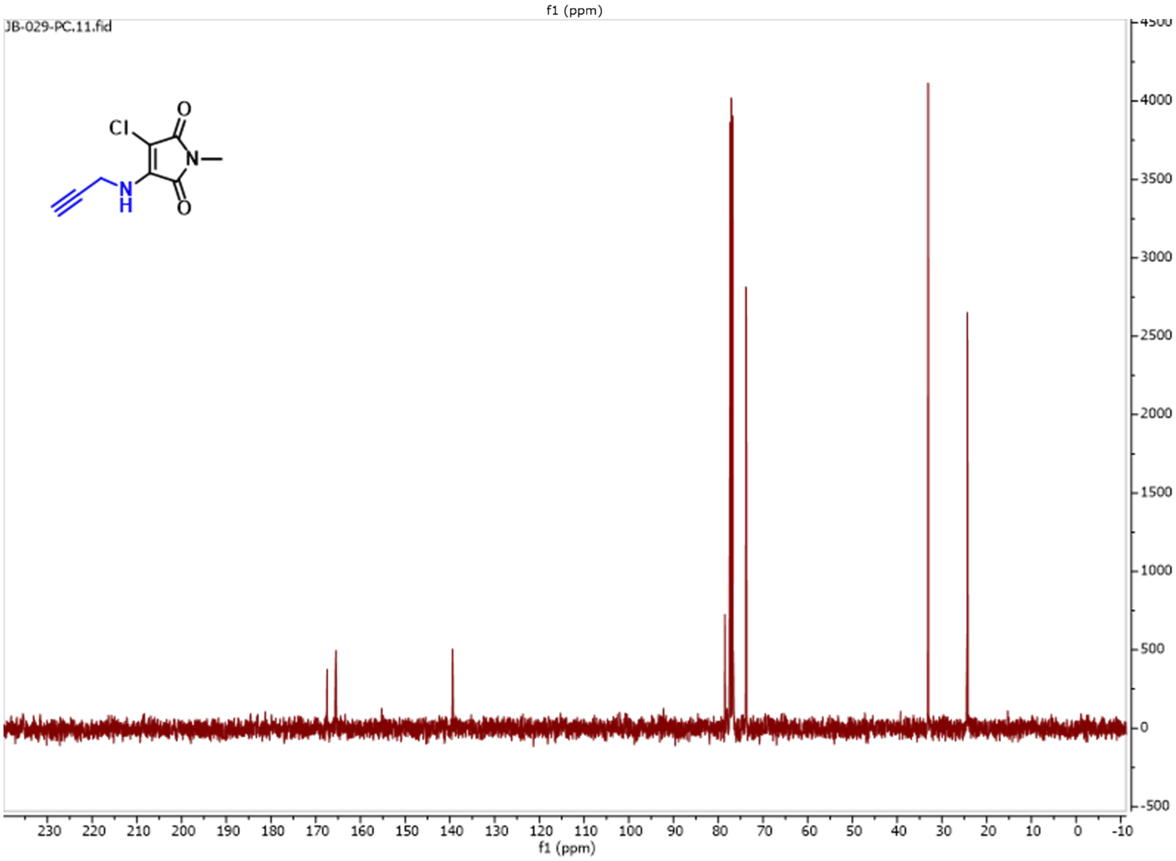


*Figure 16: $^{1}$H and $^{13}$C NMR Spectra of 1j*

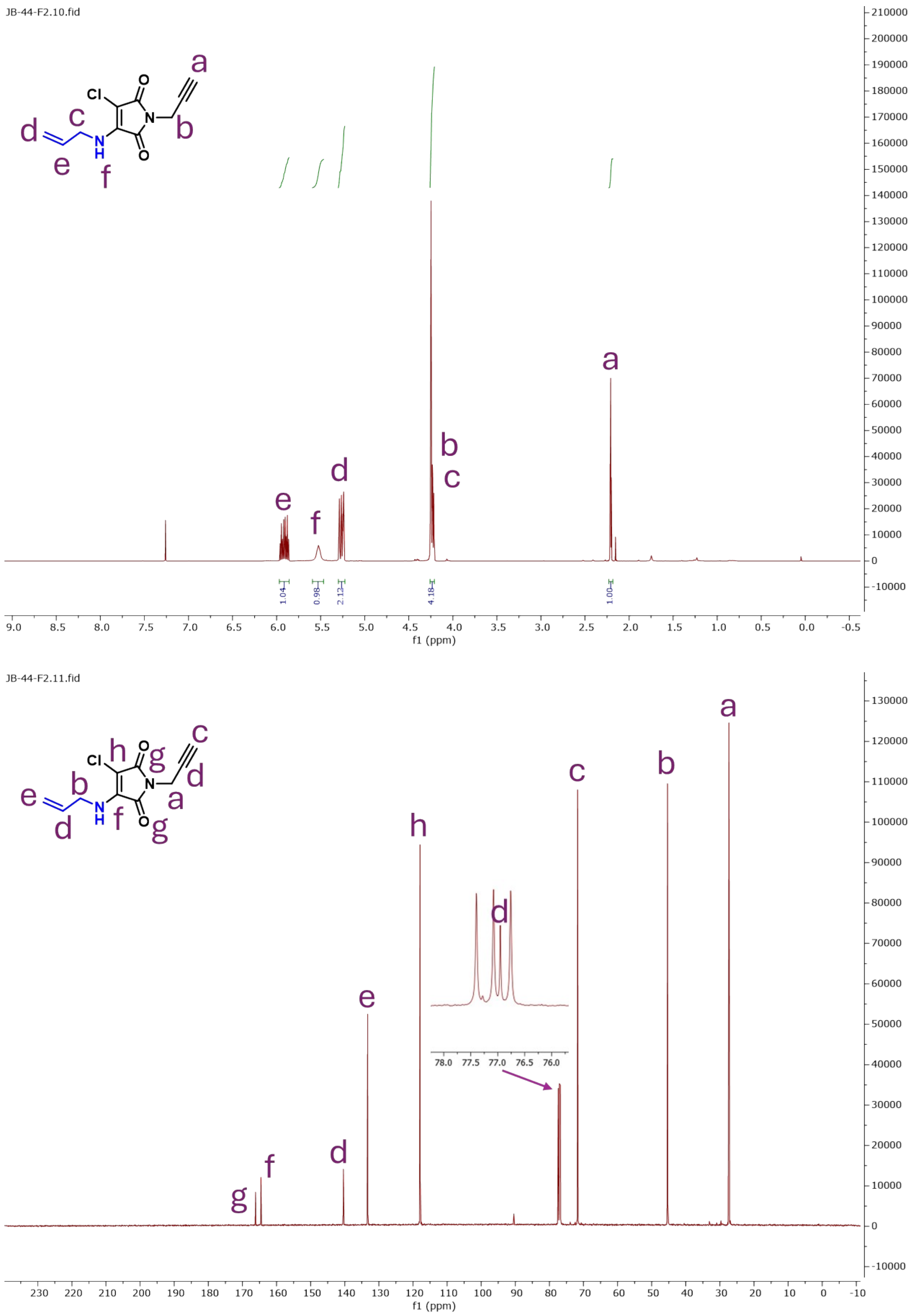


*Figure 17: $^{1}H$ and $^{13}C$ NMR Spectra of 1k*

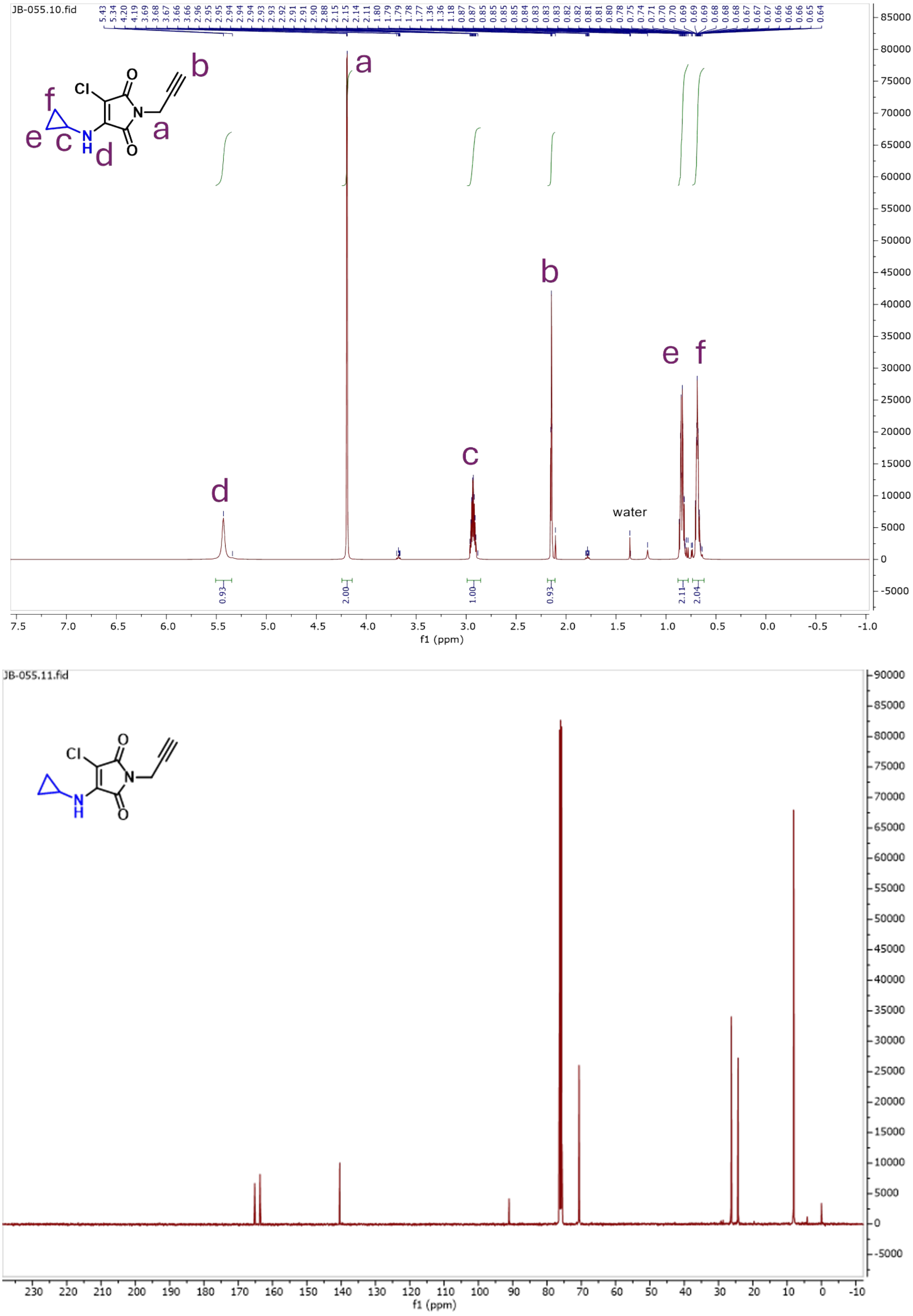


*Figure 18: ¹H and ¹³C NMR Spectra of 1l*

# UV-visible Spectra

UV-visible absorbance spectra were recorded using samples of $1x10^{-4}$M concentration in various solvents. A stock solution was used to dispense a quantity, dried, and then diluted with the appropriate solvent to give the final 2 mL sample for UV-visible measurement. The measurements were performed using a 1 cm quartz cuvette in an Agilent Cary 3500 UV-vis spectrophotometer and supplied software. A blank of each solvent (primarily diethyl ether) was run in each case before sample measurement. The wavelengths and absorbances were recorded directly from the Agilent Cary UV-vis software.

## AM UV-visible spectra

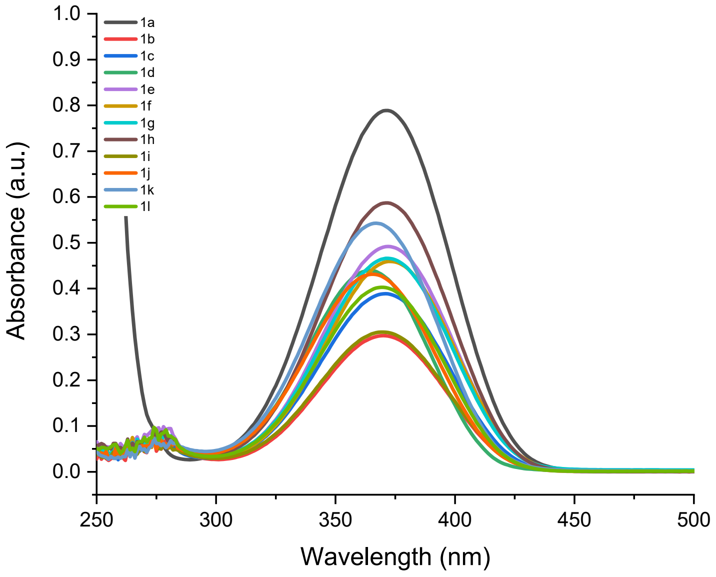


*Figure 19: Stacked UV-visible spectra of AMs in diethyl ether*

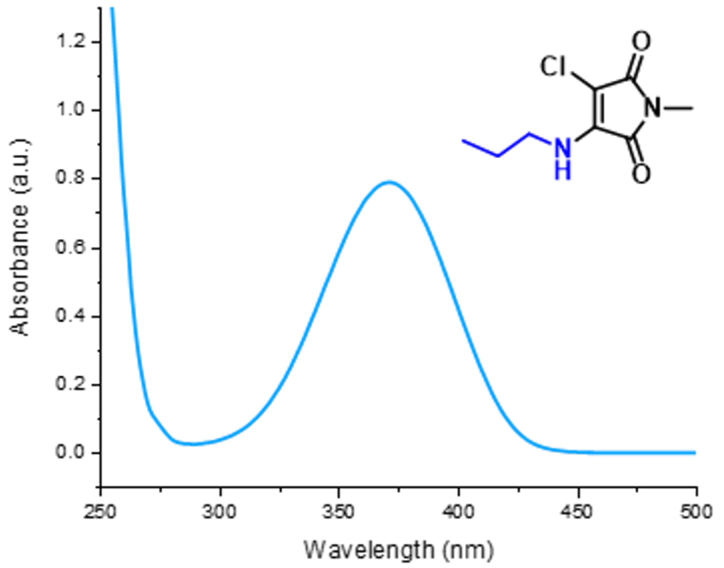


*Figure 20: UV-visible spectrum of 1a in diethyl ether, $\lambda_{max}$ = 372 nm*

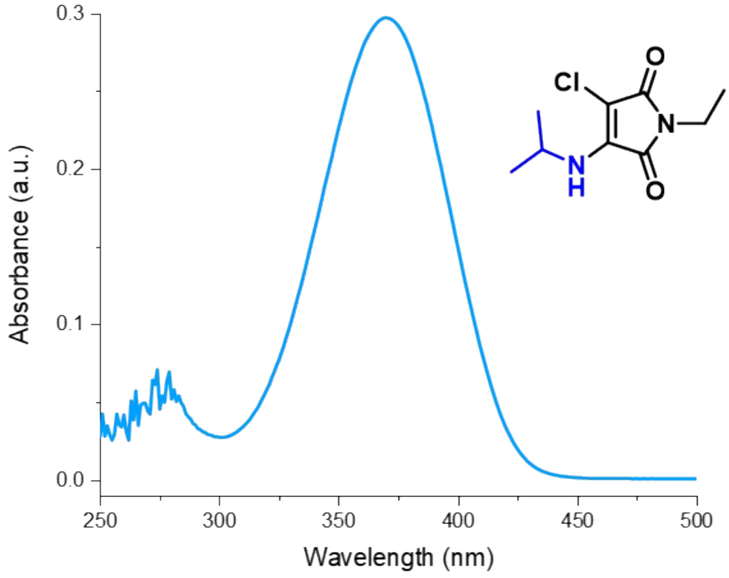


*Figure 21: UV-visible spectrum of 1b in diethyl ether, $\lambda_{max}$ = 370 nm*

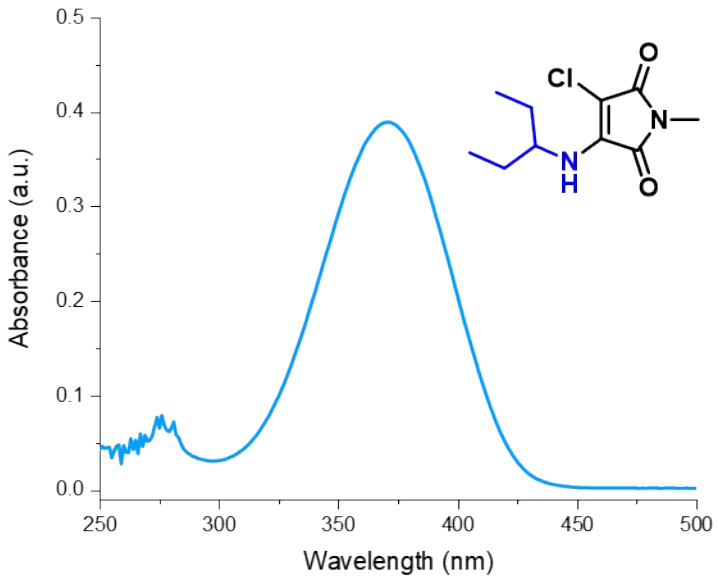


*Figure 22: UV-visible spectrum of 1c in diethyl ether, $\lambda_{max}$ = 371 nm*

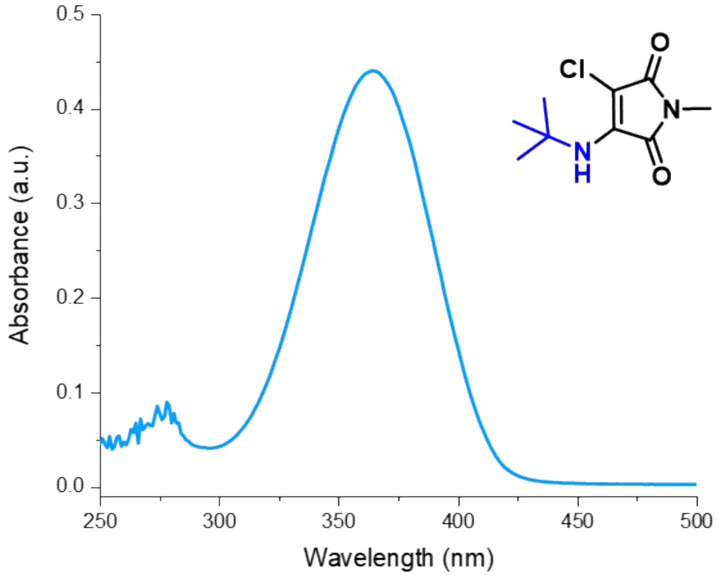


*Figure 23: UV-visible spectrum of 1d in diethyl ether, $\lambda_{max}$ = 364 nm*

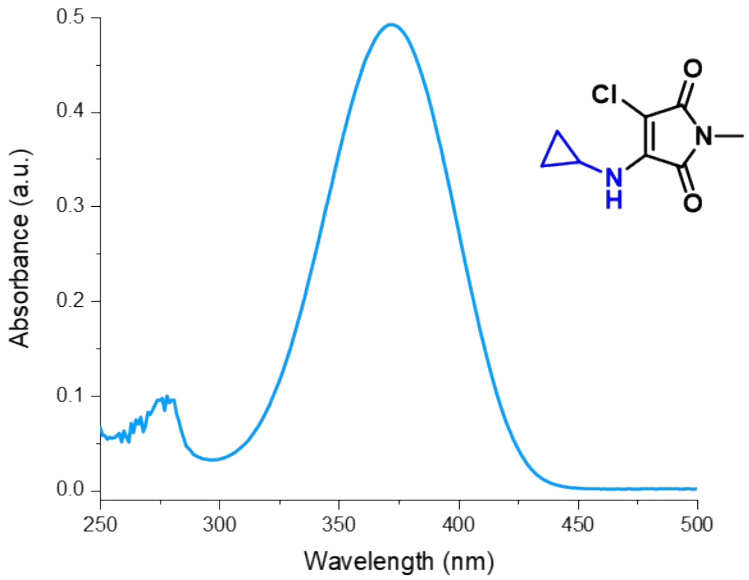


*Figure 24: UV-visible spectrum of 1e in diethyl ether, $\lambda_{max}$ = 374 nm*

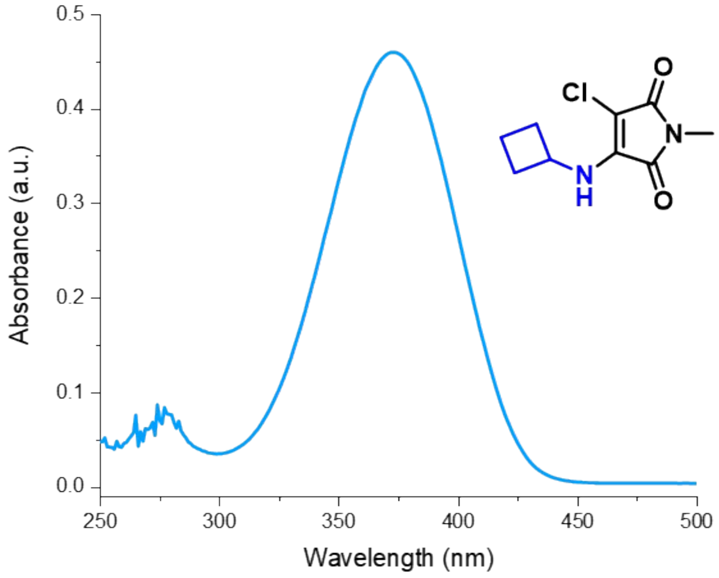


*Figure 25: UV-visible spectrum of 1f in diethyl ether, $\lambda_{max}$ = 373 nm*

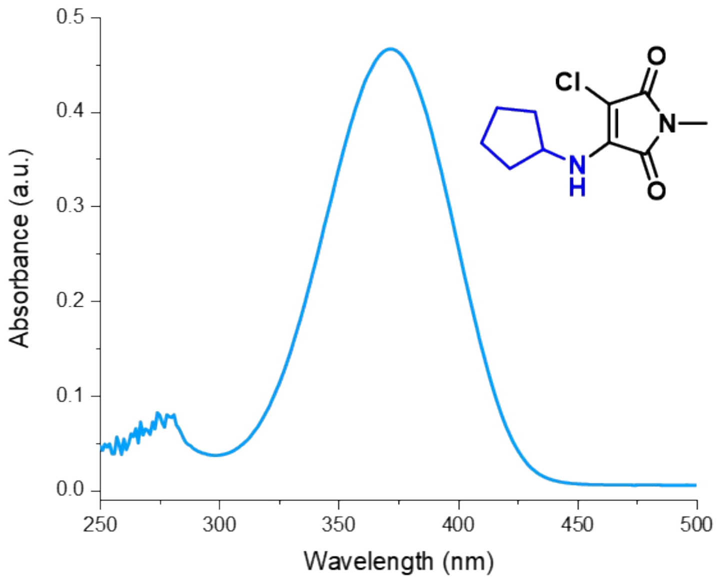


*Figure 26: UV-visible spectrum of 1g in diethyl ether, $\lambda_{max}$ = 372 nm*

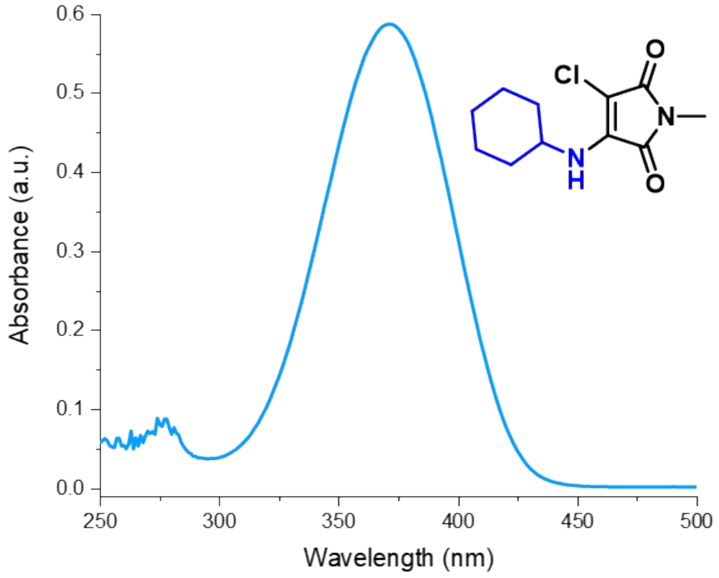


*Figure 27: UV-visible spectrum of 1h in diethyl ether, $\lambda_{max}$ = 371 nm*

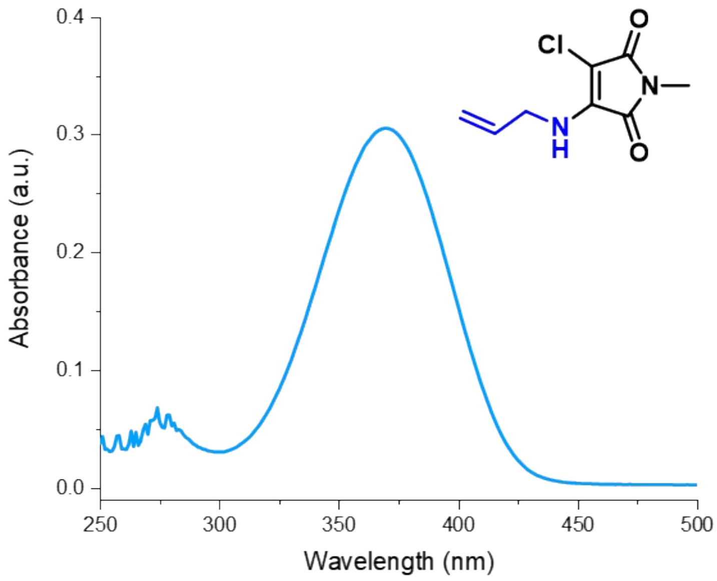


*Figure 28: UV-visible spectrum of 1i in diethyl ether, $\lambda_{max}$ = 370 nm*

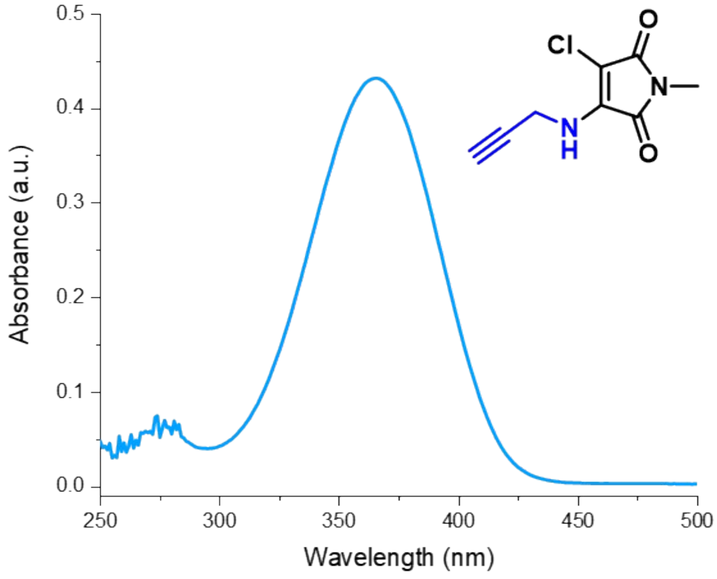


*Figure 29: UV-visible spectrum of 1j in diethyl ether, $\lambda_{max}$ = 364 nm*

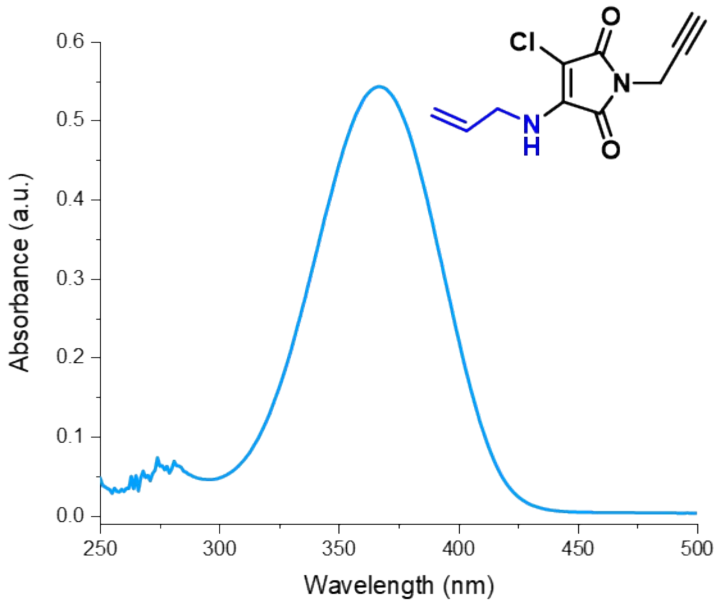


*Figure 30: UV-visible spectrum of 1k in diethyl ether, $\lambda_{max}$ = 367 nm*

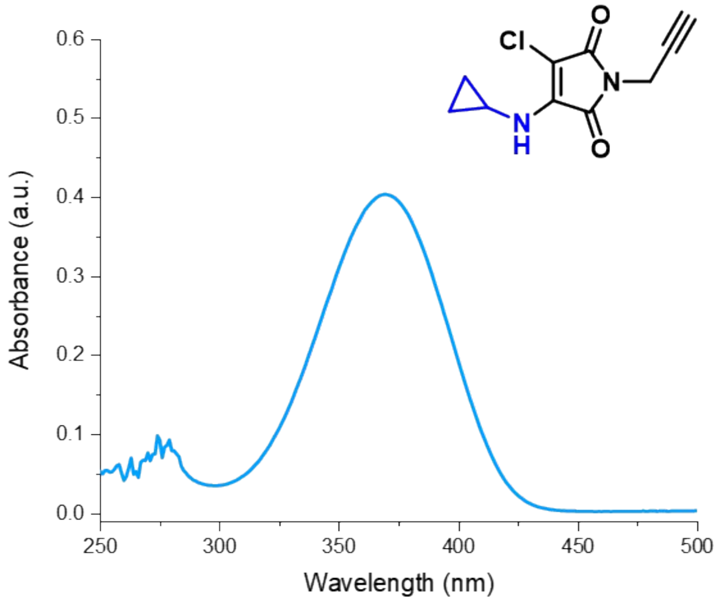


*Figure 31: UV-visible spectrum of 1k in diethyl ether, $\lambda_{max}$ = 369 nm*

| Molecule | λabs $_{max}$ (nm) | Absorbance (a.u.) | Extinction Coefficient ($M^{-1}$ $cm^{-1}$) |
|---|---|---|---|
| **1a** | 371 | 0788 | 3154 |
| **1b** | 370 | 0.297 | 2970 |
| **1c** | 371 | 0.389 | 3890 |
| **1d** | 364 | 0.440 | 4397 |
| **1e** | 373 | 0.492 | 4920 |
| **1f** | 373 | 0.459 | 4590 |
| **1g** | 372 | 0.466 | 4660 |
| **1h** | 371 | 0.587 | 5870 |
| **1i** | 370 | 0.305 | 3050 |
| **1j** | 364 | 0.434 | 4340 |
| **1k** | 367 | 0.543 | 5430 |
| **1l** | 369 | 0.403 | 4030 |

*Table 2: AM UV-visible characterisation summary (Samples measured at appropriate concentrations in diethyl ether)*

## AM Solvatochromism

Several AM derivatives were selected to assess their solvatochromic properties, and subsequently the efficacy of current *in-silico* predictive models. Here we present the absorbance spectra of these molecules, measured at varying concentrations suitable for determining the maximum $\lambda_{abs}$ value. These values were obtained using the same equipment and methods as detailed above.

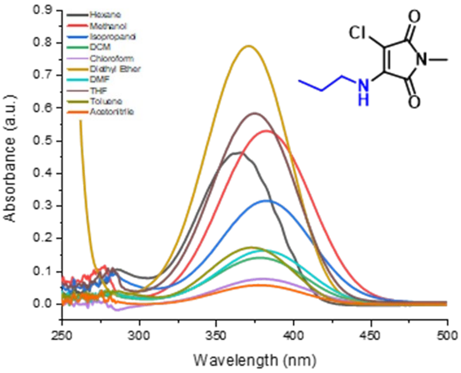


*Figure 32: Stacked UV-visible spectra of 1a*

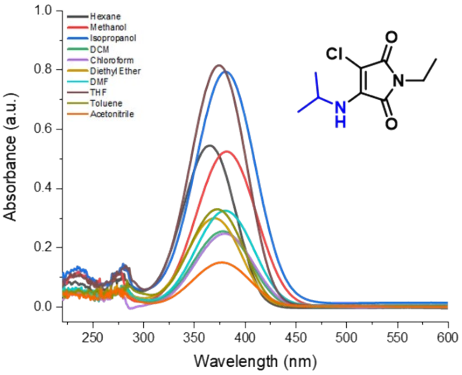


*Figure 33: Stacked UV-visible spectra of 1b*

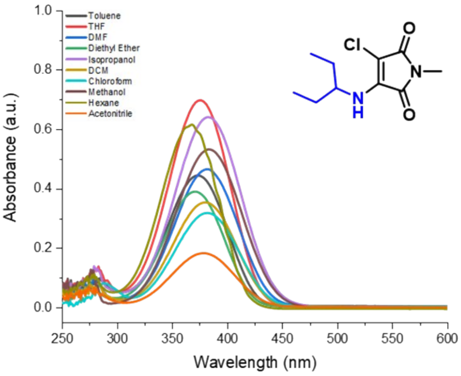


*Figure 34: Stacked UV-visible spectra of 1c*

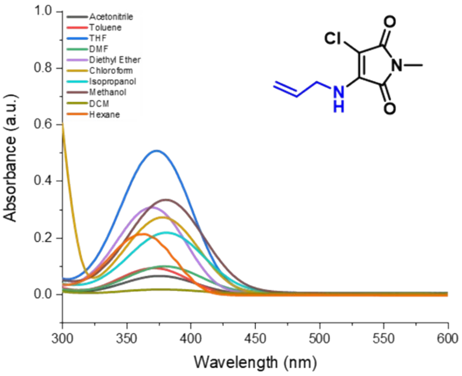


*Figure 35: Stacked UV-visible spectra of 1i*

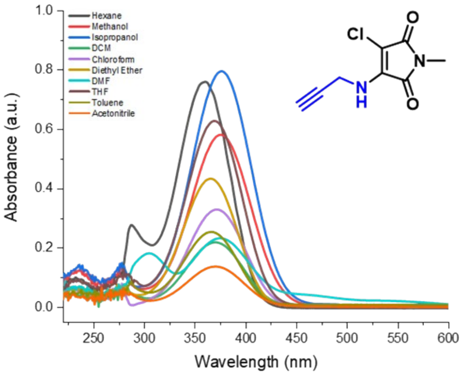


*Figure 36: Stacked UV-visible spectra of 1j*

## AM Solvatochromism- TD-DFT Predicted Spectra

When performing TD-DFT of AMs using the CPCM solvation model, it is possible to plot the predicted UV-visible spectrum of the calculation, with the parameters of the spectrum set to the range of 10000 $cm^{-1}$ to 30000 $cm^{-1}$ with 1000 $cm^{-1}$ broadening using the command:

**orca_mapspc** jobname.out ABS -x010000 -x130000 -w1000

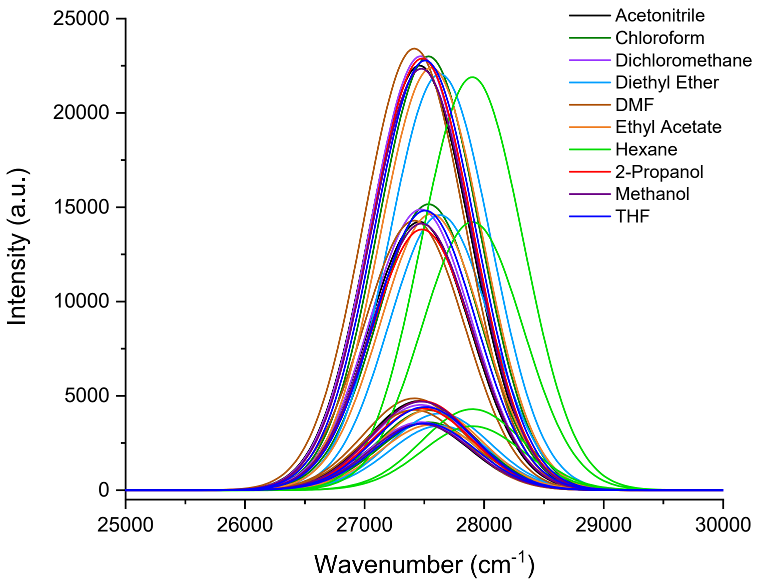


*Figure 37: 1a TD-DFT Simulated UV-visible absorbance spectra (in vacuo)*

This command uses the TD-DFT output file, and the calculated transitions to plot the corresponding absorbance wavenumber, along with the relative intensity (a.u.). Each peak within a solvent class appears to have the same wavenumber, and thus wavelength.

Converting the wavenumber to wavelength allows for a comparison between the experimental and predicted spectra. While there does not appear to be a large difference in wavenumber ($cm^{-1}$), the difference is much more noticeable when converted to wavelength (nm). The following spectra are for molecule **AM 1a.**

## Acetonitrile

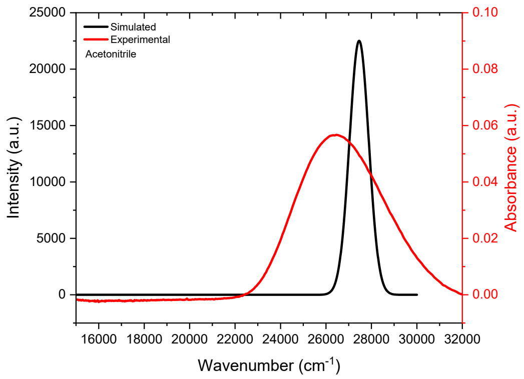


## Chloroform

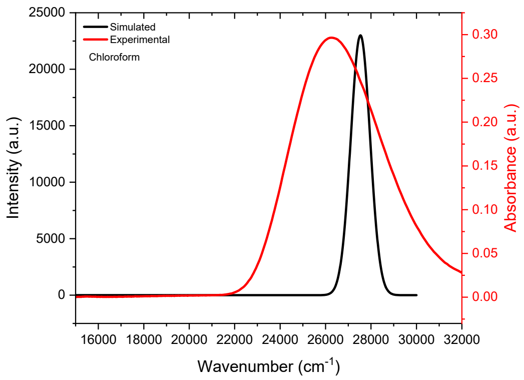


## Dichloromethane

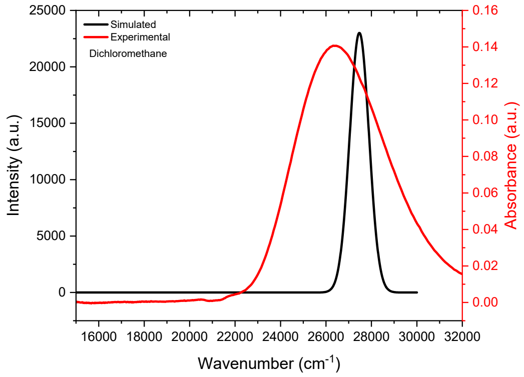


## Diethyl Ether

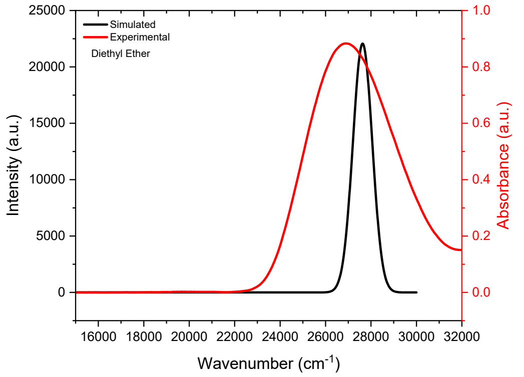


## Ethyl Acetate

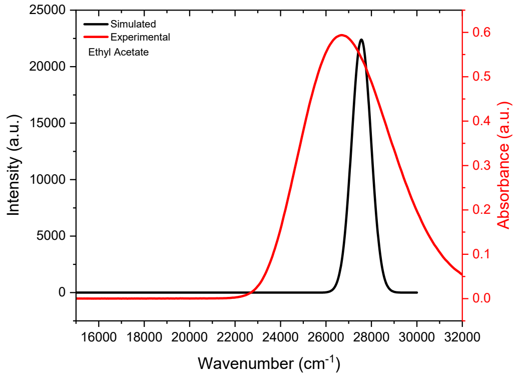


## Hexane

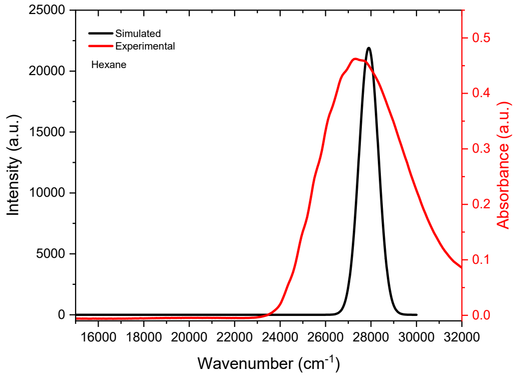

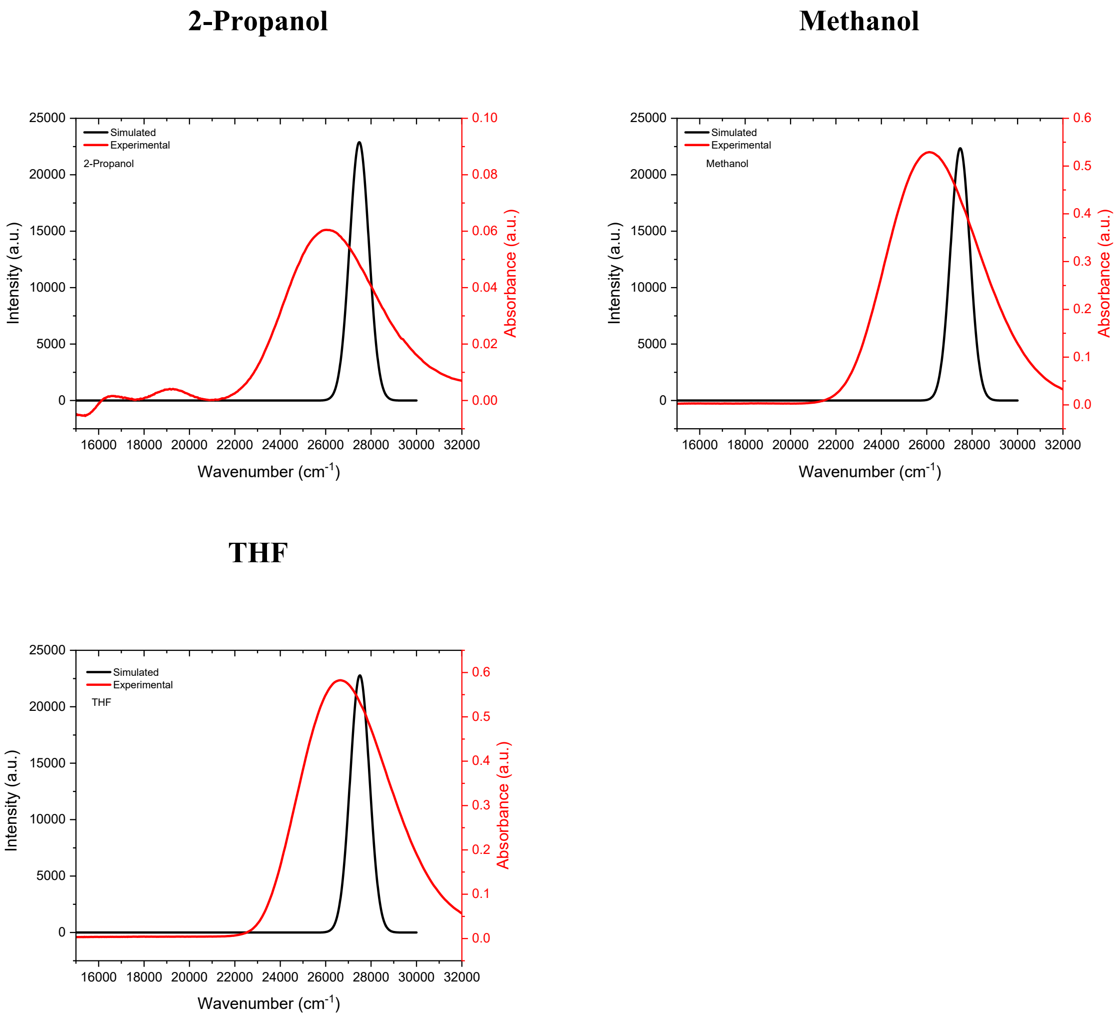


*Figure 38: TD-DFT Predicted and experimental UV-visible spectra of 1a in various solvents (adjusted for wavenumber)*

# Fluorescence Analysis

The following fluorescence spectra were recorded using an Edinburgh FS5 spectrofluorometer, with a xenon light source. Sample concentrations were typically between $1x10^{-4}$ to $1x10^{-5}$M, where concentrations were adjusted as appropriate between classes of maleimide. The spectra were recorded and processed within the Fluoracle software supplied with the Edinburgh instrument. The excitation wavelength in each case was set to the $\lambda_{max}$ observed from the UV-visible spectra obtained using the Agilent Cary software.

# AM Fluorescence Spectra:

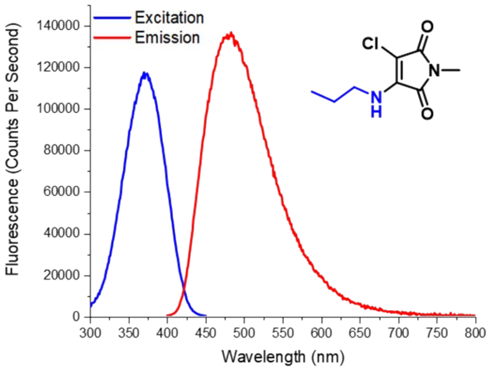


*Figure 39: Fluorescence Spectra of 1a in diethyl ether*

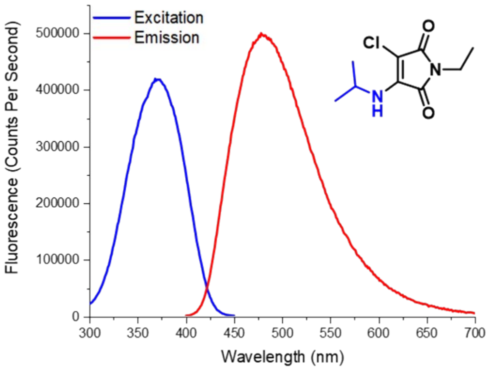


*Figure 40: Fluorescence Spectra of 1b in diethyl ether*

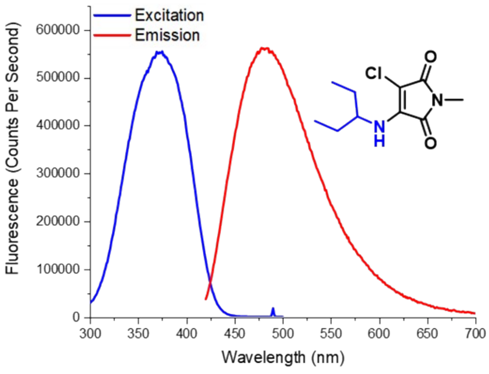


*Figure 41:Fluorescence Spectra of 1c in diethyl ether*

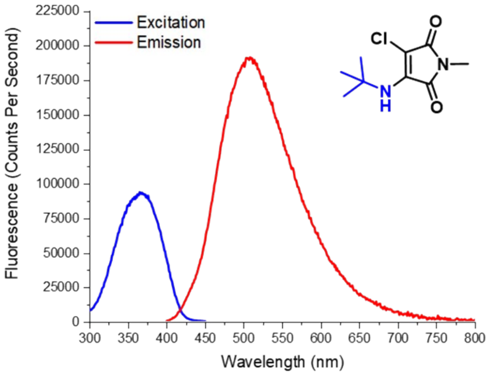


*Figure 42:Fluorescence Spectra of 1d in diethyl ether*

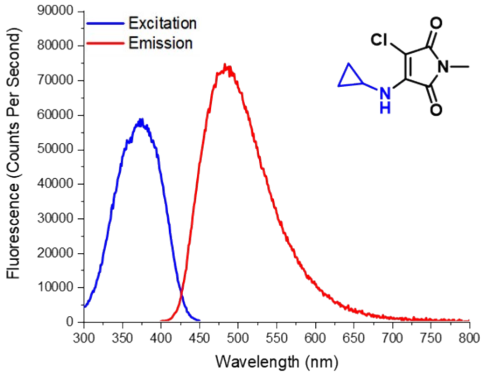


*Figure 43: Fluorescence Spectra of 1e in diethyl ether*

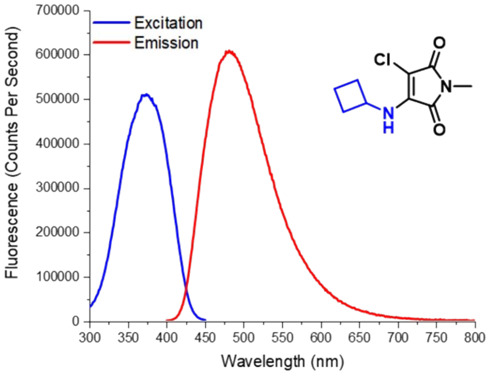


*Figure 44: Fluorescence Spectra of 1f in diethyl ether*

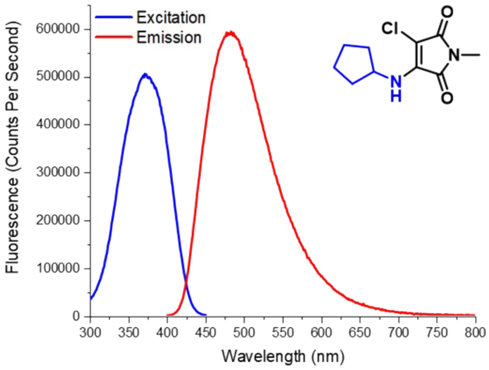


*Figure 45: Fluorescence Spectra of 1g in diethyl ether*

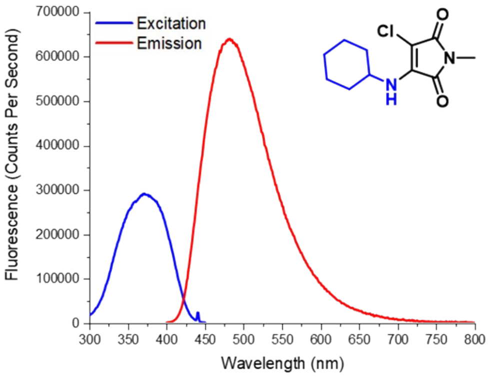


*Figure 46: Fluorescence Spectra of 1h in diethyl ether*

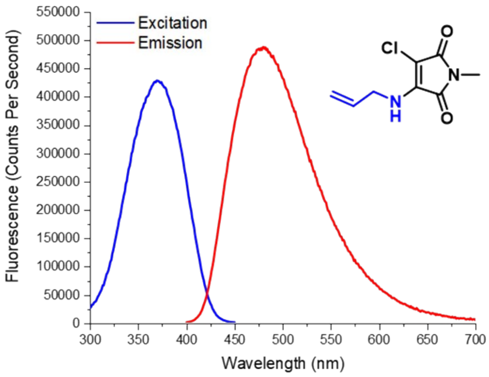


*Figure 47: Fluorescence Spectra of 1i in diethyl ether*

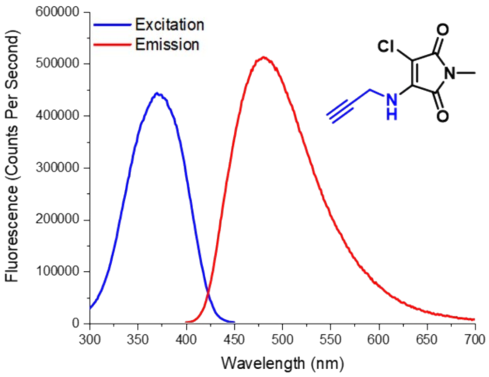


*Figure 48: Fluorescence Spectra of 1j in diethyl ether*

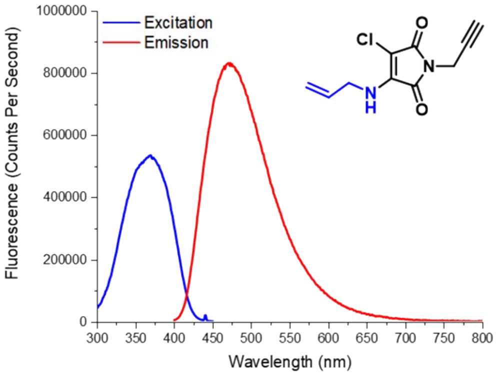


*Figure 49: Fluorescence Spectra of 1k in diethyl ether*

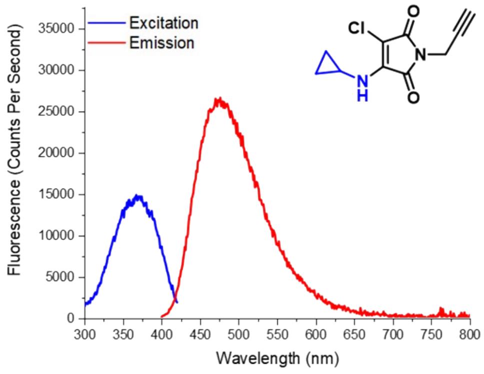


*Figure 50: Fluorescence Spectra of 1l in diethyl ether*

# AM Fluorescence Spectra- Solvatochromism:

We analysed several AM derivatives in numerous solvents, corresponding to the same UV-vis solvation studies, and assessed their fluorescence properties. The elucidation of emission wavelengths and consequently, the Stokes shift allows us to compare these values with our ANN-predicted values, and thus evaluate the predictive capability of the ANN for emission values.

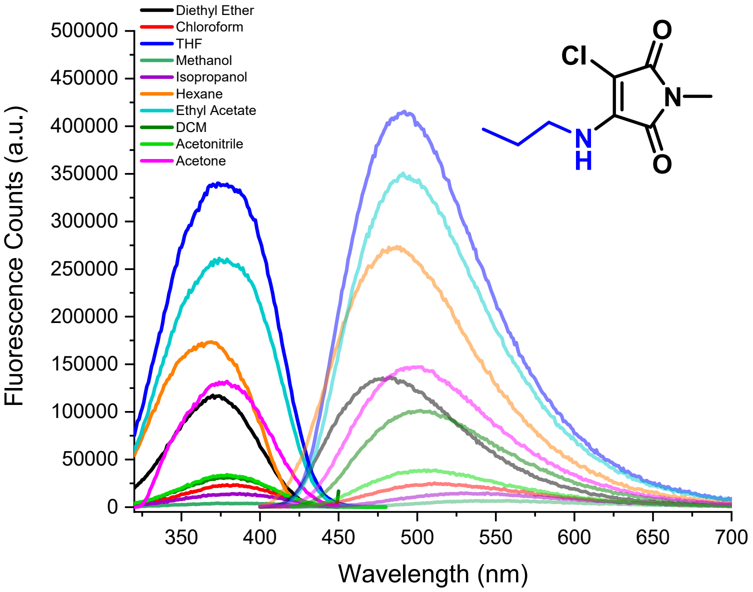


*Figure 51: Stacked Fluorescence Spectra of 1a in various solvents*

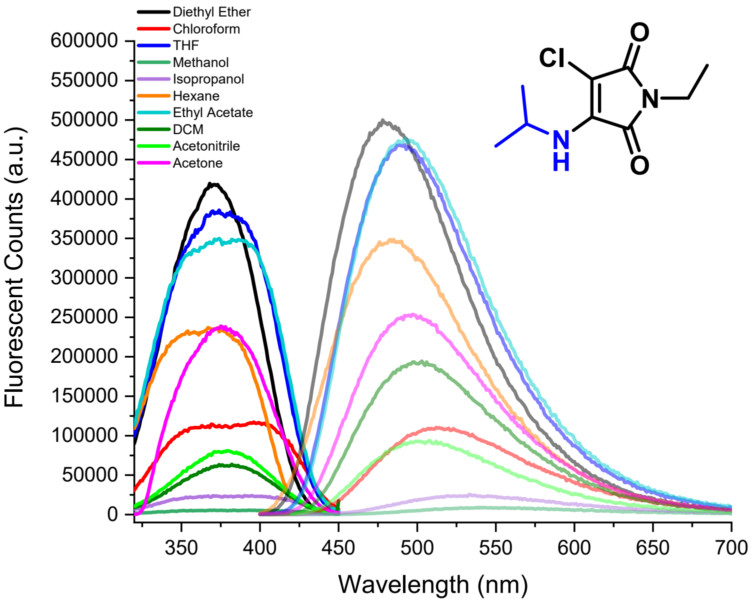


*Figure 52: Stacked Fluorescence Spectra of 1b in various solvents*

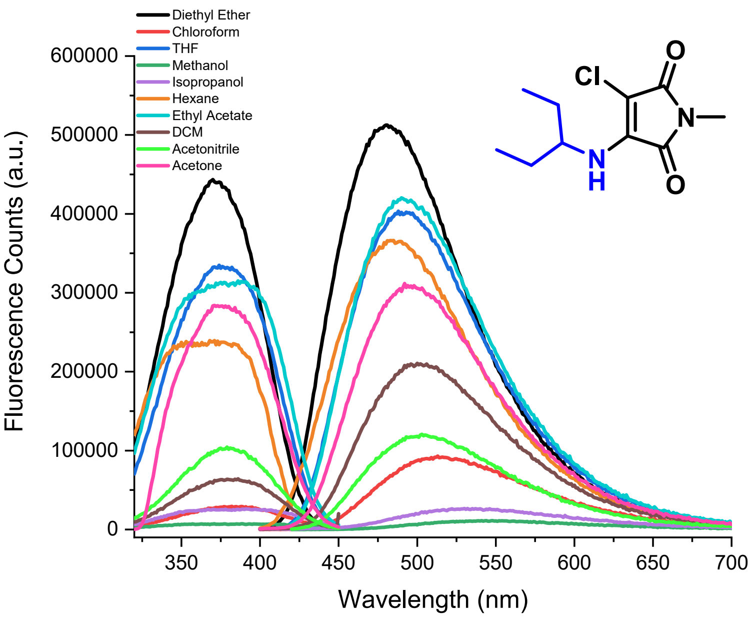


*Figure 53: Stacked Fluorescence Spectra of 1c in various solvents*

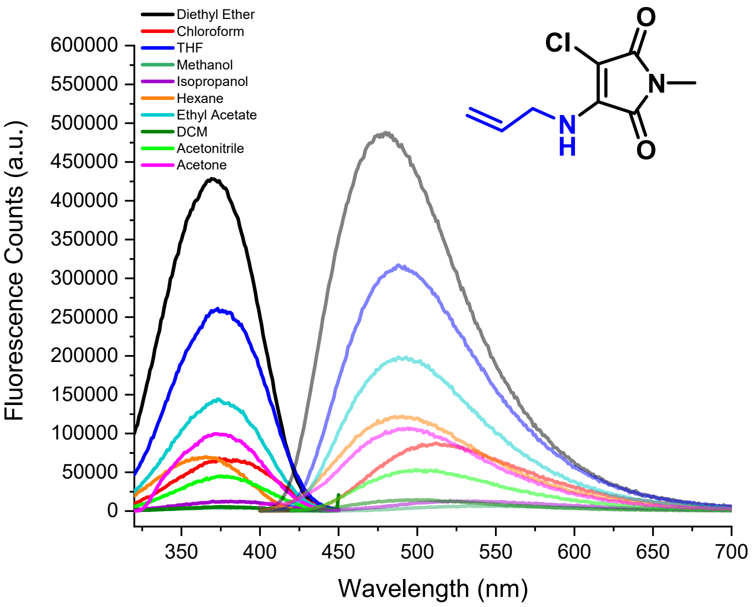


*Figure 54: Stacked Fluorescence Spectra of 1k in various solvents*

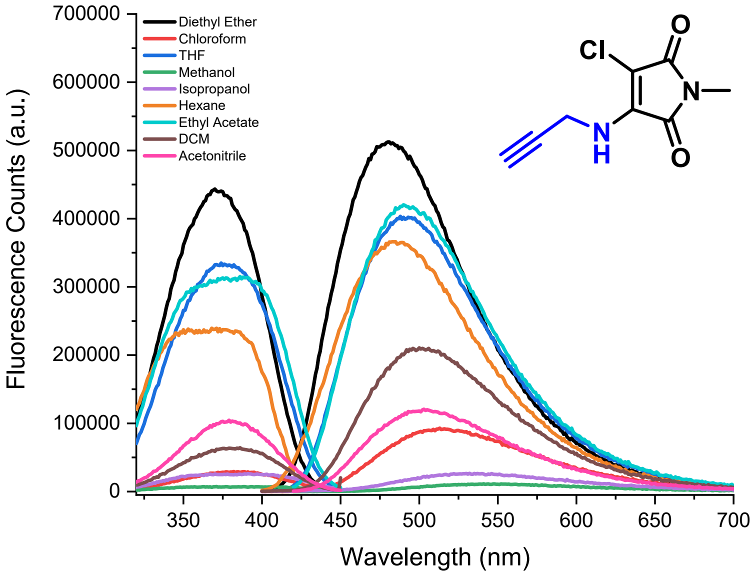


*Figure 55: Stacked Fluorescence Spectra of 1j in various solvents*

## AM Contour Plots

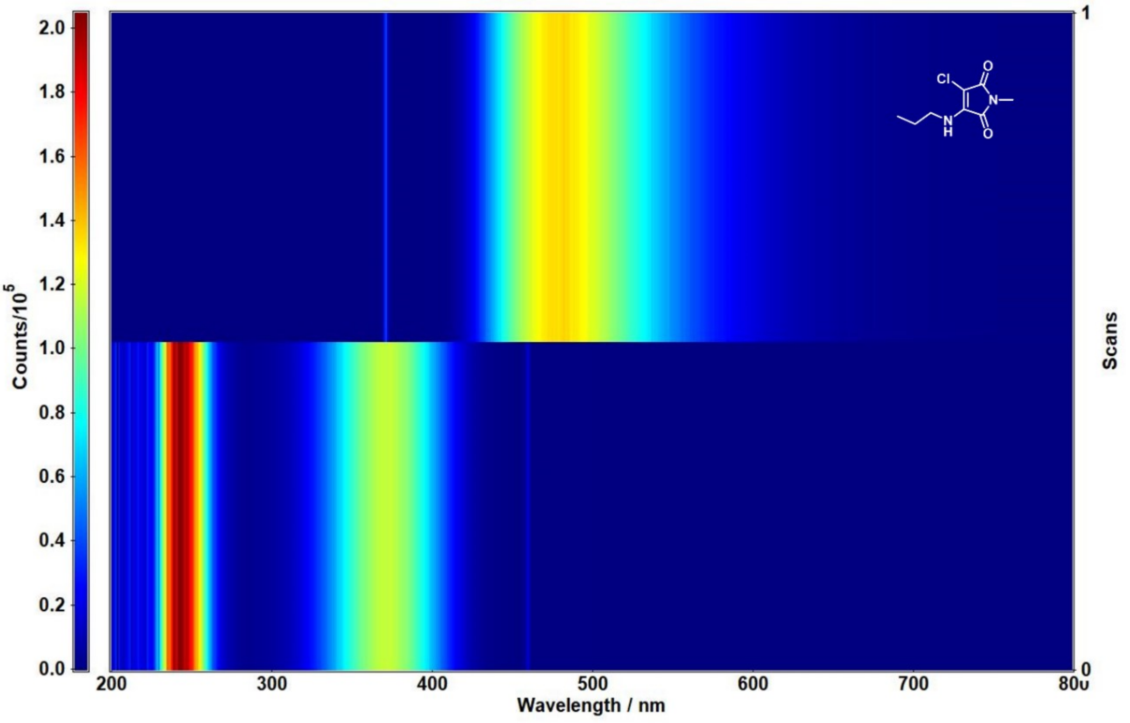


*Figure 56: Fluorescence contour plot of 1a in diethyl ether*

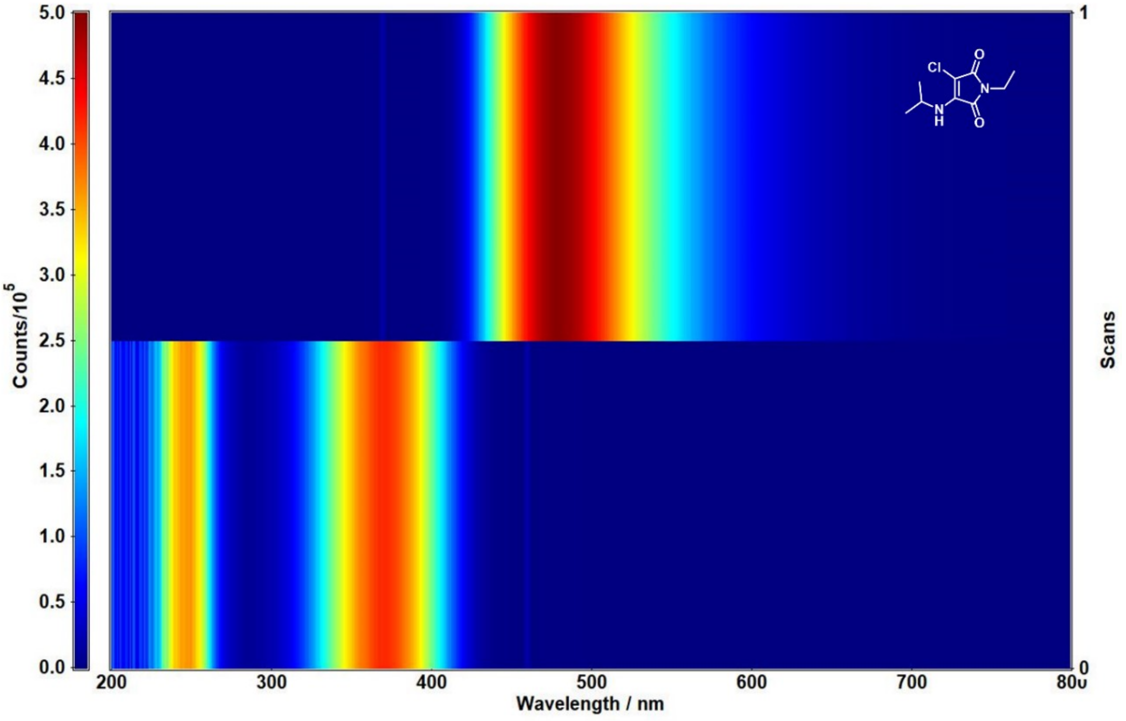


*Figure 57: Fluorescence contour plot of 1b in diethyl ether*

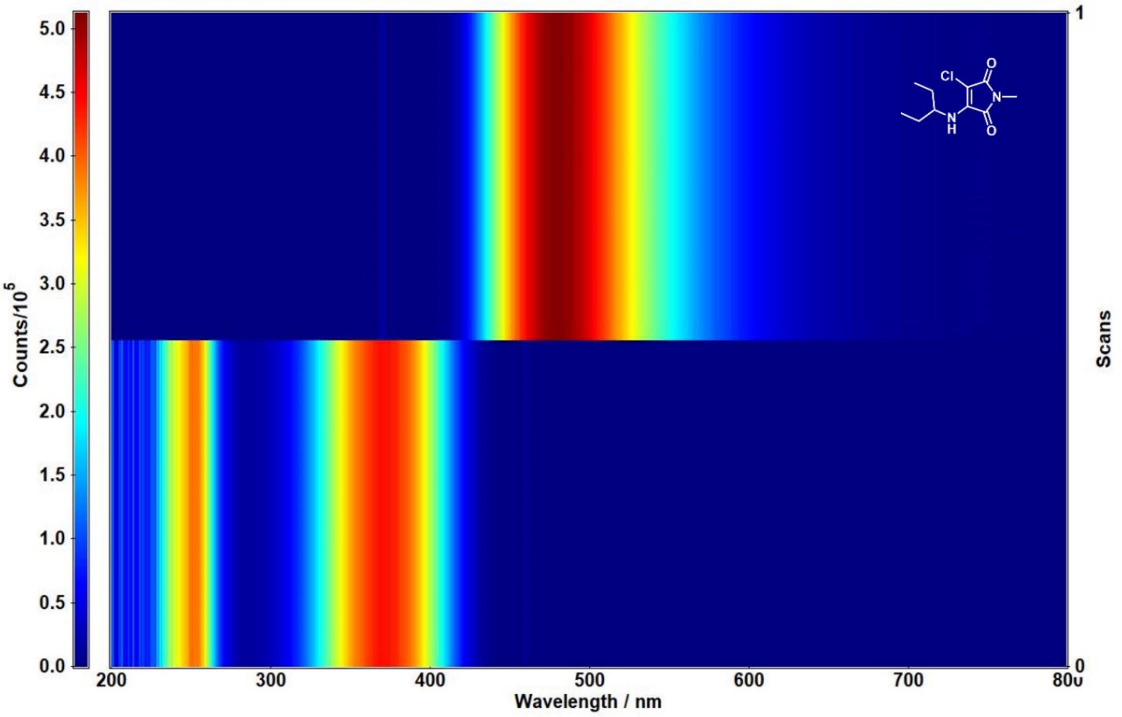


*Figure 58: Fluorescence contour plot of 1c in diethyl ether*

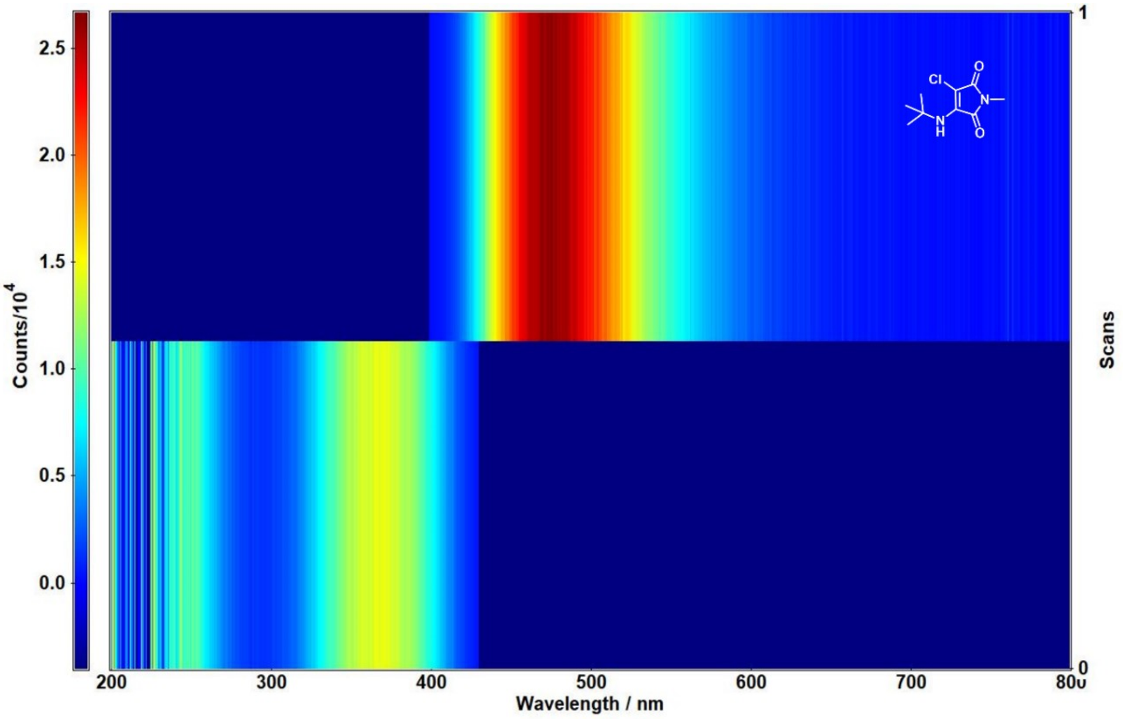


*Figure 59: Fluorescence contour plot of 1d in diethyl ether*

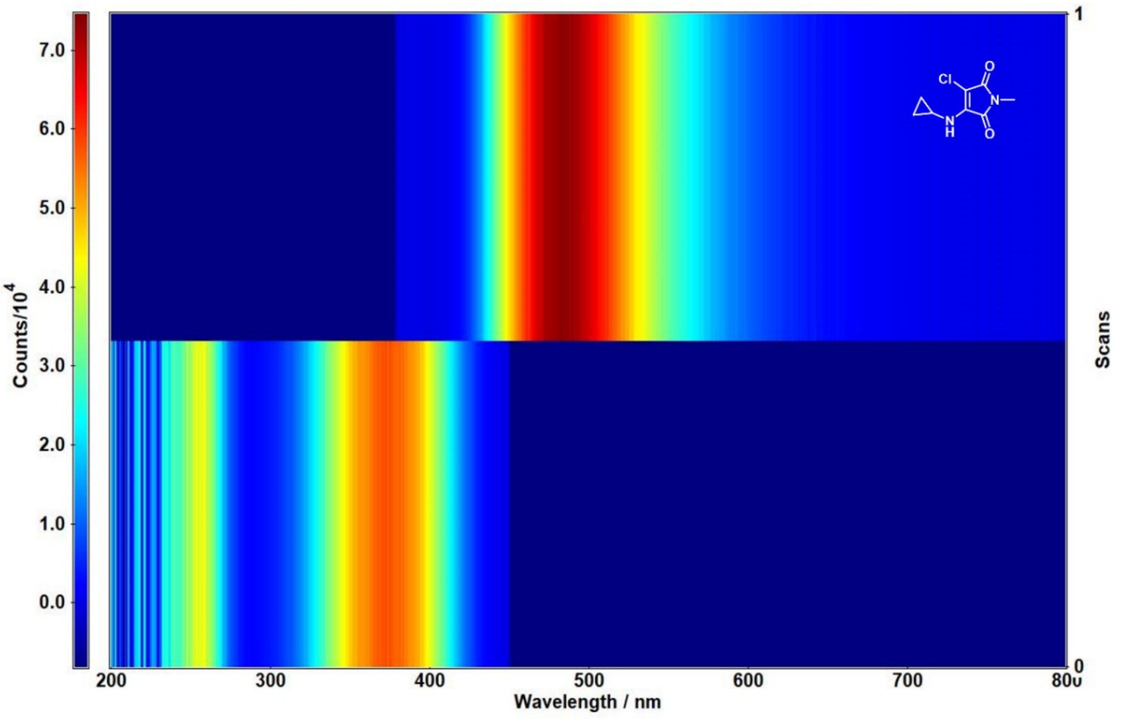


*Figure 60: Fluorescence contour plot of 1e in diethyl ether*

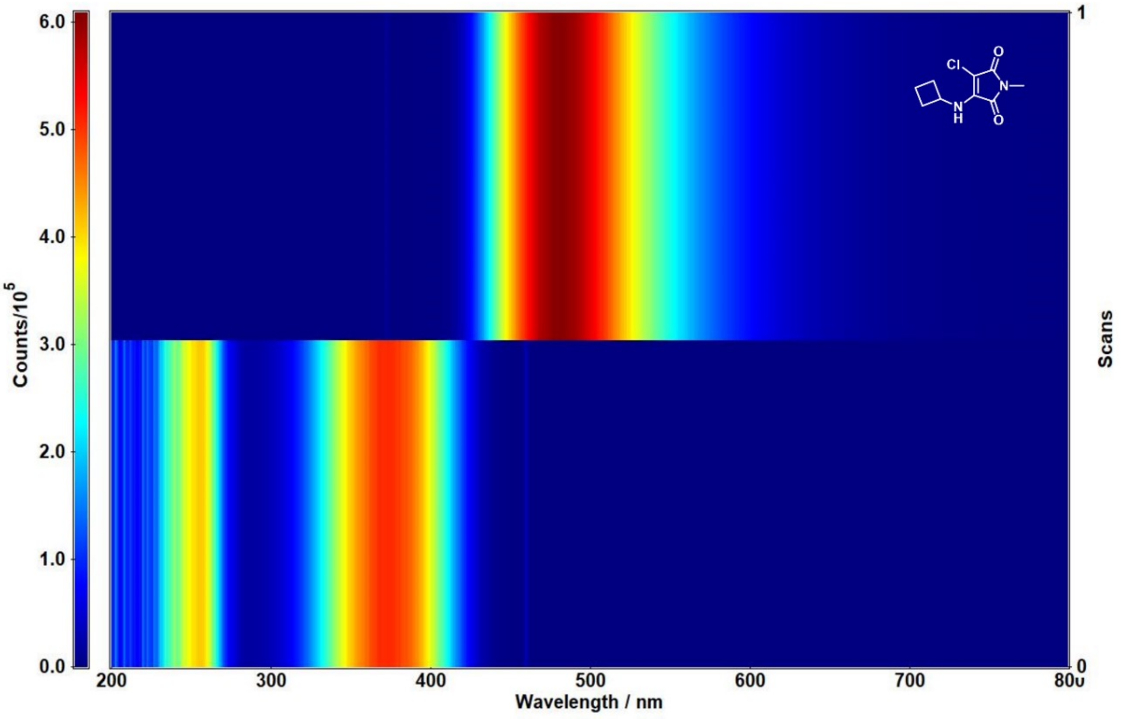


*Figure 61: Fluorescence contour plot of 1f in diethyl ether*

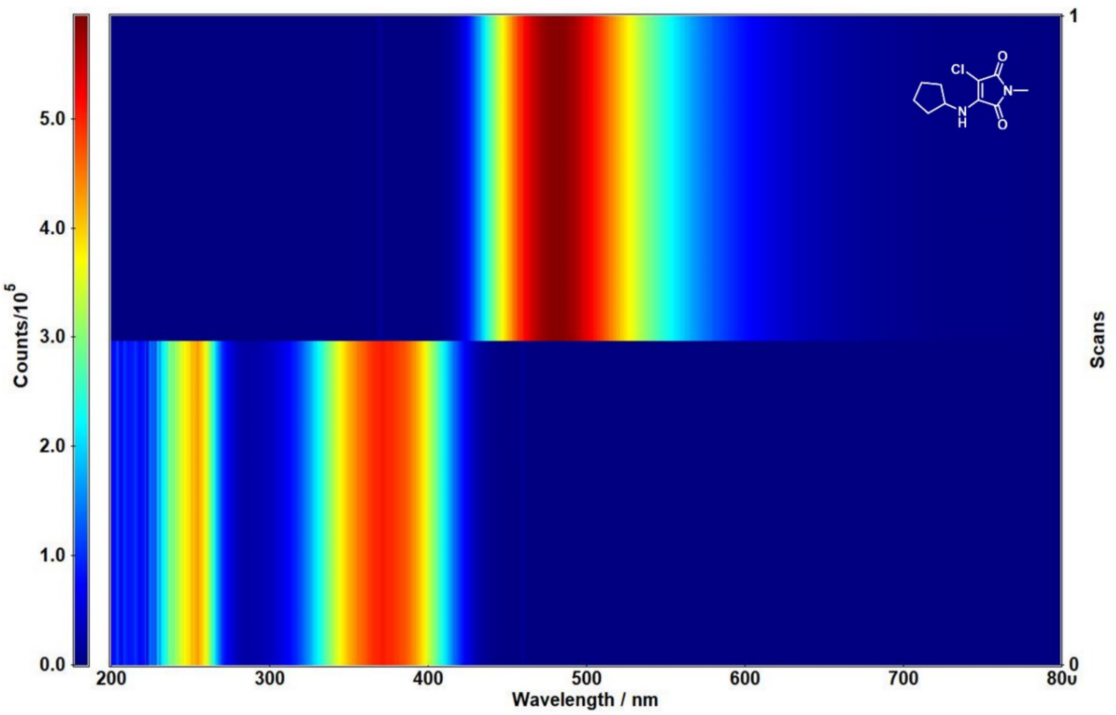


*Figure 62: Fluorescence contour plot of 1g in diethyl ether*

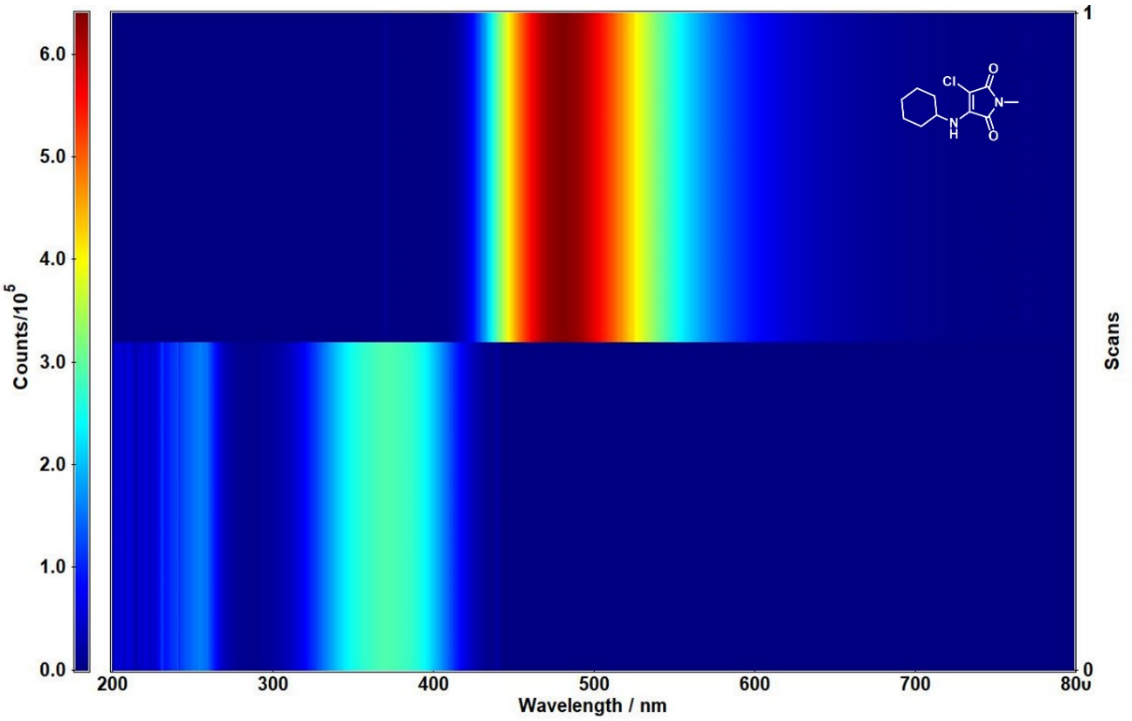


*Figure 63: Fluorescence contour plot of 1h in diethyl ether*

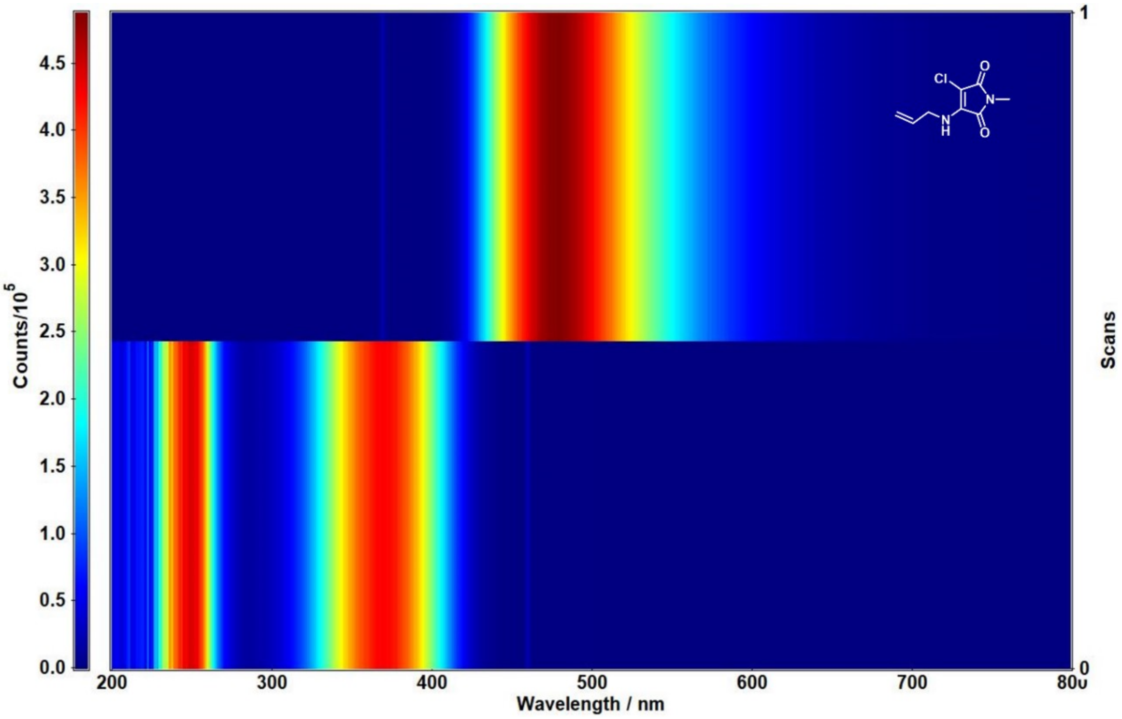


*Figure 64: Fluorescence contour plot of 1i in diethyl ether*

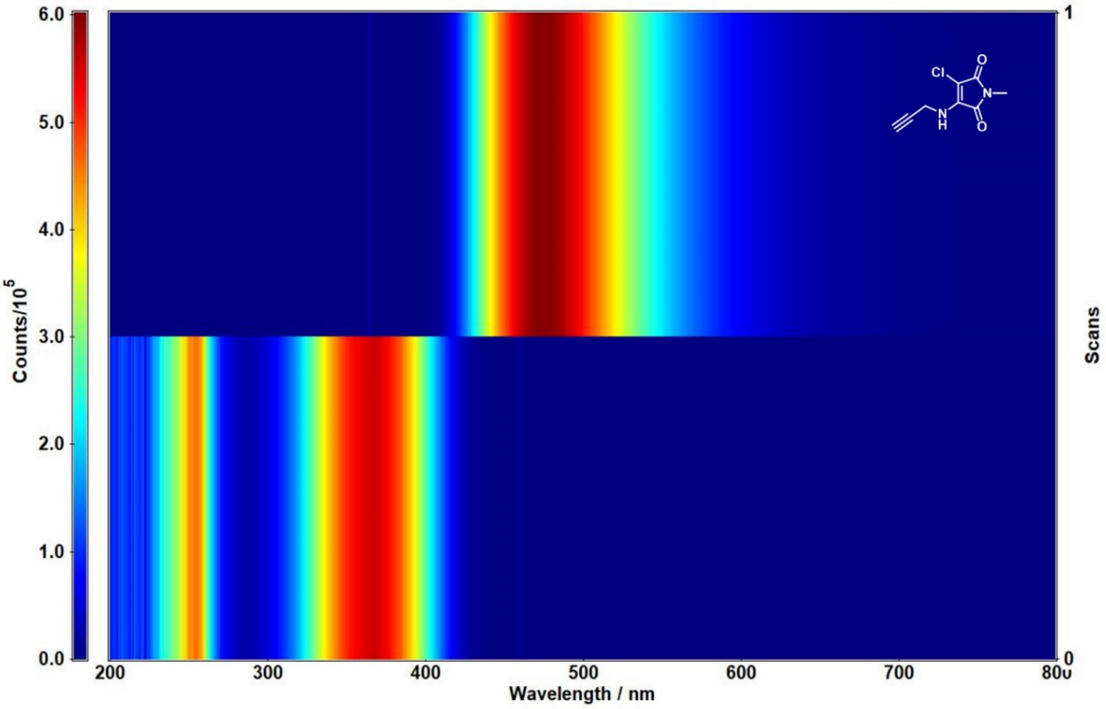


*Figure 65: Fluorescence contour plot of 1j in diethyl ether*

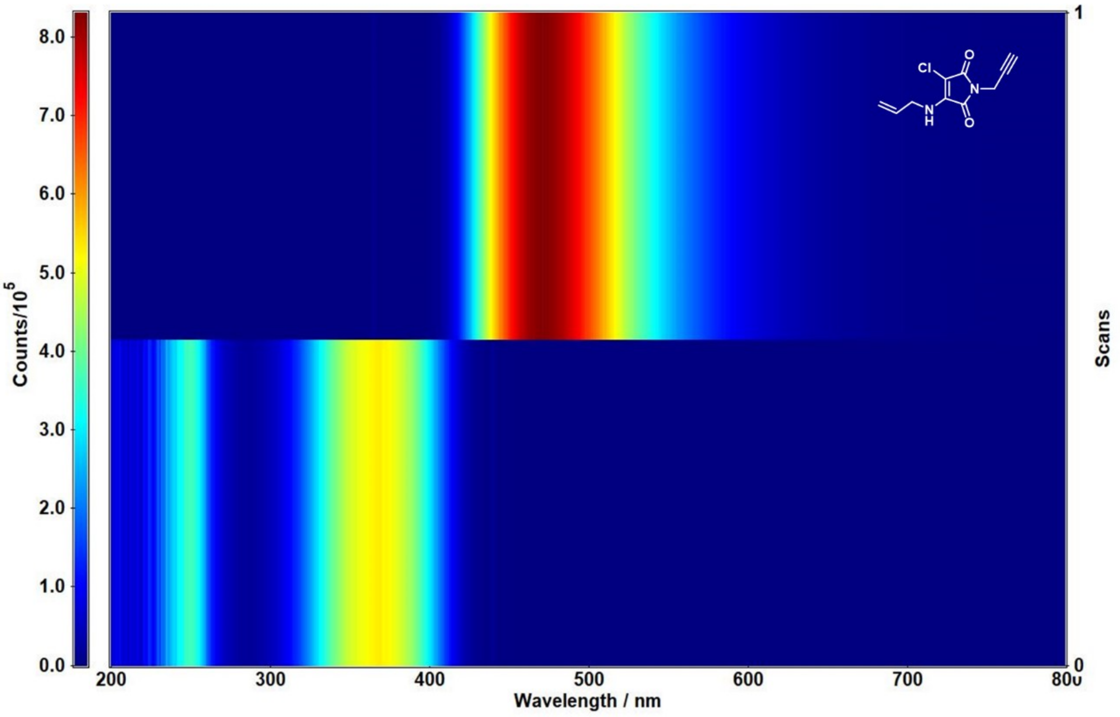


*Figure 66: Fluorescence contour plot of 1k in diethyl ether*

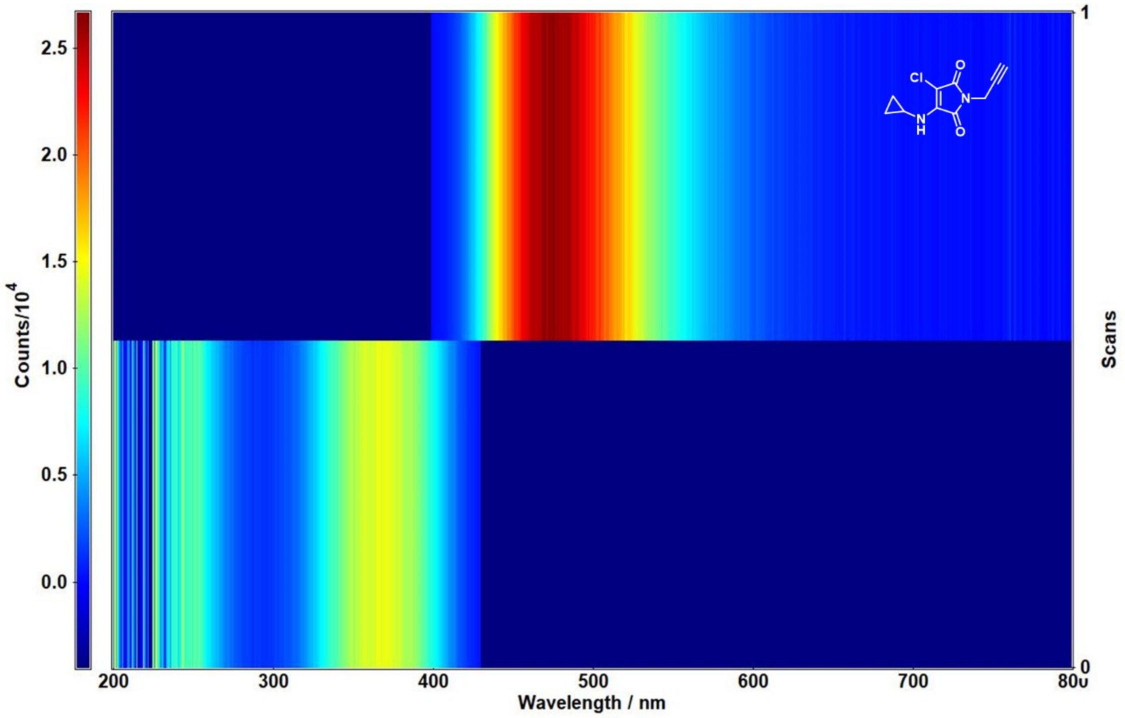


*Figure 67: Fluorescence contour plot of 1l in diethyl ether*

# ANN Solvatochromism of maleimides

Here we tabulate the corresponding wavelength obtained from our ANN notebook when changing the solvent key word. *In our notebook, we include a section which outlines a list of solvents and their corresponding dielectric constant to be used in the calculation. This section allows for easier integration of solvents in the training set, without the user having to convert the solvent to a value for the training set beforehand.*

We observe a general increase in ANN predicted wavelength with an increase in the dielectric constant, which is mostly observed in the experimental findings.

| **1a** | | |
|---|---|---|
| **Solvent** | Dielectric Constant | Wavelength (nm) |
| **Hexane** | 1.88 | 360.39785 |
| **Toluene** | 2.38 | 360.43165 |
| **Diethyl ether** | 4.33 | 360.68033 |
| **Chloroform** | 4.81 | 360.76438 |
| **Ethyl Acetate** | 6.02 | 361.01994 |
| **THF** | 7.58 | 361.80266 |
| **DCM** | 8.93 | 362.66502 |
| **2-propanol** | 17.9 | 367.14524 |
| **Acetone** | 20.7 | 368.09355 |
| **Methanol** | 32.7 | 372.25564 |
| **Acetonitrile** | 37.5 | 373.96409 |
| **DMF** | 37.51 | 373.96765 |

| **1b** | | |
|---|---|---|
| **Solvent** | Dielectric Constant | Wavelength (nm) |
| **Hexane** | 1.88 | 364.50773 |
| **Toluene** | 2.38 | 364.62605 |
| **Diethyl ether** | 4.33 | 365.11432 |
| **Chloroform** | 4.81 | 365.24222 |

| | | |
|---|---|---|
| **Ethyl Acetate** | 6.02 | 365.56464 |
| **THF** | 7.58 | 365.98032 |
| **DCM** | 8.93 | 366.35676 |
| **2-propanol** | 17.9 | 369.23043 |
| **Acetone** | 20.7 | 370.22344 |
| **Methanol** | 32.7 | 374.63421 |
| **Acetonitrile** | 37.5 | 376.42791 |
| **DMF** | 37.51 | 376.43164 |

| **1c** | | |
|---|---|---|
| **Solvent** | Dielectric Constant | Wavelength (nm) |
| **Hexane** | 1.88 | 357.38422 |
| **Toluene** | 2.38 | 357.52017 |
| **Diethyl ether** | 4.33 | 358.09294 |
| **Chloroform** | 4.81 | 358.23687 |
| **Ethyl Acetate** | 6.02 | 358.63659 |
| **THF** | 7.58 | 359.21153 |
| **DCM** | 8.93 | 359.63714 |
| **2-propanol** | 17.9 | 362.79572 |
| **Acetone** | 20.7 | 363.8336 |
| **Methanol** | 32.7 | 368.38375 |
| **Acetonitrile** | 37.5 | 370.17222 |
| **DMF** | 37.51 | 370.1754 |

| 1d | | |
|---|---|---|
| **Solvent** | Dielectric Constant | Wavelength (nm) |
| **Hexane** | 1.88 | 361.58972 |
| **Toluene** | 2.38 | 361.73608 |
| **Diethyl ether** | 4.33 | 362.42294 |
| **Chloroform** | 4.81 | 362.60297 |
| **Ethyl Acetate** | 6.02 | 363.05678 |
| **THF** | 7.58 | 363.68363 |
| **DCM** | 8.93 | 364.24892 |
| **2-propanol** | 17.9 | 367.7419 |
| **Acetone** | 20.7 | 368.80211 |
| **Methanol** | 32.7 | 373.38735 |
| **Acetonitrile** | 37.5 | 375.21668 |
| **DMF** | 37.51 | 375.21987 |

| 1e | | |
|---|---|---|
| **Solvent** | Dielectric Constant | Wavelength (nm) |
| **Hexane** | 1.88 | 368.1072 |
| **Toluene** | 2.38 | 368.23117 |
| **Diethyl ether** | 4.33 | 368.71463 |
| **Chloroform** | 4.81 | 368.83364 |
| **Ethyl Acetate** | 6.02 | 369.13364 |
| **THF** | 7.58 | 369.52041 |
| **DCM** | 8.93 | 369.91825 |
| **2-propanol** | 17.9 | 372.58964 |
| **Acetone** | 20.7 | 373.50082 |
| **Methanol** | 32.7 | 377.99516 |

| | | |
|---|---|---|
| **Acetonitrile** | 37.5 | 379.82926 |
| **DMF** | 37.51 | 379.83308 |

| **1f** | | |
|---|---|---|
| **Solvent** | Dielectric Constant | Wavelength (nm) |
| **Hexane** | 1.88 | 368.38728 |
| **Toluene** | 2.38 | 368.50378 |
| **Diethyl ether** | 4.33 | 368.97399 |
| **Chloroform** | 4.81 | 369.094 |
| **Ethyl Acetate** | 6.02 | 369.39299 |
| **THF** | 7.58 | 369.84962 |
| **DCM** | 8.93 | 370.26014 |
| **2-propanol** | 17.9 | 373.00604 |
| **Acetone** | 20.7 | 373.86317 |
| **Methanol** | 32.7 | 377.99289 |
| **Acetonitrile** | 37.5 | 379.82113 |
| **DMF** | 37.51 | 379.82495 |

| 1g | | |
|---|---|---|
| **Solvent** | Dielectric Constant | Wavelength (nm) |
| **Hexane** | 1.88 | 363.44695 |
| **Toluene** | 2.38 | 363.56983 |
| **Diethyl ether** | 4.33 | 364.07675 |
| **Chloroform** | 4.81 | 364.2029 |
| **Ethyl Acetate** | 6.02 | 364.54241 |
| **THF** | 7.58 | 364.98249 |
| **DCM** | 8.93 | 365.37488 |
| **2-propanol** | 17.9 | 368.09973 |
| **Acetone** | 20.7 | 368.97876 |
| **Methanol** | 32.7 | 373.24478 |
| **Acetonitrile** | 37.5 | 375.03674 |
| **DMF** | 37.51 | 375.04056 |

| 1h | | |
|---|---|---|
| **Solvent** | Dielectric Constant | Wavelength (nm) |
| **Hexane** | 1.88 | 367.00003 |
| **Toluene** | 2.38 | 367.13001 |
| **Diethyl ether** | 4.33 | 367.67481 |
| **Chloroform** | 4.81 | 367.80949 |
| **Ethyl Acetate** | 6.02 | 368.15953 |
| **THF** | 7.58 | 368.62137 |
| **DCM** | 8.93 | 369.02103 |
| **2-propanol** | 17.9 | 371.75785 |
| **Acetone** | 20.7 | 372.61498 |
| **Methanol** | 32.7 | 376.54832 |

| | | |
|---|---|---|
| **Acetonitrile** | 37.5 | 378.25517 |
| **DMF** | 37.51 | 378.25899 |

| **1i** | | |
|---|---|---|
| **Solvent** | Dielectric Constant | Wavelength (nm) |
| **Hexane** | 1.88 | 361.87441 |
| **Toluene** | 2.38 | 361.99594 |
| **Diethyl ether** | 4.33 | 362.50727 |
| **Chloroform** | 4.81 | 362.63133 |
| **Ethyl Acetate** | 6.02 | 362.98467 |
| **THF** | 7.58 | 363.50071 |
| **DCM** | 8.93 | 364.00504 |
| **2-propanol** | 17.9 | 367.6821 |
| **Acetone** | 20.7 | 368.69027 |
| **Methanol** | 32.7 | 373.19834 |
| **Acetonitrile** | 37.5 | 375.03244 |
| **DMF** | 37.51 | 375.03626 |

| 1j | | |
|---|---|---|
| **Solvent** | Dielectric Constant | Wavelength (nm) |
| **Hexane** | 1.88 | 369.35854 |
| **Toluene** | 2.38 | 369.52039 |
| **Diethyl ether** | 4.33 | 370.15161 |
| **Chloroform** | 4.81 | 370.30698 |
| **Ethyl Acetate** | 6.02 | 370.73293 |
| **THF** | 7.58 | 371.31296 |
| **DCM** | 8.93 | 371.81491 |
| **2-propanol** | 17.9 | 375.17063 |
| **Acetone** | 20.7 | 376.188 |
| **Methanol** | 32.7 | 380.77324 |
| **Acetonitrile** | 37.5 | 382.60733 |
| **DMF** | 37.51 | 382.61115 |

| 1k | | |
|---|---|---|
| **Solvent** | Dielectric Constant | Wavelength (nm) |
| **Hexane** | 1.88 | 362.50101 |
| **Toluene** | 2.38 | 362.62055 |
| **Diethyl ether** | 4.33 | 363.19213 |
| **Chloroform** | 4.81 | 363.33666 |
| **Ethyl Acetate** | 6.02 | 363.75806 |
| **THF** | 7.58 | 364.34599 |
| **DCM** | 8.93 | 364.85315 |
| **2-propanol** | 17.9 | 367.92995 |
| **Acetone** | 20.7 | 368.94168 |
| **Methanol** | 32.7 | 373.45272 |

| | | |
|---|---|---|
| **Acetonitrile** | 37.5 | 375.29291 |
| **DMF** | 37.51 | 375.29674 |

| **1l** | | |
|---|---|---|
| **Solvent** | Dielectric Constant | Wavelength (nm) |
| **Hexane** | 1.88 | 370.33218 |
| **Toluene** | 2.38 | 370.46169 |
| **Diethyl ether** | 4.33 | 370.9668 |
| **Chloroform** | 4.81 | 371.09113 |
| **Ethyl Acetate** | 6.02 | 371.40455 |
| **THF** | 7.58 | 371.87781 |
| **DCM** | 8.93 | 372.29222 |
| **2-propanol** | 17.9 | 375.24666 |
| **Acetone** | 20.7 | 376.25098 |
| **Methanol** | 32.7 | 380.84087 |
| **Acetonitrile** | 37.5 | 382.67906 |
| **DMF** | 37.51 | 382.68289 |

*Table 3: ANN-derived absorbance values with varying solvent*

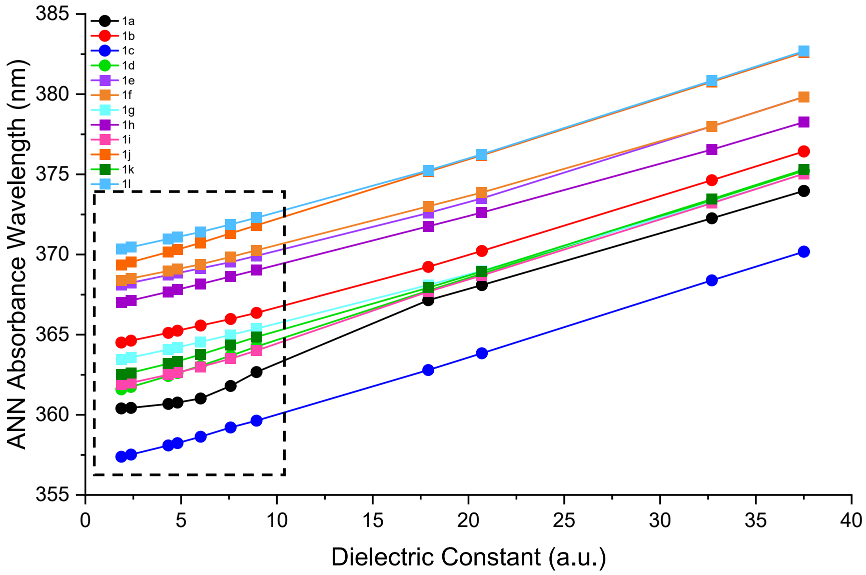


*Figure 68: Figure 68: ANN wavelength response to varying the dielectric constant for AM derivatives*

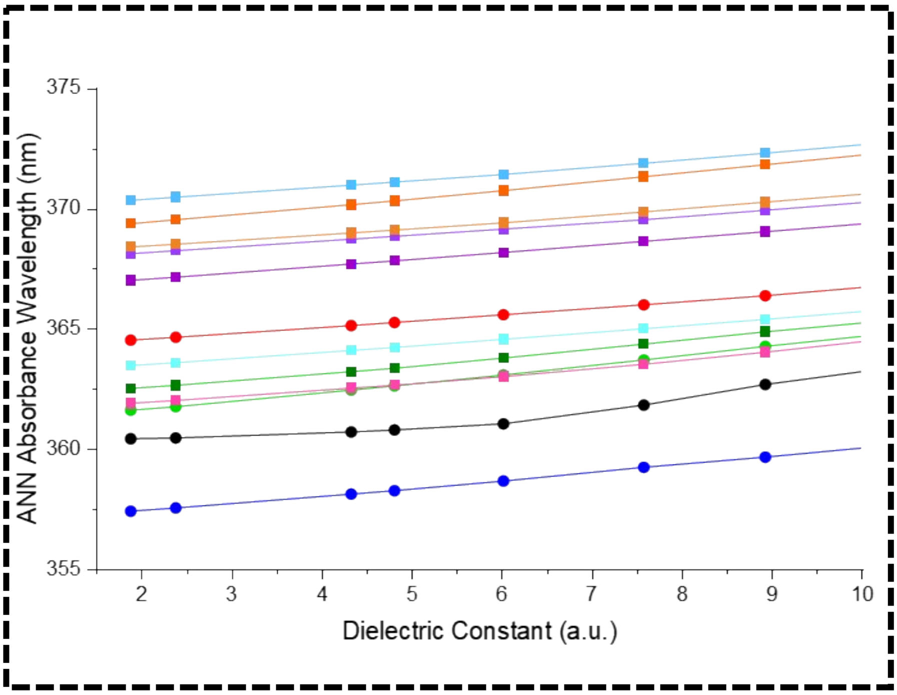


*Figure 69: ANN wavelength response to varying the dielectric constant for AM derivatives (zoomed to dielectric constant 0-10).*

# ANN Combination with Experimental and TD-DFT values: AMs

We also analysed the wavelength values attributed to their maximum absorbance or emission values and plotted the difference in predicted (ANN/ TD-DFT values) *vs* experimental values as a function of the solvent dielectric constant. Generally, we found that solvents with lower dielectric constants fared better in TD-DFT or ANN calculations. Overall, we noticed that the TD-DFT values tend to plateau with relatively low dielectric constants, contrary to the experimental results.

We observed two solvents in which the experimental findings significantly differed from predicted values. Both chloroform and acetonitrile, despite having distinctly different dielectric constant values, showed poor performance and substantial overprediction of excitation wavelengths in some cases. In the case of acetonitrile, we hypothesise that this is due to the high dielectric constant it possesses while remaining a medium-polarity solvent (compared to water).

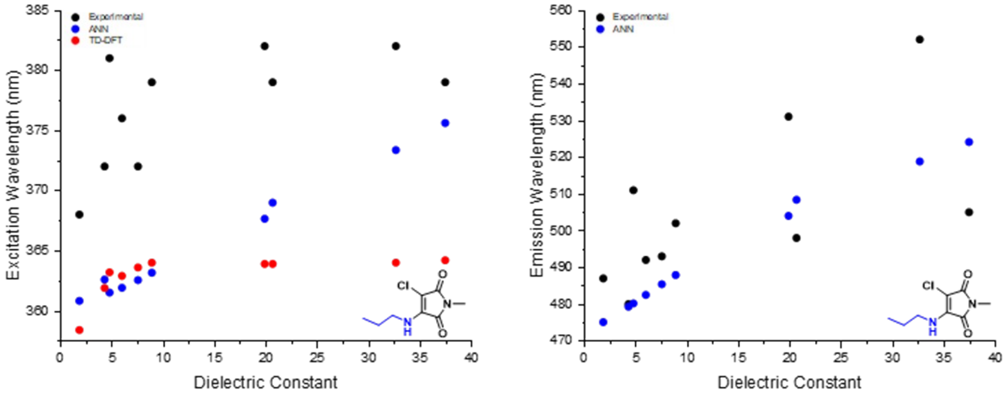


*Figure 70: Experimental, TD-DFT and ANN-predicted wavelengths, excitation (left) and emission (right) for 1a*

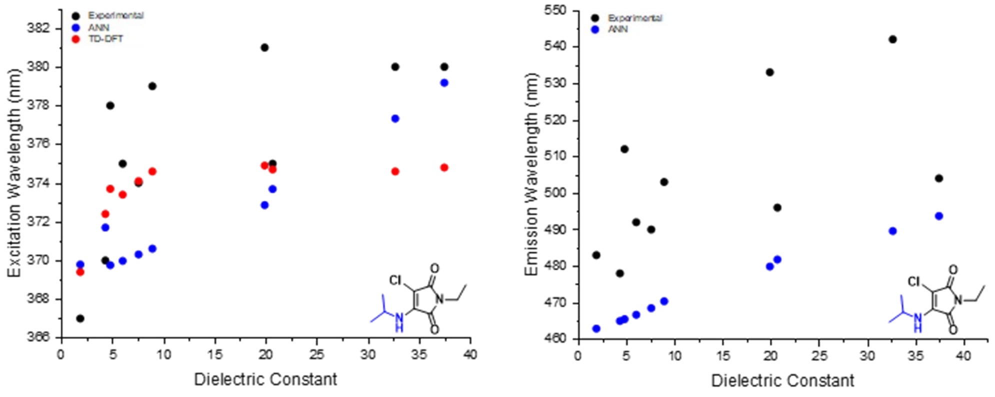


*Figure 71: Experimental, TD-DFT and ANN-predicted wavelengths, excitation (left) and emission (right) for 1b*

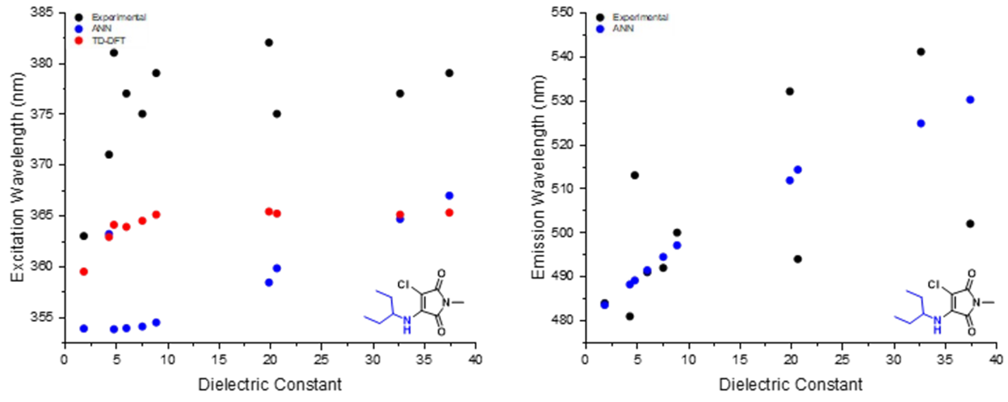


*Figure 72: Experimental, TD-DFT and ANN-predicted wavelengths, excitation (left) and emission (right) for 1c*

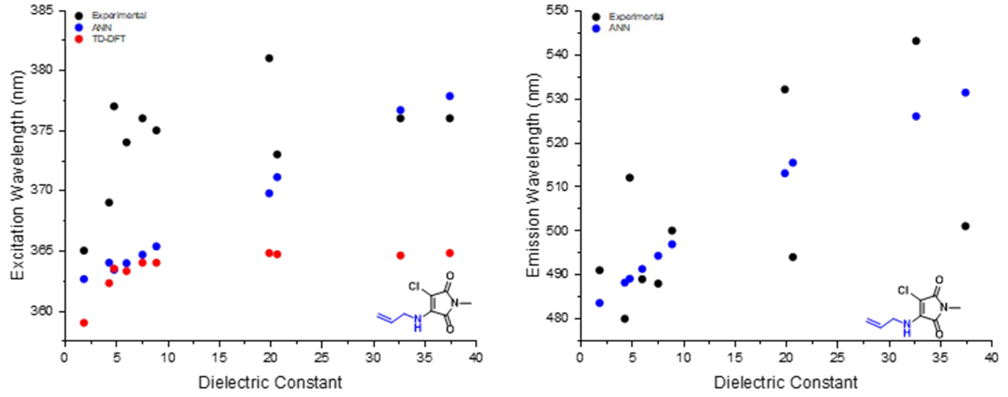


*Figure 73: Experimental, TD-DFT and ANN-predicted wavelengths, excitation (left) and emission (right) for 1i*

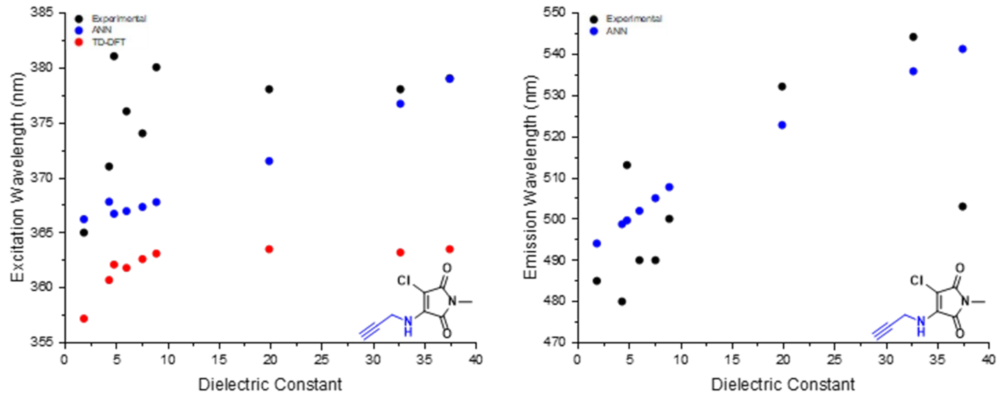


*Figure 74: Experimental, TD-DFT and ANN-predicted wavelengths, excitation (left) and emission (right) for 1j*

We also split the above graphs to plot the difference from experimental values as a function of dielectric constant to identify common trends exhibited by the ANN.

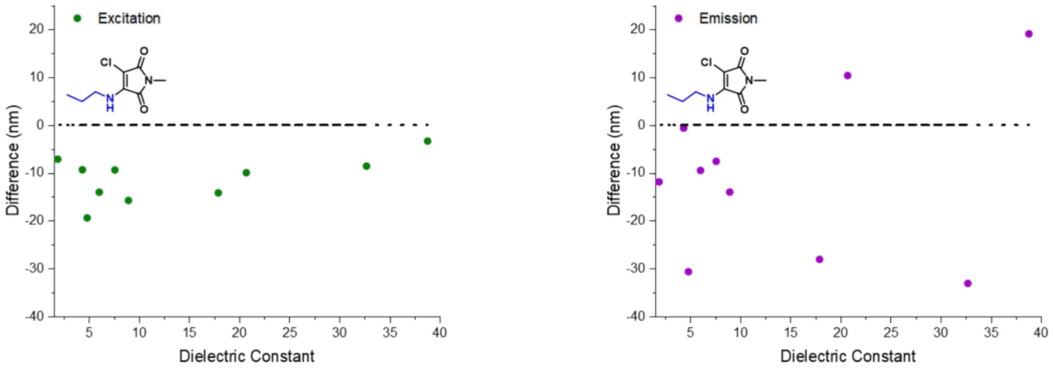


*Figure 75: ANN predicted difference as a function of dielectric constant for excitation (left) and emission (right) of 1a*

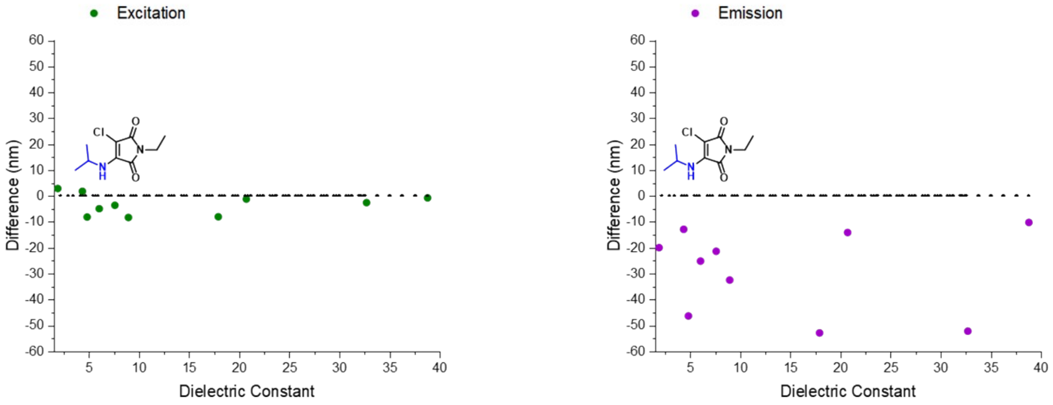


*Figure 76: ANN predicted difference as a function of dielectric constant for excitation (left) and emission (right) of 1b*

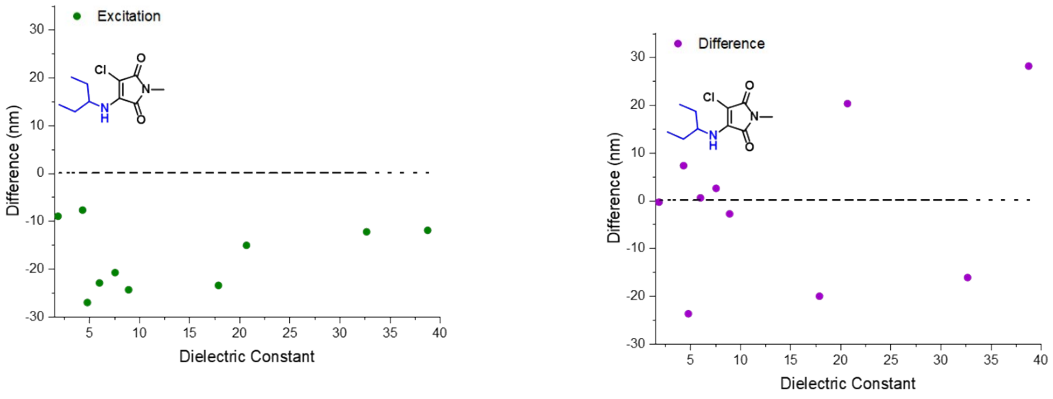


*Figure 77: ANN predicted difference as a function of dielectric constant for excitation (left) and emission (right) of 1c*

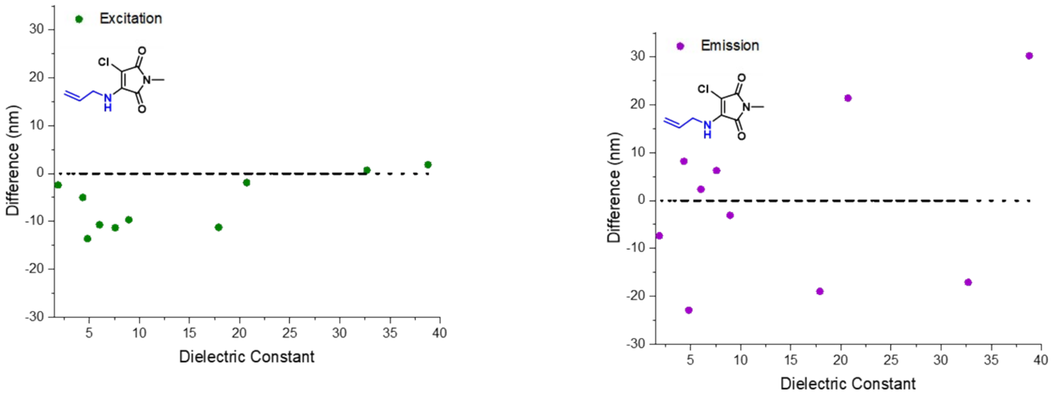


*Figure 78: ANN predicted difference as a function of dielectric constant for excitation (left) and emission (right) of 1i*

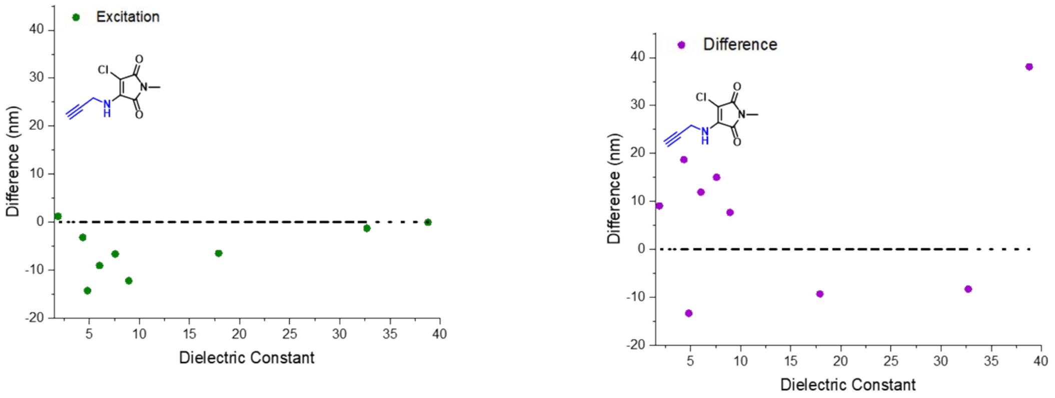


*Figure 79: ANN predicted difference as a function of dielectric constant for excitation (left) and emission (right) of 1j*

From our analysis of the above graphs, we found that typically, the excitation predictions outperform the emission predictions, as there is less scatter about the "zero" line in these measurements. While still valid, and predicting typically within 25 nm, the predicted emission values tend to exhibit a higher scatter about the "zero" line, appearing to be more stochastic than the excitation values.

Interestingly, the excitation predictions tend to be more underestimated, sometimes predicting 30 nm lower wavelengths than the experimental measurement. Conversely, despite showing more variation across the series, the emission values tend to overpredict heavily, while also showing underestimation in some cases. Among both excitation and emission, however, the predictions tend to be more accurate towards lower solvent dielectric constants.

# General procedure for DTM synthesis

A dibromo starting material was required due to the relative lability of the carbon-bromine bond compared to the chlorinated analogue. This was synthesised from the corresponding brominated maleic anhydride, and the maleimide was synthesised using methylamine hydrochloride in the presence of sodium acetate in acetic acid as outlined previously (DBM).

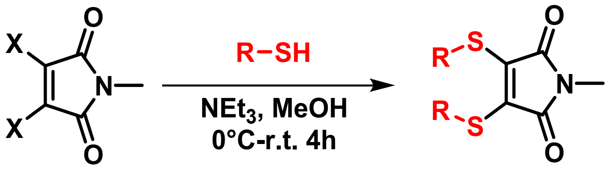


*Figure 80: General procedure for synthesis of DTMs*

An excess of thiol was added in the presence of triethylamine to a solution of dibromo-N-methylmaleimide and stirred overnight. The solution was initially cooled in an ice bath prior to thiol addition, as we observed this cooling reduced a white precipitate formation. The reaction progress was monitored *via* TLC, and a UV spotlight was used to observe a colour change from blue, to green, to yellow. The reaction mixture was then diluted in dichloromethane, and washed with ammonium chloride (10 mL), and water (3x 20 mL), before drying over magnesium sulphate and drying under vacuum. The resulting product was additionally purified through silica gel chromatography, using a varied gradient of hexane and ethyl acetate.

The general procedure for synthesising DTMs was adapted from ***J. Am. Chem. Soc. 2013, 135, 8, 2875–2878.***

## **2a** (3,4-bis((4-chlorophenyl)thio)-1-methyl-1H-pyrrole-2,5-dione)

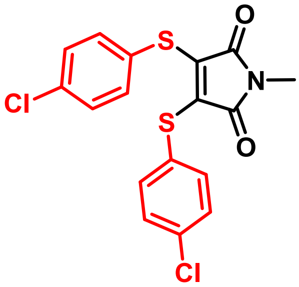

DBM (0.846 mmol, 152.2 mg) was dissolved in THF (8 mL) and cooled in an ice bath. 4-chlorothiophenol (1.71 mmol) was then added gradually along with dropwise triethylamine (2.49 mmol, 0.31 mL). A yellow and clumpy solution was observed upon the addition of all reagents, and the solution was allowed to warm to room temperature, before quickly sonicating and returning to the stirrer plate. This act dispersed the clumps in the solution. The solution was left to stir overnight at room temperature. The solution was diluted with chloroform (50 mL), washed with a 30 mL portion of ammonium chloride, a further three 30 mL portions of distilled water, brine and dried over magnesium sulphate before concentrating under reduced pressure. This yielded an orange solid with a 90.4% yield.

*$^{1}$H NMR (400 MHz, Chloroform-d) δ 7.18 – 7.11 (m, 4H), 7.08 – 7.02 (m, 4H), 2.92 (s, 3H).*

*$^{13}$C NMR (101 MHz, Chloroform-d) δ 165.52, 134.28, 133.82, 132.06, 128.17, 126.13, 23.72.*

MS (ESI) *m/z* calculated - 396, observed- 394.9603.

FTIR (cm$^{-1}$): *2971-2819 broad, 1692- broad.*

## **2b** (3,4-bis(isopropylthio)-1-methyl-1H-pyrrole-2,5-dione)

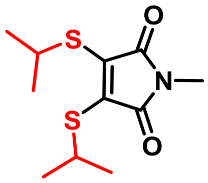

DBM (0.55 mmol, 100 mg) was dissolved in THF (6 mL) to form a yellow solution. 2-propanethiol (1.21 mmol, 0.11 mL) was then added *via a* syringe, where an immediate change in solution viscosity was observed. Triethylamine (1.65 mmol, 0.23mL) was added dropwise where the solution became brighter. The solution was left to stir for four hours at room temperature before being diluted with dichloromethane (50 mL), subjected to an ammonium chloride wash (30 mL), a saturated sodium carbonate wash, three distilled water washes (3x 30 mL). Afterwards, the solution was washed finally with brine and dried over magnesium sulphate before drying under reduced pressure. This afforded a yellow solid in 66.4% yield.

*$^{1}$H NMR (400 MHz, Chloroform-d) δ 4.26 (hept, J = 6.8 Hz, 2H), 2.96 (s, 3H), 1.27 (d, J = 6.7 Hz, 12H).*

*$^{13}$C NMR (101 MHz, Chloroform-d) δ 23.96, 24.52, 36.67, 136.94, 166.96. *

MS (ESI) *m/z* calculated 259.0695, found 259.0695.

FTIR (cm$^{-1}$): *3007-2770 broad, 1693- sharp.*

## **2c** (3,4-bis(ethylthio)-1-methyl-1H-pyrrole-2,5-dione)

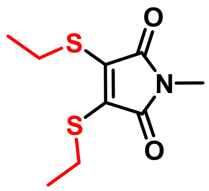


To a 20 mL vial was added DBM (0.76 mmol, 204.3 mg) and dissolved in THF (15 mL). Triethylamine (1.66 mmol, 0.23 mL) was added *via* micropipette to the yellow solution. An excess of ethanethiol was also added using a micropipette (1.89 mmol, 0.136 mL) yielding a strong yellow solution. This reaction was stirred for 16 hours, before dilution with 20 mL of dichloromethane, and a single wash with saturated ammonium chloride (10 mL), then three water washes (10 mL each), brine (10 mL) and drying over magnesium sulphate, before concentration under reduced pressure. The resulting oil was then purified *via* column chromatography using an ethyl acetate and hexane solvent system. Initial 1:9 to 3:7 ratio respectively. The resulting fractions were dried under reduced pressure and in a vacuum oven, whereby 96.9 mg of oil was obtained in 55.4% yield.

*1H NMR (400 MHz, Chloroform-d) δ 3.24 (q, J = 7.4 Hz, 4H), 2.95 (s, 3H), 1.27 (t, J = 7.4 Hz, 6H).*

*13C NMR (101 MHz, Chloroform-d) δ 165.76, 134.71, 25.30, 24.65, 24.48, 23.41, 14.56, 12.40.*

MS (ESI) *m/z* calculated - 231, observed- 231.0382.

FTIR ($cm^{-1}$): *3008-2784 broad, 1669- sharp.*

# DTM NMR Spectra

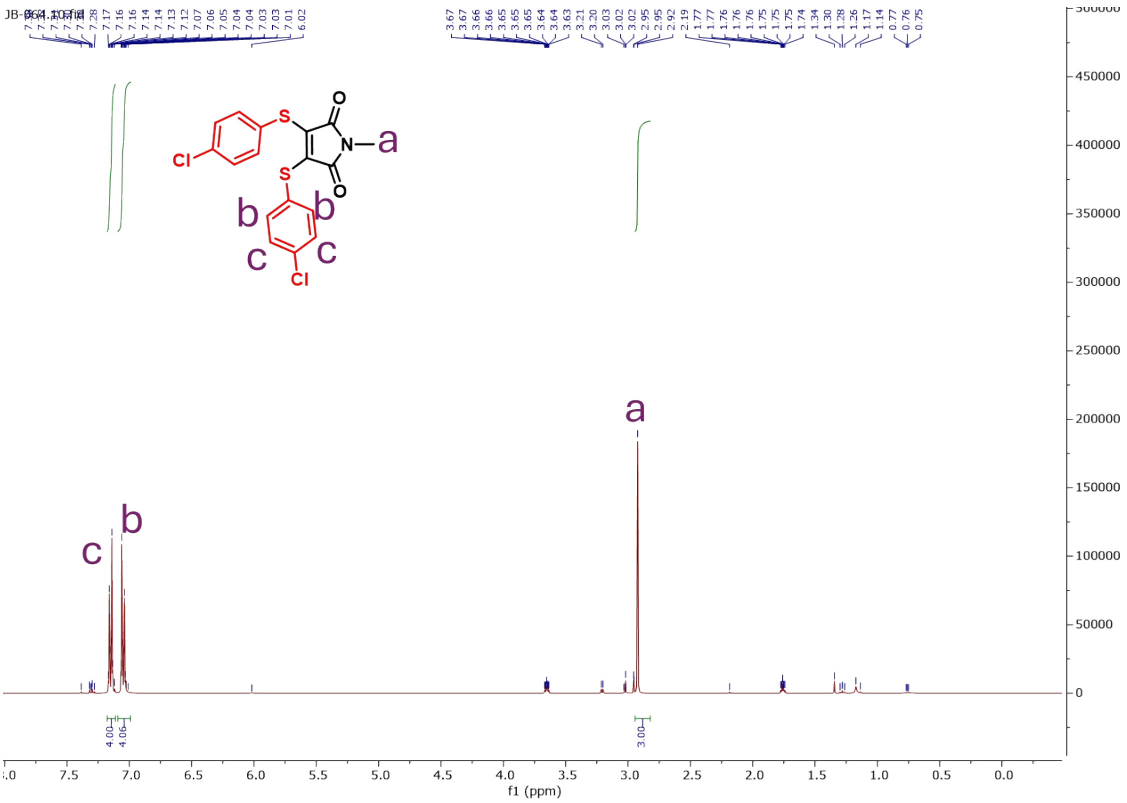


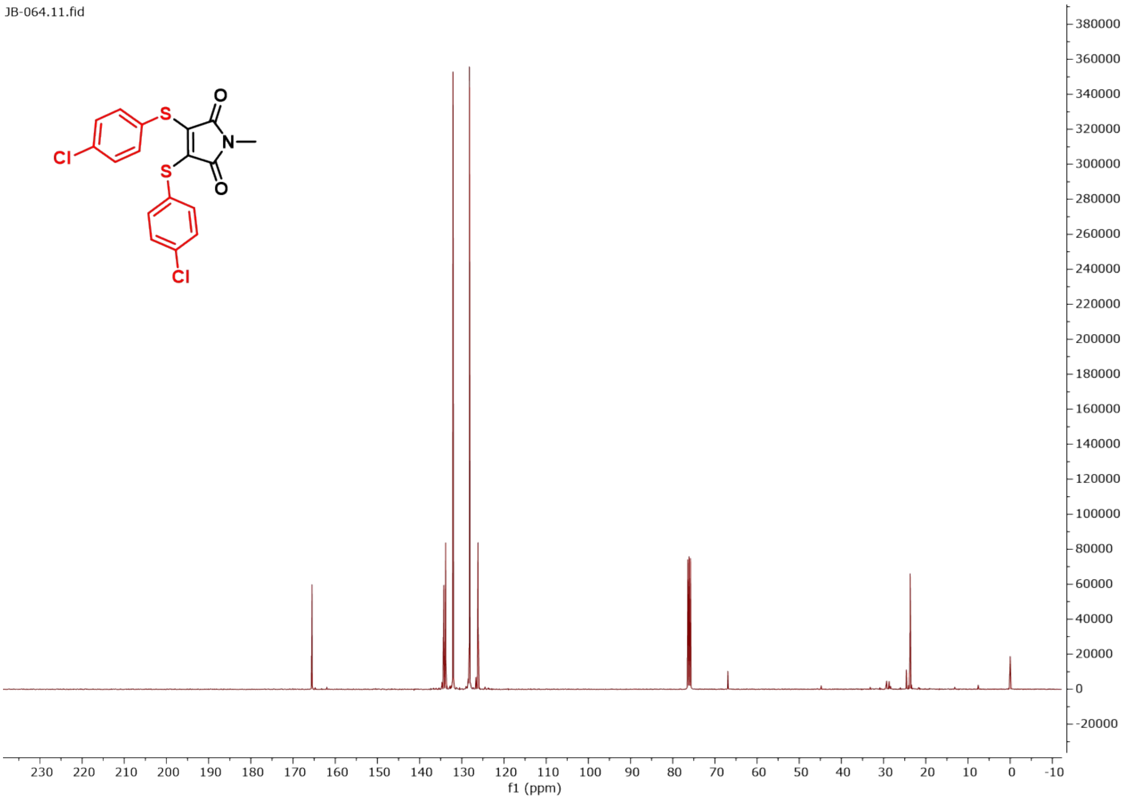


*Figure 81: $^{1}H$ and $^{13}C$ NMR Spectra of 2a*

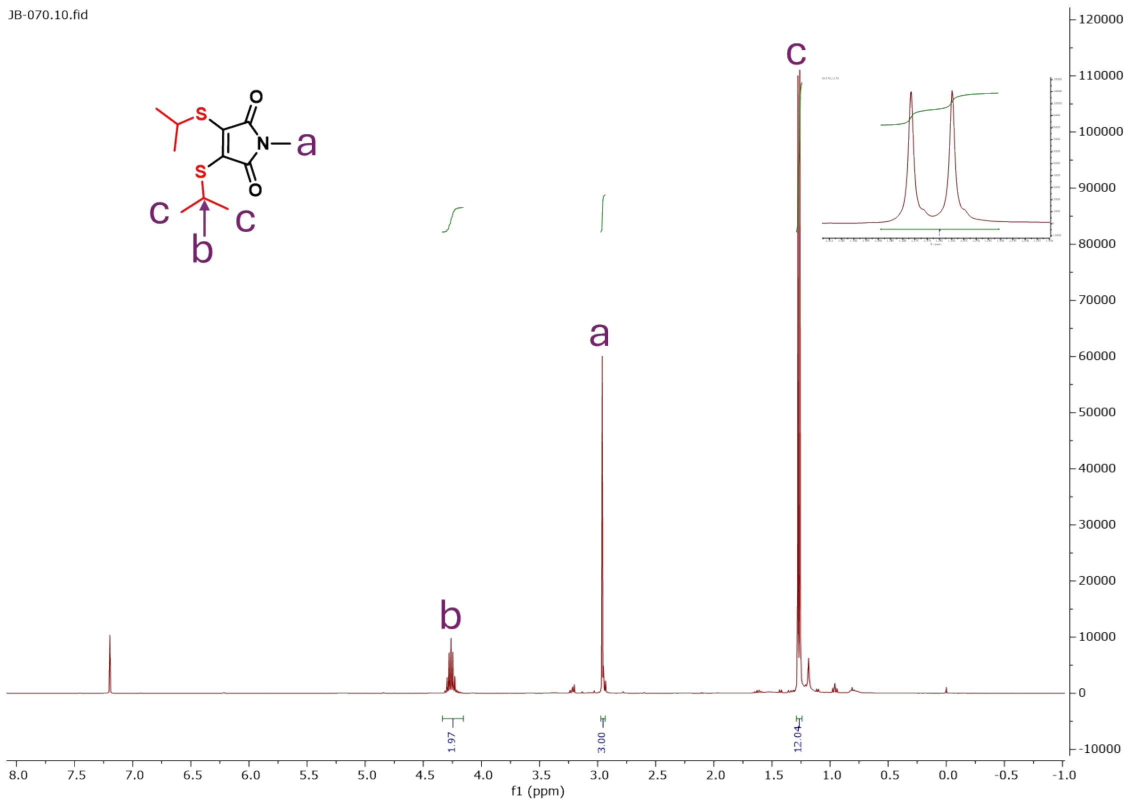


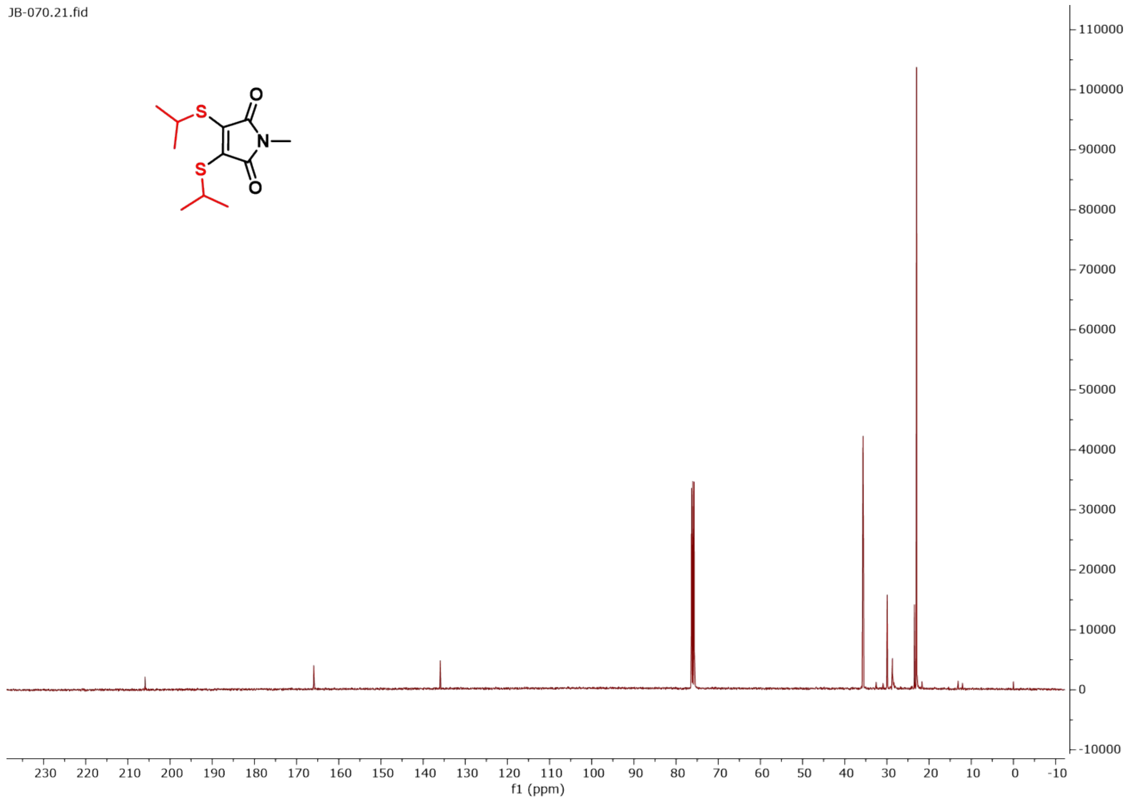


*Figure 82: $^{1}H$ and $^{13}C$ NMR Spectra of 2b*

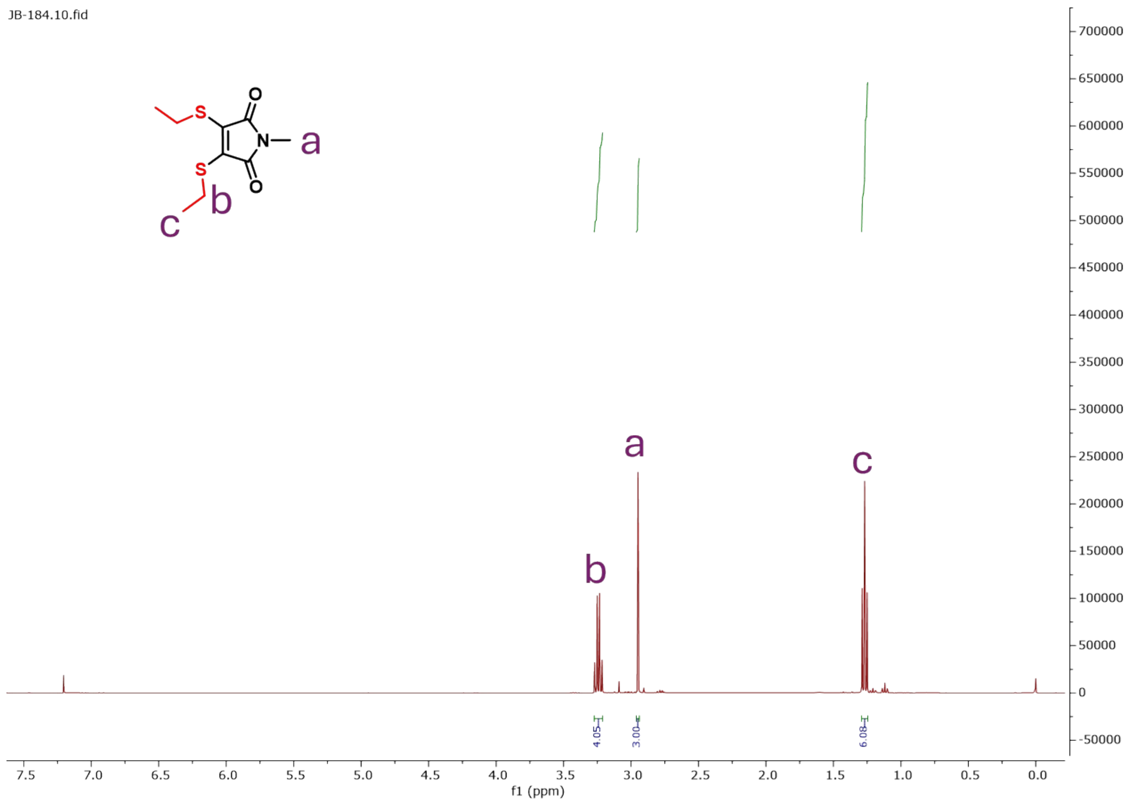


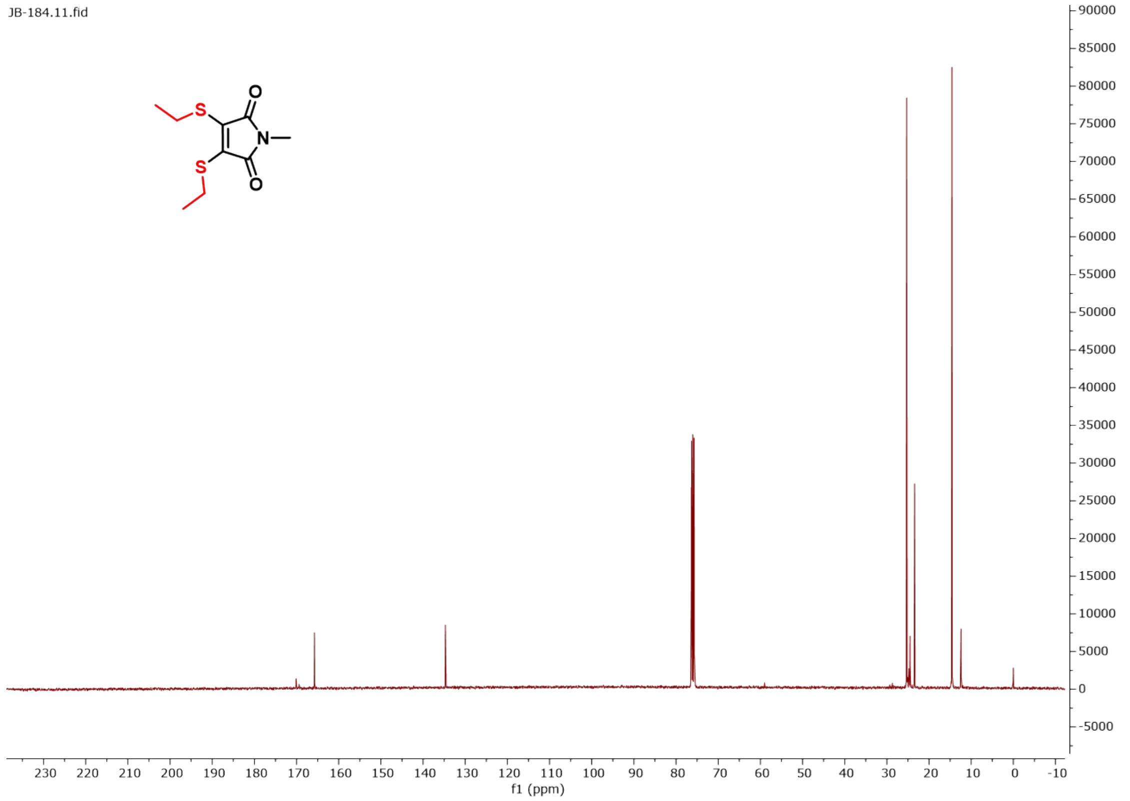


*Figure 83: $^{1}H$ and $^{13}C$ NMR Spectra of 2c*

# DTM UV-visible spectra

The UV-visible spectra for this class of maleimide derivatives were recorded and measured using the same methodology as the aminomaleimide derivatives. Initial attempts at solubilising these molecules in hexane were unsuccessful, as the samples appeared to aggregate in solution, whereas we observed that diethyl ether enabled sufficient dissolution of the sample, while remaining relatively non-polar. For DTMs, we observed a stronger secondary absorbance band around 270 nm, which was not as pronounced in the AMs previously. We theorised that this is due to the presence of the sulphur atom potentially engaging in some triplet behaviour.

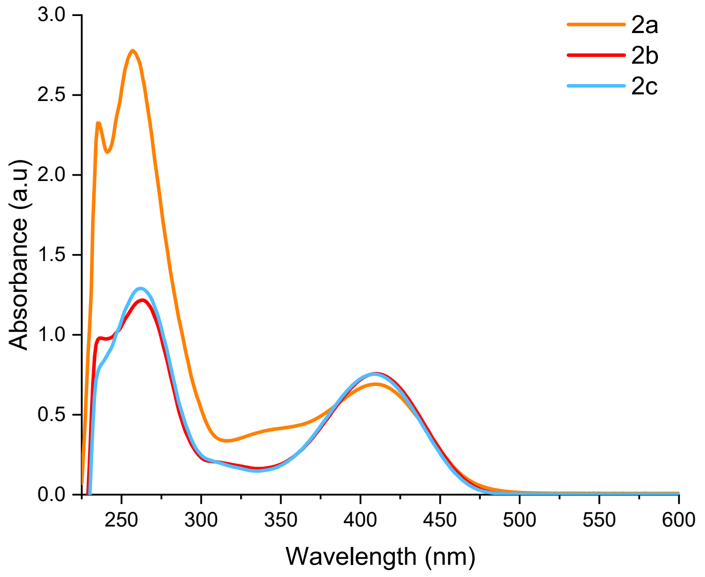


*Figure 84: Stacked UV-visible spectra of DTMs a-c*

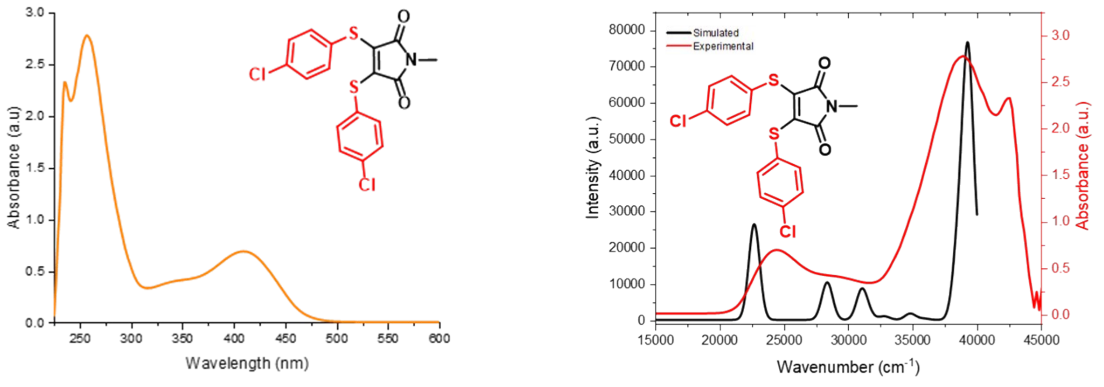


*Figure 85: Experimental (left) and simulated (right, adjusted for wavenumber) of 2a.*

*Experimental $\lambda_{max}$= 410 nm, 257 nm*

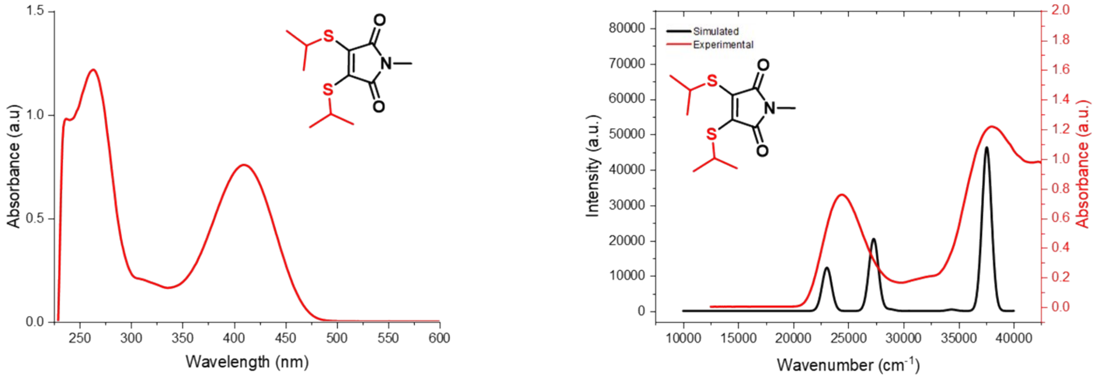


*Figure 86: Experimental (left) and simulated (right, adjusted for wavenumber) of 2b.*

*Experimental $\lambda_{max}$= 410 nm, 263 nm*

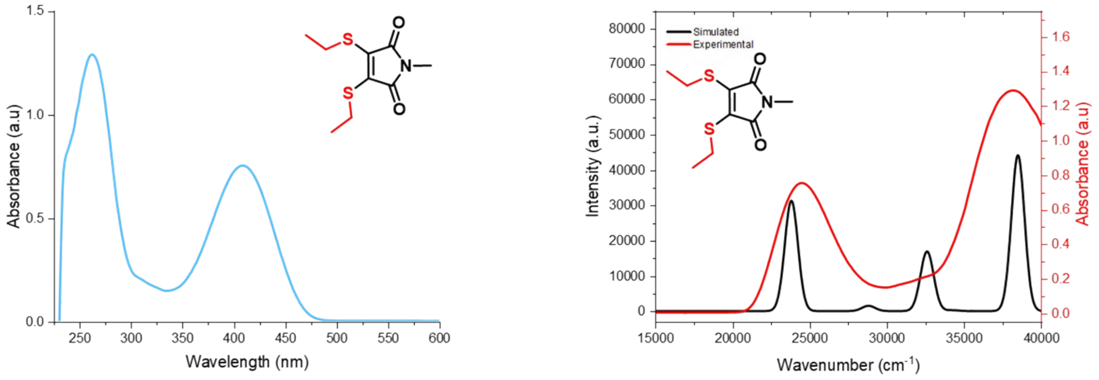


*Figure 87: Experimental (left) and simulated (right, adjusted for wavenumber) of 2c.*

*Experimental $\lambda_{max}$= 409 nm, 263 nm*

| **Molecule** | **λabs $_{max}$ (nm)** | **Absorbance (a.u.)** | **Extinction Coefficient ($M^{-1}$ $cm^{-1}$)** |
|---|---|---|---|
| **2a** | 411 | 0.6901 | 27604 |
| **2b** | 410 | 0.753 | 30120 |
| **2c** | 408 | 0.753 | 30120 |

*Table 4: DTM UV-visible characterisation summary (Samples measured at appropriate concentrations in diethyl ether)*

# DTM Fluorescence Spectra

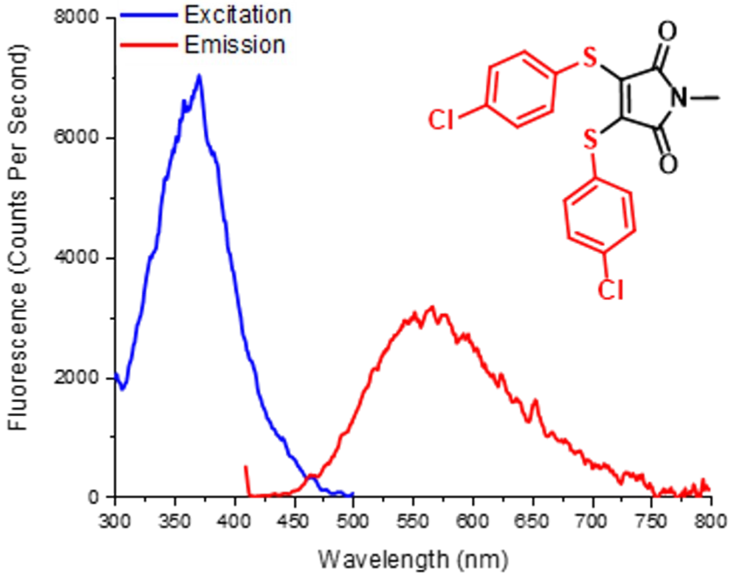


*Figure 88: Fluorescence emission spectra of 2a in diethyl ether*

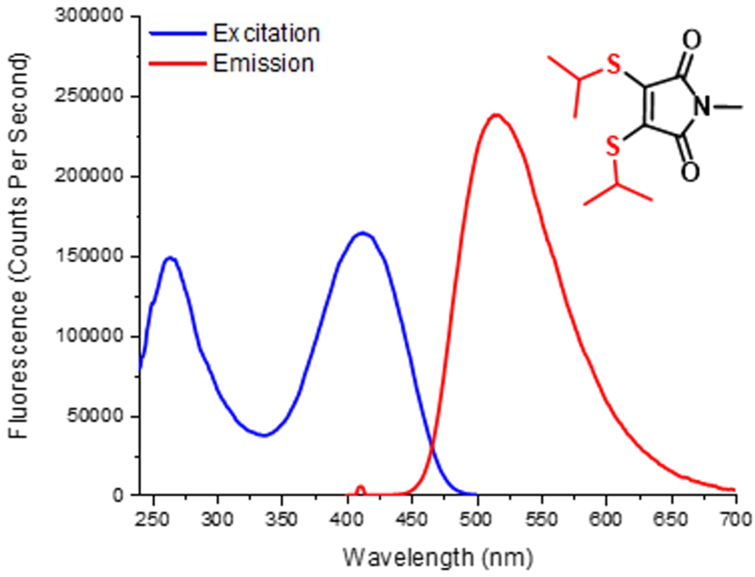


*Figure 89: Fluorescence excitation/ emission spectra of 2b in diethyl ether*

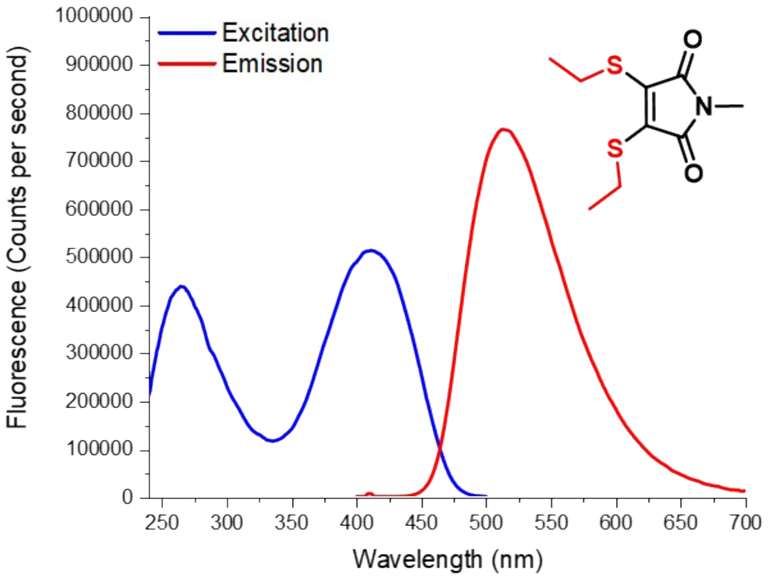


*Figure 90: Fluorescence excitation/ emission spectra of 2c in diethyl ether*

# General procedure for ATM synthesis

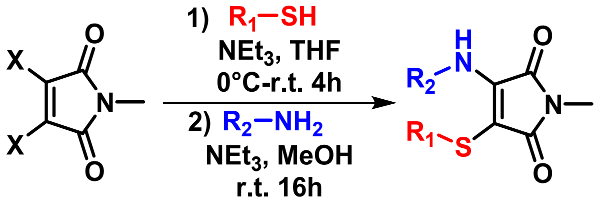


*Figure 91: General procedure for synthesis of ATMs*

A thiol was added to a THF solution of dichloro N-methylmaleimide (DCM), and the substitution was assisted using triethylamine. After four hours at room temperature, the solution was evaporated under reduced pressure, taken up in methanol, where an excess of secondary amine and triethylamine was added. The reaction was left to stir overnight at room temperature. The reaction mixture was dried under reduced pressure, and the residue was taken up in dichloromethane, and washed with ammonium chloride (10 mL), and water (3x 20 mL), before drying over magnesium sulphate and drying under vacuum to afford the corresponding ATM.

The synthetic procedure for obtaining ATMs was adapted from ***Chem. Commun., 2018, 54, 3339***

## **3a** (1-methyl-3-morpholino-4-(quinolin-2-ylthio)-1H-pyrrole-2,5-dione)

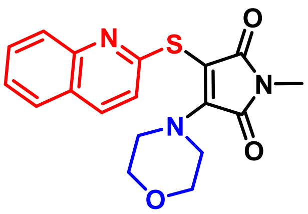

DCM (0.256 mmol, 100 mg) was dissolved in 6 mL of methanol in a 20 mL vial before the addition of 2-quinoline thiol (0.56 mmol, 90 mg) to yield a strong yellow solution. After several minutes, triethylamine (0.84 mmol, 116 µL) was added dropwise and left to stir for four hours at room temperature. An excess of morpholine (0.168 mmol, 144 µL) was added to the solution with additional triethylamine (1.68 mmol, 232µL ) and left to stir overnight at room temperature. The solution was dried under compressed air, before dilution with dichloromethane (50 mL) and washes with saturated ammonium chloride (20 mL) and distilled water (3 x 15 mL). before drying over magnesium sulphate and evaporating under reduced pressure. The resulting orange oil was recrystallised from warm petroleum spirit to yield a yellow powder in 89.8% yield (0.23 mmol, 82.5 mg).

*1H NMR (400 MHz, Chloroform-d) δ 8.06 (s, 1H), 7.99 (d, J = 8.6 Hz, 1H), 7.70 (d, J = 8.1 Hz, 1H), 7.64 (t, J = 7.6 Hz, 1H), 7.44 (t, J = 7.4 Hz, 1H), 7.24 (d, J = 8.6 Hz, 1H), 4.15 (t, J = 4.8 Hz, 4H), 3.66 (t, J = 4.7 Hz, 5H), 3.04 – 2.99 (m, 3H).*

MS (ESI) *m/z* calculated - 355, observed- 355.0986.

FTIR (cm$^{-1}$): *2989-2807 broad, 1682- sharp.*

## **3b** (3-((4-chlorophenyl)thio)-1-methyl-4-morpholino-1H-pyrrole-2,5-dione)

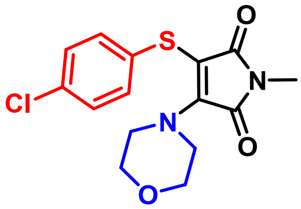


DCM (2.75 mmol, 500mg) was dissolved in THF (20 mL) to afford a pale yellow solution, before the addition of 4-chlorothiophenol (3.03 mmol, 438 mg) and dropwise addition of triethylamine (3 mmol, 0.415 mL) in an ice bath. After 2 hours, the solution appeared clumpy, and when stirring was stopped, a white precipitate was observed in the darker solution. The solvent was removed, before redissolving in an equal volume of methanol. To the orange solution was added morpholine dropwise (8.25 mmol, 0.711 mL) and stirred overnight at room temperature. The resulting solution was dried under reduced pressure for being taken up in dichloromethane, washing with saturated ammonium chloride solution (20 mL) and distilled water (3 x 20 mL), before drying over magnesium sulphate, and drying under a vacuum. The resulting oil was purified *via* column chromatography (1:9 to 2.5: 7.5 ethyl acetate to hexane) to yield the final product in 3.53% yield (0.097 mmol, 33 mg).

*1H NMR (400 MHz, Chloroform-d) δ 7.21 – 7.12 (m, 2H), 7.06 – 6.98 (m, 2H), 4.12 (dd, J = 5.4, 4.2 Hz, 3H), 3.68 – 3.60 (m, 4H), 2.97 (d, J = 1.1 Hz, 3H), 2.10 (d, J = 1.0 Hz, 3H).*

*13C NMR (101 MHz, Chloroform-d) δ 206.96, 169.84,166.14, 149.68, 135.71, 131.76, 129.33, 127.46, 67.08, 48.57, 30.93, 24.40.*

MS (ESI) *m/z* calculated - 339, observed- 338.0488.

FTIR ($cm^{-1}$): *2984- 2777 broad, 1682- sharp, 1574- sharp.*

## **3c** (3-(ethylthio)-1-methyl-4-morpholino-1H-pyrrole-2,5-dione)

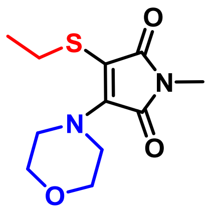

DCM (2.75 mmol, 500mg) was dissolved in THF (20 mL) to afford a pale yellow solution, before the addition of ethanethiol (6.05 mmol, 0.435 mL) and triethylamine (3 mmol, 0.415 mL) in an ice bath. The solution was left to stir at room temperature for four hours and monitored *via* TLC. The solution was then dried under compressed air, and redissolved in methanol (20 mL). Morpholine (13.75 mmol, 1.185 mL) was added dropwise in large excess and left to stir overnight. An orange solution was observed with minor white precipitate observed. This solution was dried and redissolved dichloromethane (100 mL) and washed with ammonium chloride (50 mL), distilled water (2 x 40 mL) before drying over magnesium sulphate and evaporating under reduced pressure. The resulting oil was purified *via* column chromatography (1:9 to 3:7 ethyl acetate to hexane) to yield the final product as an orange oil in 18.2% yield (34.6 mg, 0.135 mmol).

*1H NMR (400 MHz, Chloroform-d) δ 4.16 – 4.09 (m, 2H), 3.94 – 3.87 (m, 1H), 3.76 – 3.69 (m, 3H), 2.92 (s, 2H), 2.66 (q, J = 7.4 Hz, 1H), 1.17 (t, J = 7.4 Hz, 1H).*

*13C NMR (101 MHz, Chloroform-d) δ 170.29, 166.79, 165.82, 148.44, 141.00, 95.14, 93.81, 67.01, 48.62, 48.26, 29.90, 24.10, 14.73.*

MS (ESI) *m/z* calculated - 256, observed- 256.0876.

FTIR ($cm^{-1}$): *3031-2787 broad, 1692- sharp, 1584- sharp.*

# ATM NMR Spectra

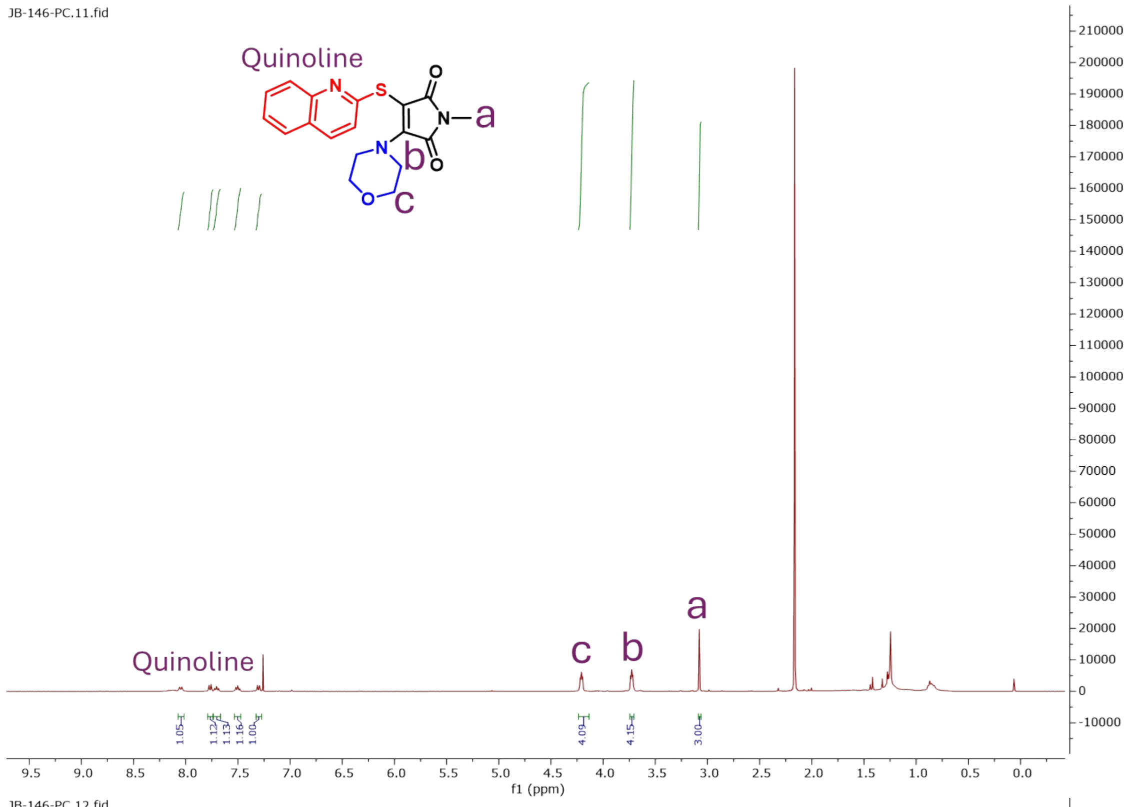


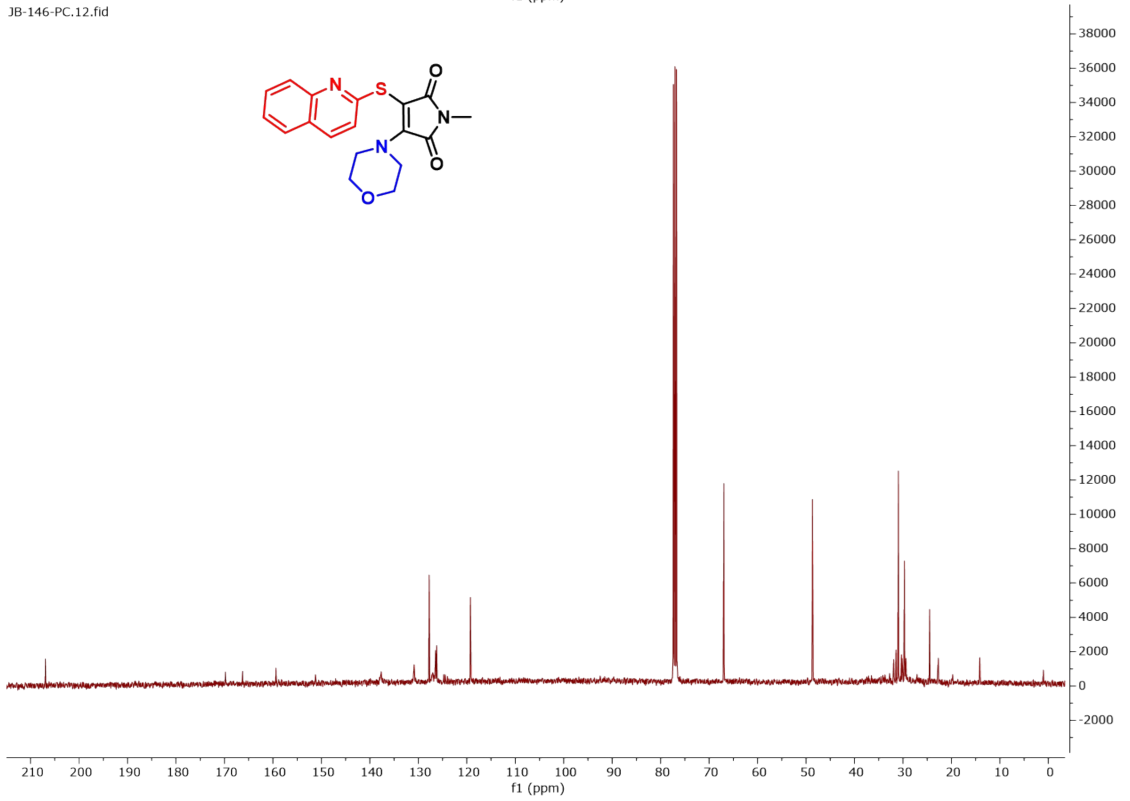


*Figure 92: $^{1}H$ and $^{13}C$ NMR Spectra of 3a*

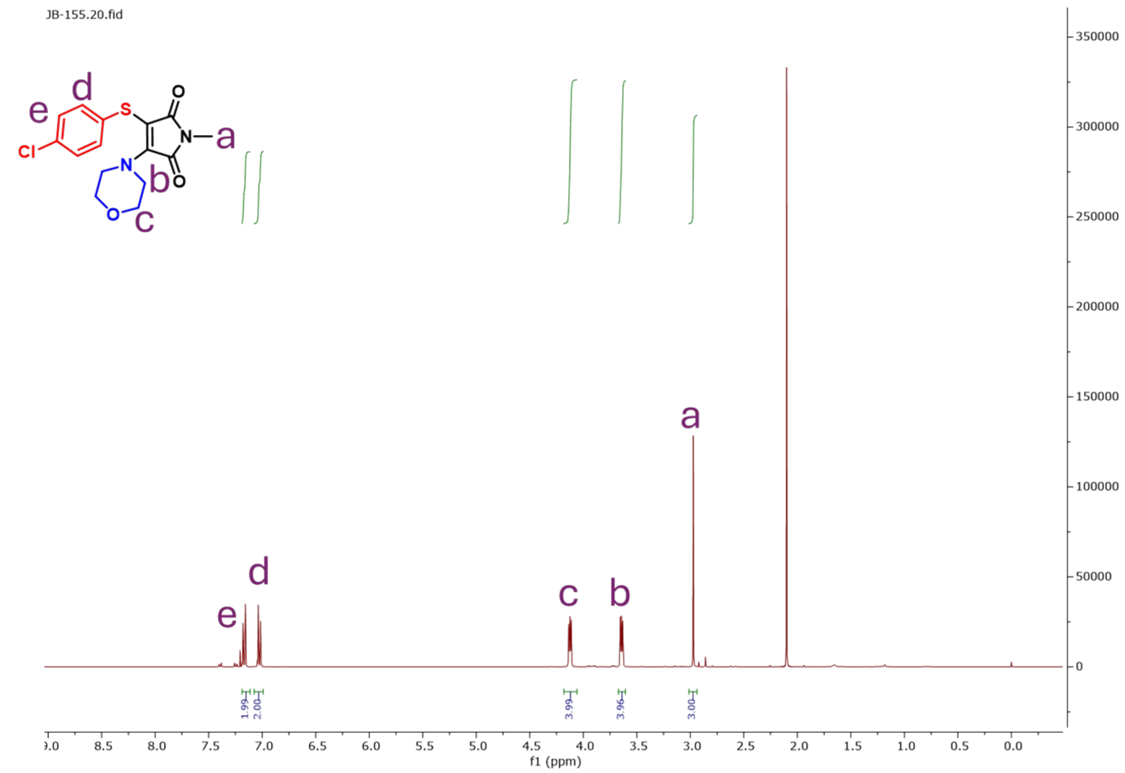


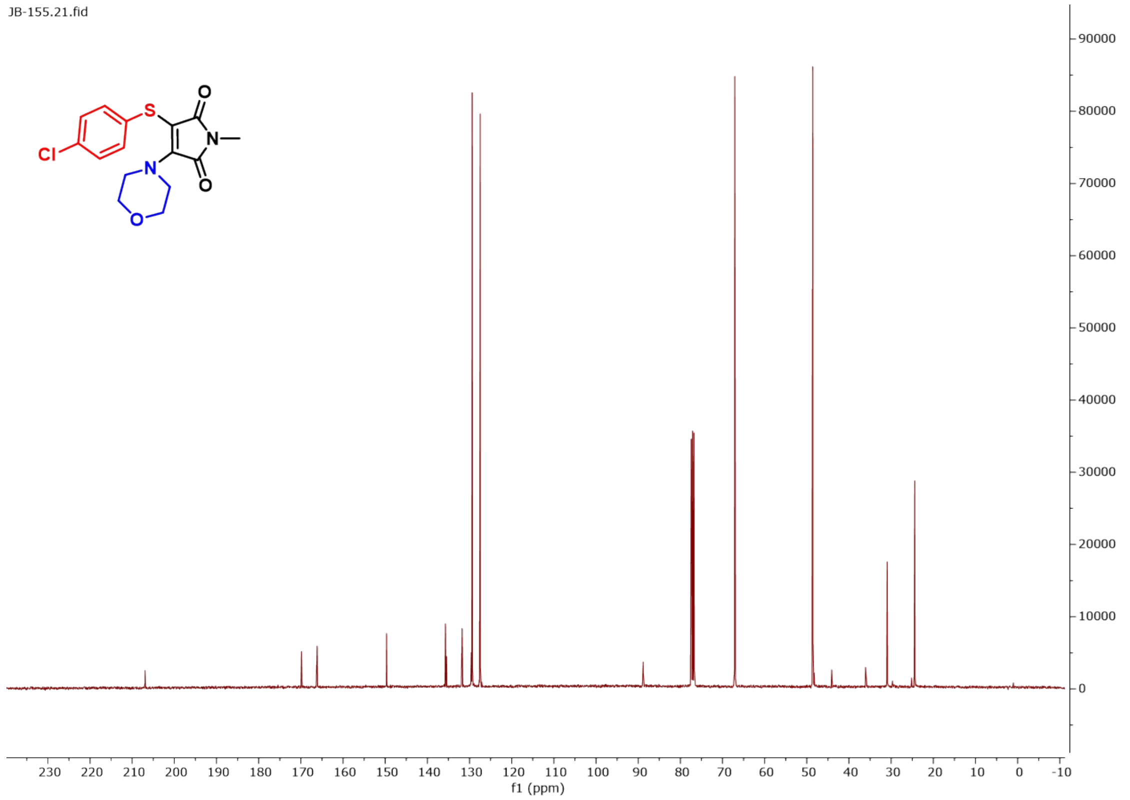


*Figure 93: $^{1}H$ and $^{13}C$ NMR Spectra of 3b*

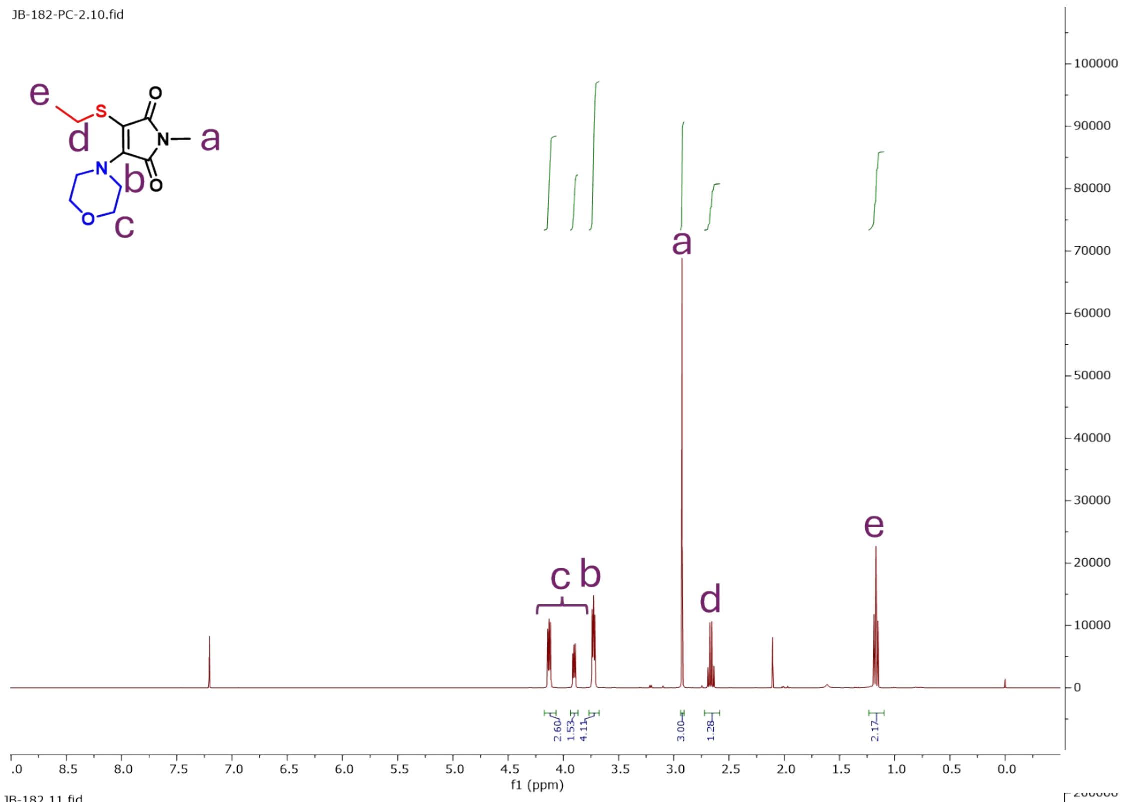


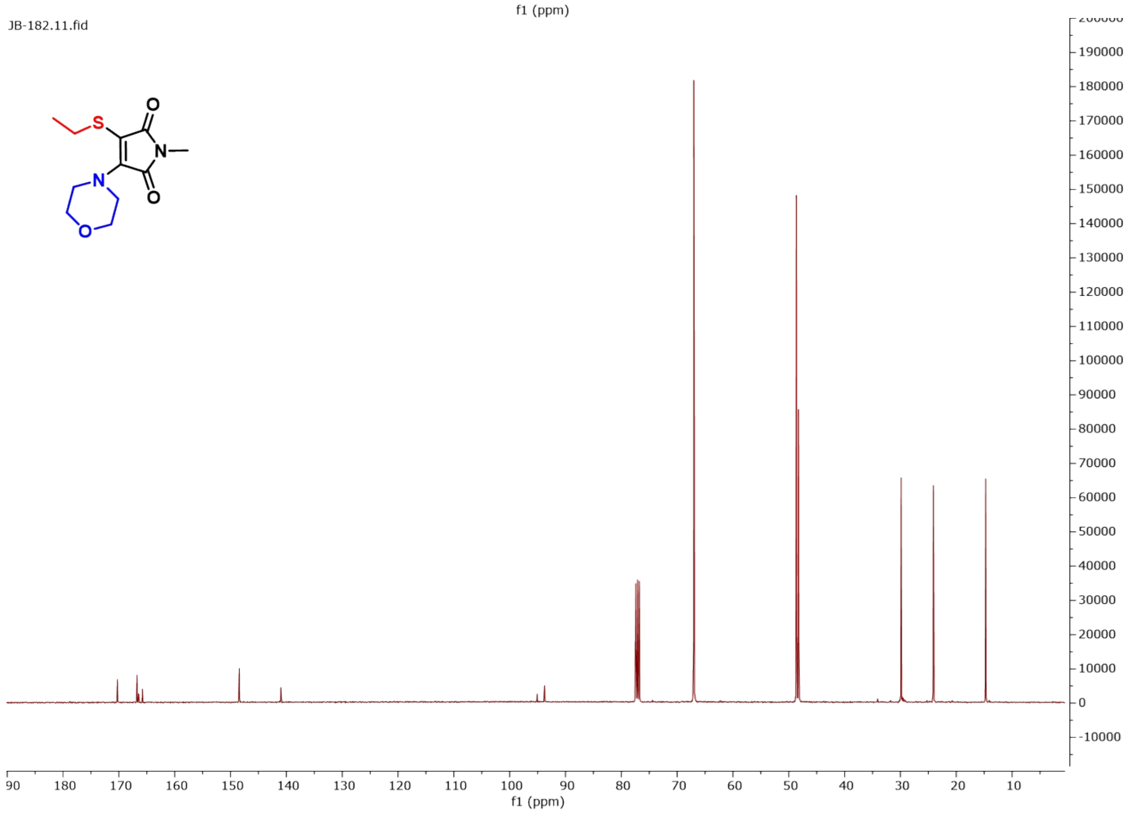


*Figure 94: $^{1}H$ and $^{13}C$ NMR Spectra of 3c*

# ATM UV-visible spectra

The UV-visible spectra for mixed amino-thiol maleimide derivatives were recorded in the same way as the previous maleimide derivatives. Once more, our initial attempts at solubilising these molecules in hexane were unsuccessful, as these samples also appeared to aggregate in solution, whereas diethyl ether enabled sufficient dissolution of sample.

As with the DTMs, we also observed an additional band around 250 nm, which we also attribute to the thiol motif. Molecule *3a* additionally features a band at approximately 325 nm, which we theorised is linked to the quinoline moiety in this molecule.

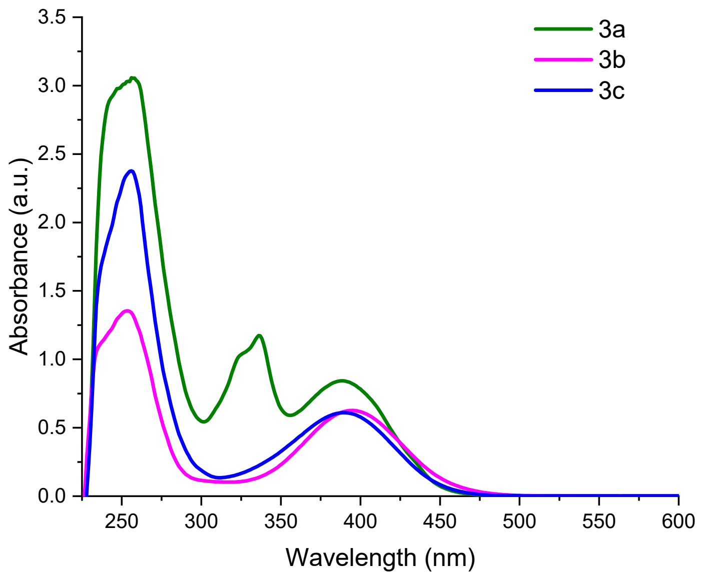


*Figure 95: Stacked UV-visible spectra of ATMs a-c*

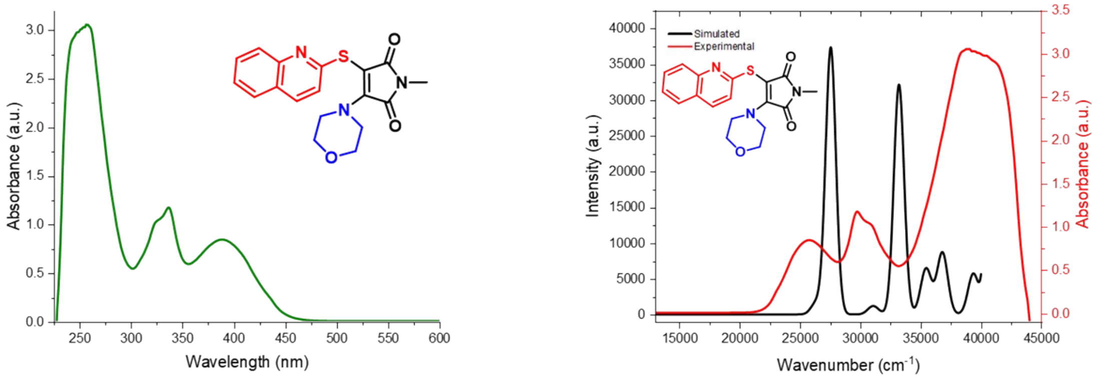


*Figure 96: Experimental (left) and simulated (right, adjusted for wavenumber) of 3a.*

*Experimental $\lambda_{max}$= 388 nm, 336 nm, 256 nm*

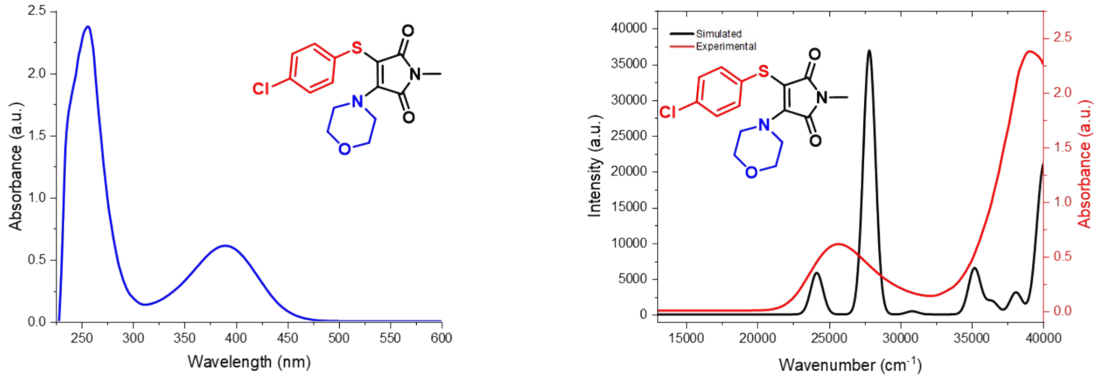


*Figure 97: Experimental (left) and simulated (right, adjusted for wavenumber) of 3b.*

*Experimental $\lambda_{max}$= 390 nm, 256 nm*

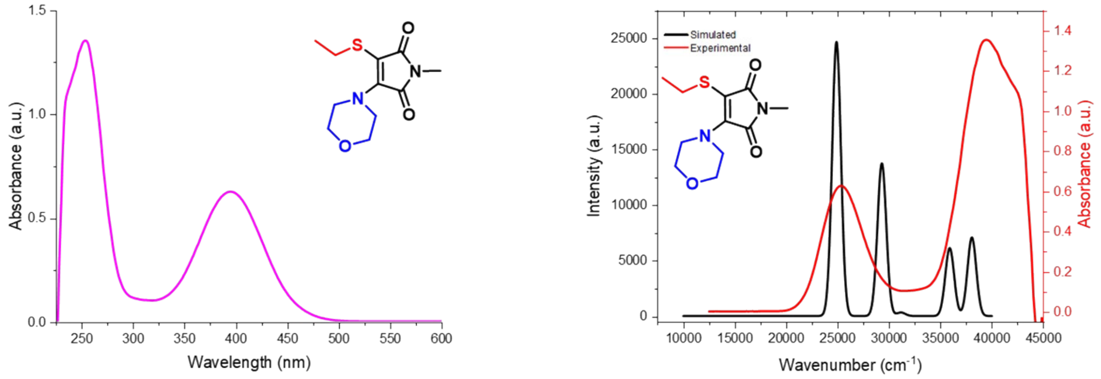


*Figure 98: Experimental (left) and simulated (right, adjusted for wavenumber) of 3c.*

*Experimental $\lambda_{max}$= 396 nm, 253 nm*

| **Molecule** | **λabs $_{max}$ (nm)** | **Absorbance (a.u.)** | **Extinction Coefficient ($M^{-1}$ $cm^{-1}$)** |
|---|---|---|---|
| **3a** | 388 | 0.843 | 16860 |
| **3b** | 390 | 0.619 | 49520 |
| **3c** | 395 | 0.626 | 50080 |

*Table 5: ATM UV-visible characterisation summary (Samples measured at appropriate concentrations in diethyl ether)*

# ATM Fluorescence Spectra

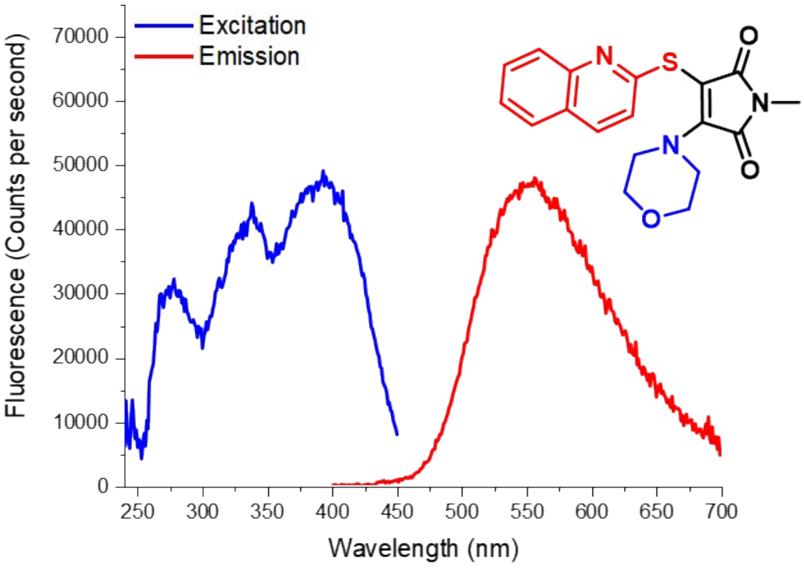


*Figure 99: Fluorescence emission spectra of 3a in diethyl ether*

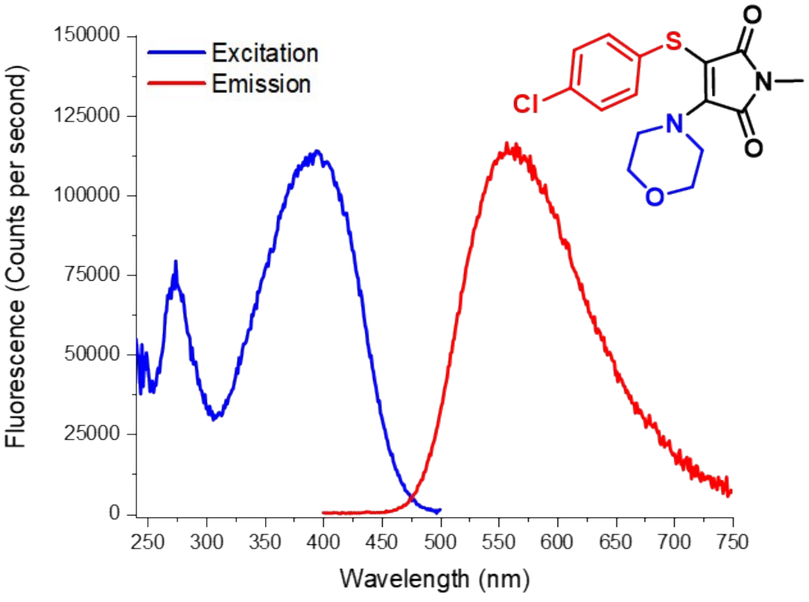


*Figure 100: Fluorescence emission spectra of 3b in diethyl ether*

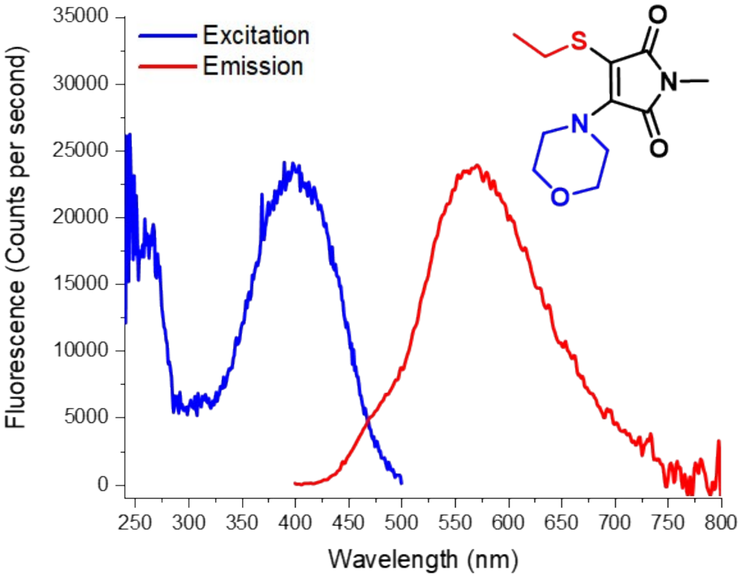


*Figure 101: Fluorescence emission spectra of 3c in diethyl ether*

## Photophysical Summary:

We include a table of all molecules synthesised, along with their most important photophysical properties relating to this work.

| Molecule | Code | Diethyl Ether (nm) | | | | | |
|---|---|---|---|---|---|---|---|
| | | Abs | Ex | Em | **ANN (Ex)** | TD-DFT | **ANN (Em)** |
| 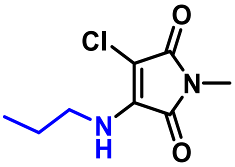 | **1a** | 371 | 372 | 480 | **362.6** | 361.9 | **479.34** |
| 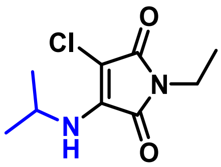 | **1b** | 370 | 370 | 478 | **371.7** | 372.4 | **465.01** |
| 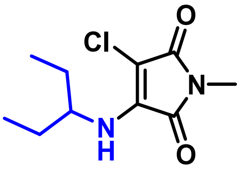 | **1c** | 370 | 371 | 481 | **363.2** | 362.9 | **488.244** |
| 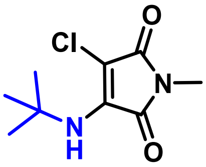 | **1d** | 364 | 365 | 508 | **363.9** | 345.7 | **485.4** |
| 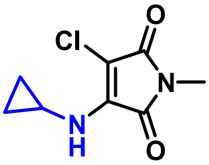 | **1e** | 374 | 374 | 483 | **365.9** | 351.3 | **483** |
| 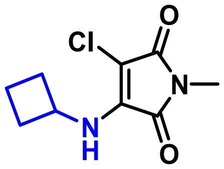 | **1f** | 373 | 374 | 481 | **367.8** | 350.6 | **482.1** |
| 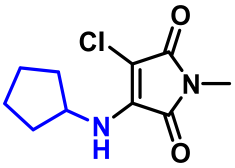 | **1g** | 372 | 373 | 483 | **361.1** | 350.2 | **477.4** |
| 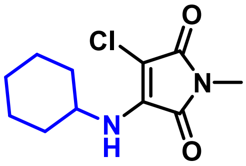 | **1h** | 371 | 371 | 481 | **363.7** | 350 | **472** |

| | | | | | | | |
|---|---|---|---|---|---|---|---|
| 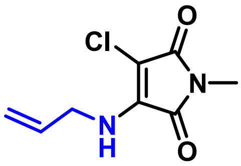 | **1i** | 370 | 369 | 480 | **364** | 362.3 | **488.19** |
| 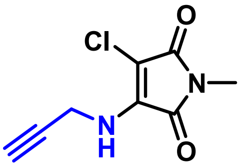 | **1j** | 365 | 371 | 480 | **367.8** | 360.7 | **498.71** |
| 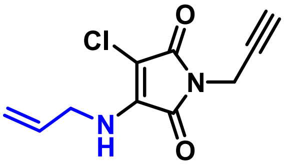 | **1k** | 367 | 369 | 471 | **372.8** | 357.6 | **473.3** |
| 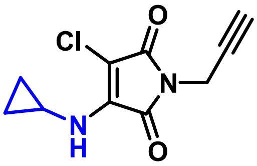 | **1l** | 369 | 368 | 476 | **368.3** | 351.8 | **481.8** |
| 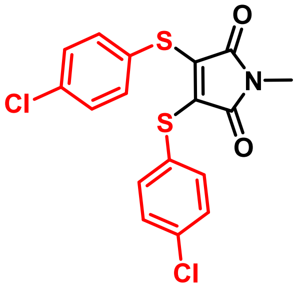 | **2a** | 411 | 371 | 565 | **428.2** | 377.9 | **528.3** |
| 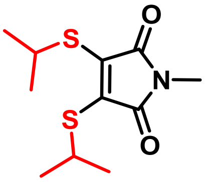 | **2b** | 410 | 413 | 516 | **413.6** | 381.5 | **518.4** |
| 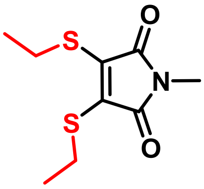 | **2c** | 408 | 410 | 513 | **406.4** | 368.5 | **507.3** |
| 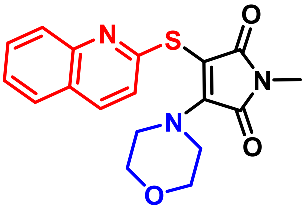 | **3a** | 388 | 390 | 556 | **352.2** | 378.5 | **500.8** |

| | | | | | | | |
|---|---|---|---|---|---|---|---|
| 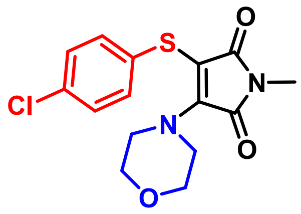 | **3b** | 390 | 395 | 562 | **364.2** | 408 | **517.3** |
| 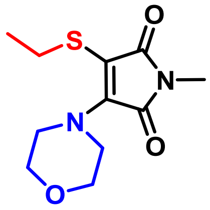 | **3c** | 395 | 398 | 571 | **386.6** | 409 | **537.9** |

*Table 6: UV-visible/ Fluorescence properties summary (n.b. ANN values correspond to diethyl ether, and TD-DFT values were calculated in vacuo)*

# CIE Diagram- Combined

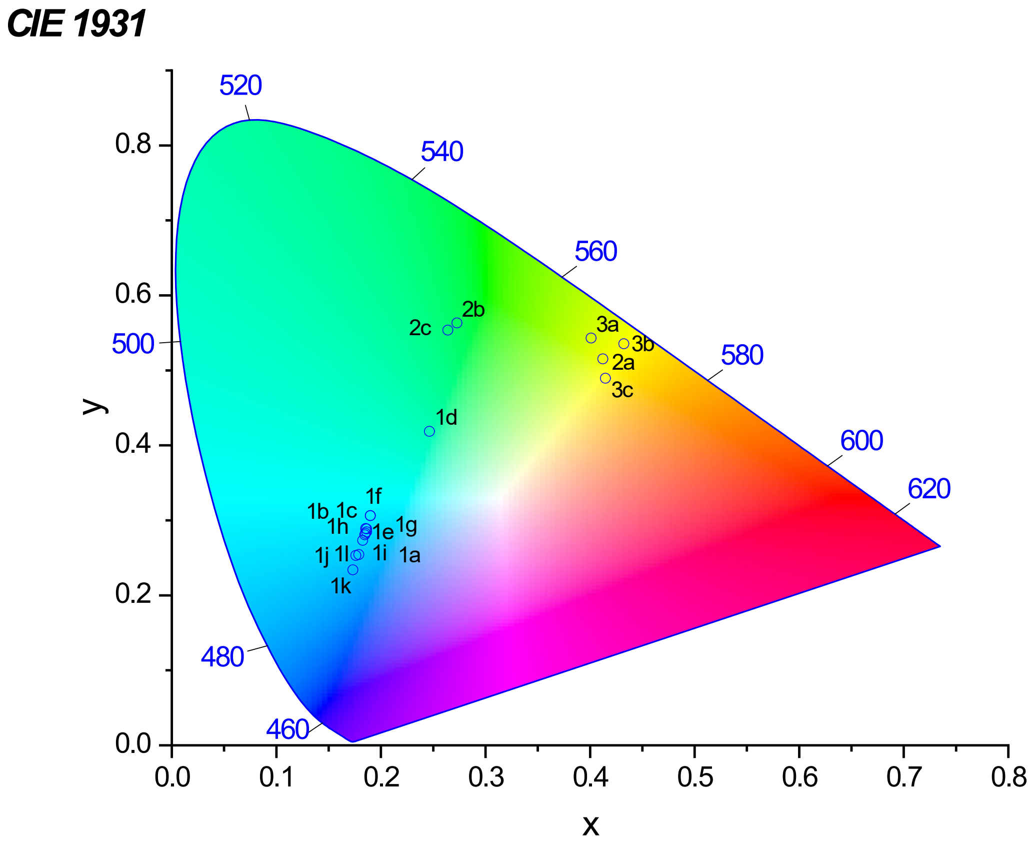


*Figure 102: CIE 1931 Chromaticity Diagram of all synthesised maleimide derivatives in this work.*

# HOMO-LUMO Projections

Using Avogadro, we visualised the Kohn-Sham orbital projections of the HOMO, HOMO -1, LUMO and LUMO +1 along with their energy levels, which we obtained from our TD-DFT calculations computed with ORCA. This indicated the electron distribution present in the maleimide derivatives and allows for a greater understanding of photophysical properties when linked to structure.

## HOMO-LUMO Projections- AM

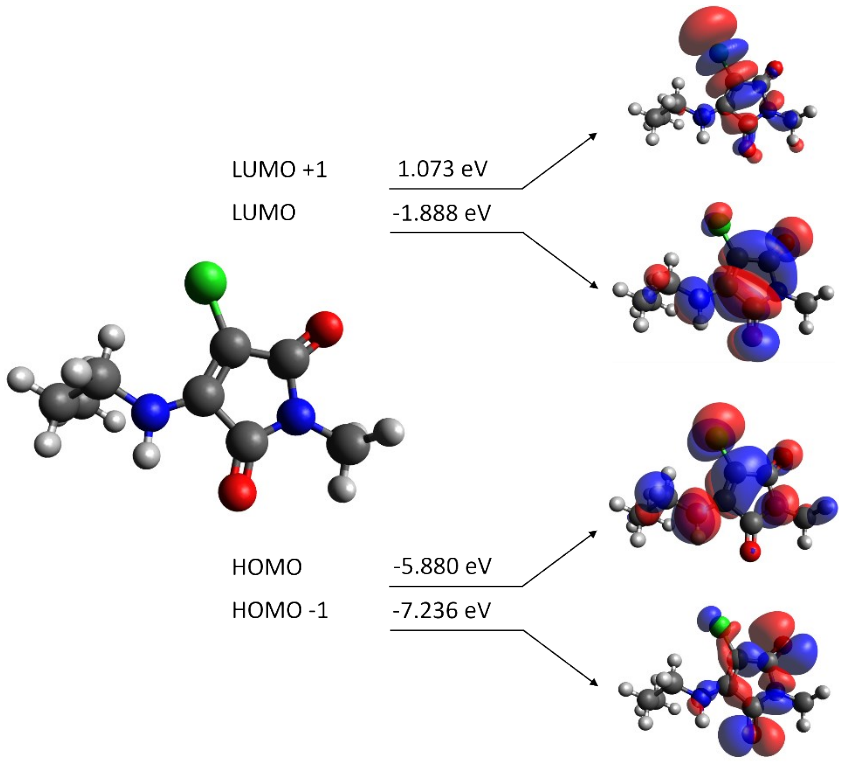


*Figure 103: HOMO-1, HOMO, LUMO and LUMO +1 projection of 1a, plus corresponding energies (eV).*

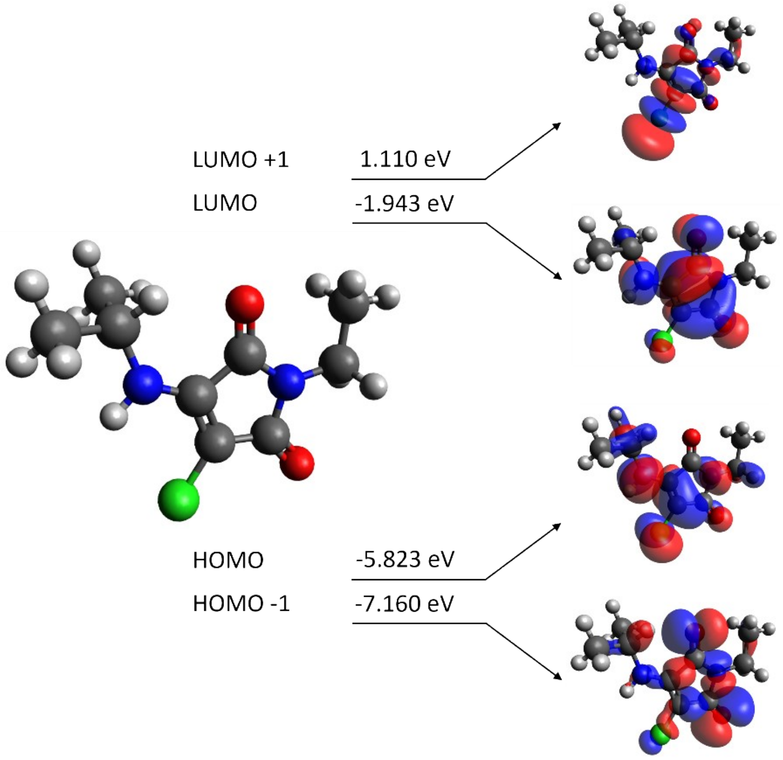


*Figure 104: HOMO-1, HOMO, LUMO and LUMO +1 projection of 1b, plus corresponding energies (eV).*

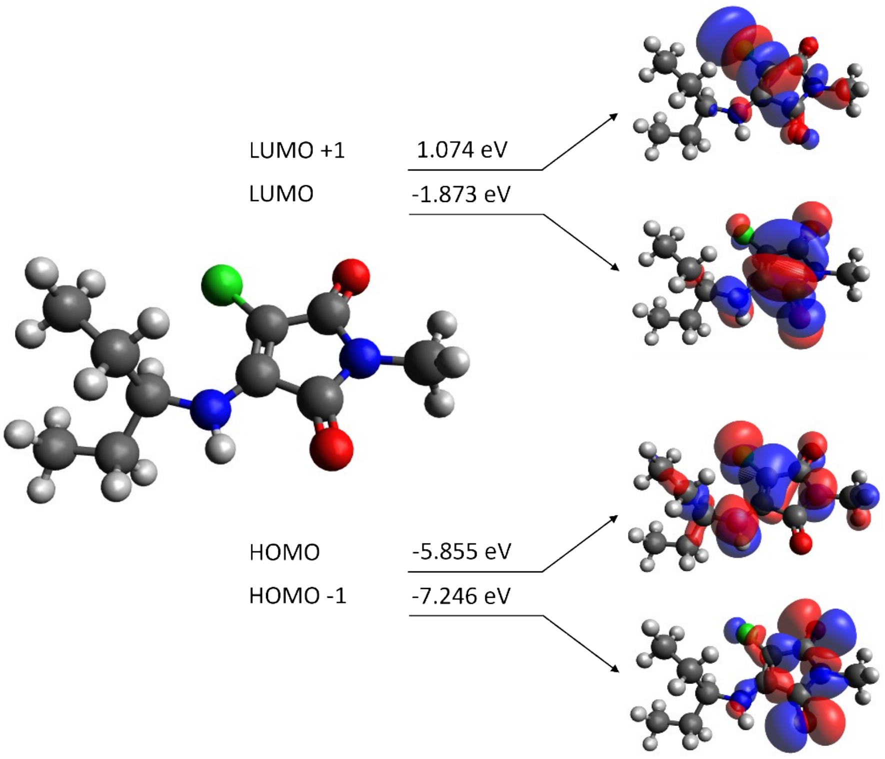


*Figure 105: HOMO-1, HOMO, LUMO and LUMO +1 projection of 1c, plus corresponding energies (eV).*

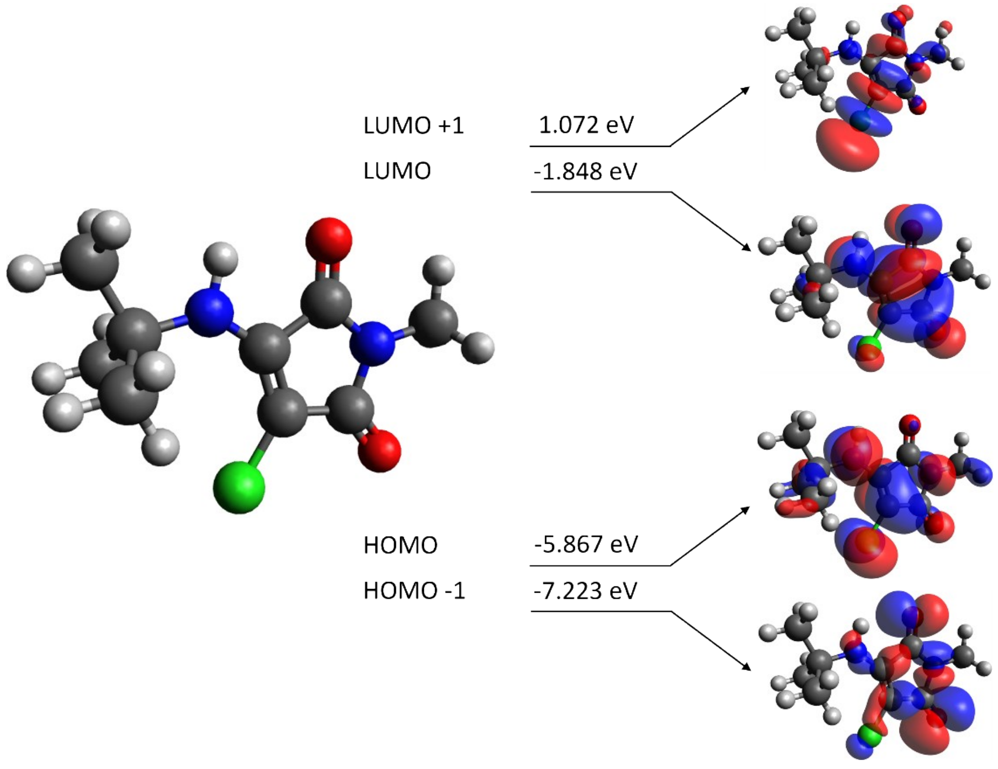


*Figure 106: HOMO-1, HOMO, LUMO and LUMO +1 projection of 1d, plus corresponding energies (eV).*

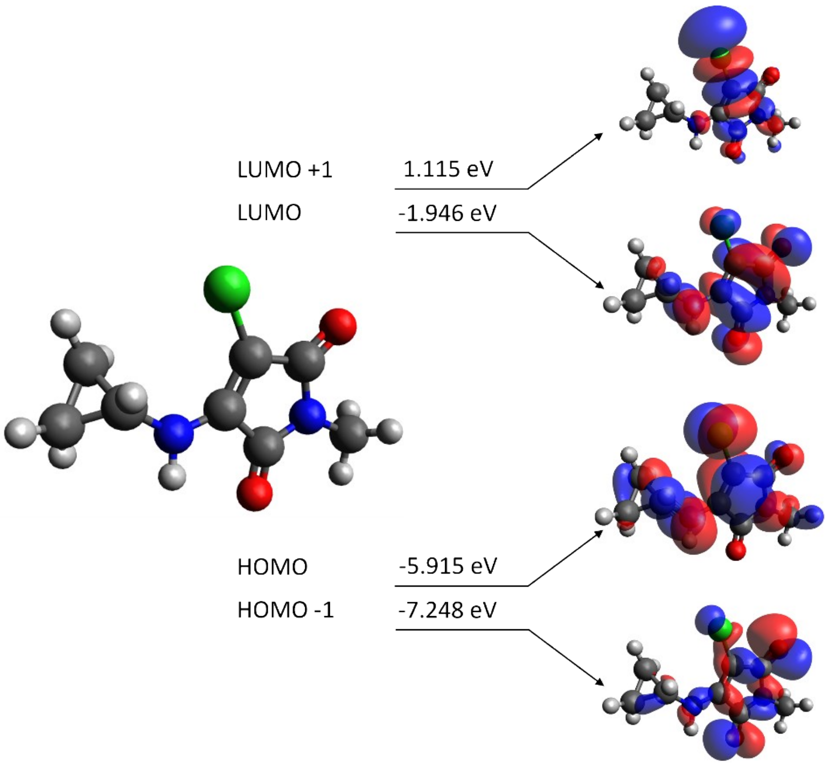


*Figure 107: HOMO-1, HOMO, LUMO and LUMO +1 projection of 1e, plus corresponding energies (eV).*

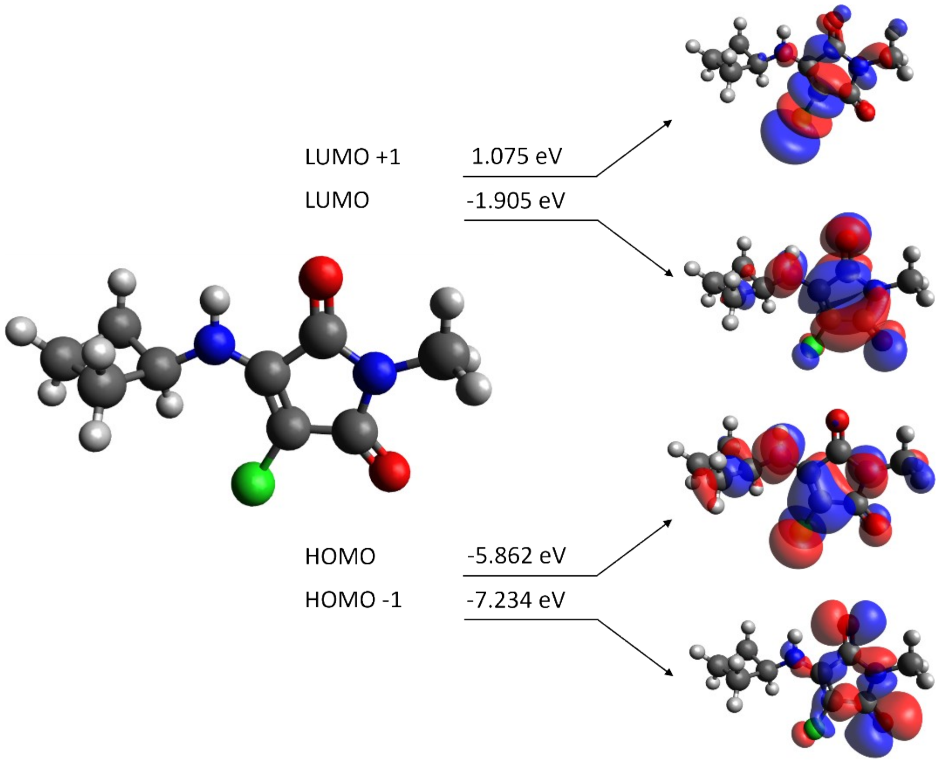


*Figure 108: HOMO-1, HOMO, LUMO and LUMO +1 projection of 1f, plus corresponding energies (eV).*

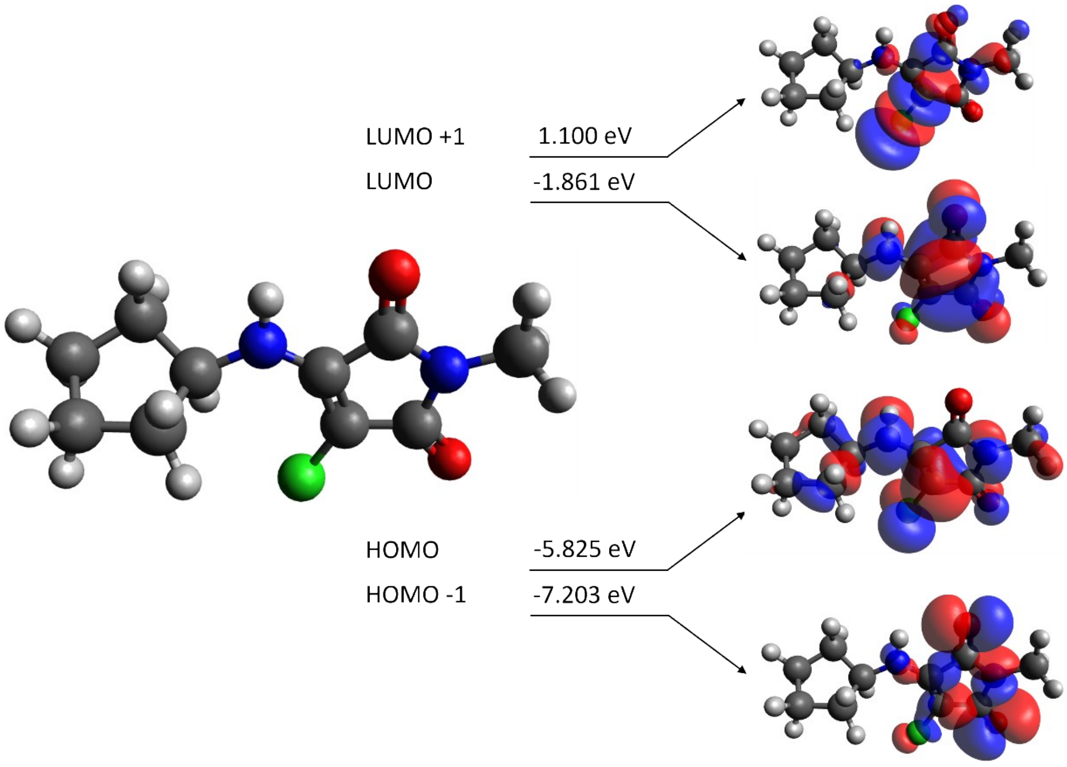


*Figure 109: HOMO-1, HOMO, LUMO and LUMO +1 projection of 1g, plus corresponding energies (eV).*

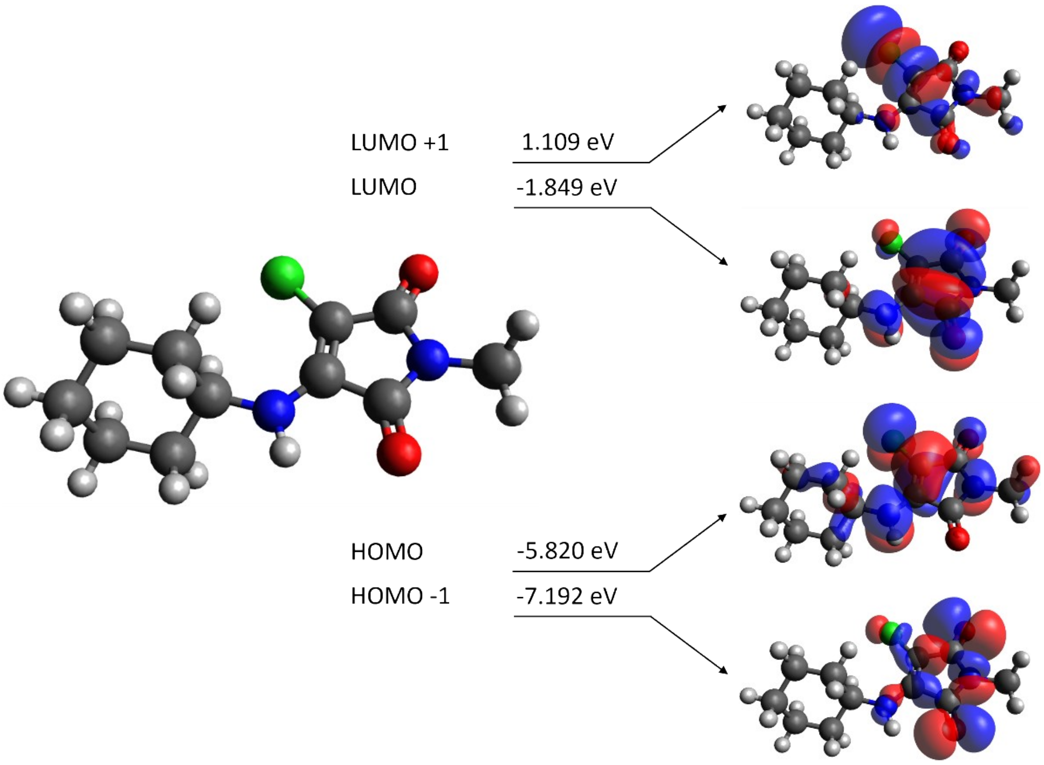


*Figure 110: HOMO-1, HOMO, LUMO and LUMO +1 projection of 1h, plus corresponding energies (eV).*

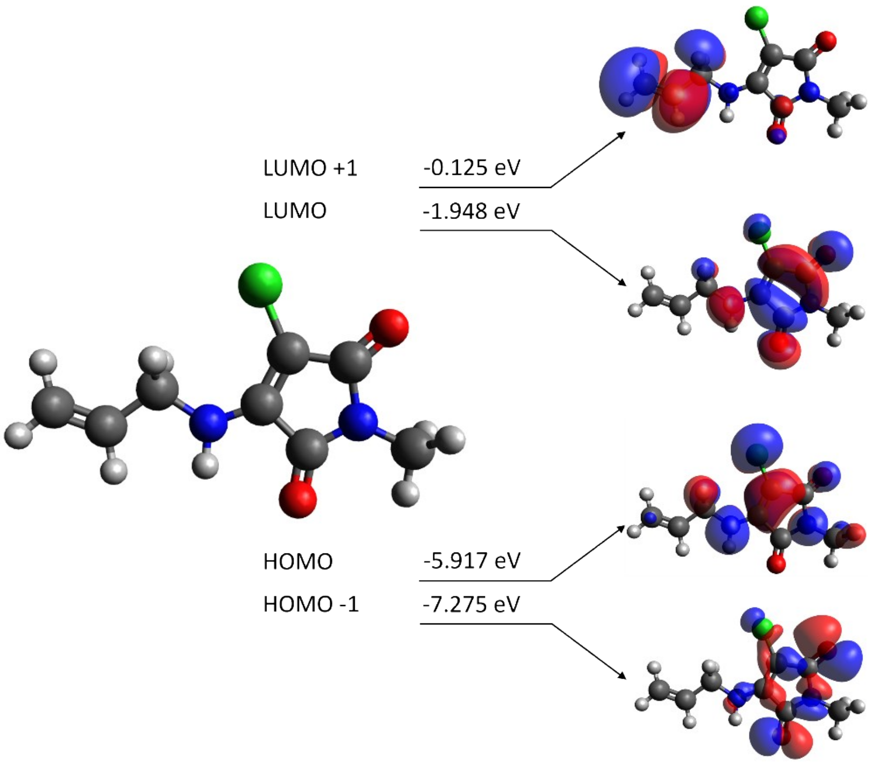


*Figure 111: HOMO-1, HOMO, LUMO and LUMO +1 projection of 1i, plus corresponding energies (eV).*

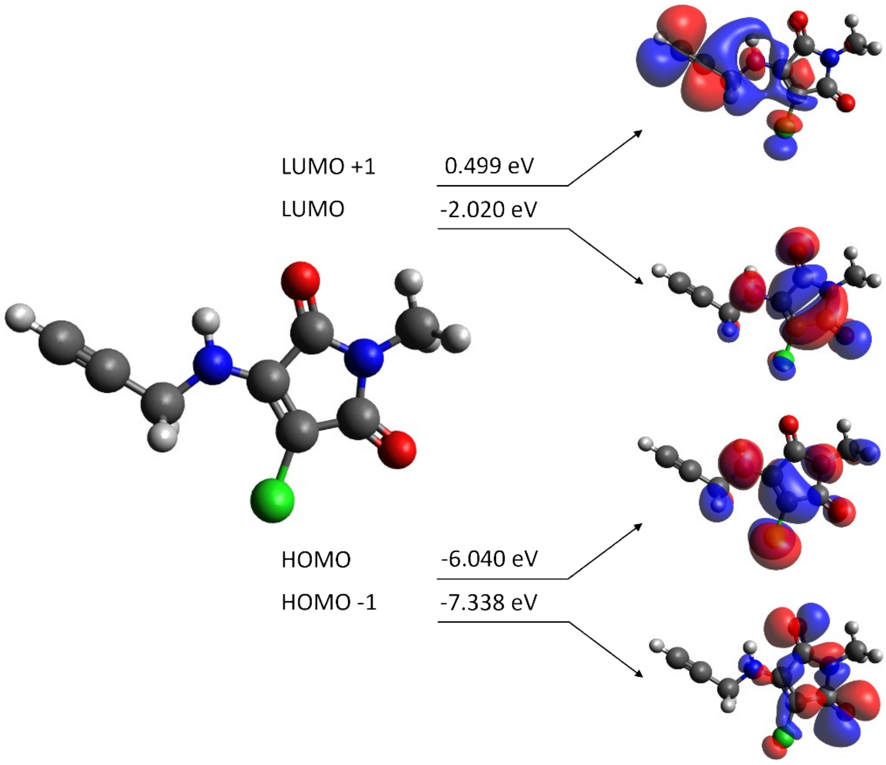


*Figure 112: HOMO-1, HOMO, LUMO and LUMO +1 projection of 1j, plus corresponding energies (eV).*

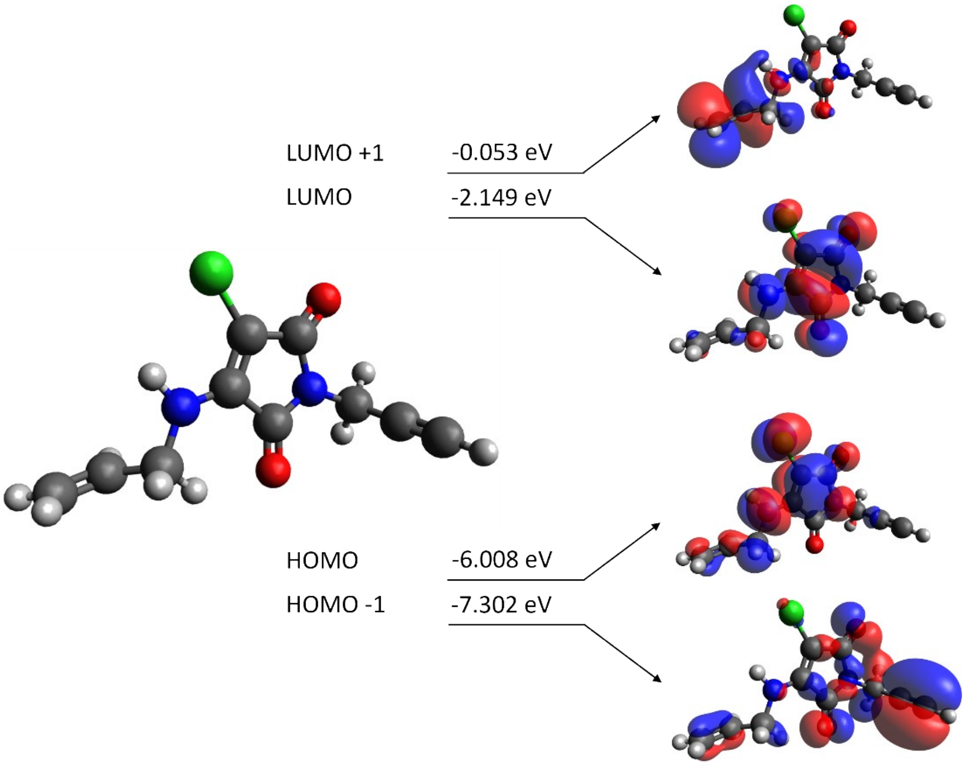


*Figure 113: HOMO-1, HOMO, LUMO and LUMO +1 projection of 1k, plus corresponding energies (eV).*

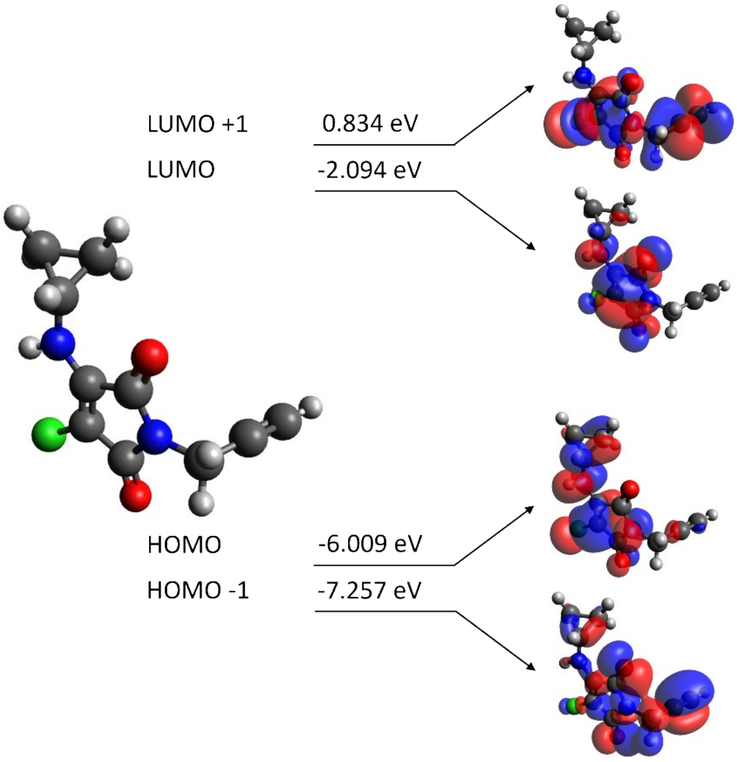


*Figure 114: HOMO-1, HOMO, LUMO and LUMO +1 projection of 1l, plus corresponding energies (eV).*

## HOMO-LUMO Projections- DTM

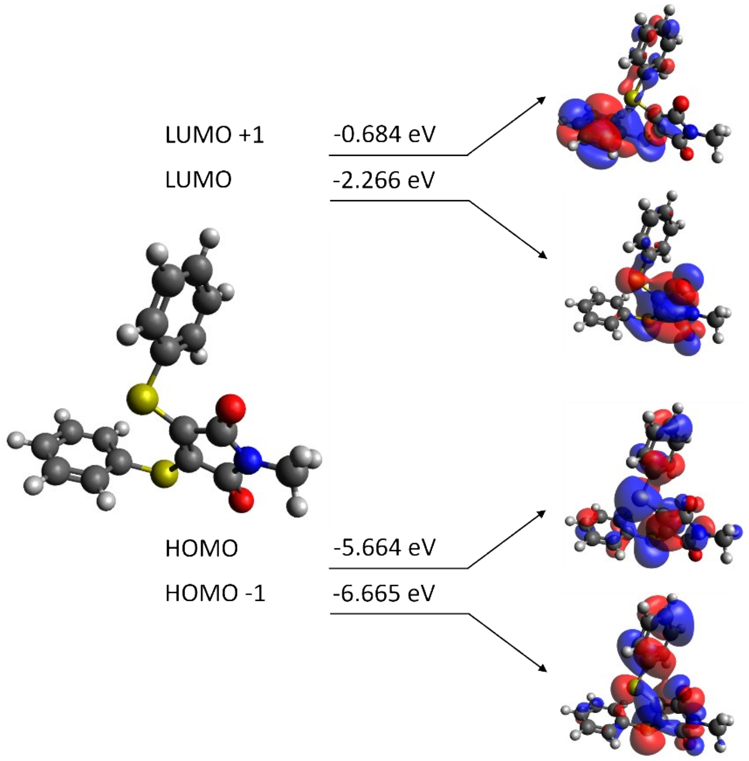


*Figure 115: HOMO-1, HOMO, LUMO and LUMO +1 projection of 2a, plus corresponding energies (eV).*

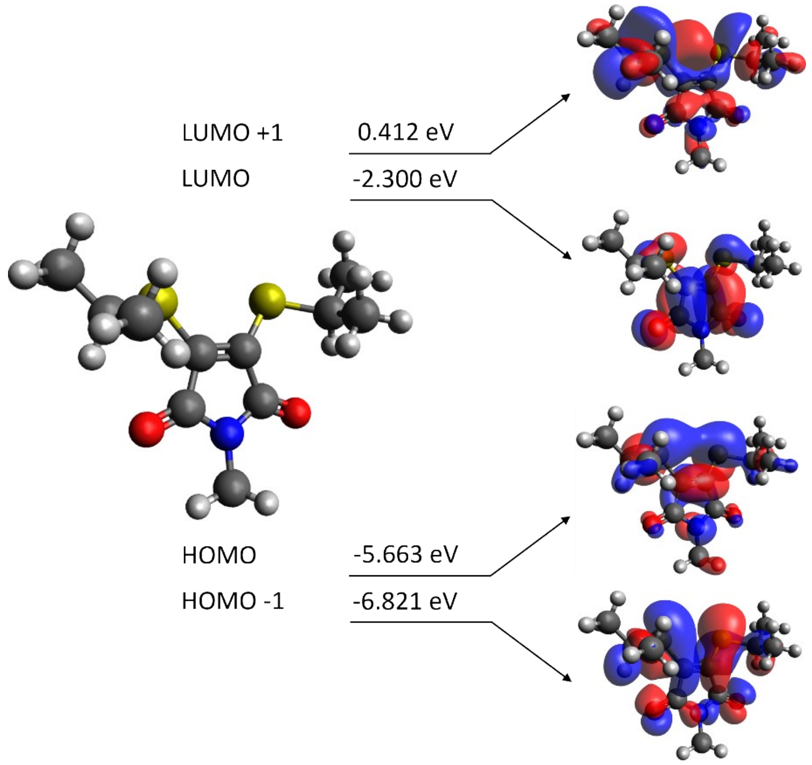


*Figure 116: HOMO-1, HOMO, LUMO and LUMO +1 projection of 2b, plus corresponding energies (eV).*

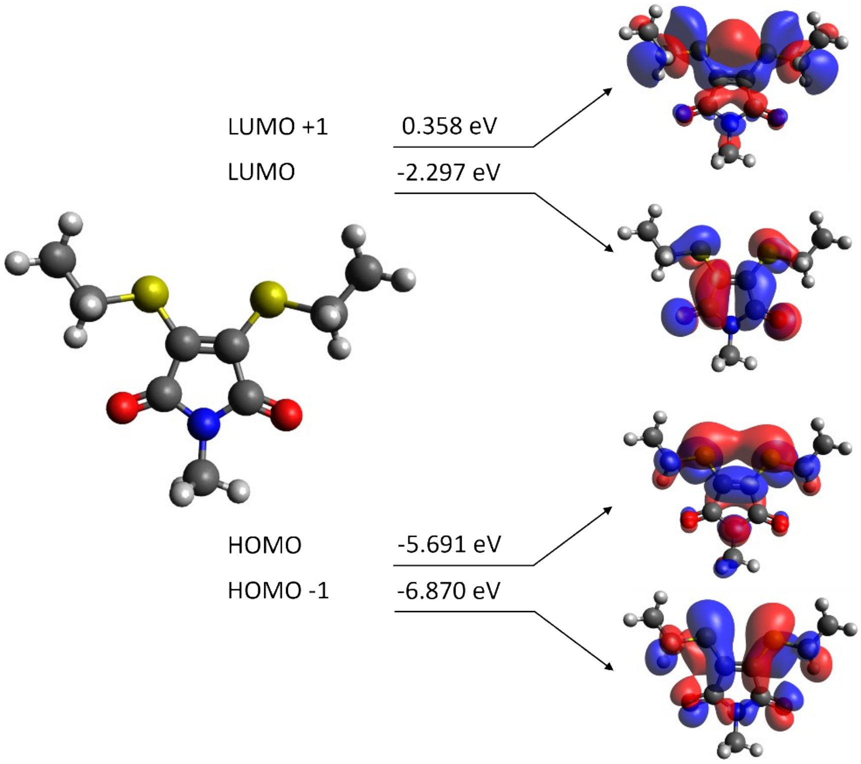


*Figure 117: HOMO-1, HOMO, LUMO and LUMO +1 projection of 2c, plus corresponding energies (eV).*

# HOMO-LUMO Projections- ATM

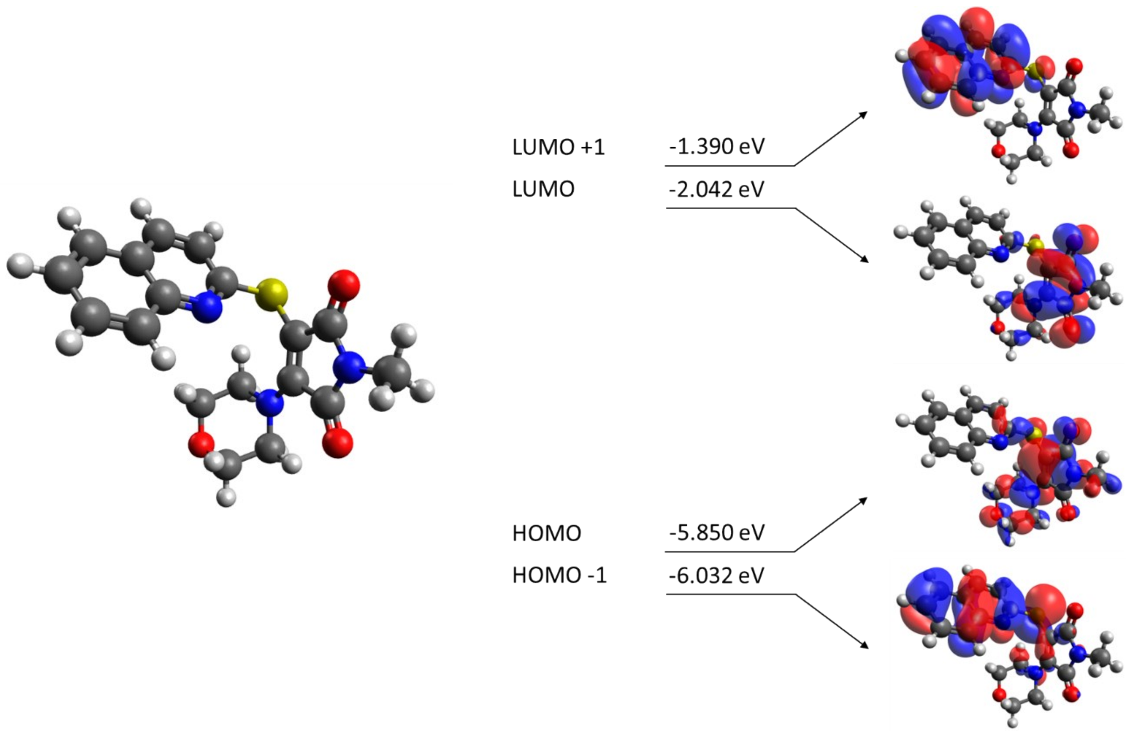


*Figure 118: HOMO-1, HOMO, LUMO and LUMO +1 projection of 3a, plus corresponding energies (eV).*

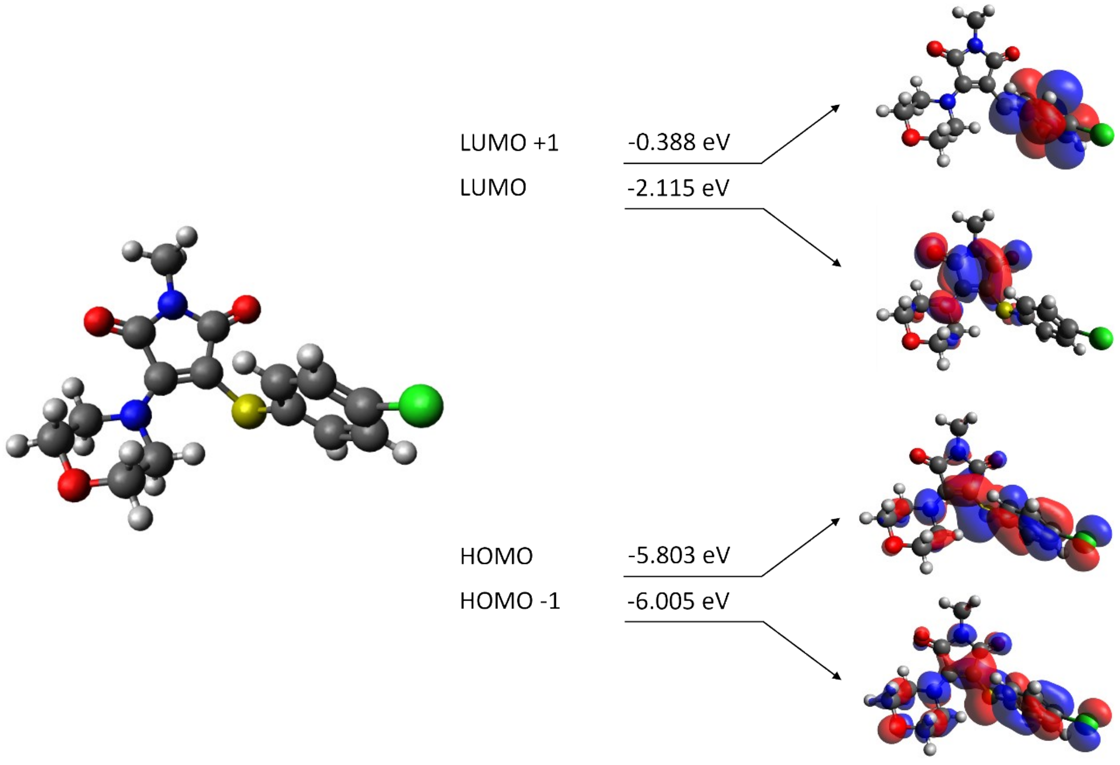


*Figure 119: HOMO-1, HOMO, LUMO and LUMO +1 projection of 3b, plus corresponding energies (eV).*

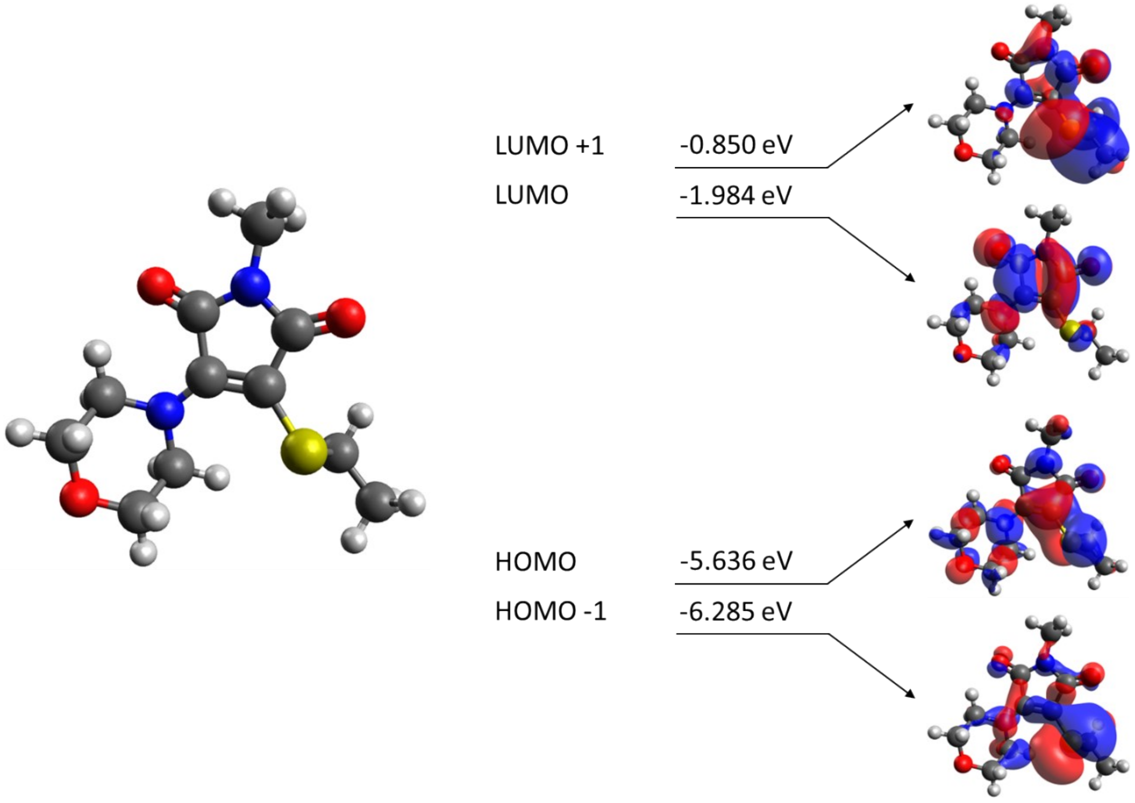


*Figure 120: HOMO-1, HOMO, LUMO and LUMO +1 projection of 3c, plus corresponding energies (eV).*

# Computational methods: TD-DFT

TD-DFT calculations were performed with ORCA (version 5.0.3) using the B3LYP functional and 6-31G** basis sets, With initial modelling, optimisation and analysis in Avogadro (version 1.2.0). We would like to acknowledge the University of Birmingham's advanced computing services; BLUEBEAR for resources and aiding in the calculations. Unless specified, calculations were performed *in vacuo*, with no solvent considerations. When solvation effects were considered, the CPCM model built-in to ORCA was used, either with solvent specification, or manual inputs of dielectric constant and refractivity index.

| | Molecule | HOMO (eV) | LUMO (eV) | Energy Gap (eV) | TD-DFT $\lambda_{max}$ (nm) | fosc |
|---|---|---|---|---|---|---|
| **AMs** | **1a** | -5.880 | -1.888 | 3.992 | 348.1 | 0.076732886 |
| | **1b** | -5.823 | -1.943 | 3.88 | 359.6 | 0.078407435 |
| | **1c** | -5.855 | -1.873 | 3.982 | 349.5 | 0.077871820 |
| | **1d** | -5.867 | -1.848 | 4.019 | 345.7 | 0.076380839 |
| | **1e** | -5.915 | -1.946 | 3.969 | 351.3 | 0.074174049 |
| | **1f** | -5.862 | -1.905 | 3.957 | 350.6 | 0.090861957 |
| | **1g** | -5.825 | -1.861 | 3.964 | 350.2 | 0.085910360 |
| | **1h** | -5.820 | -1.849 | 3.971 | 350.0 | 0.083437281 |
| | **1i** | -5.917 | -1.948 | 3.969 | 350.3 | 0.076465229 |
| | **1j** | -6.040 | -2.020 | 4.02 | 347.2 | 0.074327219 |
| | **1k** | -6.008 | -2.149 | 3.859 | 357.6 | 0.105502581 |
| | **1l** | -6.009 | -2.094 | 3.915 | 351.8 | 0.100891137 |
| **DTMs** | **2a** | -5.664 | -2.266 | 3.398 | 428.2 | 0.115400510 |
| | **2b** | -5.663 | -2.300 | 3.363 | 413.6 | 0.133383671 |
| | **2c** | -5.691 | -2.297 | 3.394 | 409.0 | 0.125760074 |
| **ATMs** | **3a** | -5.850 | -2.042 | 3.808 | 378.5<br>364.3 | 0.007767101<br>0.161501343 |
| | **3b** | -5.803 | -2.115 | 3.688 | 408.0 | 0.005156446 |
| | **3c** | -5.636 | -1.984 | 3.652 | 409.0 | 0.000099466 |

*Table 7: Summary of TD-DFT-determined values, including energies, absorbance wavelengths and their strength.*

# Additional ANN training statistics

Additional ANN test-set performance statistics are shown in Table 8 for a single representative ANN instance.

| Training type | Minimum error (nm) | Maximum error (nm) | Standard deviation (nm) |
|---|---|---|---|
| **Absorption** | 0.3 | 29.6 | 7.8 |
| **Emission** | 0.2 | 59.8 | 16.6 |

*Table 8: Representative test-set statistics for ANN fitting in the case of absorption and emission.*

# ANN training with validation set

Additional ANN training was performed using a validation set comprising 10% of the original training dataset; the resulting loss curve and validation-set $R^2$ values are shown in Fig. 121.

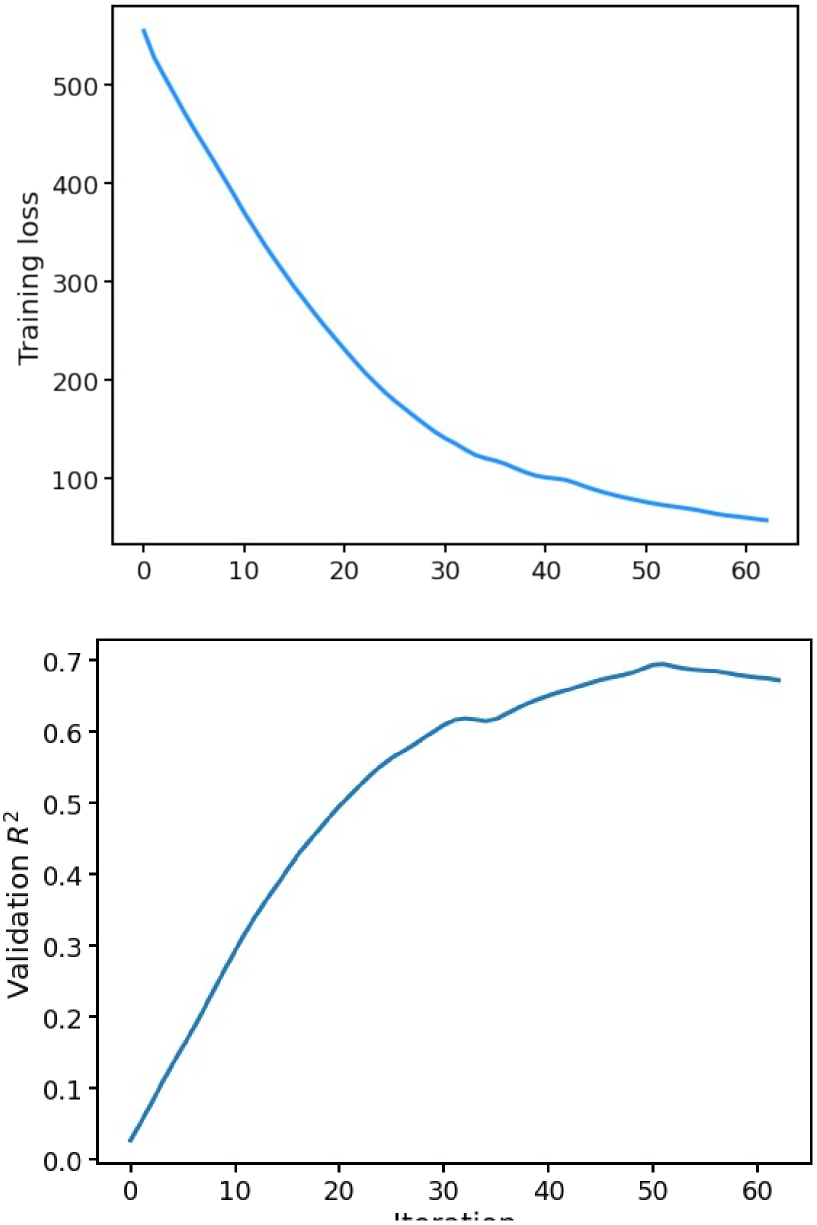


*Figure 121: Training loss curve (upper) and R2 value in validation set (lower panel) for ANN training in which 10% of the dataset is used as a validation set.*

# DFT functional/basis-set comparisons

As representative tests of the predictions made by different combinations of exchange-correlation functional and basis sets in TD-DFT calculations, Fig. 122 presents further calculations for the novel maleimides synthesised in this article (*cf.* Fig. 10 in main text).

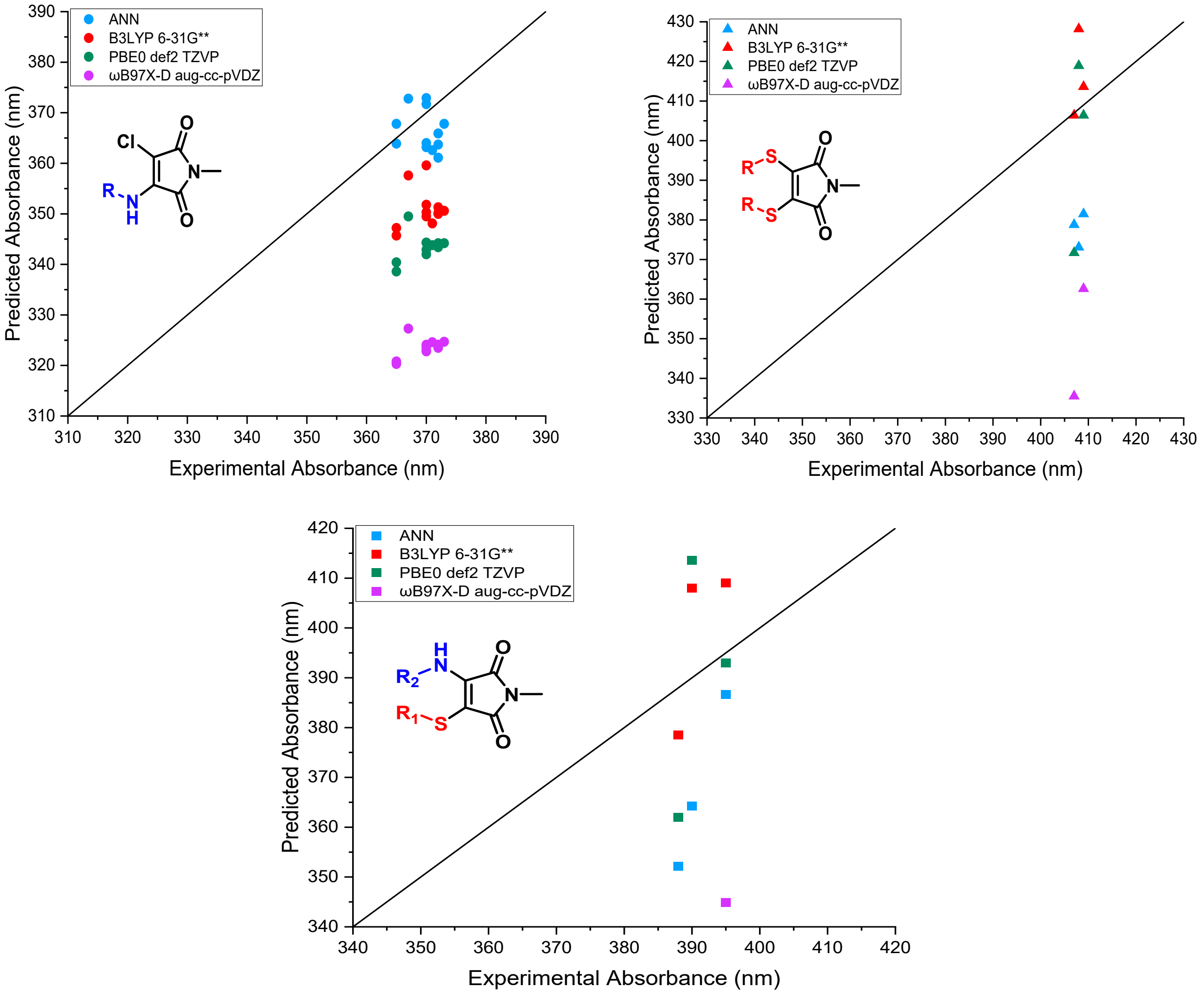


*Figure 122: Comparison of three different combinations of exchange-correlation functional and basis sets in TD-DFT calculations for novel maleimides synthesised in this article (see Figs. 7-10 in main text).*

# Appendix- IR Spectra

All IR spectra were recorded using an Agilent Technologies Cary FTIR spectrometer. Sixteen scans were selected, and each spectrum was normalised by taking a background scan before each sample scan.

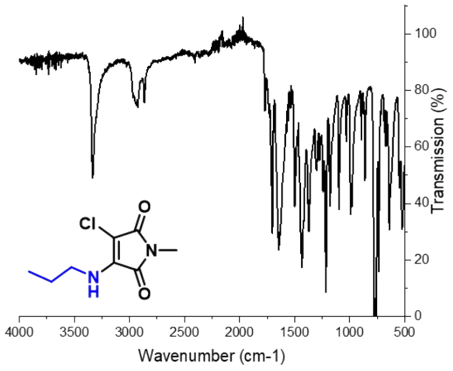


*Figure 123: FTIR Spectrum of 1a*

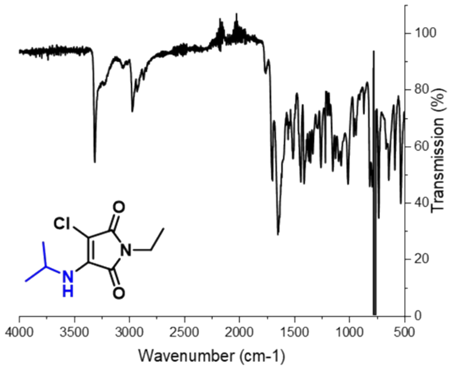


*Figure 124: FTIR Spectrum of 1b*

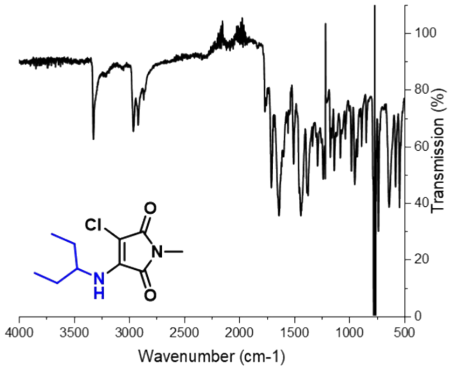


*Figure 125: FTIR Spectrum of 1c*

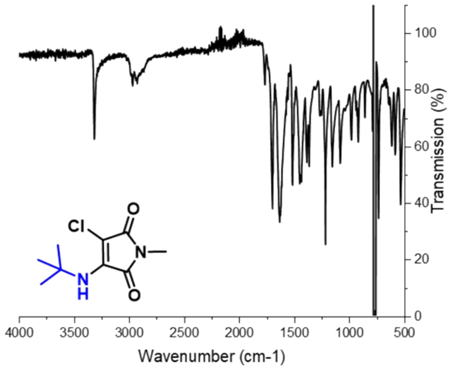


*Figure 126: FTIR Spectrum of 1d*

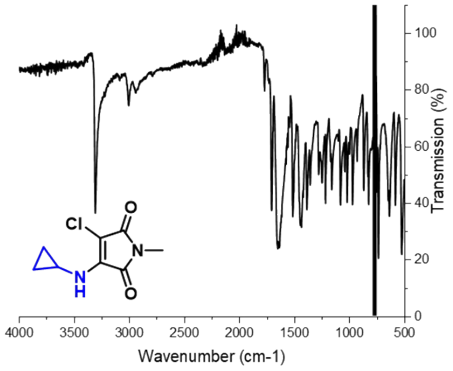


*Figure 127: FTIR Spectrum of 1e*

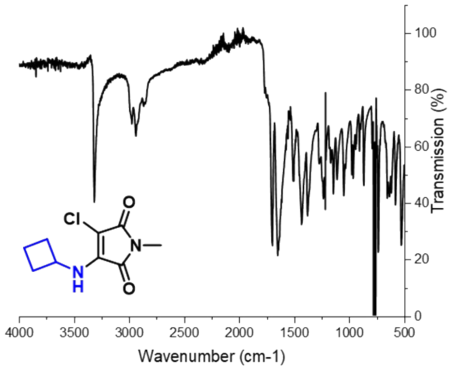


*Figure 128: FTIR Spectrum of 1f*

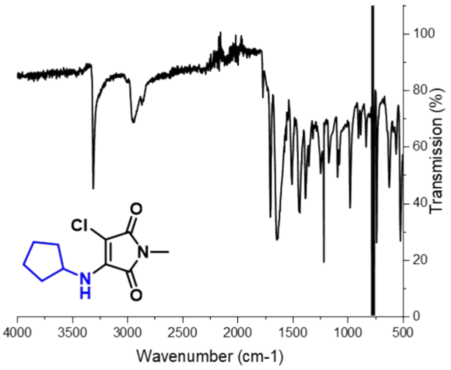


*Figure 129: FTIR Spectrum of 1g*

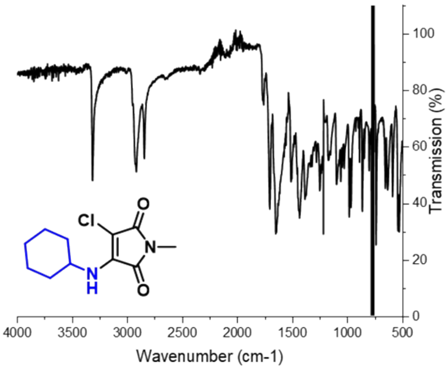


*Figure 130: FTIR Spectrum of 1h*

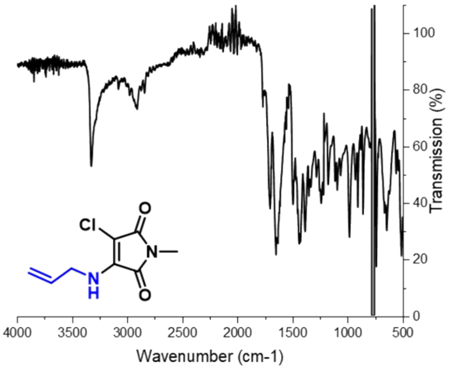


*Figure 131: FTIR Spectrum of 1i*

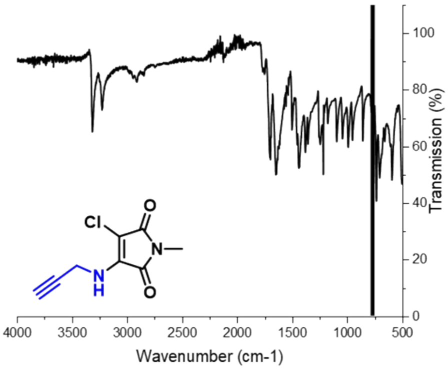


*Figure 132: FTIR Spectrum of 1j*

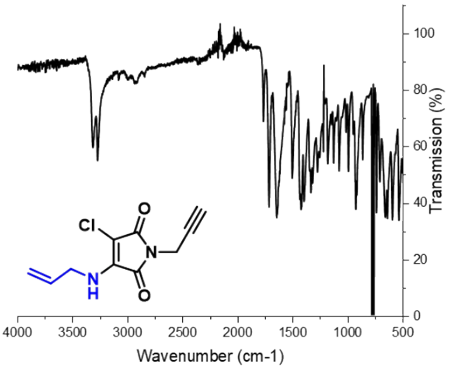


*Figure 133: FTIR Spectrum of 1k*

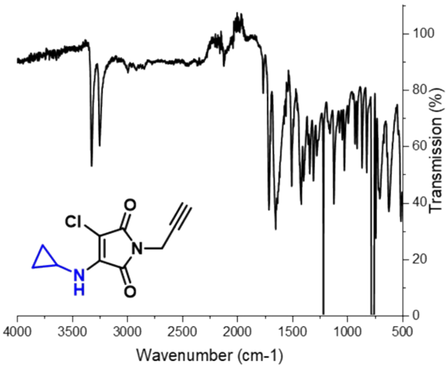


*Figure 134: FTIR Spectrum of 1l*

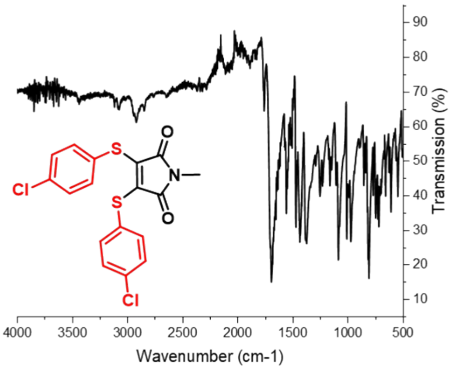


Figure 135: FTIR Spectrum of 2a

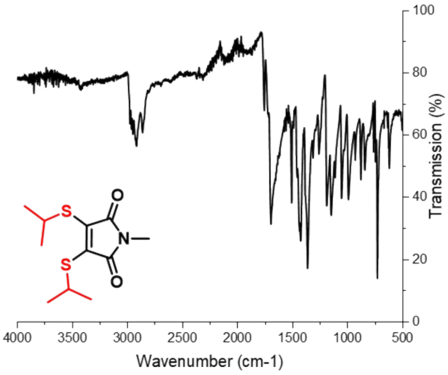


Figure 136: FTIR Spectrum of 2b

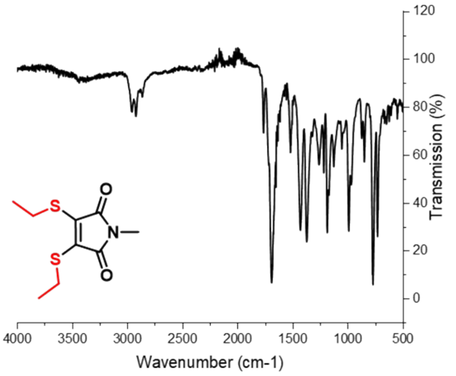


Figure 137: FTIR Spectrum of 2c

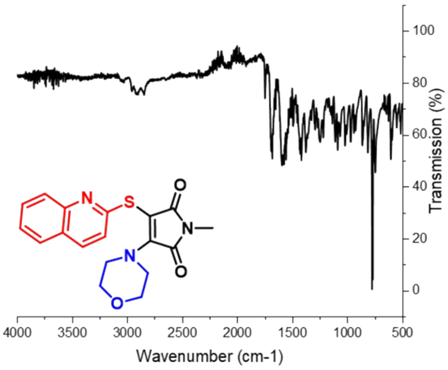


Figure 138: FTIR Spectrum of 3a

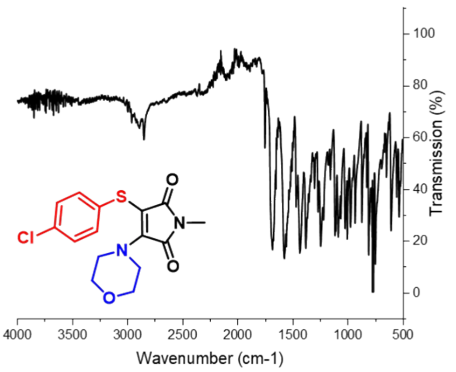


Figure 139: FTIR Spectrum of 3b

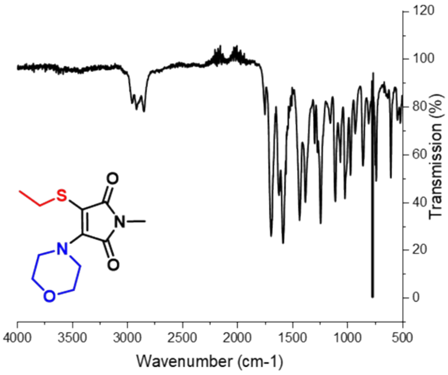


Figure 140: FTIR Spectrum of 3c

# Appendix- TD-DFT Geometries (*in vacuo*)

## AMs

### 1a (3-chloro-1-methyl-4-(propylamino)-1H-pyrrole-2,5-dione)

| | | | |
|---|---|---|---|
| C | -5.01190033056482 | -0.65879045355944 | -0.24080978432001 |
| C | -3.92951065071340 | -0.79281484809517 | 0.57629916804165 |
| C | -5.11775150886891 | 0.81639479768089 | -0.60430663108697 |
| C | -3.30953334887069 | 0.52946941614641 | 0.78124818214334 |
| N | -4.09090469236407 | 1.46006225536982 | 0.03428160454682 |
| O | -2.33702501177201 | 0.84442236896197 | 1.43272951568553 |
| O | -5.97238877879507 | 1.28868038551538 | -1.33516323468736 |
| C | -3.80613739109774 | 2.88139037494199 | -0.04266077789099 |
| H | -4.56390760927040 | 3.33582552515533 | -0.68240001085153 |
| H | -2.81312902116999 | 3.05138994681415 | -0.46800263695332 |
| H | -3.84256009558399 | 3.33348418117877 | 0.95225661241944 |
| N | -5.96051141886356 | -1.44858401852476 | -0.76756452818621 |
| Cl | -3.26588188083335 | -2.20228554145796 | 1.33530482973849 |
| C | -6.07153015721218 | -2.90267704488000 | -0.69637222601678 |
| C | -7.51801948397706 | -3.34980880542652 | -0.44803235264107 |
| H | -5.42096282824010 | -3.25299004861744 | 0.10702623215145 |
| H | -5.70002862393601 | -3.34493396181704 | -1.63197439331273 |
| C | -8.07158870409530 | -2.92498803257391 | 0.91537665923541 |
| H | -7.54698852119158 | -4.44225928314940 | -0.54362104066403 |
| H | -8.15721985712790 | -2.95960601776097 | -1.25281963167390 |
| H | -7.47736140762037 | -3.35086419281796 | 1.73166773086265 |
| H | -9.10474953102617 | -3.26328491724608 | 1.04149569404991 |
| H | -8.05893963596390 | -1.83629914070464 | 1.02820987080780 |
| H | -6.59320951084128 | -0.95023294513339 | -1.38644885139761 |

## 1b (3-chloro-1-ethyl-4-(isopropylamino)-1H-pyrrole-2,5-dione)

| | | | |
|---|---|---|---|
| C | -3.97481882758302 | -0.62385428384034 | -0.31090758981316 |
| C | -2.77546996399326 | -0.28717019979342 | -0.86088765461824 |
| C | -2.54383575024882 | 1.15013057667172 | -0.71357686459864 |
| N | -3.69189166518330 | 1.67143767704048 | -0.03813940489346 |
| C | -4.59139834364839 | 0.66388164754627 | 0.23776713205067 |
| O | -1.60808263484384 | 1.83771094252272 | -1.06630952035526 |
| O | -5.66916386185360 | 0.75139676144046 | 0.80222573532768 |
| C | -3.76330011099430 | 3.10878569781143 | 0.26405183229109 |
| N | -4.54230494726284 | -1.84078898992925 | -0.20319581490104 |
| Cl | -1.65342425227009 | -1.36003241661481 | -1.62391928878054 |
| C | -5.12398951227193 | 3.61213703448363 | 0.73976104923508 |
| H | -3.46658568005520 | 3.63033028645594 | -0.65141872183978 |
| H | -2.99153243488266 | 3.33518887830735 | 1.00964084303392 |
| H | -5.90621192700973 | 3.43093692051022 | -0.00164872712742 |
| H | -5.04535039516130 | 4.69291479444111 | 0.89692018220529 |
| H | -5.43462382612237 | 3.14547776614679 | 1.67507847498799 |
| C | -5.90352070028774 | -2.17037519179945 | 0.25874180331754 |
| H | -4.04448940773235 | -2.58168699636918 | -0.68369933641959 |
| C | -6.93867826220373 | -1.97146047999628 | -0.85748605752079 |
| H | -6.94910030685723 | -0.93014314684972 | -1.19023247403267 |
| H | -7.94164180233108 | -2.22043771574874 | -0.49479584455070 |
| H | -6.71824519915274 | -2.61281367448863 | -1.71877580062031 |
| C | -5.89063442854212 | -3.60609871171074 | 0.79029638815166 |
| H | -6.13409945581227 | -1.48074132700281 | 1.07216973123745 |
| H | -5.16714949711428 | -3.72084546147325 | 1.60287404210038 |
| H | -5.63388363782441 | -4.31969063972070 | -0.00349333753167 |
| H | -6.88033316875730 | -3.87770974804080 | 1.16838922366449 |

## 1c (3-chloro-1-methyl-4-(pentan-3-ylamino)-1H-pyrrole-2,5-dione)

| | | | |
|---|---|---|---|
| C | -6.72501511114828 | -1.02296798962198 | 0.18819554567002 |
| C | -5.61849923620016 | -1.16464038423101 | 0.97193737746296 |
| C | -4.84156148253982 | 0.08699398458723 | 0.94781806463886 |
| N | -5.55577411341120 | 0.98674002265961 | 0.09682321586258 |
| C | -6.68332492545678 | 0.38430050969286 | -0.39238738112610 |
| O | -3.80344241864982 | 0.37073791098942 | 1.50588464267857 |
| O | -7.51597776503513 | 0.84714561967290 | -1.15351516335205 |
| C | -5.12385084915982 | 2.33928452185237 | -0.20293171740581 |
| H | -4.19042088445523 | 2.50439881887901 | 0.33713168758203 |
| H | -5.87289426609457 | 3.06642191909133 | 0.12404284977612 |
| H | -4.95607789401226 | 2.46366917862934 | -1.27677232075110 |
| N | -7.78689926302222 | -1.76392626946973 | -0.16186220637676 |
| C | -8.01096025025693 | -3.19424173121794 | 0.09464046341279 |
| H | -8.39197255796486 | -1.29890684027594 | -0.83244056531126 |
| C | -9.53816395635817 | -3.40191843638197 | 0.10950857272619 |
| C | -10.02013554642850 | -4.81912062456913 | 0.43453151974338 |
| H | -9.93567279818564 | -3.10305301505565 | -0.87222049316730 |
| H | -9.95963485120855 | -2.70043916501067 | 0.83954422210214 |
| H | -9.74918727350896 | -5.53597479618692 | -0.34573614867691 |
| H | -11.11098097861102 | -4.83196074345251 | 0.52472323619681 |
| H | -9.60574924471387 | -5.17745195360739 | 1.38332736070213 |
| C | -7.23888868089614 | -4.06729007507003 | -0.93077386204776 |
| H | -7.61544478897860 | -3.40077453614341 | 1.09478979419224 |
| C | -6.77572669375781 | -5.42987332022784 | -0.39706577559475 |
| H | -6.35131164779176 | -3.50595442283847 | -1.24313704863455 |
| H | -7.85386461928354 | -4.19493102689199 | -1.83233364561778 |
| H | -6.12311825918948 | -5.30080117430582 | 0.47305263344518 |
| H | -6.20319347318579 | -5.96285758517667 | -1.16315578498007 |

| | | | |
|---|---|---|---|
| H | -7.60702772184386 | -6.07395081482332 | -0.09952434305699 |
| Cl | -5.08152844865110 | -2.51441758149563 | 1.91754526990718 |

## 1d (3-(tert-butylamino)-4-chloro-1-methyl-1H-pyrrole-2,5-dione)

| | | | |
|---|---|---|---|
| C | -2.52766901881646 | -0.18647130426272 | -0.96777985414857 |
| C | -1.64347085181971 | -0.56805669577454 | 0.00058462794375 |
| C | -2.83446465475157 | 1.24853324337388 | -0.81207883223052 |
| C | -1.35460241482670 | 0.67849106900261 | 0.83938363536364 |
| N | -2.08435801201836 | 1.70561723967427 | 0.30899954734658 |
| O | -3.56374258512432 | 1.96444459718381 | -1.46430065812868 |
| O | -0.60091189744077 | 0.70094806572581 | 1.79846362805559 |
| N | -0.97683905536320 | -1.65428227900358 | 0.42659226123718 |
| C | -0.93363354264539 | -3.05178658987502 | -0.05998005519050 |
| C | -2.32855335820521 | -3.69855423769780 | 0.03950624349376 |
| H | -2.69469243205589 | -3.66325659898157 | 1.07052833348352 |
| H | -2.27491476016611 | -4.74759852345347 | -0.26992822938227 |
| H | -3.05282632494864 | -3.19412493707391 | -0.60277201999231 |
| C | -0.39050078326920 | -3.10325070189609 | -1.50103517470952 |
| H | -1.04830845649409 | -2.58111981213622 | -2.19833976254772 |
| H | -0.30680170119932 | -4.14436964264491 | -1.82984222040697 |
| H | 0.60292271700874 | -2.64630093806174 | -1.55239940913962 |
| C | 0.03994163348248 | -3.78404538133509 | 0.88115058941782 |
| H | -0.31314281792762 | -3.75126331359092 | 1.91789764012902 |
| H | 1.04034583059136 | -3.33912905727751 | 0.83839780935956 |
| H | 0.12572822757518 | -4.83362692404379 | 0.58796895537637 |
| Cl | -3.28576737112521 | -1.06639536269999 | -2.25492783814865 |
| H | -0.40056656150815 | -1.43473731981577 | 1.23426900217076 |
| C | -2.10329862720273 | 3.07273703063208 | 0.79689552830388 |
| H | -1.43030010186078 | 3.12737864786525 | 1.65360207474569 |

| | | | |
|---|---|---|---|
| H | -3.11425895014064 | 3.35658963262859 | 1.10197500312903 |
| H | -1.76687412974766 | 3.76154009353836 | 0.01717917446918 |

## 1e (3-chloro-4-(cyclopropylamino)-1-methyl-1H-pyrrole-2,5-dione)

| | | | |
|---|---|---|---|
| C | -5.33695258332292 | 5.00564591266607 | -0.19777671450053 |
| C | -4.03423632971753 | 4.61765174372296 | -0.25535926727748 |
| C | -5.35299072687715 | 6.50459865426268 | 0.05010809073530 |
| C | -3.17041105437283 | 5.79900678392623 | -0.04552562703439 |
| N | -4.04473348845487 | 6.90852880942981 | 0.13678876562971 |
| C | -3.59311984046063 | 8.26880093049535 | 0.36681187102100 |
| H | -4.47837266381176 | 8.89878668071488 | 0.46395314158008 |
| H | -2.98206913517864 | 8.61694448750251 | -0.47056805581216 |
| H | -2.99736417844371 | 8.32495760729198 | 1.28201329840930 |
| O | -1.96187492732888 | 5.88645759352449 | -0.02171213915954 |
| O | -6.36260056888235 | 7.18229681444884 | 0.14275966454490 |
| N | -6.54356661547676 | 4.41512213046495 | -0.29504751965912 |
| Cl | -3.34276924878684 | 3.05551551242218 | -0.52667512227144 |
| C | -6.79693943302016 | 3.04486819656337 | -0.64199037551564 |
| H | -7.31748254659877 | 5.07104688842497 | -0.28208924832184 |
| C | -8.02072976204899 | 2.39873594151255 | -0.04826046556792 |
| C | -6.64847433221847 | 1.97616221729158 | 0.41334222178167 |
| H | -8.59536829066236 | 1.72947320712377 | -0.68064712348020 |
| H | -8.61783155007473 | 2.99520082286581 | 0.63604480900479 |
| H | -6.25895064892298 | 1.01308600328785 | 0.09956627417596 |
| H | -6.32630786296351 | 2.29743873086331 | 1.39832373376079 |
| H | -6.53008421237507 | 2.77580433119377 | -1.66290021204325 |

## 1f (3-chloro-4-(cyclobutylamino)-1-methyl-1H-pyrrole-2,5-dione)

| | | | |
|---|---|---|---|
| C | -5.15224758690272 | 4.88376676533499 | -0.19061338273556 |
| C | -3.92799403653130 | 4.74737274449135 | -0.77239189125728 |
| C | -5.21513172408104 | 6.28559560693948 | 0.39783042444537 |
| C | -3.16838831599511 | 6.00057527457043 | -0.60332684824883 |
| N | -4.01977249197302 | 6.89242837855435 | 0.11287614665557 |
| C | -3.64273528643693 | 8.24025326590717 | 0.49813968098786 |
| H | -4.48175932095714 | 8.67487358975500 | 1.04320742213184 |
| H | -3.42403708834804 | 8.84463377914597 | -0.38649919154755 |
| H | -2.75583631988275 | 8.22221940935842 | 1.13755082060611 |
| O | -2.04857571402694 | 6.29069335659963 | -0.96582563342799 |
| O | -6.17018825711744 | 6.73806698807335 | 1.00669260666510 |
| N | -6.25817342013659 | 4.13761104204558 | -0.02883240206424 |
| Cl | -3.22951791642119 | 3.39604036428600 | -1.60129996307939 |
| C | -6.45422029701840 | 2.74543447331697 | -0.37181649208704 |
| H | -6.96843819783486 | 4.59649672491528 | 0.53400567246735 |
| C | -7.90073418366914 | 2.29162872234676 | -0.69065316300426 |
| C | -7.58348923142449 | 0.95045878716219 | 0.02651002593446 |
| H | -8.20849518432258 | 2.27245805559495 | -1.73964224980435 |
| H | -8.62917367386515 | 2.88213916506541 | -0.12211105715103 |
| C | -6.40034336606169 | 1.65755649167226 | 0.74246711045890 |
| H | -7.23702617639445 | 0.18685368214664 | -0.67623603420786 |
| H | -8.36685215644176 | 0.51837437061197 | 0.65349584930518 |
| H | -5.46066416813385 | 1.11329577860908 | 0.87057937376905 |
| H | -6.70518203521994 | 2.07472485236767 | 1.70830292587904 |
| H | -5.77427385080335 | 2.49391833112901 | -1.18694975069045 |

## 1g (3-chloro-4-(cyclopentylamino)-1-methyl-1H-pyrrole-2,5-dione)

| | | | |
|---|---|---|---|
| C | -5.17774307416894 | 4.70005293006235 | -0.17286453235712 |

| | | | |
|---|---|---|---|
| C | -3.94905429027730 | 4.58693329105811 | -0.75079498571710 |
| C | -5.29478372909736 | 6.12224849095703 | 0.35692239478347 |
| C | -3.23762500354772 | 5.87315265771052 | -0.63426312746017 |
| N | -4.12250007102046 | 6.76078697850930 | 0.04680843071726 |
| C | -3.79863644996682 | 8.13751873802299 | 0.37336563187901 |
| H | -4.65542692839448 | 8.56246653684679 | 0.89811042640796 |
| H | -3.60075138005166 | 8.71102133413475 | -0.53641698144282 |
| H | -2.91349330664095 | 8.18185540775071 | 1.01402119478569 |
| O | -2.12959002037375 | 6.19131089008604 | -1.00956535533576 |
| O | -6.26681048027787 | 6.56393211650191 | 0.94642812997409 |
| N | -6.25219410593997 | 3.91891569183992 | 0.01880714311501 |
| Cl | -3.19861019343614 | 3.22724573767554 | -1.52012933422359 |
| C | -6.38558057946066 | 2.49894016000867 | -0.27744015686077 |
| H | -6.98075177558261 | 4.36579314164809 | 0.56742992284081 |
| C | -7.84696525561817 | 2.08604638135918 | -0.51353501773046 |
| C | -5.92771808729744 | 1.55260049219411 | 0.85753831287991 |
| H | -5.80105633193220 | 2.29492400956548 | -1.17956578439574 |
| C | -7.80017879742185 | 0.54908552232176 | -0.43436683561396 |
| H | -8.24878326891114 | 2.46409492407662 | -1.45837823678560 |
| H | -8.46837127176906 | 2.49365221656049 | 0.29712082178060 |
| C | -6.66058809583555 | 0.21497822163283 | 0.57811523363787 |
| H | -7.56025113799238 | 0.13762540604011 | -1.42109479970670 |
| H | -8.76299162977656 | 0.12180230287752 | -0.13995066980124 |
| H | -5.97864039138584 | -0.53088036644029 | 0.15865284521138 |
| H | -7.05768427177281 | -0.21144861411692 | 1.50408649741752 |
| H | -6.24838521655539 | 1.97861148678636 | 1.81607981372223 |
| H | -4.83909485549482 | 1.45287391432994 | 0.89332901827822 |

## 1h (3-chloro-4-(cyclohexylamino)-1-methyl-1H-pyrrole-2,5-dione)

| | | | |
|---|---|---|---|
| C | -7.59611721623929 | 4.27627749216779 | 0.84175728629647 |
| C | -6.13423579779000 | 4.10727718067857 | 0.93113547937793 |
| C | -5.76087973015521 | 3.02785924909611 | 0.18742299603874 |
| C | -7.02700368161486 | 2.44963243927560 | -0.42938664338862 |
| N | -8.07069051002684 | 3.22781279787056 | -0.00123723798384 |
| O | -8.32010928803915 | 5.10745351503275 | 1.34654078372461 |
| O | -7.05759605104721 | 1.47747100700128 | -1.16507816588783 |
| C | -9.46665193777966 | 3.03590156391296 | -0.34923146354606 |
| H | -9.52662571296483 | 2.16668002178958 | -1.00570008959140 |
| H | -9.85791291458549 | 3.91685755450075 | -0.86562026214853 |
| H | -10.06551466733314 | 2.86256747656565 | 0.54918929615731 |
| N | -4.62427450653225 | 2.37623031716615 | -0.10299850778358 |
| Cl | -5.17528856292828 | 5.20109501458972 | 1.87340646997400 |
| C | -3.25511043621396 | 2.76472672705980 | 0.24114302678565 |
| C | -2.67771180377414 | 3.78870589458514 | -0.75672839355152 |
| C | -2.37550932490137 | 1.50677086998769 | 0.31663583508405 |
| H | -3.29120684215959 | 3.22734817143594 | 1.23338051107760 |
| C | -0.91698785983336 | 1.85986046500483 | 0.65047252295866 |
| H | -2.41165787976108 | 0.99166844841521 | -0.65579825499348 |
| H | -2.78708899818316 | 0.81406002708441 | 1.05969077844436 |
| C | -0.33967060529493 | 2.88695731215325 | -0.33586853854214 |
| H | -0.30920724995218 | 0.94763225477662 | 0.65507176821269 |
| H | -0.87085416543301 | 2.26921055737652 | 1.66948441748431 |
| C | -1.22189343169062 | 4.14250876826114 | -0.41252070221526 |
| H | -0.26942900806341 | 2.43152183222024 | -1.33416082587932 |
| H | 0.68188201401718 | 3.15730419532375 | -0.04356561145662 |
| H | -3.30406952650476 | 4.68766177147693 | -0.75900191679352 |
| H | -2.72795150568896 | 3.35900442973266 | -1.76745216050887 |

| | | | |
|---|---|---|---|
| H | -0.82597958435164 | 4.84510176311675 | -1.15492241041048 |
| H | -1.19618294399767 | 4.66518394029350 | 0.55415241568433 |
| H | -4.75565027117704 | 1.61587694204809 | -0.76329240261961 |

## 1i (3-(allylamino)-4-chloro-1-methyl-1H-pyrrole-2,5-dione)

| | | | |
|---|---|---|---|
| C | -4.80961763989736 | 1.50285288727090 | 0.00523762274636 |
| C | -4.47589176210667 | 0.18299209025828 | 0.01142866856067 |
| C | -3.51345193423403 | 2.29787795308223 | -0.00056700251575 |
| C | -3.00497305970205 | 0.05775014245661 | 0.01002634572796 |
| N | -2.48907384949296 | 1.38715187690562 | 0.00259706349973 |
| C | -1.07331514222189 | 1.70837618094322 | -0.00112152552724 |
| H | -0.98147708415543 | 2.79532783534060 | -0.00697323420366 |
| H | -0.58820417145646 | 1.29151342638370 | -0.88802527550089 |
| H | -0.58562473705699 | 1.30086478474828 | 0.88870961637031 |
| O | -2.30531569539631 | -0.93184632843254 | 0.01405179580766 |
| O | -3.44827129003368 | 3.51563947518121 | -0.00678955300974 |
| N | -5.93867730471473 | 2.22596721024478 | 0.00315056548915 |
| Cl | -5.48782996871469 | -1.22383907430025 | 0.01977810604175 |
| C | -7.30298899199630 | 1.71054913710951 | 0.00815718937840 |
| C | -8.30004918627586 | 2.84818338562695 | 0.00339377433314 |
| H | -7.45176957615850 | 1.07662076212309 | 0.89212650899268 |
| H | -7.45452856979222 | 1.06685350054492 | -0.86824614870524 |
| C | -9.62192361364352 | 2.69036669393546 | 0.00728946492571 |
| H | -7.88452578990302 | 3.85644279705881 | -0.00379866096121 |
| H | -10.08693950375120 | 1.70700618926365 | 0.01443697955458 |
| H | -10.29121235925870 | 3.54461220623633 | 0.00343966082402 |
| H | -5.78459877003740 | 3.22793686801862 | -0.00208196182837 |

## 1j (3-chloro-1-methyl-4-(prop-2-yn-1-ylamino)-1H-pyrrole-2,5-dione)

| | | | |
|---|---|---|---|
| C | -5.41373801445431 | 5.08657243687623 | 0.02303482220592 |
| C | -4.13452973642573 | 4.62394511677736 | 0.00198766122024 |
| C | -5.34709706565740 | 6.60565064182706 | 0.05372341323942 |
| C | -3.20571205714357 | 5.77276402729974 | 0.01747636107617 |
| N | -4.01752759155356 | 6.94321857125593 | 0.04884219460488 |
| C | -3.48998012698653 | 8.29589764697544 | 0.07181756071160 |
| H | -4.33865735289379 | 8.98086287636933 | 0.09359207935173 |
| H | -2.88432852120600 | 8.48583021824405 | -0.81851261578906 |
| H | -2.86817482735049 | 8.44935170364645 | 0.95803061021942 |
| O | -1.99431016289347 | 5.79567231710929 | 0.00725488648011 |
| O | -6.31784304218904 | 7.34181535354768 | 0.07754749169315 |
| N | -6.64519937598994 | 4.55248967175984 | 0.02312815657494 |
| Cl | -3.53826563936253 | 2.99735795367584 | -0.03723479286425 |
| C | -6.96220132837877 | 3.12457015135324 | -0.00287288369054 |
| C | -8.40896268687218 | 2.92069238247005 | 0.00606505720796 |
| H | -6.50992771845863 | 2.62464071829903 | 0.86389514277903 |
| H | -6.52584376154114 | 2.66025258817369 | -0.89716191667275 |
| C | -9.60346670964886 | 2.75201397668837 | 0.01318144287546 |
| H | -7.40496141821208 | 5.22243048996353 | 0.04386998597778 |
| H | -10.65861286278190 | 2.59890115768782 | 0.01963534279879 |

## 1k (3-(allylamino)-4-chloro-1-(prop-2-yn-1-yl)-1H-pyrrole-2,5-dione)

| | | | |
|---|---|---|---|
| C | -0.69493064730039 | 0.98018027299339 | -0.37136586757000 |
| C | 0.57017795220788 | 1.23228383402256 | -0.81642860446994 |
| C | -1.14503662512250 | 2.21683002795179 | 0.40111923615280 |
| N | -0.08748066335061 | 3.09926359279119 | 0.35478487714024 |
| C | 1.00886241176297 | 2.56258119319656 | -0.38657497048443 |
| C | -0.10618895417091 | 4.44909422699344 | 0.90640155778546 |

| | | | |
|---|---|---|---|
| O | -2.20932155381974 | 2.40112659727767 | 0.96394055454388 |
| O | 2.05568942210750 | 3.14778621949827 | -0.56254607489021 |
| Cl | 1.56484431557196 | 0.15703305851783 | -1.73402604430381 |
| N | -1.43551650944881 | -0.13147863567139 | -0.53565735940134 |
| C | -2.81475045900065 | -0.37712132086831 | -0.09633485380837 |
| C | -2.91466864644918 | -1.69439639770975 | 0.62578995314324 |
| H | -3.48531163731899 | -0.37315904728122 | -0.96637028214549 |
| H | -3.10414078835659 | 0.45317095276005 | 0.55045817714770 |
| C | -3.67356866191680 | -2.71174890508636 | 0.21815979883408 |
| H | -2.31531200656272 | -1.78492689363214 | 1.53125224403949 |
| H | -4.27739853361286 | -2.64651591819195 | -0.68457849490576 |
| H | -3.72983240489452 | -3.64187107519855 | 0.77576663936696 |
| C | -0.78568197686624 | 5.42651060067173 | 0.04937578693710 |
| H | 0.94087838676363 | 4.73585586376754 | 1.04754659820174 |
| H | -0.59097551230033 | 4.40836216588649 | 1.88751086800158 |
| C | -1.33912133329827 | 6.24520724236819 | -0.64317816336678 |
| H | -1.82881251791383 | 6.96028888624016 | -1.26337705269587 |
| H | -0.99618305670998 | -0.87180654129726 | -1.06943852325225 |

## 11 (3-chloro-4-(cyclopropylamino)-1-(prop-2-yn-1-yl)-1H-pyrrole-2,5-dione)

| | | | |
|---|---|---|---|
| C | -0.55682269624218 | 0.91717866038692 | 0.00063016369852 |
| C | 0.64444257851735 | 1.16449150170146 | -0.59166281824019 |
| C | -1.04251793475250 | 2.23263685155406 | 0.59400293078254 |
| N | -0.07832868579830 | 3.16969824223322 | 0.27498874069645 |
| C | 0.99929227467664 | 2.58301246149038 | -0.44961716607492 |
| C | -0.15322290348996 | 4.58668349357141 | 0.60864606882385 |
| O | -2.05234787881232 | 2.42636237124662 | 1.24171431529405 |
| O | 1.97898390072204 | 3.19249677052426 | -0.82154466796381 |
| Cl | 1.65373998399757 | 0.01276728631057 | -1.39319945463212 |

| | | | |
|---|---|---|---|
| N | -1.23722345617554 | -0.24382613668804 | 0.09944531289797 |
| C | -2.48982581644611 | -0.44407267267966 | 0.78423113003217 |
| C | -3.38214779215042 | -1.54031675363847 | 0.26646765936827 |
| C | -0.96054635439031 | 5.37109367512459 | -0.33221468738924 |
| H | 0.87717080536477 | 4.95654618875639 | 0.61861863445914 |
| H | -0.56532012730572 | 4.67467121186206 | 1.61951579290045 |
| C | -1.61501415520476 | 6.03495840947049 | -1.09851462319663 |
| H | -2.20235110233953 | 6.60869764126245 | -1.77791734977737 |
| H | -0.75220989201101 | -1.06571974561152 | -0.23729301815210 |
| C | -3.78074405367119 | -0.09454323648706 | 0.09258483048349 |
| H | -2.44989991512715 | -0.28551130096699 | 1.85924231723529 |
| H | -3.71001255565602 | 0.32968047052171 | -0.90375390897524 |
| H | -4.57863083824025 | 0.31186377740695 | 0.70525861916754 |
| H | -3.90515718055048 | -2.15347966954878 | 0.99354288626805 |
| H | -3.05395620491454 | -2.07757949780314 | -0.61977170770618 |

# DTMs

## 2a (3,4-bis((4-chlorophenyl)thio)-1-methyl-1H-pyrrole-2,5-dione)

| | | | |
|---|---|---|---|
| C | -8.12311422414932 | -4.52352422727871 | 0.03906755567290 |
| C | -6.91280826453255 | -5.07301163157015 | -0.24196769624830 |
| C | -7.97270324586971 | -3.01907007628758 | 0.04187871585411 |
| C | -5.94714597112054 | -3.94273923658953 | -0.51855782411387 |
| N | -6.65012418753486 | -2.75335056098992 | -0.28631904105296 |
| C | -6.07796182901195 | -1.42291981212811 | -0.39294957213425 |
| H | -5.09283032456673 | -1.52620285437814 | -0.84981031941992 |
| H | -5.97705290730152 | -0.95868402143523 | 0.59307733379926 |
| H | -6.71624803459745 | -0.79103991144327 | -1.01530984356420 |
| O | -4.79273023860285 | -4.00768734534874 | -0.88296069326827 |
| O | -8.82551061829504 | -2.19013586986756 | 0.28981390337172 |
| S | -9.73294757638250 | -5.10996604757703 | 0.40743182377115 |
| S | -6.46399578866893 | -6.76289429086774 | -0.43000216593756 |
| C | -4.84936840935315 | -6.82320164436856 | 0.36206350863884 |
| C | -9.77801347141897 | -6.79075707119566 | -0.22710543382573 |
| C | -9.98760863607701 | -7.84738579424082 | 0.66607550073409 |
| C | -10.13111843334741 | -9.14743468759511 | 0.17853889936109 |
| C | -10.05727134358250 | -9.39530031925485 | -1.19341642144498 |
| C | -9.85565406867645 | -8.33734752339523 | -2.08372335927116 |
| C | -9.73120211898518 | -7.03247623947617 | -1.60694723171483 |
| H | -9.59657304077797 | -6.20574743734036 | -2.29747022342272 |
| H | -10.16187873098210 | -10.40903144924304 | -1.56928739530424 |
| H | -9.80747218667942 | -8.52514098927999 | -3.15254655275081 |
| H | -10.02688923847080 | -7.65174409783220 | 1.73290736809000 |
| H | -10.29080354687780 | -9.96691378751152 | 0.87347536641979 |
| C | -4.68461910439745 | -6.43276090830181 | 1.69689451366651 |

| | | | |
|---|---|---|---|
| C | -3.43869409873292 | -6.56925334001875 | 2.30873850366092 |
| C | -2.36487288066237 | -7.11998046334946 | 1.60456615913915 |
| C | -2.53810886599918 | -7.52597827884937 | 0.28022583366344 |
| C | -3.77553654647864 | -7.37133094521391 | -0.34694795289774 |
| H | -3.90639646619295 | -7.66100541669681 | -1.38477857953050 |
| H | -5.52568663434847 | -6.02636628735734 | 2.25050956203633 |
| H | -3.31097135195344 | -6.25681264379792 | 3.34152584058864 |
| H | -1.39821185409575 | -7.23293491227228 | 2.08702370500579 |
| H | -1.70556576127550 | -7.95060987764659 | -0.27371378757169 |

## 2b (3,4-bis(isopropylthio)-1-methyl-1H-pyrrole-2,5-dione)

| | | | |
|---|---|---|---|
| C | -1.65038134891868 | -2.10276819033976 | -0.70554020806944 |
| C | -0.28818896713058 | -2.06183368291645 | -0.76419140489526 |
| C | -2.17053902371294 | -0.71356907632114 | -0.96622794035888 |
| N | -1.05251579544917 | 0.09743880062430 | -1.15718354722241 |
| C | 0.12641804116877 | -0.64671104277185 | -1.06331181414074 |
| O | 1.24610556207368 | -0.18745472926347 | -1.19358136121982 |
| O | -3.32035928884154 | -0.31552922273562 | -1.00197569894785 |
| C | -1.10976482752984 | 1.53022732109922 | -1.38660832921204 |
| H | -0.08281025812689 | 1.88672052230430 | -1.47573199355294 |
| H | -1.60718984991949 | 2.03487680410519 | -0.55342417131960 |
| H | -1.65781949268148 | 1.74926943771774 | -2.30722711482059 |
| S | -2.62178437937195 | -3.54795547253840 | -0.55753002009627 |
| S | 0.77917074821309 | -3.44588946684086 | -0.70633050146156 |
| C | 2.38546433059032 | -2.79219902454254 | -0.01305212248522 |
| C | 3.38620423709202 | -3.94531144811378 | -0.13483625921690 |
| H | 3.07222712972946 | -4.81330630247522 | 0.45585523289841 |
| H | 4.36128828019063 | -3.61891040454427 | 0.24261159307854 |
| H | 3.51399988903755 | -4.26315278001593 | -1.17377092023868 |

| | | | |
|---|---|---|---|
| C | 2.22613338053821 | -2.30322000644311 | 1.42603614329703 |
| H | 2.68239860688315 | -1.96374762618984 | -0.65687749480603 |
| H | 1.50980029331108 | -1.48039143090642 | 1.49350227148750 |
| H | 3.19076057016350 | -1.93495093445505 | 1.79611682005078 |
| H | 1.89014170136522 | -3.11067186920489 | 2.08489192771376 |
| C | -4.21524009109919 | -2.98494372753750 | 0.23802056449895 |
| C | -3.99061342706380 | -2.47593210184259 | 1.66135266158646 |
| H | -3.56200107024071 | -3.25583404210097 | 2.29930986535393 |
| H | -4.94924682542025 | -2.16477797114737 | 2.09409281851753 |
| H | -3.32409251402052 | -1.60984898505869 | 1.67685729606173 |
| C | -5.15089125698563 | -4.19656952063856 | 0.18770701746295 |
| H | -4.60266707720729 | -2.18063162361896 | -0.38805041962177 |
| H | -5.32649320671815 | -4.53150768523956 | -0.83877943621649 |
| H | -6.11729032891094 | -3.92495906469520 | 0.62598347532316 |
| H | -4.74832374100757 | -5.03871545335263 | 0.76188307057175 |

## 2c (3,4-bis(ethylthio)-1-methyl-1H-pyrrole-2,5-dione)

| | | | |
|---|---|---|---|
| C | -3.52461809639104 | -1.37799784819292 | 0.35442530606730 |
| C | -2.16380839809930 | -1.29998179339720 | 0.34415132104086 |
| C | -4.07305647414150 | -0.04411932646466 | -0.07855676114810 |
| C | -1.77604861312730 | 0.08566076872287 | -0.09714312597966 |
| N | -2.97031416202578 | 0.76502510154403 | -0.35064663950260 |
| O | -0.66533052294694 | 0.56423962792470 | -0.23032027627680 |
| O | -5.23098531181905 | 0.31179636620376 | -0.19330185283279 |
| C | -3.05689111927071 | 2.13113137877865 | -0.83508286681595 |
| H | -2.03790215547069 | 2.50982200137969 | -0.92414563555772 |
| H | -3.62213332331378 | 2.74988534868154 | -0.13277904551370 |
| H | -3.55108653428574 | 2.16594286433390 | -1.81035474827700 |
| S | -4.46802551411313 | -2.72861398541339 | 0.93818261834880 |

| | | | |
|---|---|---|---|
| S | -1.06366650431522 | -2.53357438830974 | 0.91424233154758 |
| C | 0.49550151324531 | -2.24010910489920 | -0.04117093127438 |
| C | 1.50992243375046 | -3.30632007699645 | 0.36843361497818 |
| H | 0.84997860851384 | -1.23287686795712 | 0.17243575570308 |
| H | 0.25950744589595 | -2.30689179859879 | -1.10630109192185 |
| H | 1.74546351203307 | -3.24592723361327 | 1.43567684453225 |
| H | 2.43987966135089 | -3.15649420629061 | -0.18965093184841 |
| H | 1.14622481481638 | -4.31636704593847 | 0.15460443337754 |
| C | -6.06783832814594 | -2.60726004907181 | 0.01289421713812 |
| C | -6.95484585171232 | -3.77351478438982 | 0.44468997434237 |
| H | -5.84618304087314 | -2.65371002856601 | -1.05642475651315 |
| H | -6.52277880619875 | -1.64224247207272 | 0.23084315489866 |
| H | -6.48849596057532 | -4.74008077732431 | 0.22890401563286 |
| H | -7.90465585807383 | -3.72792387519338 | -0.09797932754708 |
| H | -7.17745341470638 | -3.73209779487925 | 1.51557440340159 |

# ATMs

## 3a (1-methyl-3-morpholino-4-(quinolin-2-ylthio)-1H-pyrrole-2,5-dione)

| | | | |
|---|---|---|---|
| C | 5.32415133128299 | 1.42098250239676 | -1.89921906686637 |
| C | 6.16174287076561 | 1.31168686211765 | -0.79063947874569 |
| C | 4.71218166843995 | 2.75010293235532 | -1.89784978331683 |
| C | 6.09769234700063 | 2.68340036961914 | -0.07331951309475 |
| N | 5.22147795853280 | 3.45350416355796 | -0.78138964763246 |
| O | 6.69293160529307 | 3.05703510569007 | 0.92754292411062 |
| O | 3.90584122763198 | 3.23875489364924 | -2.67713313562835 |
| C | 4.88211386592577 | 4.82524953304474 | -0.43730811190620 |
| H | 4.15648669680135 | 5.17487702348623 | -1.17152895487533 |
| H | 5.77188044965294 | 5.46010929588305 | -0.46905533148039 |
| H | 4.44405342543066 | 4.87027913191086 | 0.56331635820670 |
| N | 6.92641898295129 | 0.31660167641843 | -0.32460139411674 |
| S | 4.92415118330794 | 0.32395071013944 | -3.21337401738522 |
| C | 3.49944078542370 | -0.60844275601007 | -2.60703016858099 |
| C | 7.01158897626695 | -1.01480189558930 | -0.95089205295353 |
| C | 6.57931102137726 | -2.08495983490949 | 0.05552340012563 |
| H | 8.05637811034778 | -1.17981315147759 | -1.24074192079512 |
| H | 6.39277855874567 | -1.05716884857679 | -1.84279060694889 |
| O | 7.37060680342729 | -2.03729581230019 | 1.23837198766654 |
| H | 6.71701729688857 | -3.07747116498621 | -0.38241666583228 |
| H | 5.51453748503761 | -1.94799007531707 | 0.29964827401859 |
| C | 7.28240701956588 | -0.75649064947052 | 1.85682351897004 |
| C | 7.73567707092812 | 0.35942847523999 | 0.91253342153474 |
| H | 7.93569352964881 | -0.78031332403839 | 2.73352667884605 |
| H | 6.25018417222022 | -0.56287097130182 | 2.18812918149712 |
| H | 8.78118871789812 | 0.19372516065126 | 0.62557977893292 |

| | | | |
|---|---|---|---|
| H | 7.64404403281648 | 1.32881049651801 | 1.39064133395568 |
| N | 3.10433865794682 | -0.47278109036712 | -1.36424837112094 |
| C | 2.88500539163091 | -1.47611906394978 | -3.55771300560506 |
| C | 1.80555585535679 | -2.21885930878353 | -3.15316122791876 |
| C | 1.33090811458097 | -2.10718073264154 | -1.81625895109735 |
| C | 2.02303633854779 | -1.20750208353252 | -0.94599585655653 |
| H | 3.26646837177932 | -1.53643136322483 | -4.57234534246709 |
| H | 1.30443788022477 | -2.89189053032690 | -3.84443358200431 |
| C | 1.58061365804896 | -1.06962172396716 | 0.39505823835238 |
| C | 0.49721876623940 | -1.79484661504290 | 0.84904188200745 |
| C | -0.18889562020688 | -2.68552793303038 | -0.01289633737592 |
| C | 0.22091250558227 | -2.83883549705039 | -1.32180969807749 |
| H | 2.11560914514536 | -0.38316832709569 | 1.04467563242959 |
| H | 0.16465672081397 | -1.68352595524216 | 1.87739097663747 |
| H | -1.03937468899033 | -3.24751852299186 | 0.36203978866715 |
| H | -0.29890829030980 | -3.52008110145394 | -1.99083115357604 |

## 3b (3-((4-chlorophenyl)thio)-1-methyl-4-morpholino-1H-pyrrole-2,5-dione)

| | | | |
|---|---|---|---|
| C | -1.86952763653305 | 1.15578763955455 | 0.50300485284769 |
| C | -0.57174856590670 | 0.71224571668636 | 0.28180404828640 |
| C | 0.15073943748660 | 1.84007355266116 | -0.48749033553151 |
| C | -2.00042427259454 | 2.50763900036783 | -0.05567688646095 |
| N | -0.75840380486173 | 2.85437389670127 | -0.63033351827277 |
| O | 1.28820247803696 | 1.83871882755774 | -0.93377643053473 |
| O | -2.96190145535527 | 3.26246300117813 | -0.05306573046750 |
| S | -3.15467428460386 | 0.50034506004370 | 1.50805031036163 |
| N | 0.04511317220340 | -0.44664300148778 | 0.55227271136540 |
| C | -0.67324814495469 | -1.67270715347647 | 0.93762929828905 |

| | | | |
|---|---|---|---|
| C | -0.14967933518275 | -2.84543225917843 | 0.10126470056432 |
| H | -1.73948040033624 | -1.55097428135319 | 0.76349178806905 |
| H | -0.51019887956673 | -1.86670231989355 | 2.00513109756139 |
| C | 1.51081737576574 | -0.61837694635797 | 0.63588971955689 |
| C | 1.94239497078095 | -1.82946901431325 | -0.18810002193551 |
| H | 1.75762974071957 | -0.79255038116918 | 1.69139226729105 |
| H | 2.01261663251034 | 0.27995027829113 | 0.28995364389273 |
| O | 1.25808143445390 | -3.00725668943724 | 0.23324817720570 |
| H | 3.01075634403026 | -2.01180858597812 | -0.04338098749630 |
| H | 1.75438132167339 | -1.63977344615696 | -1.25612442014980 |
| H | -0.41776807089328 | -2.68823485508327 | -0.95504903915464 |
| H | -0.61368137772767 | -3.77274559130573 | 0.44866370882167 |
| C | -4.35443313336284 | -0.17948097097870 | 0.35258254902605 |
| C | -4.10997277341735 | -0.32889128917367 | -1.01682890183147 |
| C | -0.50481080197763 | 4.10761879850296 | -1.32516835498440 |
| H | 0.52109624962696 | 4.08438661342485 | -1.69245639589362 |
| H | -0.63104477612162 | 4.95228364741263 | -0.64306254438412 |
| H | -1.19281048883461 | 4.21955411930376 | -2.16707249218799 |
| C | -5.58599771898500 | -0.58818918181157 | 0.88666509012479 |
| C | -6.56389477241205 | -1.14206775721017 | 0.06184685356287 |
| C | -5.08714732808336 | -0.87828585140836 | -1.85047518133307 |
| C | -6.30355190355321 | -1.27978530030950 | -1.30234129866212 |
| H | -4.89720485131695 | -0.99182848539563 | -2.91267079385646 |
| H | -5.78924455548299 | -0.47367181330341 | 1.94806094265187 |
| H | -7.51574836258116 | -1.45702872613527 | 0.47637492939385 |
| H | -3.16049025225769 | -0.01866954857089 | -1.44109173648493 |
| Cl | -7.54184121038501 | -1.97441670219777 | -2.35364161925050 |

### 3c (3-(ethylthio)-1-methyl-4-morpholino-1H-pyrrole-2,5-dione)

| | | | |
|---|---|---|---|
| C | -7.20025249154747 | 2.59559265590724 | -0.07925150166465 |
| C | -7.20077537807120 | 1.27202492494789 | 0.32964158590598 |
| C | -5.80690020817770 | 0.80577078690729 | 0.38235223780812 |
| N | -4.97904683142789 | 1.87641451065790 | -0.02264263481389 |
| C | -3.53425479845090 | 1.78561591399475 | -0.15522393081440 |
| H | -3.07764604819229 | 1.55047595608929 | 0.80976844313553 |
| H | -3.17044956460582 | 2.75090094690291 | -0.50792101562962 |
| H | -3.26575704815093 | 1.00741848957436 | -0.87484016232661 |
| C | -5.72950575295231 | 2.98075651850598 | -0.33369830858774 |
| O | -5.36394348231654 | -0.28614182283817 | 0.70549606655898 |
| O | -5.29149628488047 | 4.03448417131088 | -0.77043444443065 |
| N | -8.19089312543402 | 3.47463660969122 | -0.33005465069560 |
| C | -8.02219514351936 | 4.93853681103530 | -0.37720027545184 |
| C | -8.94212359437988 | 5.58517211430998 | 0.66315204858320 |
| H | -8.29789086362282 | 5.28912808552982 | -1.38011736407061 |
| H | -6.98612700242820 | 5.20500227904879 | -0.19032202527301 |
| C | -9.60050110462704 | 3.08517011378128 | -0.48254124905773 |
| C | -10.46573600539365 | 3.80223563837546 | 0.55536332321291 |
| H | -9.70332225665491 | 2.00658124501209 | -0.38555300722997 |
| H | -9.91733897060245 | 3.38850506248224 | -1.48920940258033 |
| O | -10.30338806009599 | 5.21515838952627 | 0.47003308122412 |
| H | -10.20494321741676 | 3.44729433877464 | 1.56490085991294 |
| H | -11.52319618019534 | 3.59227864888552 | 0.37122363951185 |
| H | -8.89368629386735 | 6.67419998312157 | 0.57119307368752 |
| H | -8.60983423960843 | 5.30056342956205 | 1.67441344581462 |
| S | -8.49037050952650 | 0.23044782699694 | 0.93685066729167 |

| | | | |
|---|---|---|---|
| C | -8.56984364972881 | -1.09206270806162 | -0.36117252191637 |
| C | -9.58506426156674 | -2.15557603578266 | 0.05061992973484 |
| H | -7.56690575331925 | -1.51462898564772 | -0.45468602679569 |
| H | -8.84758659828431 | -0.62243410958857 | -1.30905003027175 |
| H | -9.30735957852508 | -2.62619331094436 | 0.99918757732895 |
| H | -9.62940152560268 | -2.93763930330050 | -0.71461614700147 |
| H | -10.58925417682666 | -1.73240917476811 | 0.15872871890071 |

# Appendix- TD-DFT Geometries and absorbance values of conformers (*in vacuo*)

## 1k conformers

| **Conformer (1K)** | **Absorbance Wavelength (nm)** |
|---|---|
| **1** | 345.5 |
| **2** | 345.5 |
| **3** | 356.0 |
| **4** | 357.3 |
| **5** | 345.4 |

**Experimental** = 367 nm. **Initial TD-DFT (B3LYP 6-31G**)** = 357.6 nm.

| **1K-1** | | | |
|---|---|---|---|
| C | -0.63316300212091 | 1.87657114661161 | 0.02642947479148 |
| C | 0.82506020342711 | 1.89296330167156 | 0.46021312297251 |
| N | -1.38449844073102 | 1.73359021742197 | 1.16579588811428 |
| C | -0.53920336155665 | 1.64804735839832 | 2.31456152882213 |
| C | 0.84414906426645 | 1.75267386178095 | 1.81502338248067 |
| Cl | 2.17959390969394 | 1.67914124213413 | 2.91492559530499 |
| N | 1.72267464360877 | 2.00743476160336 | -0.53385139791679 |
| C | -2.83629602497021 | 1.60587067675585 | 1.21512229151571 |
| H | -3.26514547962431 | 2.32820775123007 | 0.51236869560468 |
| H | -3.13456717908313 | 1.89024876013068 | 2.22945852652505 |
| C | -3.31814985111227 | 0.25523409270291 | 0.90741569181461 |
| O | -1.01805551780542 | 1.99308795250628 | -1.12296001157530 |
| O | -0.95483870477864 | 1.52854281198367 | 3.44603202870757 |
| C | -3.73023056050937 | -0.85199614918229 | 0.66183835831693 |
| H | -4.08366618497700 | -1.83274941061608 | 0.44042827610839 |
| H | 1.28828520928082 | 2.13725160470226 | -1.44197768295949 |

| | | | |
|---|---|---|---|
| C | 3.15068100381640 | 2.30510341745317 | -0.39867753430572 |
| H | 3.53462219787165 | 1.78897331208151 | 0.48309335610676 |
| C | 3.44100762872528 | 3.78559234492020 | -0.31387784093285 |
| H | 3.64688511739862 | 1.87050252178555 | -1.27516511873998 |
| C | 4.07343915343561 | 4.36423308085988 | 0.70585778787028 |
| H | 3.10165565674757 | 4.38601730688171 | -1.15827983763035 |
| H | 4.41266215054740 | 3.79180548614194 | 1.56566342296878 |
| H | 4.27243836844925 | 5.43186255004077 | 0.72015199603565 |

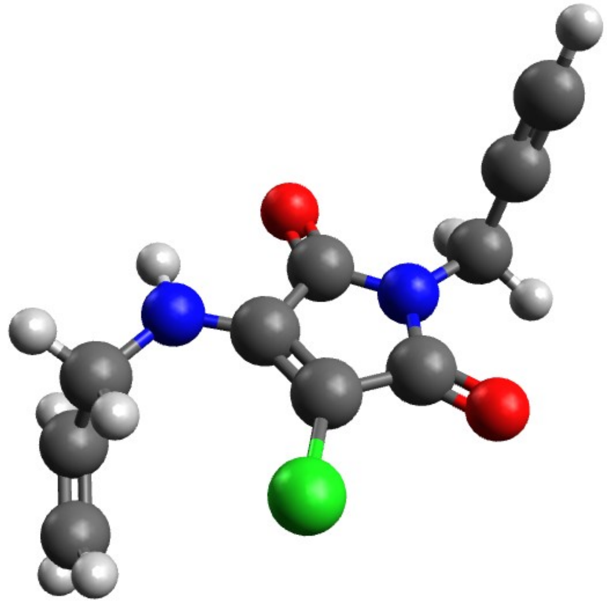

FINAL SINGLE POINT ENERGY -1105.972012164286 (Eh)

| 1K-2 | | | |
|---|---|---|---|
| C | -0.42236611242093 | 1.78995657287498 | 0.27877949691390 |
| C | 0.97944545759161 | 1.48458681582667 | 0.78522251908100 |
| N | -1.27581267588892 | 1.60433778031510 | 1.33741689310169 |
| C | -0.55106301201871 | 1.19009937014638 | 2.49719691558121 |
| C | 0.86638121082467 | 1.12965580817256 | 2.09551673115355 |
| Cl | 2.07998616557625 | 0.65133540569964 | 3.23437802298275 |
| N | 1.96094733039718 | 1.59870873687690 | -0.12618588568703 |
| C | -2.72876648646570 | 1.71688049635244 | 1.28795280253337 |
| H | -2.98175054176000 | 2.60436005274809 | 0.69799034523338 |
| H | -3.05834843044074 | 1.88103090543368 | 2.31902971050306 |
| C | -3.39093903519667 | 0.53379685135947 | 0.72833124895777 |
| O | -0.68992124446839 | 2.14881586479778 | -0.85360456774762 |
| O | -1.07038235211620 | 0.96619536165370 | 3.56838119160756 |
| C | -3.94731549971938 | -0.43139064147941 | 0.26446924036173 |
| H | -4.43112793077985 | -1.29012549039050 | -0.14108350822837 |
| H | 1.62804540580873 | 1.94356431315218 | -1.02087174527632 |
| C | 3.40275361620440 | 1.62207279114538 | 0.13293042782620 |
| H | 3.62689327217946 | 0.91467174187778 | 0.93338177137904 |
| C | 3.91887321686917 | 2.99837807517075 | 0.48273632806956 |
| H | 3.89162574064493 | 1.25308816281525 | -0.77707849414319 |
| C | 4.55106885491461 | 3.29162376618933 | 1.61809586420314 |
| H | 3.74991192498102 | 3.77384166496284 | -0.26482947869253 |
| H | 4.72335752486146 | 2.54114262970161 | 2.38565075002948 |
| H | 4.91662360042197 | 4.29354296459732 | 1.82336342025662 |

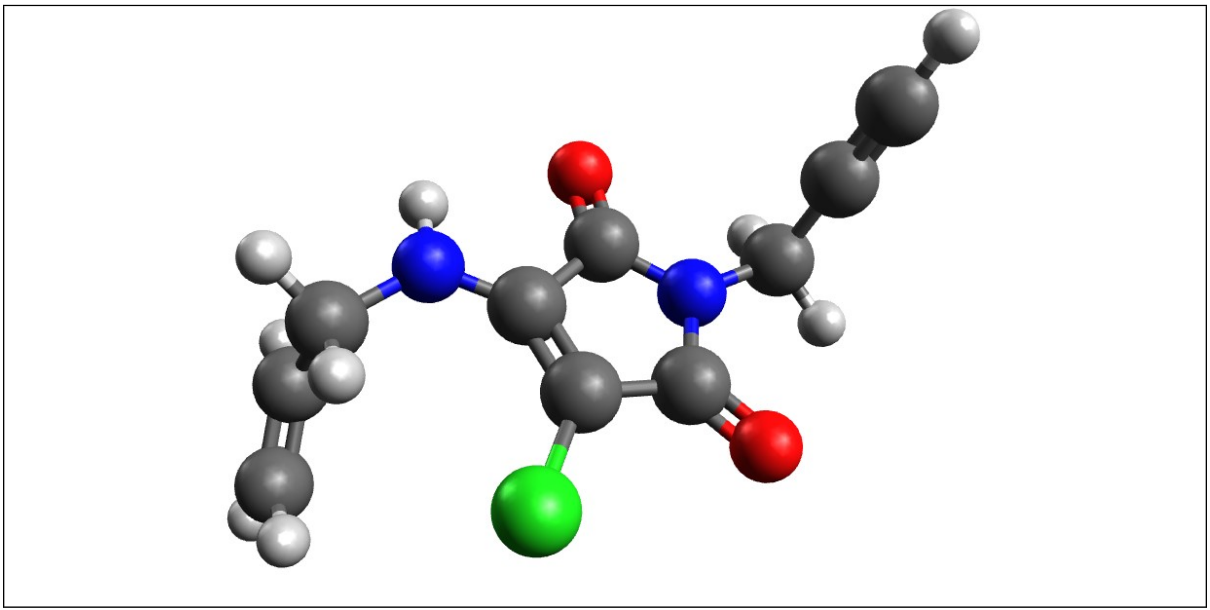

FINAL SINGLE POINT ENERGY -1105.972004690359 (Eh)

| 1K-3 | | | |
|---|---|---|---|
| C | -0.48613324594624 | 2.44624939691355 | -0.49485218102455 |
| C | 1.02660787334360 | 2.45517139808886 | -0.29651307330864 |
| N | -1.02846114190293 | 2.13008352016394 | 0.73228829089799 |
| C | -0.01249823244539 | 1.90710550413813 | 1.71023890285293 |
| C | 1.25512541983113 | 2.12665792632659 | 1.00757319381571 |
| Cl | 2.79810713980092 | 2.00876424657150 | 1.77745606974658 |
| N | 1.92242270751977 | 2.75823958216523 | -1.25461590289948 |
| C | -2.45033049796743 | 1.92453111430641 | 0.98330430344317 |
| H | -3.00393418164181 | 2.70371654433915 | 0.44910423002955 |
| H | -2.59609509416808 | 2.06922552992810 | 2.05856053599195 |
| C | -2.93350254624675 | 0.59879047401699 | 0.58237562490550 |
| O | -1.10798468610723 | 2.67863388998878 | -1.51588302622506 |
| O | -0.23852790596522 | 1.61468607364685 | 2.86448231876392 |
| C | -3.34676550823395 | -0.48749628546348 | 0.25772463819284 |
| H | -3.70444930909176 | -1.44893711653601 | -0.03124920323778 |
| H | 2.89399960322689 | 2.71090992197133 | -0.97159147555864 |
| C | 1.67005706194125 | 3.03130460591096 | -2.66546523847088 |

| | | | |
|---|---|---|---|
| H | 1.99438784802967 | 2.16933516743043 | -3.26913661497541 |
| C | 2.37163344754659 | 4.27075837128121 | -3.16153440697447 |
| H | 0.58961439941739 | 3.11762747644106 | -2.80562730327520 |
| C | 2.96409070774944 | 5.19723586474249 | -2.40891464098869 |
| H | 2.36782388955329 | 4.37705737157893 | -4.24587537534389 |
| H | 2.98097305063061 | 5.13570172331110 | -1.32429011390648 |
| H | 3.44465920112620 | 6.06113769873769 | -2.85689955245089 |

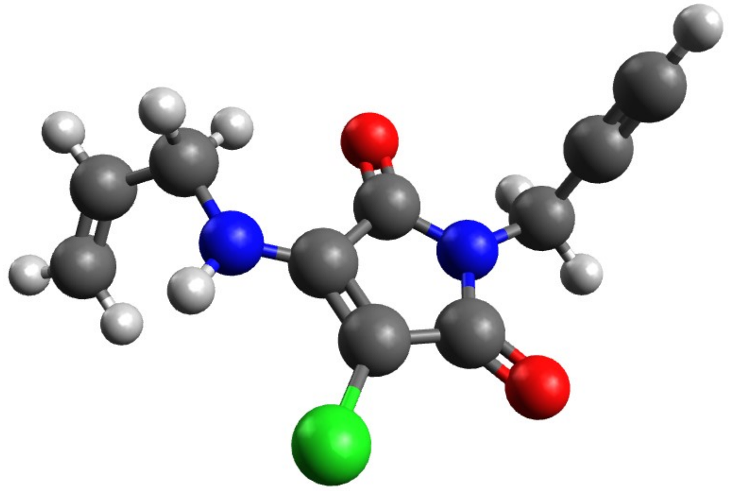

FINAL SINGLE POINT ENERGY -1105.974910494450 (Eh)

| **1K-4** | | | |
|---|---|---|---|
| C | -0.54258798426379 | 2.52879606399548 | 0.31077948444319 |
| C | 0.96262313841691 | 2.47478701355091 | 0.55505161344968 |
| N | -1.12198590722387 | 1.84125693197853 | 1.35569836381893 |
| C | -0.13733255044061 | 1.32752904278697 | 2.25276881088854 |
| C | 1.15026300939602 | 1.75935621212856 | 1.70159176517222 |
| Cl | 2.66704069926190 | 1.40808228808050 | 2.45207452868669 |
| N | 1.88583780615608 | 3.06435957152535 | -0.22758647292764 |
| C | -2.54899308204910 | 1.56873153183178 | 1.48102668519137 |
| H | -3.09242357557565 | 2.47367339269469 | 1.18985317308884 |
| H | -2.73385552961704 | 1.37837770178429 | 2.54307503854577 |
| C | -3.00338619600652 | 0.42820385704028 | 0.67807553428961 |

| | | | |
|---|---|---|---|
| O | -1.13338578784889 | 3.07455215650340 | -0.60379914220821 |
| O | -0.39914904296333 | 0.68826176771933 | 3.24868775281130 |
| C | -3.39363057004367 | -0.50838238865991 | 0.02484983426104 |
| H | -3.72763023417808 | -1.33933506246573 | -0.55285729656213 |
| H | 2.84769146285971 | 2.93687824840242 | 0.06349735877757 |
| C | 1.68187282914790 | 3.74670300615630 | -1.51155715800340 |
| H | 0.60957265895863 | 3.91724877161879 | -1.62438430871341 |
| C | 2.44711447516155 | 5.04242600571818 | -1.54878567735941 |
| H | 2.00498154889177 | 3.09455110986202 | -2.33468251384439 |
| C | 3.40503024402188 | 5.31659037711263 | -2.43436506535116 |
| H | 2.17310661542963 | 5.77626559598653 | -0.79141036638328 |
| H | 3.69885364113177 | 4.59855200013204 | -3.19715205164007 |
| H | 3.92255233137676 | 6.27124480451645 | -2.43860989043168 |

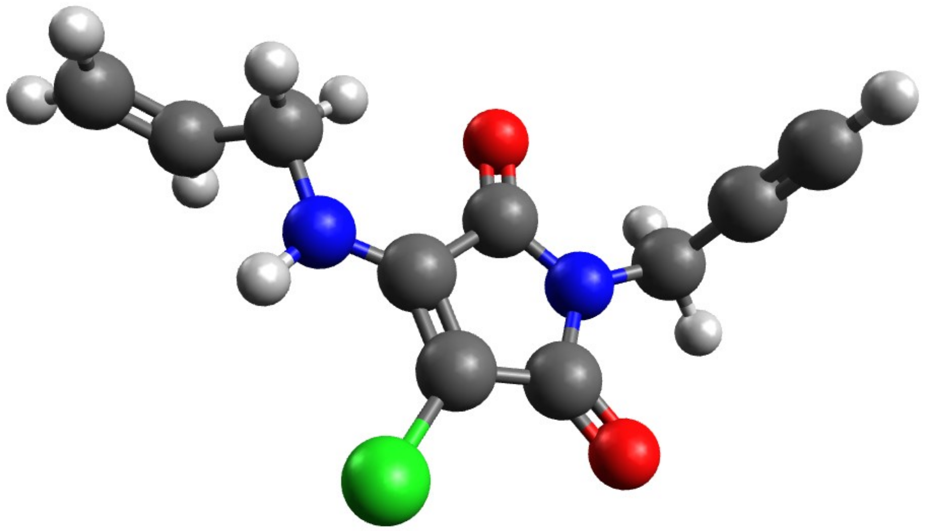

FINAL SINGLE POINT ENERGY -1105.976052224298 (Eh)

| 1K-5 | | | |
|---|---|---|---|
| C | -0.40549851196810 | 2.02781903986605 | -0.30080043762172 |
| C | 0.76497743169886 | 2.90539718624270 | 0.11524504478784 |
| N | -1.32594284753252 | 2.08511427232112 | 0.71601745320810 |
| C | -0.84175226109249 | 2.90468696259932 | 1.78204339611836 |
| C | 0.47706867190229 | 3.40324796407612 | 1.34990251540768 |
| Cl | 1.37397947536860 | 4.49246846373718 | 2.35382487124499 |
| N | 1.75247100519231 | 3.02080176594898 | -0.79023767520463 |
| C | -2.56528381250290 | 1.31949085173073 | 0.78242524265905 |
| H | -3.03638315050282 | 1.35160849155082 | -0.20557404038663 |
| H | -3.21234701928846 | 1.84451162688552 | 1.49242415035530 |
| C | -2.37556915696667 | -0.07208507519643 | 1.20548104522772 |
| O | -0.47580375207653 | 1.41216178180627 | -1.34889523118444 |
| O | -1.46101039851522 | 3.11508170460688 | 2.80154054005885 |
| C | -2.23693707535086 | -1.21964985523338 | 1.55193869388897 |
| H | -2.10315237655143 | -2.23042520733915 | 1.86212184309638 |
| H | 1.61059230584533 | 2.42695770596290 | -1.60120214556942 |
| C | 3.09397280535444 | 3.56280064264979 | -0.55833185190849 |
| H | 3.01334724691355 | 4.43195676687829 | 0.09726080483142 |
| C | 4.04936194175504 | 2.54707134335085 | 0.02276338756472 |
| H | 3.46012618337589 | 3.91807695744434 | -1.52932301977422 |
| C | 4.69479599914481 | 2.70378468499824 | 1.17750748823913 |
| H | 4.19777116560919 | 1.64181411445281 | -0.56655476636994 |
| H | 4.55757984671741 | 3.59028918383388 | 1.79186155755146 |
| H | 5.38183628347025 | 1.95001862682608 | 1.55063113377949 |

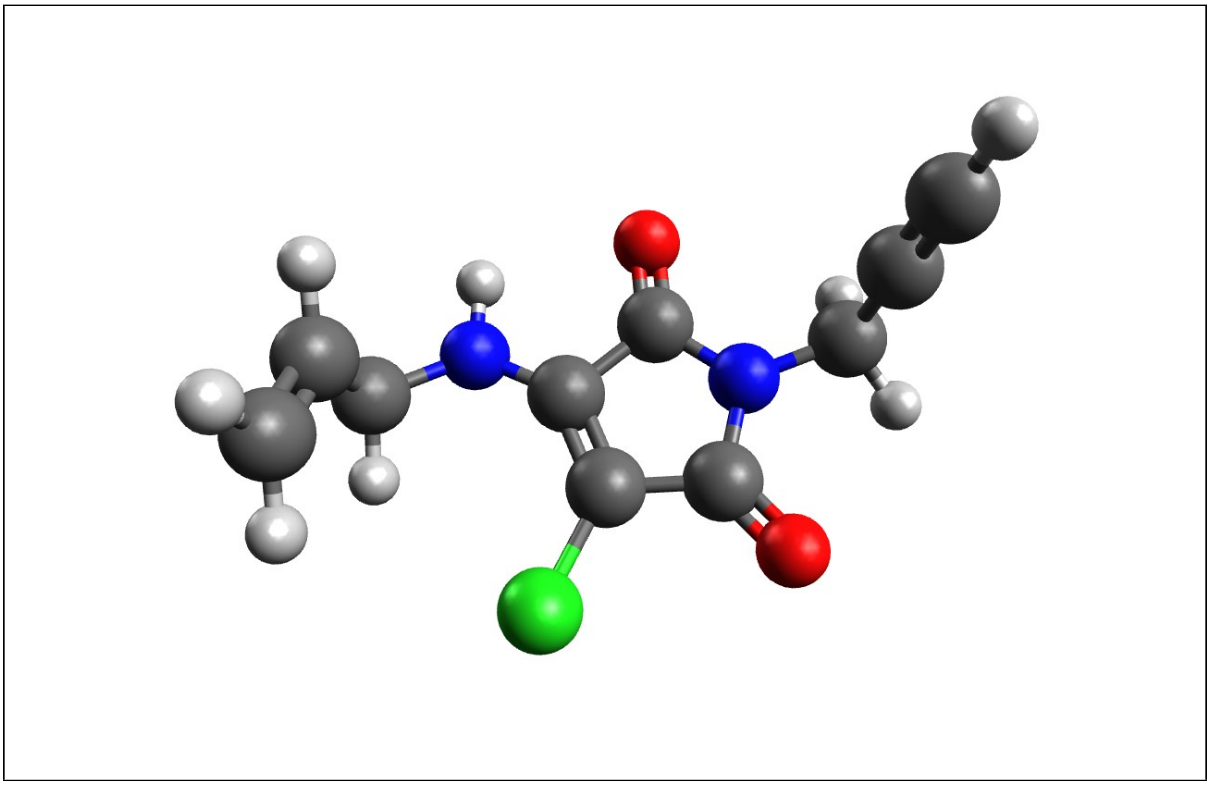

FINAL SINGLE POINT ENERGY -1105.971968306687 (Eh)

## 2b conformers

| Conformer (2b) | Absorbance Wavelength (nm) |
|---|---|
| **1** | 431.3 |
| **2** | 412.9 |
| **3** | 414.5 |
| **4** | 431.2 |
| **5** | 413.9 |

**Experimental** = 410 nm. **Initial TD-DFT (B3LYP 6-31G**)** = 413.6 nm.

| **2b-1** | | | |
|---|---|---|---|
| C | -1.07484595826628 | 2.10759801591368 | -0.38741233616113 |
| C | 0.37611248559536 | 2.48664720041756 | -0.25506178532374 |
| N | -1.63603659146017 | 2.24269070498338 | 0.88167251773800 |
| C | -0.67705559598128 | 2.65511108099437 | 1.80956259820688 |
| C | 0.61922842251846 | 2.77451800603972 | 1.05621997009346 |
| S | 2.13764854569991 | 3.34743392187371 | 1.71227904546934 |
| S | 1.57887076478898 | 2.40880511084879 | -1.52324716574165 |
| C | -3.03206954343554 | 1.99202464651179 | 1.19196400698154 |
| H | -3.33972208127881 | 1.03176269364408 | 0.77139182458614 |
| H | -3.67419331454242 | 2.77800316078190 | 0.78169352263347 |
| H | -3.13020527677049 | 1.97661683693862 | 2.27816744560704 |
| O | -1.68703000406851 | 1.73578403717133 | -1.37175458092355 |
| O | -0.90310322097267 | 2.86830107700477 | 2.98657995338637 |
| C | 2.11171455157716 | 2.87535961216013 | 3.51823530349995 |
| C | 3.32868068001566 | 3.56788237086571 | 4.13945720983405 |
| C | 2.14017095365388 | 1.35930237681545 | 3.71023446028715 |
| H | 1.19048406956898 | 3.28588279292260 | 3.93209279928780 |
| H | 4.26423949389440 | 3.21274155257686 | 3.69281965642771 |
| H | 3.36336689835124 | 3.34303314385565 | 5.21088860347755 |

| | | | |
|---|---|---|---|
| H | 3.28102431814882 | 4.65445836244256 | 4.02232716855570 |
| H | 3.03688369860187 | 0.91921087276403 | 3.26187362968695 |
| H | 1.26186746940910 | 0.88160243689383 | 3.26892743730083 |
| H | 2.14091132471516 | 1.12680165428662 | 4.78218253372490 |
| C | 0.64309702520808 | 2.66660895865592 | -3.11781617382297 |
| C | 1.64989523098971 | 2.37331378764009 | -4.23446070018008 |
| C | 0.05867182959372 | 4.07580033945267 | -3.21249313491594 |
| H | -0.15983695996867 | 1.92930355299025 | -3.12856437228279 |
| H | 2.49813506009691 | 3.06643090257105 | -4.20255319460559 |
| H | 1.15660706481453 | 2.49364568250416 | -5.20495528969511 |
| H | 2.03659698278438 | 1.35180455842187 | -4.17454116072540 |
| H | 0.84469406765752 | 4.83597728221885 | -3.15688090966564 |
| H | -0.66459038057107 | 4.26481851271370 | -2.41545466278648 |
| H | -0.46481201036794 | 4.19059475412416 | -4.16949421995478 |

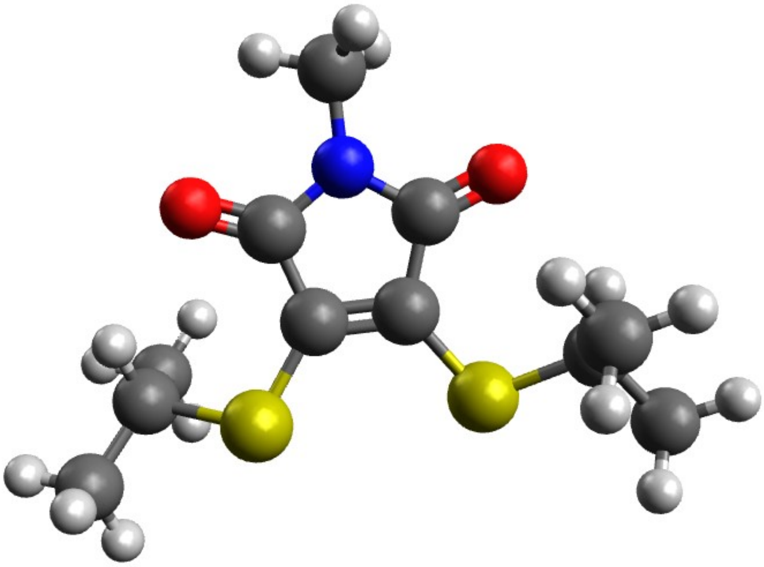

FINAL SINGLE POINT ENERGY -1430.404030720149 (Eh)

| 2b-2 | | | |
|---|---|---|---|
| C | -1.10551023106364 | 1.46239996709372 | -0.17108819409225 |
| C | 0.37422274835710 | 1.30605842203888 | 0.06221414720979 |
| N | -1.74194641789877 | 0.85941722269235 | 0.91305715731321 |
| C | -0.80944071157608 | 0.32073349151776 | 1.80353257850846 |
| C | 0.54851597542717 | 0.63085990065159 | 1.23452747443993 |
| S | 2.07330079222181 | 0.04547228478299 | 1.85963677714464 |
| S | 1.63948688321886 | 1.73173071592673 | -1.06589662011631 |
| C | -3.18048115202933 | 0.83820676000820 | 1.11023047961187 |
| H | -3.67413609163517 | 0.40491485012689 | 0.23668604020509 |
| H | -3.56912983672820 | 1.84897602884593 | 1.26820239353391 |
| H | -3.37956838855672 | 0.22711767355333 | 1.99146319424285 |
| O | -1.68732103168578 | 2.00510058902889 | -1.09243386484725 |
| O | -1.09430449207896 | -0.26730832674677 | 2.83050437022025 |
| C | 1.84839822016667 | -0.00963220574477 | 3.71317027074953 |
| C | 3.10344066566568 | -0.69608911979823 | 4.26028604951237 |
| C | 1.63907546879989 | 1.38500733371895 | 4.30200917525682 |
| H | 0.96880900018486 | -0.62771356364558 | 3.89580258244672 |
| H | 3.02503012245695 | -0.77753408502988 | 5.34977397236807 |
| H | 4.00734443445915 | -0.12002756492160 | 4.03218599705087 |
| H | 3.22478818482221 | -1.70409203394433 | 3.85298704836253 |
| H | 1.52006320057657 | 1.30841616003870 | 5.38971447853478 |
| H | 0.73842544895151 | 1.85858262338909 | 3.90303691681209 |
| H | 2.49463657343828 | 2.03599493684310 | 4.09441741942658 |
| C | 0.98927332212413 | 3.21469912893123 | -1.99649534705259 |
| C | 1.99499240445631 | 3.46440900212389 | -3.12444470503038 |
| C | 0.81802642895796 | 4.42816208668420 | -1.08334959443595 |
| H | 0.02364961222283 | 2.91945343593910 | -2.40791510478401 |

| | | | |
|---|---|---|---|
| H | 2.99010705984552 | 3.69945886339607 | -2.73022341546419 |
| H | 1.66190397264947 | 4.31942423368442 | -3.72253888601731 |
| H | 2.08239137794089 | 2.59961226465303 | -3.78861776172451 |
| H | 1.76932368374964 | 4.71790848166385 | -0.62515894159476 |
| H | 0.09472754713228 | 4.23238820728460 | -0.28774330469393 |
| H | 0.44491522542688 | 5.27693223521356 | -1.66939278309700 |

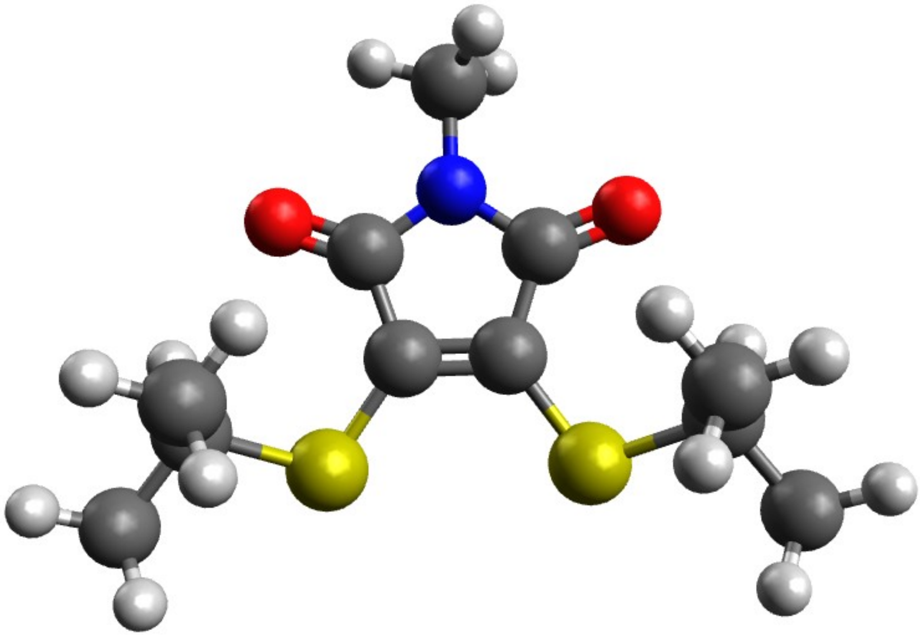

FINAL SINGLE POINT ENERGY -1430.400164850156 (Eh)

| 2b-3 | | | |
|---|---|---|---|
| C | -0.68067483518724 | 1.61607532971541 | 0.48351407273103 |
| C | 0.52455704583059 | 1.38896837517783 | 1.36058675464076 |
| N | -1.76156267211414 | 1.86478032071858 | 1.33029861704988 |
| C | -1.36898045735565 | 1.78348852120355 | 2.66212406780226 |
| C | 0.11193700477137 | 1.45355061067986 | 2.65726782600322 |
| S | 0.81396501732594 | 1.30287269091892 | 4.24798929125223 |
| S | 2.09743141229147 | 0.97596414401316 | 0.68579857716350 |
| C | -3.10286181224245 | 2.19097781107257 | 0.87954165024241 |
| H | -3.47023025356282 | 1.41876141033107 | 0.19893156488838 |
| H | -3.11596144031287 | 3.15352348510333 | 0.35931005306956 |
| H | -3.74051306771083 | 2.24375530531258 | 1.76294681445439 |
| O | -0.74927301591132 | 1.60523574578256 | -0.73138349183889 |
| O | -2.08396928208825 | 1.95975744800184 | 3.62719764589855 |
| C | 2.42923384303970 | 0.39757990833738 | 4.05029698878511 |
| C | 3.14883968490409 | 0.54528403847621 | 5.39559759650412 |
| C | 2.20452026216328 | -1.06680408447500 | 3.66891174517663 |
| H | 2.99630673938360 | 0.91042633881395 | 3.27271158572333 |
| H | 4.10962594261999 | 0.02145050390646 | 5.35058358684658 |
| H | 2.56565989289776 | 0.10352493553527 | 6.21139503503286 |
| H | 3.34248043708254 | 1.59384367744594 | 5.63910389768387 |
| H | 3.17264587126565 | -1.56903316580701 | 3.55443198485810 |
| H | 1.66500061144595 | -1.16537824839643 | 2.72342054970526 |
| H | 1.63517754247026 | -1.58908088947560 | 4.44461685760425 |
| C | 2.33082622157726 | 2.23718347086184 | -0.67415300916175 |
| C | 3.58514571121577 | 1.80694262478350 | -1.43910194403153 |
| C | 2.43999745822645 | 3.65561579148030 | -0.11664540623421 |
| H | 1.45235383802475 | 2.14490159035314 | -1.31464500221935 |

| | | | |
|---|---|---|---|
| H | 4.47289864742492 | 1.82563204989343 | -0.79661573736120 |
| H | 3.75944740435606 | 2.50046741194659 | -2.26892625223521 |
| H | 3.47987668816189 | 0.80072042719781 | -1.85491387893428 |
| H | 3.30857612229990 | 3.75624002503180 | 0.54269369360964 |
| H | 1.54782164821458 | 3.93736590547532 | 0.44985080138899 |
| H | 2.55118178949174 | 4.36879649058379 | -0.94267653609858 |

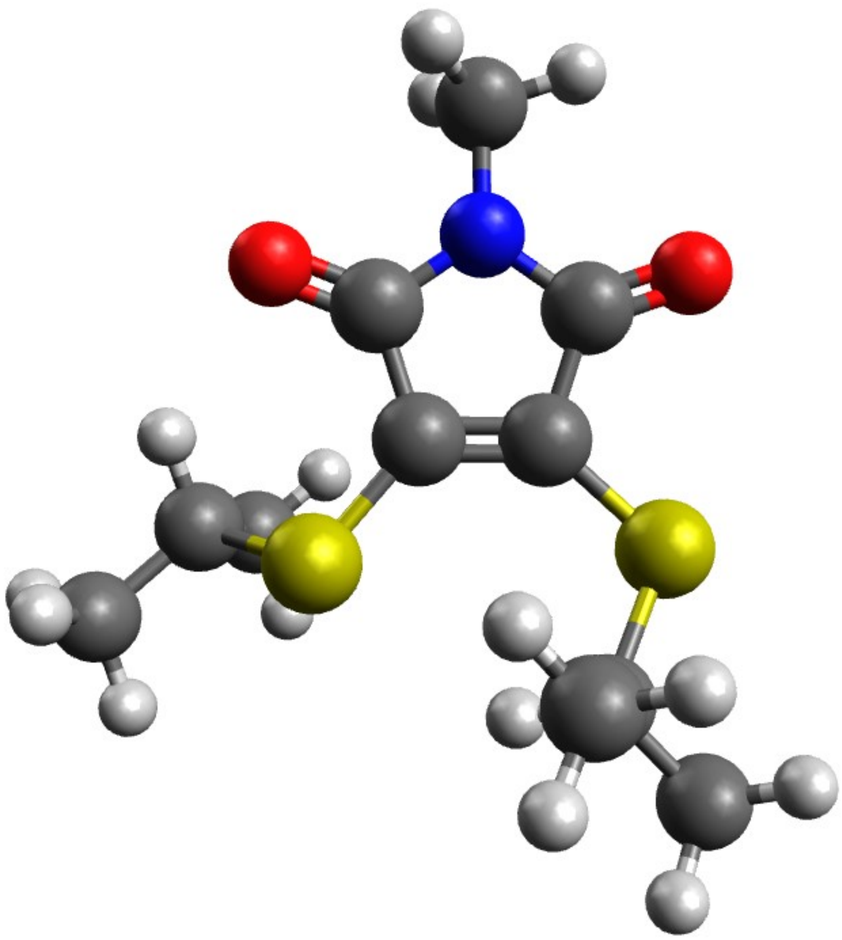

FINAL SINGLE POINT ENERGY -1430.396233896126 (Eh)

| 2b-4 | | | |
|---|---|---|---|
| C | -0.90503203160295 | 0.48112825533553 | 0.72219091874460 |
| C | 0.43369354885051 | 0.04535080430795 | 1.25555571465406 |
| N | -1.67260794655144 | 0.84309481558525 | 1.82799023656113 |
| C | -0.94443295775546 | 0.69337647582797 | 3.01075800541299 |
| C | 0.42135150251011 | 0.20831535801959 | 2.61010536639920 |
| S | 1.71372916317760 | -0.23297690412059 | 3.70538507098960 |
| S | 1.81870946894138 | -0.42735388919933 | 0.29703666067810 |
| C | -3.04681048738958 | 1.30595084910155 | 1.75565863700305 |
| H | -3.71901795419218 | 0.49456604314961 | 1.45934969172234 |
| H | -3.13148492907927 | 2.11596518417499 | 1.02707150729130 |
| H | -3.32515298027637 | 1.66258011565434 | 2.74813087197465 |
| O | -1.29254487915979 | 0.54116778829159 | -0.43035246954568 |
| O | -1.38207771328544 | 0.91957703095675 | 4.12396223737406 |
| C | 1.48056393284841 | 0.84600542343659 | 5.21118806551869 |
| C | 2.47298910404373 | 0.31717525278970 | 6.25124788888131 |
| C | 1.70070144704072 | 2.32413203227299 | 4.89067329443319 |
| H | 0.45657070012264 | 0.68676734903898 | 5.54956660783633 |
| H | 2.37491024467289 | 0.90084611929693 | 7.17288514021851 |
| H | 3.50774215750896 | 0.41270790966004 | 5.90336767076267 |
| H | 2.28583732730292 | -0.73305983055174 | 6.49339859094362 |
| H | 1.57234405688837 | 2.91901511429985 | 5.80323588958665 |
| H | 0.97871971925770 | 2.68759627087119 | 4.15517499746275 |
| H | 2.70975618822727 | 2.50034897566791 | 4.50399439566336 |
| C | 1.12046713865507 | -1.12410778060259 | -1.28803506475916 |
| C | 2.32595929923270 | -1.32023985905529 | -2.21277111720578 |
| C | 0.35013638812113 | -2.42223631238765 | -1.04798275144417 |
| H | 0.45165777813431 | -0.36434853659854 | -1.69276126321579 |

| | | | |
|---|---|---|---|
| H | 3.04230364221493 | -2.03502433180978 | -1.79223641254822 |
| H | 2.84930510423394 | -0.37869334191286 | -2.40301387621258 |
| H | 1.98219024243712 | -1.71797562367259 | -3.17365548477047 |
| H | 0.99102123569849 | -3.18677452458582 | -0.59687017130468 |
| H | -0.02400298495857 | -2.80673179180158 | -2.00470202012080 |
| H | -0.51241452586989 | -2.26246444144095 | -0.39644682898492 |

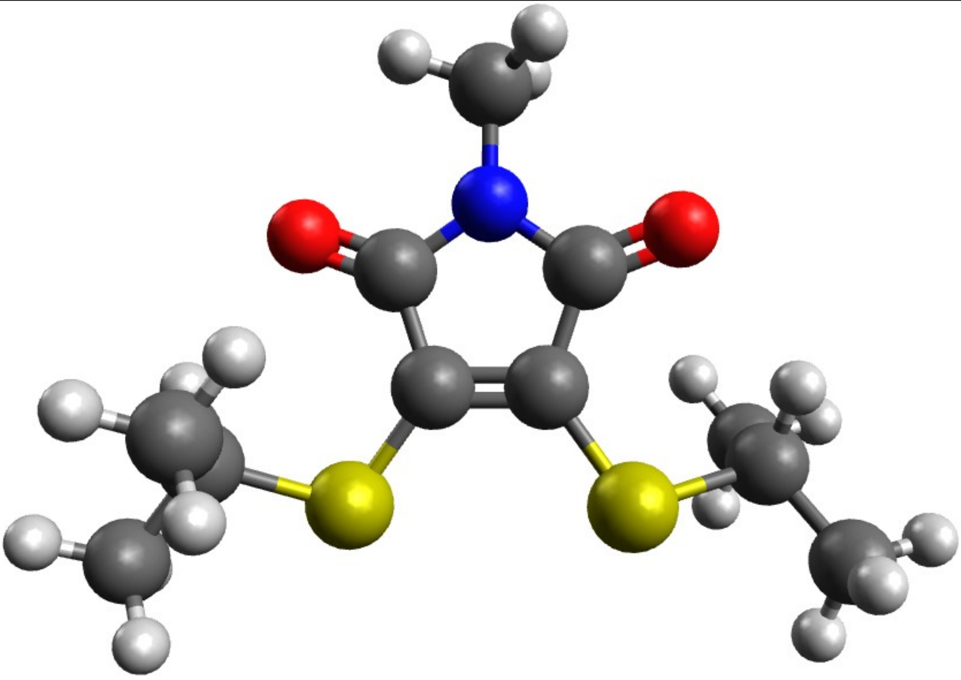

FINAL SINGLE POINT ENERGY -1430.404020928339 (Eh)

| 2b-5 | | | |
|---|---|---|---|
| C | -0.86242551126156 | 0.44028637863800 | 0.34838100812975 |
| C | 0.56128067846550 | 0.44541328068652 | 0.84760723195048 |
| N | -1.64536207339959 | 0.92397826224781 | 1.39204093696967 |
| C | -0.85632159110732 | 1.25647036186547 | 2.49878744719940 |
| C | 0.56149524651160 | 0.93133108969373 | 2.12380539229787 |
| S | 2.00999293443381 | 1.32119259551297 | 3.03006732130138 |
| S | 1.98158563251292 | 0.07569764657256 | -0.09875834992100 |
| C | -3.09309089470447 | 1.03047765719720 | 1.34744404039942 |
| H | -3.55112811084108 | 0.04568236721691 | 1.21593858687377 |
| H | -3.40167205557231 | 1.67453816179498 | 0.51966824060683 |
| H | -3.41621705107439 | 1.46437382403961 | 2.29447983864675 |
| O | -1.29944435155149 | 0.08443509729302 | -0.73072048217692 |
| O | -1.29509110764961 | 1.70547154569076 | 3.54133449462471 |
| C | 1.70989544424613 | 0.91016963630954 | 4.83366795188929 |
| C | 0.92980706128219 | -0.38885881468844 | 5.03647639118008 |
| C | 1.14533139814312 | 2.09168364582316 | 5.62543996764364 |
| H | 2.75106784747158 | 0.74380855083072 | 5.13791347095292 |
| H | 0.95613420183396 | -0.66074882104677 | 6.09826518737470 |
| H | 1.36056997945167 | -1.21366646708160 | 4.46199826607024 |
| H | -0.11799090000445 | -0.26475959022099 | 4.75272949263987 |
| H | 1.14349367058066 | 1.84388010796709 | 6.69444146434777 |
| H | 0.12333893184826 | 2.31209807490489 | 5.31326366885950 |
| H | 1.75660786790903 | 2.98816869532137 | 5.48738319041008 |
| C | 1.41286332248133 | -1.12262837469250 | -1.41379690936210 |
| C | 2.59861135626481 | -1.26545217233670 | -2.37315719009803 |
| C | 0.97810716114271 | -2.46046596703148 | -0.81620954995832 |
| H | 0.57050848347253 | -0.65057482439238 | -1.91978670857778 |

| | | | |
|---|---|---|---|
| H | 3.47753419456150 | -1.68028563726568 | -1.86679436803246 |
| H | 2.87813371882076 | -0.30632666609475 | -2.81877505398384 |
| H | 2.32609826677961 | -1.94998735824914 | -3.18361343306866 |
| H | 1.79626894486337 | -2.93320034628722 | -0.26301598523246 |
| H | 0.67137858640550 | -3.13773289417597 | -1.62261535248661 |
| H | 0.12525871768370 | -2.34072904604272 | -0.14377020747000 |

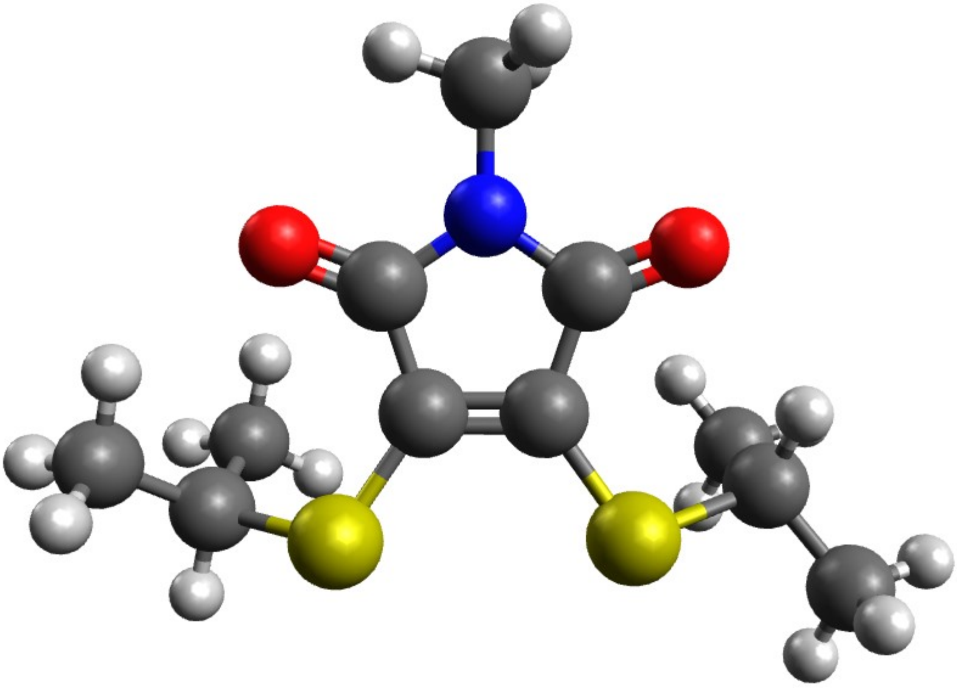

FINAL SINGLE POINT ENERGY -1430.397032094124 (Eh)

## 3c conformers

| Conformer | Absorbance Wavelength (nm) |
|---|---|
| **3c-1** | 403.6 |
| **3c-2** | 398.6 |
| **3c-3** | 404.3 |
| **3c-4** | 404.2 |
| **3c-5** | 405.3 |

**Experimental** = 395 nm. **Initial TD-DFT (B3LYP 6-31G**)** = 386.6 nm.

| **3c-1** | | | |
|---|---|---|---|
| C | -1.25046604050512 | 2.27465699756137 | -0.06057260157547 |
| C | 0.28385863362811 | 2.30983988425750 | 0.00272324522507 |
| N | -1.66982240311704 | 1.54476303839388 | 1.02318768526203 |
| C | -0.56733426960546 | 1.13427373270764 | 1.80959924085820 |
| C | 0.65675412589842 | 1.63788643659739 | 1.14958358509590 |
| S | 2.24587817567540 | 1.14822846629199 | 1.74402022162118 |
| N | 0.99127901327502 | 2.98674387688436 | -0.93584379365147 |
| C | -3.06018274915030 | 1.32200574293501 | 1.37392118498706 |
| H | -3.57252604826691 | 0.77162337165381 | 0.57985626325006 |
| H | -3.57852110833676 | 2.27265424977104 | 1.53186618875587 |
| H | -3.06938503358832 | 0.73637148245194 | 2.29401069040708 |
| O | -1.98952342352455 | 2.81188687051123 | -0.87008825427764 |
| O | -0.68182376156393 | 0.48412112882421 | 2.83266718916410 |
| C | 2.22420466400049 | 1.78121404767245 | 3.48443939333031 |
| C | 3.49982933885444 | 1.33682091006766 | 4.19688985070227 |
| H | 1.33500665725892 | 1.37172648438674 | 3.96656434514573 |
| H | 2.14572340357867 | 2.87231388167916 | 3.45669596932083 |
| H | 3.57719764062278 | 0.24559837308194 | 4.22734455002039 |

| | | | |
|---|---|---|---|
| H | 3.49356519290615 | 1.70344526418572 | 5.22884473307331 |
| H | 4.39674716321262 | 1.72895976239798 | 3.70592540100056 |
| C | 0.52250163071545 | 3.21725532126673 | -2.31363031399316 |
| C | 2.39951279213569 | 3.35791098022473 | -0.76037139928971 |
| H | 2.68321589766226 | 3.24787448868154 | 0.28488166055963 |
| C | 2.59864934692450 | 4.80411454501441 | -1.22428601648524 |
| H | 3.03636198727447 | 2.68822534898177 | -1.35691994917724 |
| H | -0.53940799917091 | 2.99681913862550 | -2.38500113135486 |
| H | 1.08442171209718 | 2.54906834107304 | -2.98344423256165 |
| C | 0.79687386108665 | 4.66623652609963 | -2.71343138612084 |
| O | 2.17348322680962 | 4.99252250091307 | -2.56498161609363 |
| H | 3.66146727570765 | 5.06250421353420 | -1.19148162099692 |
| H | 2.05048077173422 | 5.48236047559719 | -0.54915882198900 |
| H | 0.17378041338790 | 5.33967003116788 | -2.10301112656762 |
| H | 0.55032991238265 | 4.82018408650719 | -3.76801913364513 |

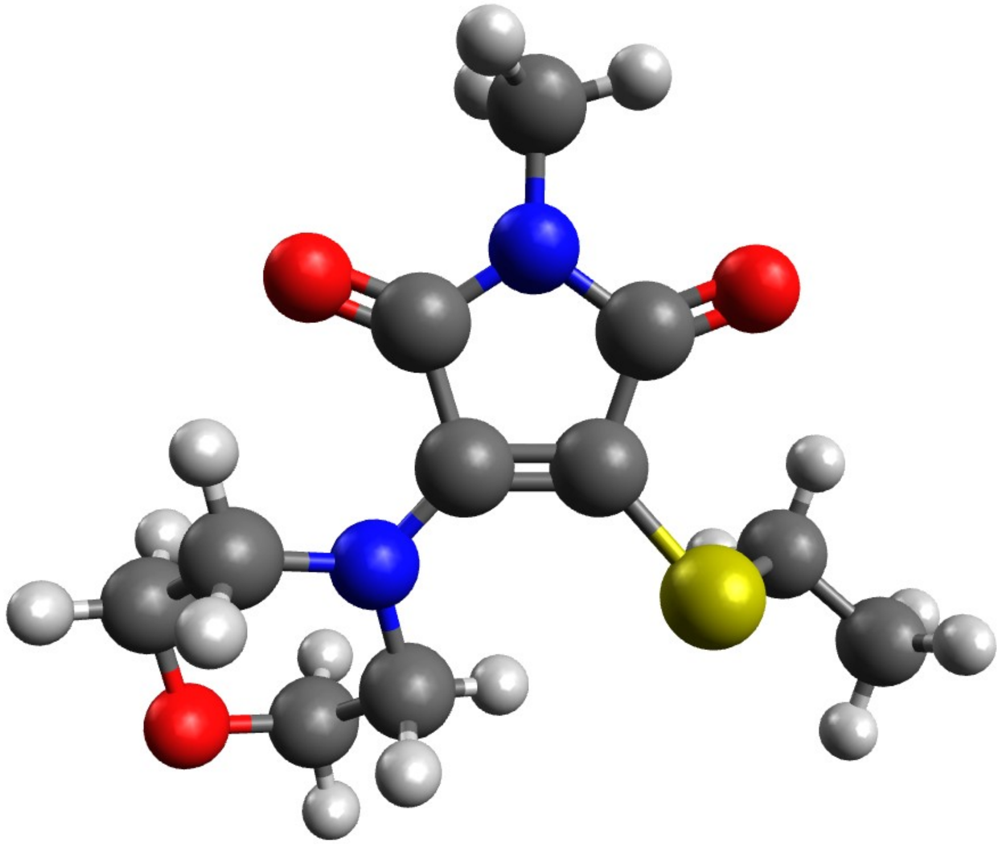

FINAL SINGLE POINT ENERGY     -1161.559148415459 (Eh)

| 3c-2 | | | |
|---|---|---|---|
| C | -1.39316682532904 | 2.27971084975710 | -0.45210698483808 |
| C | 0.12663703567249 | 2.29022119748944 | -0.22666820392665 |
| N | -1.93871754098106 | 1.59100183849799 | 0.60113049431095 |
| C | -0.93377736256154 | 1.17802591887136 | 1.50819957871885 |
| C | 0.36298918478812 | 1.64341457911823 | 0.97115897661973 |
| S | 1.88293194528541 | 1.13977026517526 | 1.71662079772023 |
| N | 0.93966450481563 | 2.93134975989069 | -1.10182299505480 |
| C | -3.36187122107574 | 1.39046364977610 | 0.80242097266662 |
| H | -3.78937436473158 | 0.81593926752215 | -0.02416299749445 |
| H | -3.88308280552100 | 2.35016768340913 | 0.86851263147807 |
| H | -3.47957813869707 | 0.83799050920897 | 1.73534894027015 |
| O | -2.03066152922461 | 2.80492955304862 | -1.35096229997543 |
| O | -1.17411923169392 | 0.55206506190118 | 2.52426328300737 |
| C | 1.81484126098361 | 1.89440680012695 | 3.41662541011343 |
| C | 1.46279935817730 | 0.89446526832307 | 4.51471434848801 |
| H | 2.80872379952041 | 2.32535921468151 | 3.57754722651383 |
| H | 1.10020893524884 | 2.71999354418390 | 3.37259345411143 |
| H | 2.18640769184776 | 0.07360336826483 | 4.54454672539680 |
| H | 1.47976642519159 | 1.39375277937758 | 5.49153046148785 |
| H | 0.46984323228172 | 0.47491698652854 | 4.34430500769937 |
| C | 0.62633054042694 | 3.11338142550482 | -2.53080489859675 |
| C | 2.32491088261128 | 3.29889998364245 | -0.78791676463629 |
| H | 2.49099596159132 | 3.22927576092831 | 0.28541962224006 |
| C | 2.58921968556188 | 4.72475650933600 | -1.28173657297680 |
| H | 3.01517039235446 | 2.60105862181981 | -1.28384359711209 |
| H | -0.42387585562300 | 2.89802858376172 | -2.70926433969980 |
| H | 1.25051944003625 | 2.41595054489777 | -3.10931240091753 |

| | | | |
|---|---|---|---|
| C | 0.95816134862733 | 4.54405017074702 | -2.95158486578065 |
| O | 2.31368475892951 | 4.86641380929819 | -2.66642089207309 |
| H | 3.64522690301393 | 4.97600091297311 | -1.14333813776639 |
| H | 1.97981627954208 | 5.43251208256305 | -0.69517476193617 |
| H | 0.27926738059305 | 5.24388899807724 | -2.43805552388651 |
| H | 0.82889792833766 | 4.65919450129782 | -4.03170169417129 |

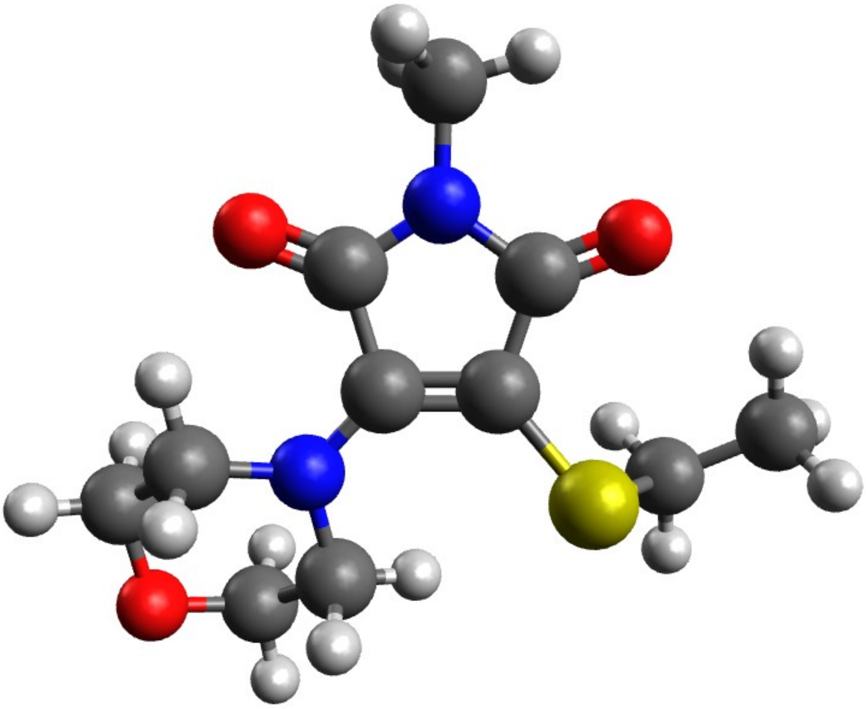

FINAL SINGLE POINT ENERGY -1161.556297498746 (Eh)

| 3c-3 | | | |
|---|---|---|---|
| C | -1.26714057191478 | 2.00302865949791 | -0.71350773715593 |
| C | 0.24774129790790 | 2.18055786664978 | -0.50416903874120 |
| N | -1.69301353084686 | 1.12047802100407 | 0.24493344851851 |
| C | -0.61010009037245 | 0.68560920370759 | 1.04757958583813 |
| C | 0.60408895700786 | 1.36134462540303 | 0.55162837583327 |
| S | 2.11992985373340 | 1.19329951571402 | 1.44368606036395 |
| N | 0.96745738782176 | 2.99208707285535 | -1.31503891189183 |
| C | -3.05589138525447 | 0.63889048164203 | 0.37023809325363 |
| H | -3.37755312864805 | 0.14025259390753 | -0.54896215675973 |
| H | -3.73939160404535 | 1.46746633016048 | 0.57657384031992 |
| H | -3.07082289024964 | -0.06739079515953 | 1.20124255216184 |
| O | -1.97955112089666 | 2.49859965260075 | -1.57183959425575 |
| O | -0.73470427080119 | -0.11014006846526 | 1.96098993550980 |
| C | 2.43975761658870 | -0.62768927167784 | 1.32375443019029 |
| C | 3.68054019016631 | -0.97911517213517 | 2.14149310835019 |
| H | 2.57225313860578 | -0.88659650283824 | 0.26909230104954 |
| H | 1.55225119182973 | -1.13342544616070 | 1.70825547852252 |
| H | 3.87241464101801 | -2.05553094047383 | 2.07939572504373 |
| H | 4.56987799113465 | -0.45730091338520 | 1.77258472654265 |
| H | 3.54630163599792 | -0.72102716748417 | 3.19649219887198 |
| C | 0.39043122731321 | 4.00150314670022 | -2.21912365487633 |
| C | 2.42996344010851 | 2.91915705941645 | -1.43282962822781 |
| H | 2.82748969360176 | 2.22425655146697 | -0.69451737653641 |
| C | 3.04703749448688 | 4.30667153508414 | -1.24700607779400 |
| H | 2.67076698639438 | 2.54808220538717 | -2.43946937237310 |
| H | -0.68050361877604 | 4.08271913071498 | -2.05526789614372 |
| H | 0.55288312102657 | 3.68077792079430 | -3.25771591921218 |

| | | | |
|---|---|---|---|
| C | 1.08688453023136 | 5.34536714855971 | -1.99086696993711 |
| O | 2.49407827971411 | 5.24756406864777 | -2.15631628717659 |
| H | 4.12216877816613 | 4.26940433471218 | -1.44609801089724 |
| H | 2.89473802928191 | 4.64089840084995 | -0.20747844055506 |
| H | 0.84529608147245 | 5.71885117608045 | -0.98157820819500 |
| H | 0.73372064819616 | 6.07526957622305 | -2.72562457964096 |

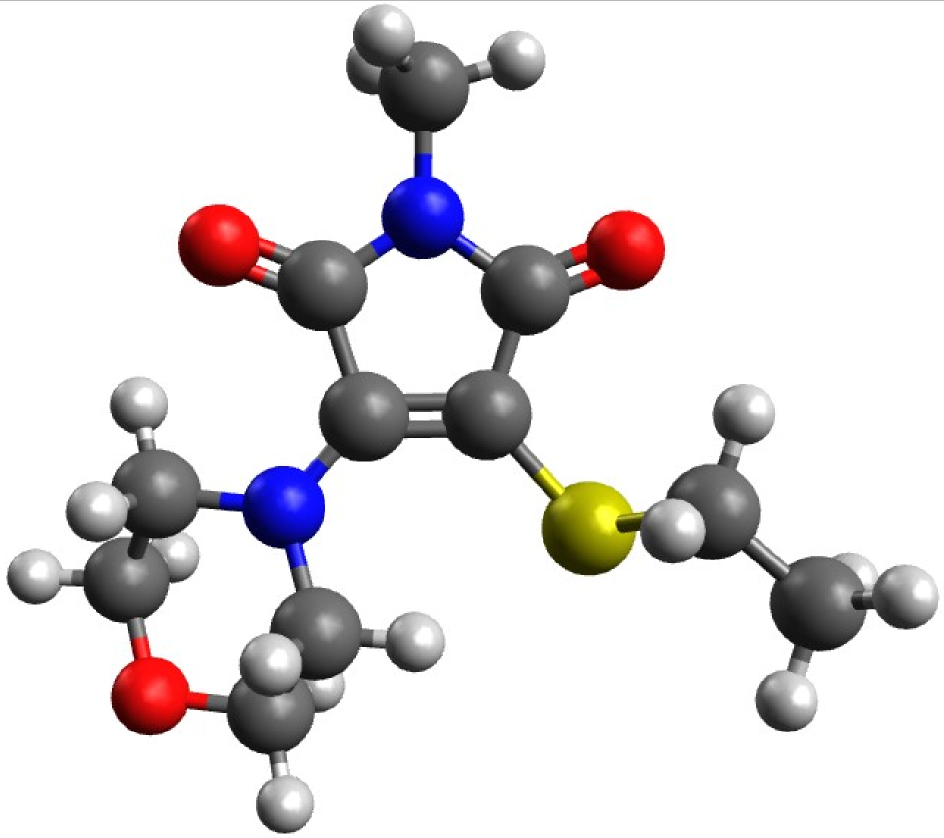

FINAL SINGLE POINT ENERGY -1161.558967764660 (Eh)

| 3c-4 | | | |
|---|---|---|---|
| C | -1.30508105331459 | 2.17596542062092 | -0.40996163927674 |
| C | 0.23323807180845 | 2.23475396608637 | -0.43023620159176 |
| N | -1.65003724496298 | 1.32816865534424 | 0.61027438122204 |
| C | -0.49604724971208 | 0.80429983776545 | 1.24298431817762 |
| C | 0.67900321304755 | 1.38263124803356 | 0.56365747719854 |
| S | 2.29444227893200 | 1.08463245966240 | 1.21444055287972 |
| N | 0.88206364492243 | 2.98973013769516 | -1.34797760119469 |
| C | -3.01184332012102 | 0.95108540002603 | 0.93951506744669 |
| H | -3.50222855590602 | 0.47968797674215 | 0.08252587321587 |
| H | -3.59296088477774 | 1.82891584256499 | 1.23603937528555 |
| H | -2.95735262872962 | 0.24672668575497 | 1.77046827723273 |
| O | -2.09642716291477 | 2.72625369354993 | -1.15891913347983 |
| O | -0.54382045469471 | 0.01779357462413 | 2.17151461725958 |
| C | 2.44305735046290 | -0.75441430471734 | 1.04754949512401 |
| C | 3.76413100618723 | -1.21481775309605 | 1.65969786091749 |
| H | 2.38703123730384 | -1.01091065401236 | -0.01452443364984 |
| H | 1.58890881105069 | -1.19238276701535 | 1.56722394519962 |
| H | 3.85285158542860 | -2.30291911414793 | 1.57279896369336 |
| H | 4.62424522852447 | -0.76575133619278 | 1.15199314124921 |
| H | 3.82095766438367 | -0.95591128613193 | 2.72148545220218 |
| C | 0.26403201705365 | 4.07194156675219 | -2.13254337789190 |
| C | 2.30489184223130 | 2.82038050924357 | -1.67152302728291 |
| H | 2.74638745731218 | 2.06682636623816 | -1.02118434695800 |
| C | 3.04332878813169 | 4.15274749934081 | -1.52618503562261 |
| H | 2.37536199161714 | 2.47967568133314 | -2.71429536090394 |
| H | -0.76749443983646 | 4.21704798321398 | -1.82363334578126 |
| H | 0.26463892163983 | 3.78325335321611 | -3.19277188422523 |

| | | | |
|---|---|---|---|
| C | 1.08104709753250 | 5.35503983966776 | -1.95827302075244 |
| O | 2.44245610142723 | 5.16799177699883 | -2.31796849231131 |
| H | 4.07554597761848 | 4.05108779978589 | -1.87427995628110 |
| H | 3.05939340085071 | 4.45082431500804 | -0.46493901548902 |
| H | 1.00497847553714 | 5.70111867770452 | -0.91377731707212 |
| H | 0.68764083196629 | 6.13801694834037 | -2.61368560853950 |

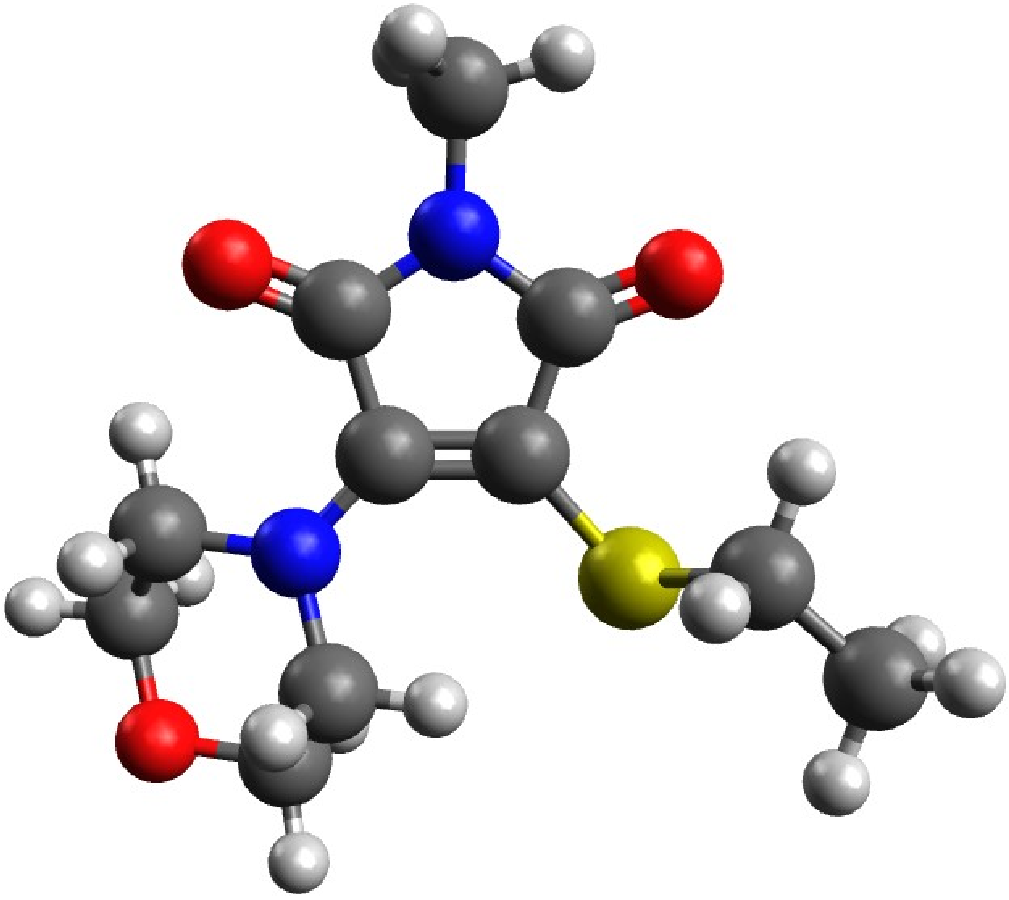

FINAL SINGLE POINT ENERGY -1161.558944122579 (Eh)

| 3c-5 | | | |
|---|---|---|---|
| C | -1.30290673010881 | 2.34077752525991 | -0.41176047409226 |
| C | 0.22855660747557 | 2.49195902424912 | -0.45468251972848 |
| N | -1.57852315395427 | 1.43500773524513 | 0.57948474287131 |
| C | -0.38484481101804 | 0.96116172219160 | 1.17619166027557 |
| C | 0.74239592944732 | 1.62964769156142 | 0.49744906275349 |
| S | 2.38431727814447 | 1.41014034615534 | 1.11607656530818 |
| N | 0.81453788875532 | 3.32541017079023 | -1.34686094596760 |
| C | -2.91001902630145 | 0.96847437544426 | 0.91801490862623 |
| H | -3.38519786236769 | 0.49019115955741 | 0.05622224375910 |
| H | -3.53753921803969 | 1.80208860621584 | 1.24570052218526 |
| H | -2.80015384048173 | 0.24759336545774 | 1.72910321358149 |
| O | -2.14026476987369 | 2.87138960414380 | -1.12385050938110 |
| O | -0.37008343378082 | 0.14488421931983 | 2.07993984376329 |
| C | 2.63142263634465 | -0.41527543397356 | 0.94353682123219 |
| C | 2.71491167495309 | -0.91027575889126 | -0.49681152596804 |
| H | 3.56721030199006 | -0.59692013484178 | 1.48237565364784 |
| H | 1.82398678137515 | -0.89750111128073 | 1.49784363268886 |
| H | 2.84659591280883 | -1.99873720276031 | -0.51422427218227 |
| H | 3.55699236607481 | -0.45652713152525 | -1.02903254308374 |
| H | 1.79729392506786 | -0.67659633966900 | -1.04599536584500 |
| C | 0.11513825305845 | 4.38883040145536 | -2.08826363917518 |
| C | 2.24154193865167 | 3.26756967360185 | -1.68980271967561 |
| H | 2.74331272428078 | 2.52418591952727 | -1.07272981111458 |
| C | 2.88753651084139 | 4.64234208388890 | -1.50333198270881 |
| H | 2.32343368478415 | 2.96924514518810 | -2.74471269309585 |
| H | -0.92078603262120 | 4.45171379047170 | -1.76705477717084 |
| H | 0.12633681783448 | 4.13727349415178 | -3.15791161257243 |

| | | | |
|---|---|---|---|
| C | 0.84246757719868 | 5.71905452138308 | -1.87510581061373 |
| O | 2.20963030236503 | 5.64144661264058 | -2.25184564466377 |
| H | 3.92049010219662 | 4.62464184830658 | -1.86337064440817 |
| H | 2.89492759290979 | 4.90269187193465 | -0.43198794408240 |
| H | 0.75309007010714 | 6.02191854738042 | -0.81825100936881 |
| H | 0.38807200188205 | 6.49503365741991 | -2.49863242579412 |

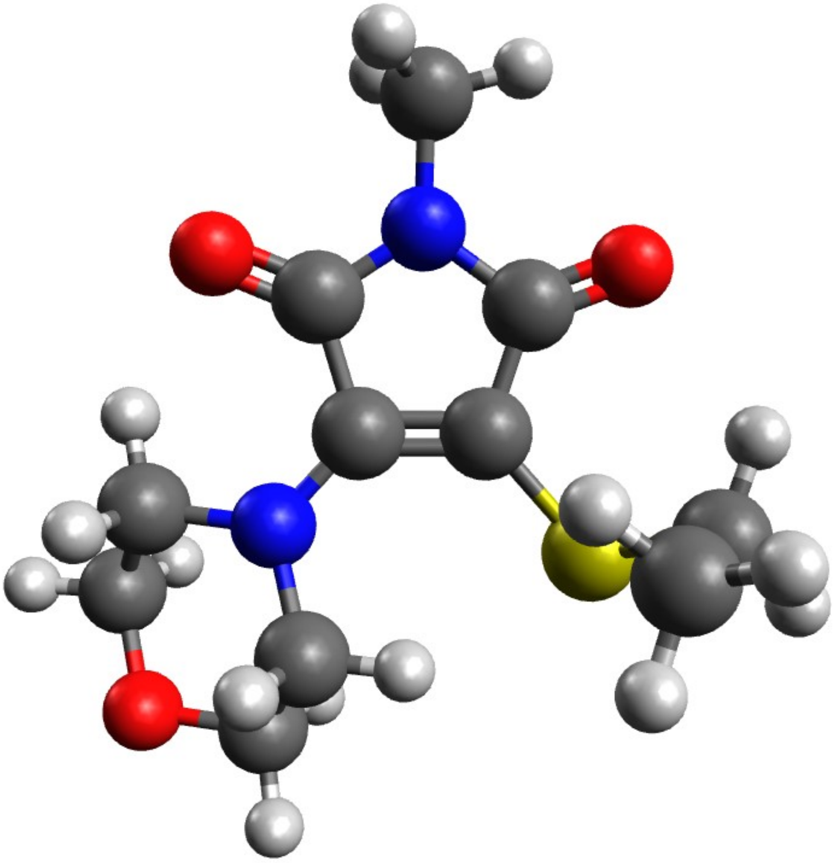

FINAL SINGLE POINT ENERGY -1161.559775080507 (Eh)

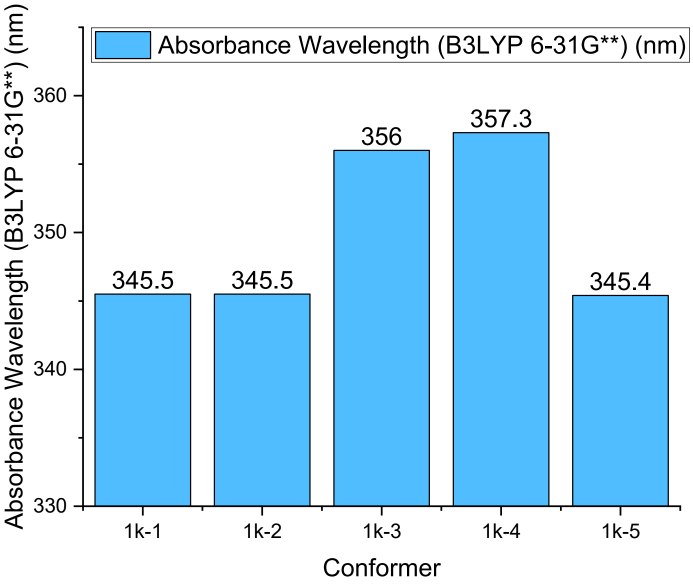


*Figure 139: Bar chart of the predicted absorbance of* ***1K*** *conformers, calculated at B3LYP 6-31G** level of theory (nm).*

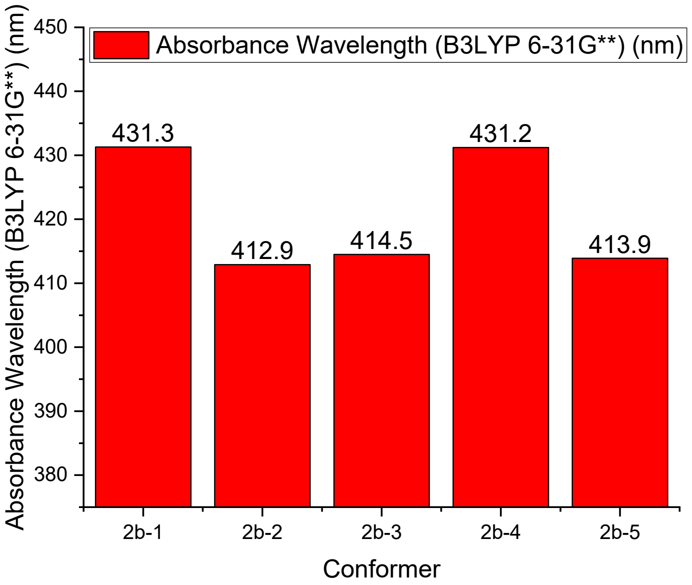


*Figure 140: Bar chart of the predicted absorbance of* ***2B*** *conformers, calculated at B3LYP 6-31G** level of theory (nm).*

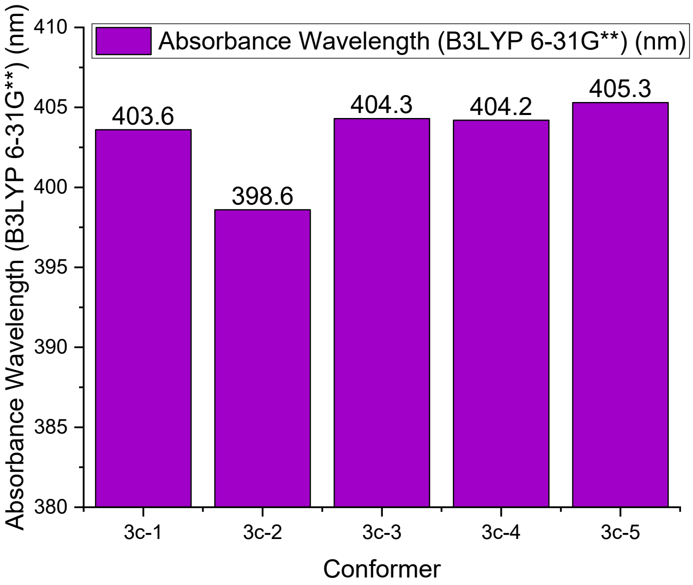


*Figure 141: Bar chart of the predicted absorbance of **3C** conformers, calculated at B3LYP 6-31G** level of theory (nm).*

# Appendix- Functional/Basis Set Combinations

The TD-DFT methodology and results heavily depend on the level of theory applied to the calculations. Due to this potential variation, we opted to run the TD-DFT absorbance calculations at three different levels of theory and compare these to our ANN-determined absorbance values. The basis set and functional choice reflect those popularised in literature for TD-DFT calculations. For the AMs, we observed that no basis set/functional combination outperformed the ANN predictions, but that B3LYP 6-31G** provided the closest values overall. As previously mentioned, the ANN performance was poorer than B3LYP 6-31G** for the DTMs. We observed similar performance between B3LYP 6-31G** and PBE0 def2 TZVP, but B3LYP 6-31G** ultimately yielded a smaller scatter about the experimental "zero line". As for the ATMs, the performance across basis sets was mixed, but B3LYP 6-31G** once more provided the smallest scatter about the "zero line". We consistently observed a ~50 nm underperformance of the ωB97X-D aug-cc-pVDZ functional/basis set combination across all maleimide derivative classes.

## Aminomaleimides (AMs)

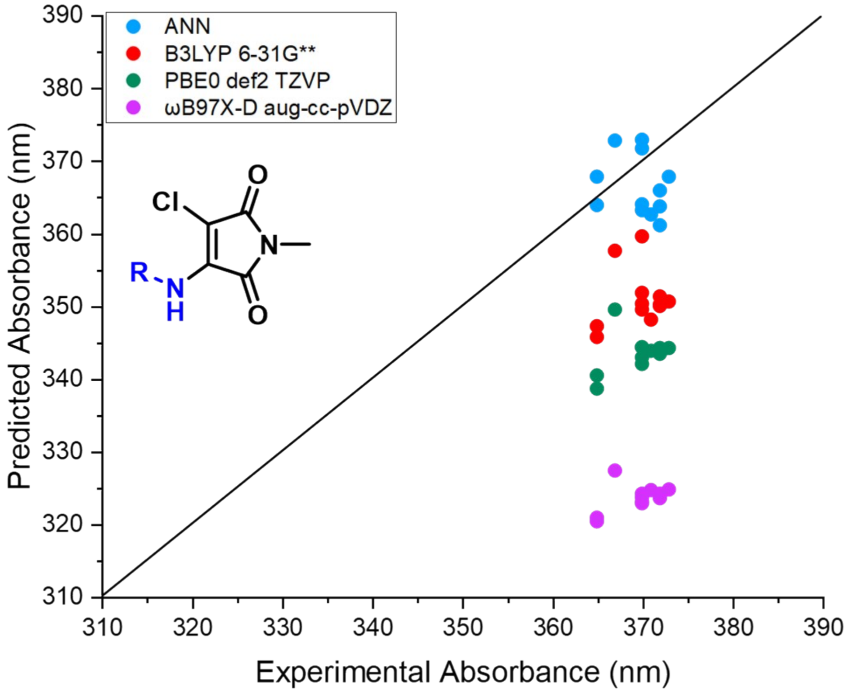


*Figure 142: Experimental vs predicted absorbance of AMs, using various basis sets.*

# Dithiomaleimides (DTMs)

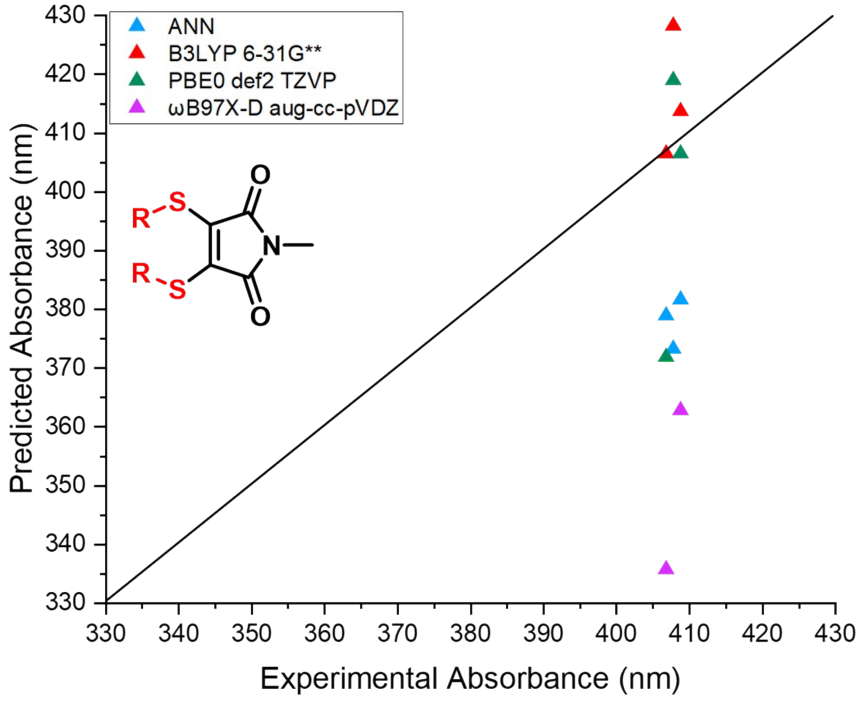


*Figure 143: Experimental vs predicted absorbance of DTMs, using various basis sets.*

# Aminothiomaleimides (ATMs)

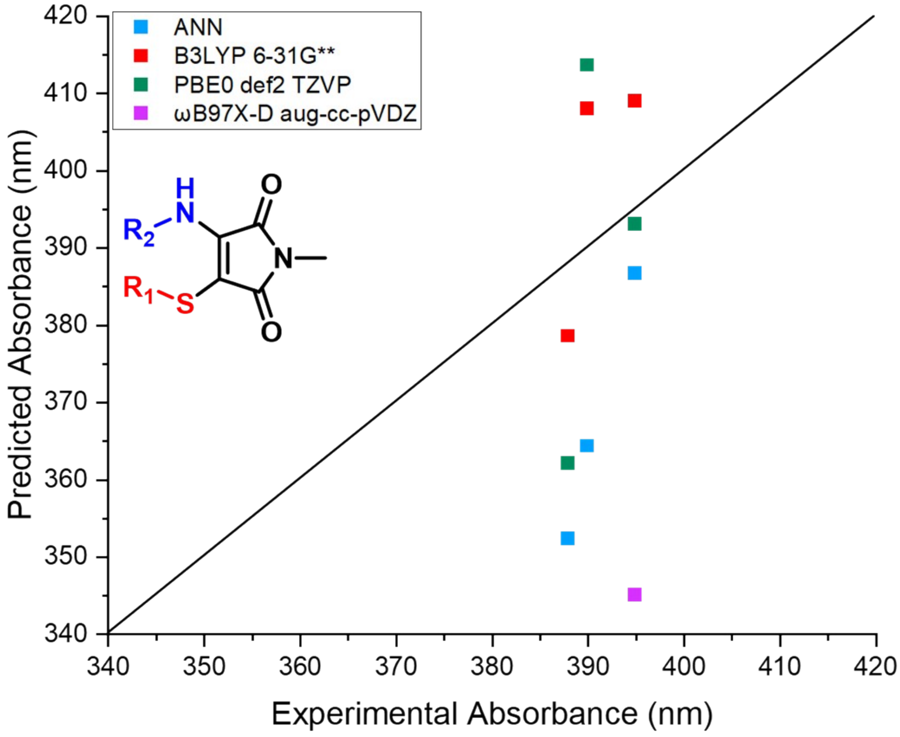


*Figure 144: Experimental vs predicted absorbance of ATMs, using various basis sets.*
